# Digital Twin Modeling for Landslide Risk Scenarios in Mountainous Regions

**DOI:** 10.3390/s26020421

**Published:** 2026-01-08

**Authors:** Lai Li, Bohui Tang, Fangliang Cai, Lei Wei, Xinming Zhu, Dong Fan

**Affiliations:** 1 Faculty of Land and Resources Engineering, Kunming University of Science and Technology, Kunming 650093, China; 20232201085@stu.kust.edu.cn (L.L.); caifangliang@stu.kust.edu.cn (F.C.);; 2 Yunnan Key Laboratory of Quantitative Remote Sensing, Kunming 650093, China; 3 Yunnan International Joint Laboratory for Integrated Sky-Ground Intelligent Monitoring of Mountain Hazards, Kunming 650093, China; 4 State Key Laboratory of Resources and Environment Information System, Institute of Geographic Sciences and Natural Resources Research, Chinese Academy of Sciences, Beijing 100101, China; 5 Yunnan Institute of Geo-Environment Monitoring, Kunming 650216, China

**Keywords:** rain-induced landslide, digital twins, displacement mutation sensitive zone, landslide monitoring, Multiphysics coupling

## Abstract

**Highlights:**

A novel 3D digital twin framework for landslides is developed using a closest-packed spherical discrete element model. The point-contact mechanism of spherical elements simplifies friction modeling and enhances computational efficiency. Steady-state slope stress exhibits a trapezoidal distribution, increasing from the surface inwards. The stress increases uniformly from 0 kPa at the surface to 210 kPa in the innermost region.

**What are the main findings?**

- A sensitive zone of the landslide mass was identified in the numerical simulations. Both stress and displacement results consistently showed the highest concentration at one-tenth of the slope height above the toe, rather than at the crest or base.
- Advanced Simulation Framework: The novel 3D digital twin approach improves computational efficiency and enables realistic landslide evolution mapping.

**What are the implications of the main findings?**

- This finding offers a new perspective for future research, as the identified stress and displacement concentration zone provides a tangible focus for further investigation.
- Advanced Predictive Modeling: The 3D digital twin framework provides a more stable method for simulating landslides. This approach introduces a physics-driven framework, effectively addressing the lack of realistic physical parameters in conventional hazard scenario modeling.

**Abstract:**

**Background**: Rainfall-induced landslides are a widespread and destructive geological hazard that resist precise prediction. They pose serious threats to human lives and property, ecological stability, and socioeconomic development. **Methods**: To address the challenges in mitigating rainfall-induced landslides in high-altitude mountainous regions, this study proposes a digital twin framework that couples multiple physical fields and is based on the spherical discrete element method. **Results**: Two-dimensional simulations identify a trapezoidal stress distribution with inward-increasing stress. The stress increases uniformly from 0 kPa at the surface to 210 kPa in the interior. The crest stress remains constant at 1.8 kPa under gravity, whereas the toe stress rises from 6.5 to 14.8 kPa with the slope gradient. While the stress pattern persists post-failure, specific magnitudes alter significantly. This study pioneers a three-dimensional close-packed spherical discrete element method, achieving enhanced computational efficiency and stability through streamlined contact mechanics. **Conclusions**: The proposed framework utilizes point-contact mechanics to simplify friction modeling, enhancing computational efficiency and numerical stability. By integrating stress, rainfall, and seepage fields, we establish a coupled hydro-mechanical model that enables real-time digital twin mapping of landslide evolution through dynamic parameter adjustments.

## 1. Introduction

The concept of the Digital Twin originated in industry, evolving from the digital mapping of physical products to broader applications such as digital twin cities, watersheds, and a digital twin of the Earth [1]. Digital twin technology constructs a virtual counterpart of a physical entity, enabling real-time data analysis throughout its lifecycle to support informed decision-making. Its implementation necessitates creating a precise digital replica that captures the entity’s physical attributes, operational states, and dynamic behaviors within complex real-world environments, effectively establishing a high-fidelity virtual mirror. This model allows researchers to perform in-depth analysis, prediction, and optimization of the entity’s performance. The technological foundations of digital twins date back to the 1960s [2], when the National Aeronautics and Space Administration (NASA) developed simulation systems to address communication and control challenges in space exploration. These systems could simulate spacecraft operations in real-time and exchange data with the physical vehicles, forming an early prototype of digital twin technology [3,4]. The term “Digital Twin” was formally coined by American computer scientist Michael Grieves in a 2002 academic lecture, establishing a theoretical foundation for its development [5]. Rapid advancements in computing, particularly the emergence of cloud computing, big data, the Internet of Things (IoT), and Artificial Intelligence (AI) [6,7], have provided critical support for the technology’s progress.

Mature cloud computing offers efficient computational resources and storage capabilities for digital twin models. Big data technologies provide the means for processing and analyzing massive datasets, uncovering their underlying value [8,9]. IoT enables real-time data transmission between physical objects and their virtual counterparts, ensuring timely and accurate information. AI supplies key algorithms for intelligent analysis and decision-making [10]. The synergistic development of these technologies has propelled digital twins from a theoretical concept to practical application, continually expanding their scope. Today, digital twins have transcended simple digital replication of physical objects to become complex systems that integrate real-time and historical data, simulation, and intelligent algorithms [11,12,13,14,15]. Such systems not only optimize the performance of physical entities but also enable predictive maintenance by identifying potential failures in advance, providing managers with scientific and rational decision-support and facilitating intelligent transformation and high-quality development across various industries [16,17].

Landslides, as the most prominent type of geological hazard in mountainous regions, pose a severe threat to human life and property. The complex topography, fragile geotechnical structures, and adverse climatic conditions such as heavy rainfall make these areas highly prone to landslides [18,19,20]. According to the Department of Natural Resources of Yunnan Province, by the end of 2020, the province alone had 23,267 registered geological hazard sites. These included 17,450 potential landslide sites, 2237 collapse sites, 3118 debris flow sites, 331 ground collapse sites, 21 land subsidence sites, and 110 ground fissure sites. Collectively, these hazards threaten the lives of 3.7804 million people, with potential economic losses estimated at 79.673 billion RMB. Research indicates that over 80% of landslides are triggered by rainfall. Traditional mitigation strategies—such as soil anchoring, slope surface reinforcement, and drainage systems—are often limited in effectiveness due to an insufficient understanding of landslide mechanisms [21,22]. Therefore, in-depth research on landslide initiation mechanisms and the development of more accurate and efficient prediction models are critical for enhancing prevention capabilities and safeguarding lives and property in mountainous regions. Current research directions in geological hazards increasingly demand physically interpretable models, which integrate multiple physical fields rather than relying on isolated properties [23,24]. This requires a multidisciplinary approach integrating geology, geomechanics, and meteorology, supported by modern information technology. Constructing a multi-source data-integrated digital twin framework for landslide hazards is essential to provide a scientific basis and technical support for their prevention and mitigation [25,26,27].

The development of digital-driven landslide models is essential for deciphering the initiation and evolution mechanisms of regional landslides and for enhancing the precision of mitigation strategies. Recent rapid advancements in digital technology and earth sciences have established digital geology as a cutting-edge focus in landslide research. Against this backdrop, this study aims to develop a digital-twin-driven framework for landslides and to explore its practical applications and value in landslide risk mitigation.

## 2. Materials and Methods

**Figure 1:**
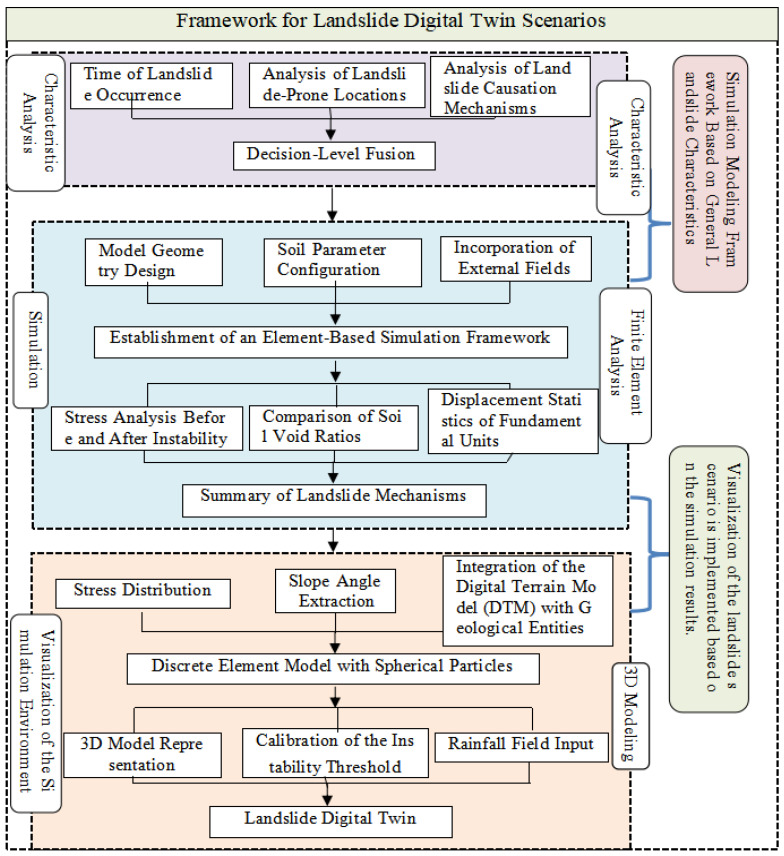
Flowchart of landslide digital twin framework.

Figure 1 outlines the workflow for developing the proposed landslide digital twin. The process begins by analyzing historical landslide characteristics and their failure mechanisms, which entails determining temporal frequency, mapping spatial distribution, and examining the failure process. These analyses form the foundational input for the digital twin’s decision-making core. The simulation phase involves three key steps: designing the model architecture, calibrating soil parameters, and constructing the simulation domain. This process establishes a robust framework for the subsequent analysis, which yields key parameters—such as the stress field, void ratio, and displacement field—under both stable conditions and during progressive failure. This synthesis of parameter dynamics throughout the landslide incubation process forms the core logic of the digital twin. Finally, the simulated soil properties and stress distributions are incorporated—based on the extracted slope gradient—into the spherical particle model, constructing a foundational 3D scenario. This base is integrated with predefined instability thresholds and a rainfall field to complete the framework.

### 2.1. Characteristic Analysis

Landslides stem from diverse causes. Scholarly studies categorize primary triggers into: rainfall-induced, earthquake-triggered, natural evolution, freeze–thaw infiltration, underground excavation, slope cutting and unloading, engineering loading, reservoir immersion, irrigation seepage, and blast vibrations. Landslides triggered by meteorological precipitation are classified as rainfall-induced landslides, which represent the most prevalent, frequent, and hazardous form of water-related slope failure [28,29]. The mechanisms underlying such landslides are inherently complex and exhibit strong interactive characteristics. From a triggering perspective, failure often results from short-duration, high-intensity rainfall acting on slopes preconditioned by prolonged antecedent precipitation or cumulative rainfall.

Antecedent rainfall progressively elevates the moisture content within the slope body, thereby priming it for subsequent failure. Short-duration, intense rainfall then acts as the direct trigger by rapidly introducing substantial water into the slope, leading to a sharp deterioration in stability. This destabilization results from several physical processes, among which seepage plays a critical role: infiltrating rainwater increases the bulk unit weight of the slope materials. The infiltrated rainwater, being denser than air, fills the soil pores and increases the mass per unit volume, thereby raising the downward driving force. Consequently, rainfall-induced landslides are prone to initiate on slopes with pre-existing fractures. Concurrently, prolonged water infiltration softens the slope materials by altering the mineral composition and structure of the soil or rock, leading to a reduction in key mechanical properties such as shear strength. This weakening effect is particularly critical in potential slip zones, where strength degradation directly undermines slope stability. As these zones constitute potential failure surfaces, their mechanical deterioration markedly increases landslide susceptibility.

Furthermore, rainfall triggers a suite of mechanical responses that further destabilize slopes. A principal mechanism is the water-wedge effect in fissures: infiltrating rainwater penetrates pre-existing cracks within the soil or rock mass, exerting substantial pressure that propagates and widens fractures. Concurrently, seepage forces act on soil particles along the hydraulic gradient, while rising pore water pressure lowers the effective stress between particles—collectively reducing the material’s shear strength. The combined effect of these mechanical processes critically undermines slope stability.

### 2.2. Simulation

Simulation is a computational technique that digitally represents real-world entities and iteratively refines the results to approximate physical reality [30]. Its fundamental distinction from the Digital Twin concept lies in their core definitions: a Digital Twin refers to the comprehensive process of mapping a physical entity into a virtual space, while simulation represents a key output generated by this digital counterpart. Through simulation, it becomes feasible to explore complex, multi-factor scenarios that have not yet occurred in reality. Generally, simulations incorporating a greater number of influencing variables yield more accurate representations of real-world behavior.

This study utilized ABAQUS (2020) to perform simulations under the proposed landslide digital twin framework. As an advanced finite element analysis platform, ABAQUS exhibits strong capabilities in multi-physics coupling simulations for engineering applications [31]. The software offers an integrated solution system capable of handling both linear and nonlinear problems, with particular strengths in detailed element modeling and material constitutive relationships. In geometric modeling, ABAQUS provides an extensive element library containing over one hundred element types, supporting the discretization of highly complex geometries and enabling accurate meshing from one-dimensional beam elements to three-dimensional solid models [32].

Principal stresses derived from simulation are fundamental mechanical parameters in material mechanics and geotechnical engineering. These can be categorized into three types: the first principal stress (Max Principal, also termed major principal stress), the second principal stress (Mid Principal, or intermediate principal stress), and the third principal stress (Min Principal, i.e., minor principal stress). According to their fundamental definition and mathematical representation, the values of the three principal stresses decrease sequentially, as formulated in Equation (1). Separately, the von Mises stress (referred to hereafter as Mises stress) is a scalar measure of deviatoric stress energy commonly used to predict yielding in ductile materials. Directional stresses, also known as true stresses, possess explicit component representations in the Cartesian coordinate system. These comprise S11 (normal stress along the *X*-axis), S22 (normal stress along the *Y*-axis), and S33 (normal stress along the *Z*-axis). Shear stress components are also defined, including S12 (shear stress acting on the plane normal to the *X*-axis in the *Y*-direction), S13 (on the plane normal to the *X*-axis in the *Z*-direction), and S23 (on the plane normal to the *Y*-axis in the *Z*-direction).

(1) $$\sigma_{1}>\sigma_{2}>\sigma_{3}$$

(2) $$\begin{array}{l} \sigma_{e}=\frac{1}{\sqrt{2}}\sqrt{{\left(\sigma_{1}-\sigma_{2}\right)}^{2}+{\left(\sigma_{2}-\sigma_{3}\right)}^{2}+{\left(\sigma_{3}-\sigma_{1}\right)}^{2}} \end{array}$$

(3) $$\sigma_{e}=\frac{1}{\sqrt{2}}\sqrt{{\left(\sigma_{x}-\sigma_{y}\right)}^{2}+{\left(\sigma_{y}-\sigma_{z}\right)}^{2}+{\left(\sigma_{z}-\sigma_{x}\right)}^{2}+6(\tau_{xy}^{2}+\tau_{yz}^{2}+\tau_{zx}^{2})}$$

where *σ_e_* is the von Mises stress, *σ*_1_ is the first principal stress, *σ*_2_ is the second principal stress, *σ*_3_ is the third principal stress, *σ_x_* is the normal stress acting on the plane perpendicular to the *x*-axis, *σ_y_* is the normal stress acting on the plane perpendicular to the *y*-axis, *σ_z_* is the normal stress acting on the plane perpendicular to the *z*-axis, *τ_xy_* is the shear stress acting on the plane perpendicular to the *x*-axis and in the *y*-direction, *τ_yz_* is the shear stress acting on the plane perpendicular to the *y*-axis and in the *z*-direction, *τ_zx_* is the shear stress acting on the plane perpendicular to the *z*-axis and in the *x*-direction. The von Mises stress can be calculated from the three principal stresses using Equation (2). In the Cartesian coordinate system, it can also be expressed in terms of the directional stress components, as shown in Equation (3).

#### 2.2.1. Slope Model Configuration

The mechanism of rainfall-induced landslides begins with water infiltration, which raises the groundwater level inside the slope, softens the soil and rock mass along potential slip surfaces, and reduces the geotechnical strength. This degradation of mechanical properties diminishes slope stability and can ultimately lead to failure. Mechanistically, the process can be described as an interplay between driving and resisting forces. Increased soil saturation elevates the overall unit weight of the slope, thereby augmenting the driving force. Concurrently, matric suction dissipates, effective stress declines, and shear strength degrades—collectively weakening the resisting capacity. Slope instability occurs when the driving force surpasses the resisting force, resulting in failure [33,34,35,36]. For slopes with different inclination angles, the in situ stress distribution generally follows a consistent pattern.

**Figure 2:**
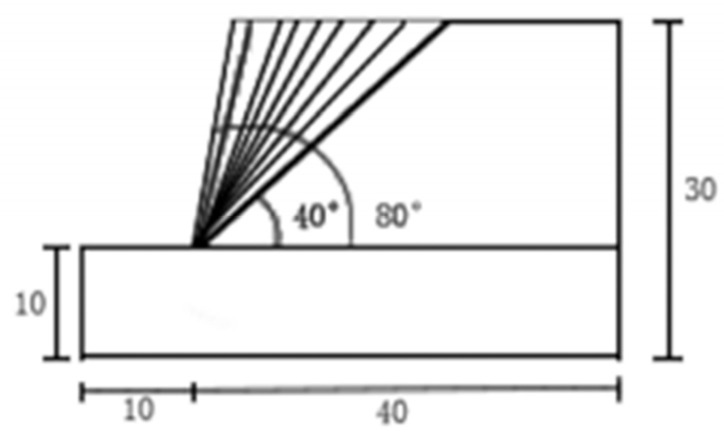
Two-dimensional simulation model.

However, under identical external conditions, the magnitude of stress varies considerably with the slope angle. While the stress at the slope crest remains largely governed by gravitational forces and shows negligible correlation with slope inclination, the stress distribution across the rest of the slope is highly sensitive to changes in angle. To systematically analyze these variations, this study performed numerical simulations on slopes with angles ranging from 40° to 80° at 5° intervals. The corresponding model configuration is shown in Figure 2. Within the established modeling framework, the lower 10-m section is defined as the foundation soil layer. Based on extensive experimental data, the soil water content at the 10-m depth of this layer is set to zero. Below this depth, the water content exhibits a consistent increasing trend with further depth. Above the foundation layer, a 20-m section is constructed as the slope structure to accurately represent realistic slope morphology.

#### 2.2.2. Key Soil Parameter Configuration

During the numerical simulations, preliminary tests indicated that excessively steep slopes induce complex and unstable force distributions within the granular soil structure, significantly compromising stability and frequently leading to non-convergent solutions. To address this issue and ensure computational accuracy and reliability, a differentiated effective cohesion strategy was adopted during parameter assignment, with values calibrated according to slope inclination. For slopes between 50° and 65°, the effective cohesion was set to 30 kPa to address the pronounced influence of steeper gradients on stability. In the extreme case of slopes ranging from 70° to 80°, where stability is most critically challenged, the effective cohesion was further raised to 40 kPa. A summary of the corresponding model parameter configurations is provided in Table 1.

The slope height was defined as 20 m in the constructed model. As a fundamental geometric parameter representing the vertical dimension of the slope, it directly governs the mechanical response under gravitational and external loading. The dry density was set to 1.3 g/cm^3^. This parameter characterizes the mass per unit volume of the soil in a completely dry state, reflects the degree of soil compactness, and significantly influences mechanical behavior. The initial void ratio was assigned a value of 1. As a key indicator of the soil’s initial pore structure, it governs permeability, compressibility, and strength properties, thereby defining the initial state of the slope. The deformation modulus was set to 30 MPa, representing the soil’s resistance to elastic deformation under load. This parameter directly affects the accuracy of simulated mechanical responses, including displacement and strain fields. Poisson’s ratio was defined as 0.3, reflecting the material’s lateral deformation behavior under axial loading and influencing stress–strain distribution within the slope.

Poisson’s ratio was set to 0.3. This parameter quantifies the ratio of transverse to axial strain under uniaxial stress conditions and is essential for realistically simulating deformation under complex stress states. The saturated permeability coefficient was assigned a value of 0.018 m/h, representing the soil’s water transport capacity in a saturated state. As a key parameter governing moisture movement during rainfall infiltration, it directly influences the variation in soil water content and the corresponding evolution of mechanical properties, thereby serving as a critical input for simulating seepage-stress coupling effects. An extreme rainstorm event is defined as one with an intensity reaching 0.02 m/h.

### 2.3. Three-Dimensional Engine-Based Terrain Representation

Unreal Engine, a leading 3D graphics platform, offers a comprehensive toolchain and high-fidelity rendering capabilities. Its high-performance rendering pipeline seamlessly integrates rigid body dynamics, soft-body deformation algorithms, and collision detection modules, supporting physically realistic scene simulation while maintaining high-frame-rate performance [37]. The engine’s distinctive Blueprint visual scripting system employs a node-based logic architecture that reduces the technical threshold for developing interactive systems, enabling developers to implement logic design and process control through intuitive drag-and-drop operations. Benefiting from an open architecture and cross-platform compatibility, Unreal Engine exhibits strong potential in digital twin visualization, industrial simulation, and Building Information Modeling (BIM) applications, establishing it as a preferred technical solution for building high-precision visualization systems [38,39,40].

Based on the simulation results, the digital twin core was constructed within the 3D engine. The three-dimensional terrain representation was generated by progressively stacking 2D simulation outcomes—a process that extends beyond mere dimensional expansion. It incorporates sophisticated algorithms and data processing techniques to accurately map two-dimensional information, such as stress and displacement, into 3D space, thereby representing slope morphology and mechanical states more realistically and intuitively.

Furthermore, an innovative methodology was introduced for digitally representing physical processes. This approach utilizes advanced mathematical models and numerical algorithms to simulate complex landslide-related phenomena, including soil deformation, failure mechanisms, and changes in pore water pressure due to rainfall infiltration. The method significantly reduces computational load while improving simulation efficiency.

#### 2.3.1. Fundamentals of the Particle Model

**Figure 3:**
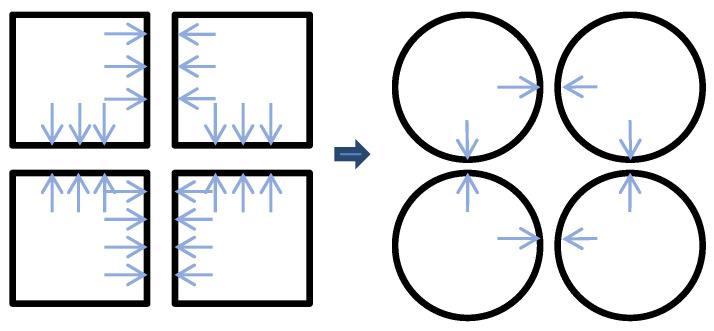
Two-dimensional Schematic of sphere–sphere and cube–cube contact.

The digitalization of natural resources serves as a foundational pillar for building a “Digital Twin Earth,” making unified, fine-grained representation a key future direction [41,42]. In this context, this study introduces a novel physics system representation method that departs from conventional cubic voxels by adopting spheres as the fundamental units to establish the modeling paradigm. In terms of contact behavior, face contact between cubic elements generates normal pressure (dominated by gravity) and tangential friction (dependent on material properties). In contrast, spherical elements interact through point contacts, a feature rooted in the theory of closest packing in three-dimensional space. In the densest arrangement, a single sphere can form point contacts with up to 12 spheres of equal volume, corresponding to a coordination number of 12. This geometric configuration inherently avoids the influence of contact surface friction, as only normal interactions between spheres need to be considered, as shown in Figure 3.

As illustrated in Figure 3, the point contact mechanism inherent to spherical primitives simplifies the representation of contact forces as a function of their physical properties—such as mass, radius, and elastic modulus—by circumventing complex friction behavior. This significantly improves the controllability of the modeling elements. Furthermore, compared to face contact models, the number of contact pairs in a point-contact network decreases dramatically. Combined with the ordered configuration of the closest packing structure, this effectively reduces the algorithmic complexity associated with contact detection in discrete element simulations, leading to a substantial reduction in computational cost. This modeling strategy establishes a foundational framework for digital twin systems that more closely approximate real physical scenarios, demonstrating considerable utility in fields requiring precise characterization of discrete element interactions, such as granular material mechanics and multi-body system dynamics.

#### 2.3.2. Three-Dimensional Terrain Scene Representation

**Figure 4:**
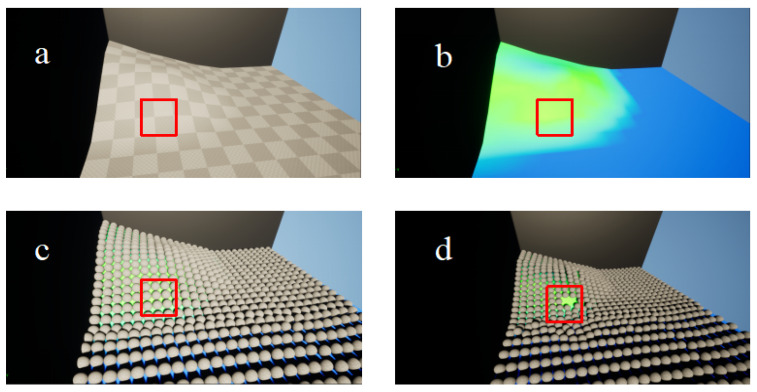
The construction process of the model scene. (**a**): Terrain texture mapping; (**b**): extraction of equal-slope points (color-coded with green indicating steeper gradients); (**c**): superposition of the spherical discrete element model; (**d**): visualization at simulation onset.

The landslide digital twin framework developed in this study employs spherical discrete elements as fundamental units, enabling dynamic simulation of real landslide processes through multi-physics field coupling. Uniformly sized spheres are arranged in a closest packing configuration to represent the terrain surface, where the elevation of each sphere—determined by its position and radius—accurately captures the undulating topography, thereby transforming the continuous surface into a discrete particle assembly. As shown in Figure 4, the implementation involves first constructing a 3D terrain base, from which slope points are extracted, and subsequently superimposing the particle model onto this structure.

At the physical field coupling level, the framework utilizes stress field data to drive the state evolution of spherical units. The stress distribution derived from numerical simulations is embedded into each sphere as an initial condition, establishing a stress–displacement relationship. A coupled hydrological model incorporating rainfall intensity and duration is introduced to dynamically simulate stress field variations induced by rainwater infiltration. This is achieved by adaptively adjusting sphere contact parameters, such as normal stiffness and gravitational coefficients. Finally, based on the contact force calculation model of the Discrete Element Method (DEM), the framework analyzes the displacement evolution of spherical units under the combined influence of self-weight, seepage force, and contact forces, thereby accomplishing the digital mapping from static terrain representation to dynamic landslide simulation [43,44].

### 2.4. Numerical Implementation and Reproducibility Details

The numerical simulation of landslides typically follows a sequential workflow comprising: geometry creation, material property definition, analysis step configuration, load application, and mesh generation. To enhance the reproducibility of this research, the following section details these operational steps in the aforementioned order, commencing from geometry creation and concluding with mesh generation.

#### 2.4.1. Step 1: Geometry Creation

As illustrated in Figure 3, the model consists of a rectangular base soil layer at the bottom with a total length of 50 m and a height of 10 m. The slope height is set to 20 m, with a 10 m buffer zone reserved at the front of the model. A series of simulations were conducted with slope angles increasing from 40° to 80° in increments of 5°. The overall model dimensions are 30 m in height and 50 m in length.

#### 2.4.2. Step 2: Material Property Definition

Preliminary simulations indicated that as the slope angle increases, the effective cohesion of the soil must be raised accordingly; otherwise, slope failure occurs prior to rainfall infiltration, leading to non-convergence of the simulation. The saturation-dependent function and pore-pressure saturation function were assigned typical empirical values (provided in the Supplementary Materials: Time-series data of simulated paradigmatic stress for slope failure instability). The remaining soil parameters are listed in Table 1.

#### 2.4.3. Step 3: Analysis Step Configuration

Two analysis steps were defined. In the first step, the pore fluid response was set to Steady-state, with a maximum of 100 increments, an initial increment size of 0.1, a minimum increment size of 0.0001, and a maximum increment size of 10. A direct solver with full Newton solution technique was employed. In the second step, the pore fluid response was switched to Transient consolidation.

#### 2.4.4. Step 4: Load Application

First, gravitational load was applied to the entire model. Second, a surface pore flow of −0.02 (corresponding to the rainfall intensity of 0.02 m/h listed in Table 1) was applied to the 10 m buffer zone at the front edge of the model. Finally, a pore flow equal to −0.02 multiplied by the cosine of the slope angle was applied to the slope surface.

#### 2.4.5. Step 5: Mesh Generation

The bottom 50 m section and the right-side 30 m section were discretized using a uniform element size of 1 m × 1 m. The remaining regions were automatically meshed by the solver’s built-in adaptive scheme.

**Figure 5:**
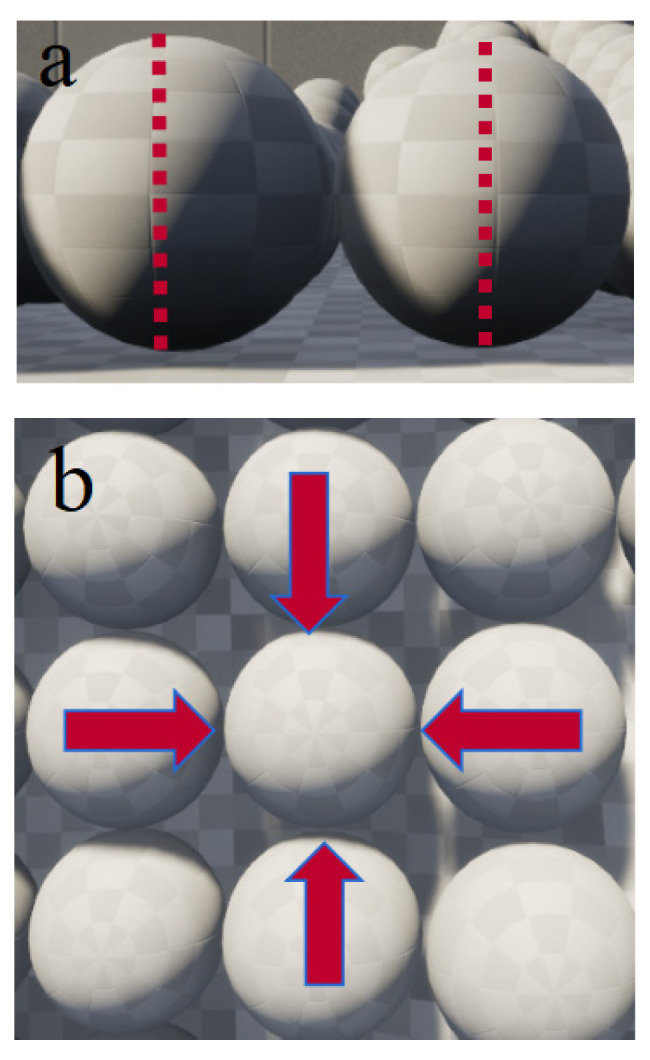
Three-Dimensional Close-packed aggregation rule. (**a**): terrain representation using spheres; (**b**): aggregation pattern of spheres.

In 3D scene modeling, the representation of surface morphology adheres to a unified set of core principles. After compiling a library of stress results from numerical simulations across various slope angles, these outcomes are incorporated into the 3D scene through spherical mass elements. In this study, each spherical unit is regarded as a representative entity of the surface properties within its diameter range. In addition to assigning gravitational attributes, a linear damping model is introduced, where the damping force increases with velocity to simulate dynamic responses such as collision and thrust forces experienced by entities in a physical environment. Using the stress values obtained from simulations, the linear damping coefficients are proportionally assigned for modeling. The actual stacking principle is illustrated in Figure 5.

## 3. Results

### 3.1. Stress Extraction and Analysis

The simulations generated detailed data on localized displacements, sectional void ratios, and multi-component stress states. The von Mises stress criterion—a widely recognized and effective method in material and geomechanics—provides an accurate and intuitive representation of complex shear-induced stress states [45,46]. Unlike conventional stress measures, it integrates all components of the stress tensor in its formulation [47,48], thereby accounting for the combined influence of tensile, compressive, and shear stresses on material response under diverse loading conditions. This holistic approach enables reliable prediction of material yield under complex stress states and aligns with the fourth strength theory in mechanics, also known as the maximum distortion energy theory [49,50]. Given these advantages, this study adopts the von Mises stress as the key metric, systematically examining its magnitude and distribution patterns across slopes with varying angles.

**Figure 6:**
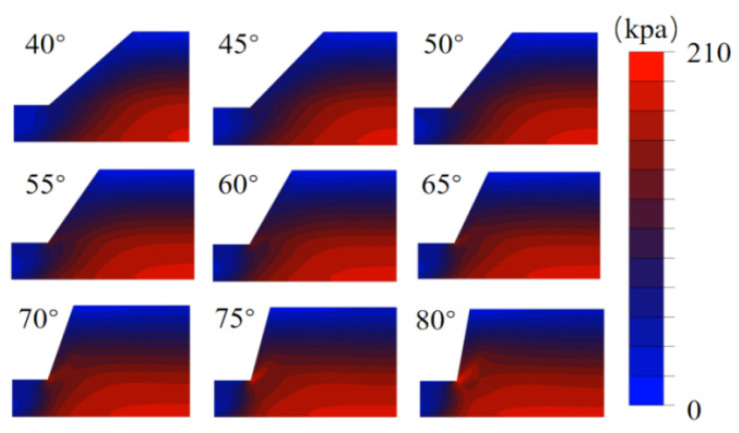
Simulation results of Von Mises Stress in the steady-state slope paradigm.

Figure 6 shows the simulated von Mises stress distribution of the slope under steady-state conditions. Analysis reveals a distinct trapezoidal spatial pattern, in which stress magnitudes increase progressively from the slope surface toward the interior. This distribution aligns closely with stress patterns observed in real-world geotechnical materials, thereby validating the model setup and confirming the authenticity and reliability of the simulation results.

**Figure 7:**
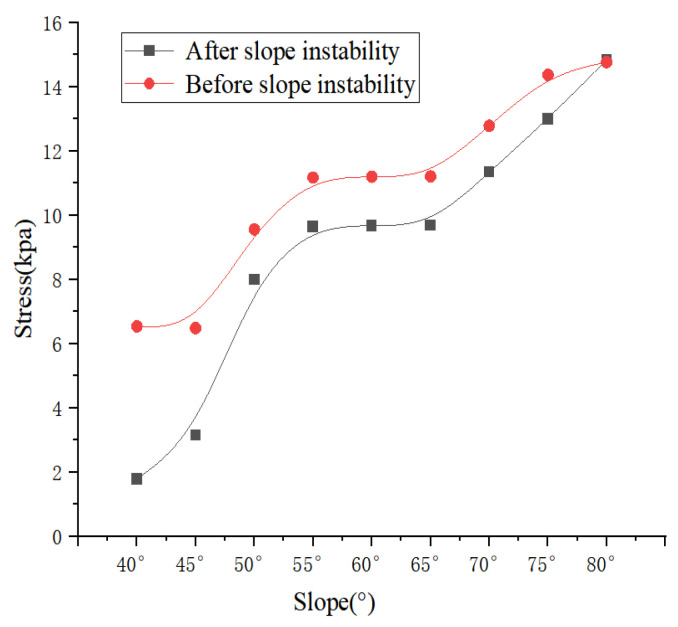
Comparison of stress variation pre- and post-slope failure at the 2-m height.

Analysis of surface stress characteristics reveals that stress at the slope crest remains largely insensitive to increases in slope angle (Figure 7). In contrast, stress at the slope toe shows high sensitivity to inclination. As shown in Figure 7, which illustrates stress distributions for slope heights ranging from 2 m to 20 m under pre-instability conditions, the toe stress exhibits a marked increase with slope angle. This behavior stems from geometric changes that modify force boundary conditions and internal stress transmission paths. Moreover, at a constant slope angle, a clear negative correlation is observed between stress magnitude and slope height; stress gradually decreases with increasing elevation, reflecting the attenuation of gravitational stress with height.

Building upon the steady-state slope model, instability was triggered by applying a rainfall field to investigate the post-failure stress state. As a common and destructive geological phenomenon, landslides significantly alter internal stress distributions. A thorough understanding of these changes offers a practical basis for subsequent landslide model development. Compared to the steady-state simulation, the post-landslide model introduced rainfall conditions as the primary variable. This section analyzes the characteristics of slope stress distribution after failure based on numerical results and compares them with the pre-failure steady state.

**Figure 8:**
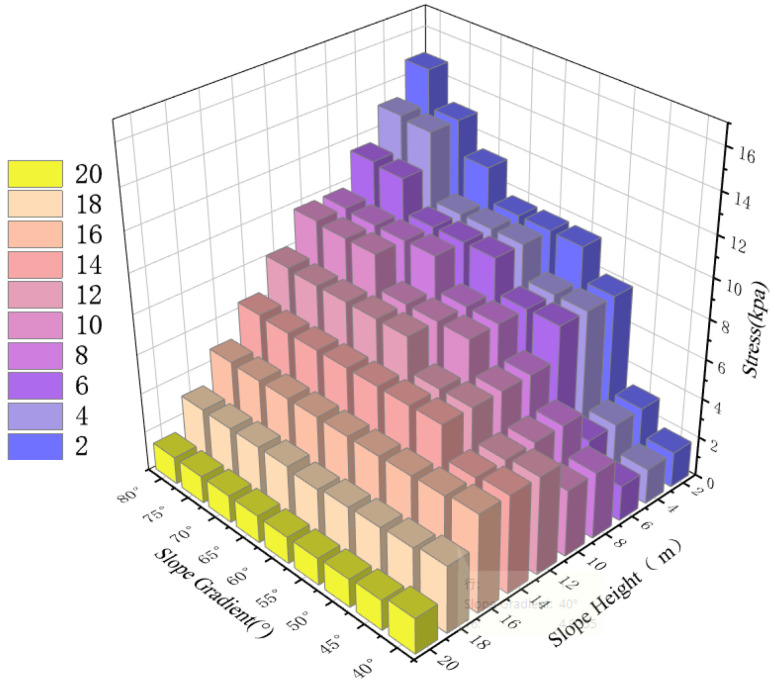
The stress conditions of unstable slopes with different gradients and different slope heights.

Analysis of the simulation results reveals significant stress redistribution in the shallow zone of the slope after instability failure, as visually summarized in Figure 8. With increasing slope angle, both shear and normal stresses at specific slope surface locations generally increase. This trend is particularly pronounced in the lower slope region (i.e., at lower elevations), where the slope toe bears greater gravitational and lateral pressures. As elevation approaches the crest, the stress gradient gradually attenuates and stabilizes, irrespective of slope angle. Quantitative analysis further indicates that surface stress amplitude decays with increasing elevation. These simulated patterns align closely with stress distributions predicted by soil mechanics principles [50], thereby validating the effectiveness and reliability of the proposed numerical model in simulating the evolution of the slope stress field.

Comparative analysis of stress field evolution before and after slope instability indicates that, regardless of the slope state (stable or failed), both the surface stress amplitude and the overall stress distribution pattern exhibit consistent trends with increasing slope angle. Specifically, for slopes of the same inclination, stress values increase with slope angle in both pre- and post-failure states—a trend visually confirmed in the stress distribution diagrams in Figure 8. The most notable difference in stress response before and after failure occurs at the 2-m height position. Combined with displacement vector analysis, this region (within the 2–4 m height range) experiences the most severe abrupt displacement, a phenomenon highly consistent with the expansion of the plastic zone during slope instability.

### 3.2. Accuracy Discussion

#### 3.2.1. Accuracy of the Numerical Simulation

**Figure 9:**
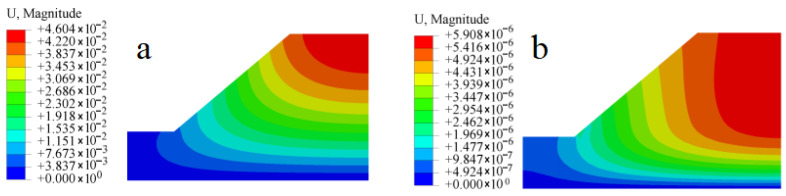
Displacement comparison pre- and post-geostatic stress equilibrium. (**a**): pre-equilibrium displacement state; (**b**): post-equilibrium displacement state.

Geostatic stress equilibrium is a critical step in the numerical simulation process, as its accuracy directly governs the physical realism of the simulation outcomes. In geotechnical and geological numerical analysis, the displacement response of a slope under initial in situ stress serves as a key indicator for assessing the plausibility of the simulation. As depicted in Figure 9a, before stress equilibrium was achieved, the maximum displacement under initial conditions reached an order of 10^−2^ meters (approximately 40 cm), which is inconsistent with actual geological settings. The slope under investigation—formed through long-term sedimentation—is expected to remain in a near-equilibrium state prior to rainfall-induced failure. Such substantial initial displacement contradicts realistic mechanical behavior.

To address this, a geostatic stress equilibrium procedure was applied to restore the slope’s stress field under natural conditions. After equilibrium, the maximum displacement was reduced to 0.005 cm, as shown in Figure 9b. This magnitude agrees well with the actual deformation characteristics expected in a stable slope, confirming that the initial stress field has been properly reconstructed. This step successfully eliminated non-physical deformations resulting from initial stress mismatch, thereby establishing a reliable initial state for subsequent simulation of rainfall infiltration and landslide evolution. As a result, the overall validity and predictive capacity of the numerical model have been assured.

Following geostatic stress equilibrium, a sensitivity analysis of key soil parameters in the numerical simulation was conducted. Since the ultimate trigger of slope failure in this study is sufficient rainfall intensity, parameters including dry density, deformation modulus, initial void ratio, and effective internal friction angle could be reasonably held constant. In contrast, Poisson’s ratio and effective cohesion were identified as the most influential factors affecting simulated stress and displacement outputs.

For typical plastic soils, Poisson’s ratio generally falls within the range of 0.3–0.35, while effective cohesion commonly varies between 10 kPa and 50 kPa. In this study, a controlled comparison was performed for slope angles of 40° and 80° by testing two values of Poisson’s ratio: 0.3 and 0.35.

At a Poisson’s ratio of 0.3, the maximum and minimum displacements were 0.005757 m and 0.0004798 m, respectively. When Poisson’s ratio was increased to 0.35, the corresponding displacements were 0.005597 m and 0.0004664 m. The difference between the two sets of results remained within 0.001 m. The data comparison is provided in Table 2.

However, at the steep slope angle of 80°, simulations with a Poisson’s ratio of 0.35 failed to converge. Therefore, a Poisson’s ratio of 0.3 is recommended for this model. This value not only aligns with the typical range for plastic soils but also ensures numerical stability and convergence across all slope configurations examined.

A sensitivity analysis was conducted on the effective cohesion, a key parameter governing soil shear strength. For plastic soils, effective cohesion typically ranges between 10 kPa and 50 kPa, with higher values indicating greater shear resistance.

Given that the present study commenced simulations from a slope angle of 40°, which already represents a relatively steep inclination, a lower bound of 10 kPa was deemed unsuitable. Accordingly, an initial value of 15 kPa was adopted. However, during simulations at 50°, convergence could not be achieved even when the cohesion was increased to 20 kPa. To maintain consistency in soil properties across simulations while ensuring numerical stability, the value was raised to 30 kPa.

A similar convergence issue reoccurred at a slope angle of 70°, necessitating a further increase in effective cohesion to 40 kPa. This stepwise adjustment ensured that the soil strength parameters remained physically plausible while allowing the numerical model to proceed without instability across the investigated range of slope angles.

#### 3.2.2. Model Accuracy

**Figure 10:**
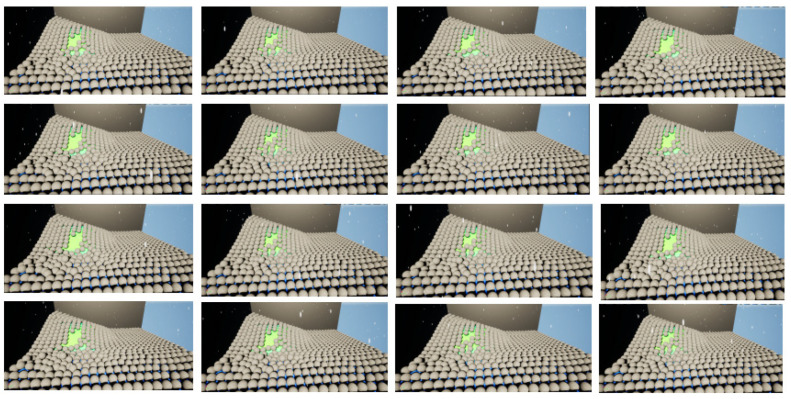
Comparison of experimental time scales.

Regarding the accuracy of the model, this paper discusses the response time of the landslide body and the displacement differences along the slip surface boundary. The experiment for landslide response time was set up as follows: using the workflow described previously, a scene was constructed as shown in the accompanying Figure 10. In this scene, all spheres were assigned stress values derived from numerical simulations. The model was started and stopped 42 times in total, with each run maintained for approximately 0.018 s (the time log of the experiment is provided in the accuracy section of the Supplementary Materials). The timing error was controlled within 0.001 s, and the screenshot angle was kept consistent throughout.

As illustrated, 16 experimental runs closest to the 0.018-s duration were extracted for analysis. It can be clearly observed that within 0.018 s, all landslide bodies had initiated sliding. Moreover, in these 16 experiments, the initial location of sliding consistently occurred at one-tenth of the slope height where the inclination was steepest—a result that aligns fully with the displacement outputs from the numerical simulations (see UH40–UH80 in the Supplementary Materials). This agreement further validates the consistency between the physical response observed in the experiments and the numerical predictions.

**Figure 11:**
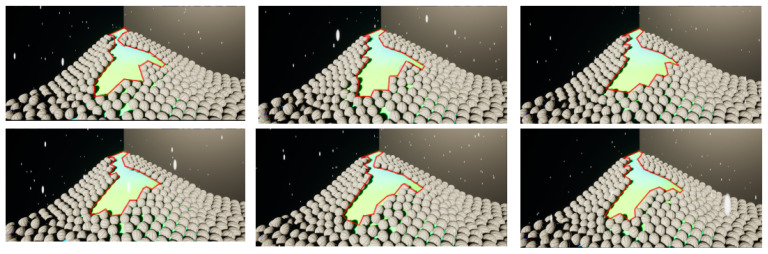
Comparison of experimental landslide boundaries.

In the comparative experiment on displacement at the landslide boundary, the same scene was used; however, the model was not terminated immediately after initiation. Instead, recordings were taken only after the landslide mass had come to a complete standstill. Six experimental runs were conducted, as shown in Figure 11, where the red frames represent the vector boundaries of displacement along the slip surface. The vector boundaries exhibited a shape similarity exceeding 80% across all six runs. Furthermore, the maximum offset positions among the six vector frames fell within a distance equivalent to two sphere diameters.

Overall, the response time of the landslide mass in the model was below the order of seconds. Moreover, comparison of the displacement differences along the slip surface boundary confirmed that the maximum displacement offset did not exceed two sphere diameters.

## 4. Discussion

In terms of numerical simulation, a key strength of the proposed model lies in its capability to simulate physical parameters across a range of slope angles. For digital twin scene modeling, the framework—constructed with spheres as basic units within a 3D digital twin environment—shows clear advantages over conventional landslide modeling approaches. The replacement of surface contacts (as in cubic elements) with point contacts between spheres significantly enhances computational efficiency. As demonstrated by the response tests in Section 3, the twin-scene reaction time is maintained below one second. Regarding terrain representation, compared to methods such as finite element analysis that employ cuboid units, the sphere-packing-based approach captures subsurface morphology and geological settings with higher precision. This is essential for revealing the spatiotemporal evolution of landslides and clarifying their initiation and triggering mechanisms, offering a novel perspective for refined terrain characterization.

Several limitations of the current framework should be acknowledged. In the numerical simulation stage, only one type of plastic soil was considered; extensive repeated simulations will be required in the future to compile the physical properties of all soil types, ultimately building a comprehensive library of slope parameters. In 3D scene modeling, although basic terrain representation was implemented, only a single soil type was incorporated, limiting the framework to small-scale, homogeneous digital twin scenarios. Modeling of slopes with integrated multiple soil types has not yet been addressed. Furthermore, achieving finer terrain detail necessitates a considerably larger number of spheres, which—despite the efficiency gained from point contacts—still demands substantial computational resources. Automation remains limited in both the numerical simulation and 3D modeling stages.

Future improvements could focus on the following directions: First, a systematic simulation library could be established to encompass physical properties for different soils and slope configurations, enabling direct retrieval of parameter sets. Second, an automated modeling module could be developed to generate sphere-based representations by directly importing topographic and material data from real-world scenes. Finally, as more field monitoring data become available, the model should be rigorously calibrated and validated to enhance the reliability of accuracy assessment and improve its predictive capability.

## 5. Conclusions

This study tackled the challenge of preventing and mitigating rainfall-induced landslides in mountainous regions by developing a multi-physics coupled digital twin framework based on a three-dimensional close-packed spherical discrete element method. The research systematically revealed the mechanical mechanisms and evolutionary patterns of landslide development, offering an innovative technical solution for accurate prediction and intelligent early warning.

Two-dimensional numerical simulations identified a trapezoidal stress distribution, showing an increase in steady-state stress from the slope surface inward. The stress at the crest, governed mainly by gravity, remained stable at approximately 1.8 kPa. In contrast, the stress at the slope toe increased from 6.5 kPa to 14.8 kPa as the slope angle rose from 40° to 80°, with stresses at different elevations exhibiting a positive correlation with slope inclination. After rainfall-induced instability, the overall stress distribution pattern remained similar, though magnitudes at specific locations changed significantly. A displacement mutation-sensitive zone was identified at approximately one-tenth of the slope height (2–4 m).

The study introduced the three-dimensional close-packed spherical discrete element method into landslide modeling, employing spheres as the fundamental units to represent slope materials. This approach offers a novel and effective methodology for simulating and analyzing landslide behavior under complex geological and hydrological conditions. The point-contact mechanism streamlined the conventional face-contact friction modeling by eliminating complex frictional effects and reducing contact force calculations to a function of the spheres’ physical properties, which significantly enhanced both simulation efficiency and numerical stability. Through the integration of stress, rainfall, and seepage fields, a coupled hydro-mechanical model was established, enabling real-time digital twin mapping of the landslide evolution process via dynamic adjustment of contact parameters. Leveraging Unreal Engine for 3D scene construction, the framework integrated 2D simulation data with 3D terrain, achieving end-to-end digital visualization spanning terrain characterization, stress evolution, and displacement mutation.

To address the challenge of preventing rainfall-induced landslides in mountainous regions, this study developed a digital-twin-driven framework for landslide simulation. Two-dimensional numerical simulations, conducted based on the geological setting, demonstrated a trapezoidal stress distribution within the slope, with values increasing from the surface inward. The stress at the crest remained governed primarily by gravity, whereas the stress at the toe rose markedly with slope inclination. Following rainfall-induced failure, the stress field in the shallow portion of the slope underwent clear redistribution, accompanied by heightened stress gradients in lower elevation areas. Furthermore, by employing a discrete element method with spherical units, the study extended the original 2D model into a three-dimensional representation. This advancement allows the digital twin to more closely approximate real physical behavior, improving the reliability of landslide simulation.

## Figures and Tables

**Table 1:** Key soil parameter configuration.

Parameter	Unit	Value
Slope Height	m	20
Dry Density	g/cm^3^	1.3
Deformation Modulus	MPa	30
Poisson’s Ratio		0.3
Saturated Permeability Coefficient	m/h	0.018
Initial Void Ratio		1
Effective Cohesion	kPa	15–40
Effective Internal Friction Angle		30
Rainfall Intensity Level		Extreme Rainstorm

**Table 2:** Comparison of Poisson’s ratio results.

Poisson’s Ratio	The Maximum Displacement	The Minimum Displacement
0.3	0.005757 m	0.0004798 m
0.35	0.005597 m	0.0004664 m

## Data Availability

The original contributions presented in this study are included in the article/Supplementary Materials.

## Supplementary Materials

The following supporting information can be downloaded at: https://www.mdpi.com/article/10.3390/s26020421/s1, Detailed naming rules are provided in the supplementary material.
