# Supplementary material for: Digital Twin Modeling for Landslide Risk Scenarios in Mountainous Regions
Source: Sensors (Basel). 2026 Jan 8;26(2):421. doi: 10.3390/s26020421 (PMC12845812; doi:10.3390/s26020421)
Supplement: Supplementary file 1 [file sensors-26-00421-s001.zip › Supplementary Materials/Materials in Sensitivity Analysis/runtime log.docx]

LogAudio: Display: Audio Occlusion Plugin: None (built-in).

LogAudioMixer: Display: Initializing audio mixer using platform API: 'XAudio2'

LogAudioMixer: Display: Using Audio Hardware Device 扬声器 (Realtek(R) Audio)

LogAudioMixer: Display: Initializing Sound Submixes...

LogAudioMixer: Display: Creating Master Submix 'MasterSubmixDefault'

LogAudioMixer: Display: Creating Master Submix 'MasterReverbSubmixDefault'

LogAudioMixer: FMixerPlatformXAudio2::StartAudioStream() called. InstanceID=41

LogAudioMixer: Display: Output buffers initialized: Frames=1024, Channels=2, Samples=2048, InstanceID=41

LogAudioMixer: Display: Starting AudioMixerPlatformInterface::RunInternal(), InstanceID=41

LogAudioMixer: Display: FMixerPlatformXAudio2::SubmitBuffer() called for the first time. InstanceID=41

LogInit: FAudioDevice initialized with ID 41.

LogAudio: Display: Audio Device (ID: 41) registered with world 'shili5'.

LogAudioMixer: Initializing Audio Bus Subsystem for audio device with ID 41

LogLoad: Game class is 'GameModeBase'

LogWorld: Bringing World /Game/UEDPIE_0_shili5.shili5 up for play (max tick rate 60) at 2025.12.10-19.23.31

LogWorld: Bringing up level for play took: 0.024953

LogOnline: OSS: Created online subsystem instance for: :Context_43

LogGameMode: FindPlayerStart: PATHS NOT DEFINED or NO PLAYERSTART with positive rating

PIE: 登陆的服务器

PIE: PIE总开始时间：0.179秒。

LogSlate: Updating window title bar state: overlay mode, drag disabled, window buttons hidden, title bar hidden

LogWorld: BeginTearingDown for /Game/UEDPIE_0_shili5

LogWorld: UWorld::CleanupWorld for shili5, bSessionEnded=true, bCleanupResources=true

LogSlate: InvalidateAllWidgets triggered. All widgets were invalidated

LogWorldMetrics: [UWorldMetricsSubsystem::Deinitialize]

LogWorldMetrics: [UWorldMetricsSubsystem::Clear]

LogPlayLevel: Display: Shutting down PIE online subsystems

LogSlate: InvalidateAllWidgets triggered. All widgets were invalidated

LogSlate: Updating window title bar state: overlay mode, drag disabled, window buttons hidden, title bar hidden

LogAudioMixer: Deinitializing Audio Bus Subsystem for audio device with ID 41

LogAudioMixer: FMixerPlatformXAudio2::StopAudioStream() called. InstanceID=41

LogAudioMixer: FMixerPlatformXAudio2::StopAudioStream() called. InstanceID=41

LogUObjectHash: Compacting FUObjectHashTables data took 0.70ms

LogPlayLevel: Display: Destroying online subsystem :Context_43

LogDebuggerCommands: Repeating last play command: 模拟

LogPlayLevel: PlayLevel: No blueprints needed recompiling

LogPlayLevel: Creating play world package: /Game/UEDPIE_0_shili5

LogPlayLevel: PIE: StaticDuplicateObject took: (0.051150s)

LogPlayLevel: PIE: Created PIE world by copying editor world from /Game/shili5.shili5 to /Game/UEDPIE_0_shili5.shili5 (0.051182s)

LogUObjectHash: Compacting FUObjectHashTables data took 0.64ms

LogWorldMetrics: [UWorldMetricsSubsystem::Initialize]

LogRenderer: SceneCulling instance hierarchy is disabled as UseNanite(PCD3D_SM5) returned false, for scene: 'World /Game/UEDPIE_0_shili5.shili5'.

LogPlayLevel: PIE: World Init took: (0.001192s)

LogAudio: Display: Creating Audio Device: Id: 42, Scope: Unique, Realtime: True

LogAudioMixer: Display: Audio Mixer Platform Settings:

LogAudioMixer: Display: Sample Rate: 48000

LogAudioMixer: Display: Callback Buffer Frame Size Requested: 1024

LogAudioMixer: Display: Callback Buffer Frame Size To Use: 1024

LogAudioMixer: Display: Number of buffers to queue: 2

LogAudioMixer: Display: Max Channels (voices): 32

LogAudioMixer: Display: Number of Async Source Workers: 0

LogAudio: Display: AudioDevice MaxSources: 32

LogAudio: Display: Audio Spatialization Plugin: None (built-in).

LogAudio: Display: Audio Reverb Plugin: None (built-in).

LogAudio: Display: Audio Occlusion Plugin: None (built-in).

LogAudioMixer: Display: Initializing audio mixer using platform API: 'XAudio2'

LogAudioMixer: Display: Using Audio Hardware Device 扬声器 (Realtek(R) Audio)

LogAudioMixer: Display: Initializing Sound Submixes...

LogAudioMixer: Display: Creating Master Submix 'MasterSubmixDefault'

LogAudioMixer: Display: Creating Master Submix 'MasterReverbSubmixDefault'

LogAudioMixer: FMixerPlatformXAudio2::StartAudioStream() called. InstanceID=42

LogAudioMixer: Display: Output buffers initialized: Frames=1024, Channels=2, Samples=2048, InstanceID=42

LogAudioMixer: Display: Starting AudioMixerPlatformInterface::RunInternal(), InstanceID=42

LogAudioMixer: Display: FMixerPlatformXAudio2::SubmitBuffer() called for the first time. InstanceID=42

LogInit: FAudioDevice initialized with ID 42.

LogAudio: Display: Audio Device (ID: 42) registered with world 'shili5'.

LogAudioMixer: Initializing Audio Bus Subsystem for audio device with ID 42

LogLoad: Game class is 'GameModeBase'

LogWorld: Bringing World /Game/UEDPIE_0_shili5.shili5 up for play (max tick rate 60) at 2025.12.10-19.23.36

LogWorld: Bringing up level for play took: 0.024701

LogOnline: OSS: Created online subsystem instance for: :Context_44

LogGameMode: FindPlayerStart: PATHS NOT DEFINED or NO PLAYERSTART with positive rating

PIE: 登陆的服务器

PIE: PIE总开始时间：0.177秒。

LogSlate: Updating window title bar state: overlay mode, drag disabled, window buttons hidden, title bar hidden

LogWorld: BeginTearingDown for /Game/UEDPIE_0_shili5

LogWorld: UWorld::CleanupWorld for shili5, bSessionEnded=true, bCleanupResources=true

LogSlate: InvalidateAllWidgets triggered. All widgets were invalidated

LogWorldMetrics: [UWorldMetricsSubsystem::Deinitialize]

LogWorldMetrics: [UWorldMetricsSubsystem::Clear]

LogPlayLevel: Display: Shutting down PIE online subsystems

LogSlate: InvalidateAllWidgets triggered. All widgets were invalidated

LogSlate: Updating window title bar state: overlay mode, drag disabled, window buttons hidden, title bar hidden

LogAudioMixer: Deinitializing Audio Bus Subsystem for audio device with ID 42

LogAudioMixer: FMixerPlatformXAudio2::StopAudioStream() called. InstanceID=42

LogAudioMixer: FMixerPlatformXAudio2::StopAudioStream() called. InstanceID=42

LogUObjectHash: Compacting FUObjectHashTables data took 0.63ms

LogPlayLevel: Display: Destroying online subsystem :Context_44

LogDebuggerCommands: Repeating last play command: 模拟

LogPlayLevel: PlayLevel: No blueprints needed recompiling

LogPlayLevel: Creating play world package: /Game/UEDPIE_0_shili5

LogPlayLevel: PIE: StaticDuplicateObject took: (0.052473s)

LogPlayLevel: PIE: Created PIE world by copying editor world from /Game/shili5.shili5 to /Game/UEDPIE_0_shili5.shili5 (0.052506s)

LogUObjectHash: Compacting FUObjectHashTables data took 0.71ms

LogWorldMetrics: [UWorldMetricsSubsystem::Initialize]

LogRenderer: SceneCulling instance hierarchy is disabled as UseNanite(PCD3D_SM5) returned false, for scene: 'World /Game/UEDPIE_0_shili5.shili5'.

LogPlayLevel: PIE: World Init took: (0.001223s)

LogAudio: Display: Creating Audio Device: Id: 43, Scope: Unique, Realtime: True

LogAudioMixer: Display: Audio Mixer Platform Settings:

LogAudioMixer: Display: Sample Rate: 48000

LogAudioMixer: Display: Callback Buffer Frame Size Requested: 1024

LogAudioMixer: Display: Callback Buffer Frame Size To Use: 1024

LogAudioMixer: Display: Number of buffers to queue: 2

LogAudioMixer: Display: Max Channels (voices): 32

LogAudioMixer: Display: Number of Async Source Workers: 0

LogAudio: Display: AudioDevice MaxSources: 32

LogAudio: Display: Audio Spatialization Plugin: None (built-in).

LogAudio: Display: Audio Reverb Plugin: None (built-in).

LogAudio: Display: Audio Occlusion Plugin: None (built-in).

LogAudioMixer: Display: Initializing audio mixer using platform API: 'XAudio2'

LogAudioMixer: Display: Using Audio Hardware Device 扬声器 (Realtek(R) Audio)

LogAudioMixer: Display: Initializing Sound Submixes...

LogAudioMixer: Display: Creating Master Submix 'MasterSubmixDefault'

LogAudioMixer: Display: Creating Master Submix 'MasterReverbSubmixDefault'

LogAudioMixer: FMixerPlatformXAudio2::StartAudioStream() called. InstanceID=43

LogAudioMixer: Display: Output buffers initialized: Frames=1024, Channels=2, Samples=2048, InstanceID=43

LogAudioMixer: Display: Starting AudioMixerPlatformInterface::RunInternal(), InstanceID=43

LogAudioMixer: Display: FMixerPlatformXAudio2::SubmitBuffer() called for the first time. InstanceID=43

LogInit: FAudioDevice initialized with ID 43.

LogAudio: Display: Audio Device (ID: 43) registered with world 'shili5'.

LogAudioMixer: Initializing Audio Bus Subsystem for audio device with ID 43

LogLoad: Game class is 'GameModeBase'

LogWorld: Bringing World /Game/UEDPIE_0_shili5.shili5 up for play (max tick rate 60) at 2025.12.10-19.23.39

LogWorld: Bringing up level for play took: 0.025226

LogOnline: OSS: Created online subsystem instance for: :Context_45

LogGameMode: FindPlayerStart: PATHS NOT DEFINED or NO PLAYERSTART with positive rating

PIE: 登陆的服务器

PIE: PIE总开始时间：0.175秒。

LogSlate: Updating window title bar state: overlay mode, drag disabled, window buttons hidden, title bar hidden

LogWorld: BeginTearingDown for /Game/UEDPIE_0_shili5

LogWorld: UWorld::CleanupWorld for shili5, bSessionEnded=true, bCleanupResources=true

LogSlate: InvalidateAllWidgets triggered. All widgets were invalidated

LogWorldMetrics: [UWorldMetricsSubsystem::Deinitialize]

LogWorldMetrics: [UWorldMetricsSubsystem::Clear]

LogPlayLevel: Display: Shutting down PIE online subsystems

LogSlate: InvalidateAllWidgets triggered. All widgets were invalidated

LogSlate: Updating window title bar state: overlay mode, drag disabled, window buttons hidden, title bar hidden

LogAudioMixer: Deinitializing Audio Bus Subsystem for audio device with ID 43

LogAudioMixer: FMixerPlatformXAudio2::StopAudioStream() called. InstanceID=43

LogAudioMixer: FMixerPlatformXAudio2::StopAudioStream() called. InstanceID=43

LogUObjectHash: Compacting FUObjectHashTables data took 0.69ms

LogPlayLevel: Display: Destroying online subsystem :Context_45

LogDebuggerCommands: Repeating last play command: 模拟

LogPlayLevel: PlayLevel: No blueprints needed recompiling

LogPlayLevel: Creating play world package: /Game/UEDPIE_0_shili5

LogPlayLevel: PIE: StaticDuplicateObject took: (0.051084s)

LogPlayLevel: PIE: Created PIE world by copying editor world from /Game/shili5.shili5 to /Game/UEDPIE_0_shili5.shili5 (0.051117s)

LogUObjectHash: Compacting FUObjectHashTables data took 0.66ms

LogWorldMetrics: [UWorldMetricsSubsystem::Initialize]

LogRenderer: SceneCulling instance hierarchy is disabled as UseNanite(PCD3D_SM5) returned false, for scene: 'World /Game/UEDPIE_0_shili5.shili5'.

LogPlayLevel: PIE: World Init took: (0.001334s)

LogAudio: Display: Creating Audio Device: Id: 44, Scope: Unique, Realtime: True

LogAudioMixer: Display: Audio Mixer Platform Settings:

LogAudioMixer: Display: Sample Rate: 48000

LogAudioMixer: Display: Callback Buffer Frame Size Requested: 1024

LogAudioMixer: Display: Callback Buffer Frame Size To Use: 1024

LogAudioMixer: Display: Number of buffers to queue: 2

LogAudioMixer: Display: Max Channels (voices): 32

LogAudioMixer: Display: Number of Async Source Workers: 0

LogAudio: Display: AudioDevice MaxSources: 32

LogAudio: Display: Audio Spatialization Plugin: None (built-in).

LogAudio: Display: Audio Reverb Plugin: None (built-in).

LogAudio: Display: Audio Occlusion Plugin: None (built-in).

LogAudioMixer: Display: Initializing audio mixer using platform API: 'XAudio2'

LogAudioMixer: Display: Using Audio Hardware Device 扬声器 (Realtek(R) Audio)

LogAudioMixer: Display: Initializing Sound Submixes...

LogAudioMixer: Display: Creating Master Submix 'MasterSubmixDefault'

LogAudioMixer: Display: Creating Master Submix 'MasterReverbSubmixDefault'

LogAudioMixer: FMixerPlatformXAudio2::StartAudioStream() called. InstanceID=44

LogAudioMixer: Display: Output buffers initialized: Frames=1024, Channels=2, Samples=2048, InstanceID=44

LogAudioMixer: Display: Starting AudioMixerPlatformInterface::RunInternal(), InstanceID=44

LogAudioMixer: Display: FMixerPlatformXAudio2::SubmitBuffer() called for the first time. InstanceID=44

LogInit: FAudioDevice initialized with ID 44.

LogAudio: Display: Audio Device (ID: 44) registered with world 'shili5'.

LogAudioMixer: Initializing Audio Bus Subsystem for audio device with ID 44

LogLoad: Game class is 'GameModeBase'

LogWorld: Bringing World /Game/UEDPIE_0_shili5.shili5 up for play (max tick rate 60) at 2025.12.10-19.23.45

LogWorld: Bringing up level for play took: 0.025277

LogOnline: OSS: Created online subsystem instance for: :Context_46

LogGameMode: FindPlayerStart: PATHS NOT DEFINED or NO PLAYERSTART with positive rating

PIE: 登陆的服务器

PIE: PIE总开始时间：0.181秒。

LogSlate: Updating window title bar state: overlay mode, drag disabled, window buttons hidden, title bar hidden

LogWorld: BeginTearingDown for /Game/UEDPIE_0_shili5

LogWorld: UWorld::CleanupWorld for shili5, bSessionEnded=true, bCleanupResources=true

LogSlate: InvalidateAllWidgets triggered. All widgets were invalidated

LogWorldMetrics: [UWorldMetricsSubsystem::Deinitialize]

LogWorldMetrics: [UWorldMetricsSubsystem::Clear]

LogPlayLevel: Display: Shutting down PIE online subsystems

LogSlate: InvalidateAllWidgets triggered. All widgets were invalidated

LogSlate: Updating window title bar state: overlay mode, drag disabled, window buttons hidden, title bar hidden

LogAudioMixer: Deinitializing Audio Bus Subsystem for audio device with ID 44

LogAudioMixer: FMixerPlatformXAudio2::StopAudioStream() called. InstanceID=44

LogAudioMixer: FMixerPlatformXAudio2::StopAudioStream() called. InstanceID=44

LogUObjectHash: Compacting FUObjectHashTables data took 0.68ms

LogPlayLevel: Display: Destroying online subsystem :Context_46

LogEditorViewport: Clicking Background

Cmd: TRANSACTION UNDO

LogEditorTransaction: Undo 点击背景

LogEditorViewport: Clicking Background

LogAssetEditorSubsystem: Opening Asset editor for Blueprint /Game/shili4/sf_1.sf_1

LogRenderer: SceneCulling instance hierarchy is disabled as UseNanite(PCD3D_SM5) returned false, for scene: 'World /Engine/Transient.World_3'.

LogStaticMesh: Display: 正在构建静态网格体PhAT_FloorBox（所需内存估计：0.024887MB）…

LogDerivedDataCache: Display: ZenLocal: Error response received from PutCacheRecords RPC: from POST http://[::1]:8558//z$/$rpc -> 507

LogStaticMesh: Built static mesh [0.00s] /Engine/EditorMeshes/PhAT_FloorBox.PhAT_FloorBox

LogDerivedDataCache: Display: ZenLocal: Error response received from PutCacheValues RPC: from POST http://[::1]:8558//z$/$rpc -> 507

LogDerivedDataCache: Display: ZenLocal: Error response received from PutCacheRecords RPC: from POST http://[::1]:8558//z$/$rpc -> 507

LogDerivedDataCache: Display: ZenLocal: Error response received from PutCacheRecords RPC: from POST http://[::1]:8558//z$/$rpc -> 507

LogDerivedDataCache: Display: ZenLocal: Error response received from PutCacheRecords RPC: from POST http://[::1]:8558//z$/$rpc -> 507

LogDerivedDataCache: Display: ZenLocal: Error response received from PutCacheValues RPC: from POST http://[::1]:8558//z$/$rpc -> 507

LogSlate: Window 'sf_1' being destroyed

LogWorld: UWorld::CleanupWorld for World_3, bSessionEnded=true, bCleanupResources=true

LogSlate: InvalidateAllWidgets triggered. All widgets were invalidated

LogUObjectHash: Compacting FUObjectHashTables data took 2.00ms

LogEOSSDK: LogEOS: Updating Product SDK Config, Time: 2850.456543

LogEOSSDK: LogEOS: SDK Config Product Update Request Completed - No Change

LogEOSSDK: LogEOS: ScheduleNextSDKConfigDataUpdate - Time: 2850.806396, Update Interval: 315.170746

LogDebuggerCommands: Repeating last play command: 模拟

LogPlayLevel: PlayLevel: No blueprints needed recompiling

LogPlayLevel: Creating play world package: /Game/UEDPIE_0_shili5

LogPlayLevel: PIE: StaticDuplicateObject took: (0.047151s)

LogPlayLevel: PIE: Created PIE world by copying editor world from /Game/shili5.shili5 to /Game/UEDPIE_0_shili5.shili5 (0.047187s)

LogUObjectHash: Compacting FUObjectHashTables data took 1.34ms

LogWorldMetrics: [UWorldMetricsSubsystem::Initialize]

LogRenderer: SceneCulling instance hierarchy is disabled as UseNanite(PCD3D_SM5) returned false, for scene: 'World /Game/UEDPIE_0_shili5.shili5'.

LogPlayLevel: PIE: World Init took: (0.001294s)

LogAudio: Display: Creating Audio Device: Id: 45, Scope: Unique, Realtime: True

LogAudioMixer: Display: Audio Mixer Platform Settings:

LogAudioMixer: Display: Sample Rate: 48000

LogAudioMixer: Display: Callback Buffer Frame Size Requested: 1024

LogAudioMixer: Display: Callback Buffer Frame Size To Use: 1024

LogAudioMixer: Display: Number of buffers to queue: 2

LogAudioMixer: Display: Max Channels (voices): 32

LogAudioMixer: Display: Number of Async Source Workers: 0

LogAudio: Display: AudioDevice MaxSources: 32

LogAudio: Display: Audio Spatialization Plugin: None (built-in).

LogAudio: Display: Audio Reverb Plugin: None (built-in).

LogAudio: Display: Audio Occlusion Plugin: None (built-in).

LogAudioMixer: Display: Initializing audio mixer using platform API: 'XAudio2'

LogAudioMixer: Display: Using Audio Hardware Device 扬声器 (Realtek(R) Audio)

LogAudioMixer: Display: Initializing Sound Submixes...

LogAudioMixer: Display: Creating Master Submix 'MasterSubmixDefault'

LogAudioMixer: Display: Creating Master Submix 'MasterReverbSubmixDefault'

LogAudioMixer: FMixerPlatformXAudio2::StartAudioStream() called. InstanceID=45

LogAudioMixer: Display: Output buffers initialized: Frames=1024, Channels=2, Samples=2048, InstanceID=45

LogAudioMixer: Display: Starting AudioMixerPlatformInterface::RunInternal(), InstanceID=45

LogAudioMixer: Display: FMixerPlatformXAudio2::SubmitBuffer() called for the first time. InstanceID=45

LogInit: FAudioDevice initialized with ID 45.

LogAudio: Display: Audio Device (ID: 45) registered with world 'shili5'.

LogAudioMixer: Initializing Audio Bus Subsystem for audio device with ID 45

LogLoad: Game class is 'GameModeBase'

LogWorld: Bringing World /Game/UEDPIE_0_shili5.shili5 up for play (max tick rate 60) at 2025.12.10-19.24.46

LogWorld: Bringing up level for play took: 0.025105

LogOnline: OSS: Created online subsystem instance for: :Context_48

LogGameMode: FindPlayerStart: PATHS NOT DEFINED or NO PLAYERSTART with positive rating

PIE: 登陆的服务器

PIE: PIE总开始时间：0.187秒。

LogSlate: Updating window title bar state: overlay mode, drag disabled, window buttons hidden, title bar hidden

LogWorld: BeginTearingDown for /Game/UEDPIE_0_shili5

LogWorld: UWorld::CleanupWorld for shili5, bSessionEnded=true, bCleanupResources=true

LogSlate: InvalidateAllWidgets triggered. All widgets were invalidated

LogWorldMetrics: [UWorldMetricsSubsystem::Deinitialize]

LogWorldMetrics: [UWorldMetricsSubsystem::Clear]

LogPlayLevel: Display: Shutting down PIE online subsystems

LogSlate: InvalidateAllWidgets triggered. All widgets were invalidated

LogAudio: Display: Audio Device unregistered from world 'None'.

LogAudioMixer: Deinitializing Audio Bus Subsystem for audio device with ID 45

LogAudioMixer: FMixerPlatformXAudio2::StopAudioStream() called. InstanceID=45

LogAudioMixer: FMixerPlatformXAudio2::StopAudioStream() called. InstanceID=45

LogSlate: Updating window title bar state: overlay mode, drag disabled, window buttons hidden, title bar hidden

LogUObjectHash: Compacting FUObjectHashTables data took 1.34ms

LogPlayLevel: Display: Destroying online subsystem :Context_48

LogDebuggerCommands: Repeating last play command: 模拟

LogPlayLevel: PlayLevel: No blueprints needed recompiling

LogPlayLevel: Creating play world package: /Game/UEDPIE_0_shili5

LogPlayLevel: PIE: StaticDuplicateObject took: (0.050352s)

LogPlayLevel: PIE: Created PIE world by copying editor world from /Game/shili5.shili5 to /Game/UEDPIE_0_shili5.shili5 (0.050392s)

LogUObjectHash: Compacting FUObjectHashTables data took 1.47ms

LogWorldMetrics: [UWorldMetricsSubsystem::Initialize]

LogRenderer: SceneCulling instance hierarchy is disabled as UseNanite(PCD3D_SM5) returned false, for scene: 'World /Game/UEDPIE_0_shili5.shili5'.

LogPlayLevel: PIE: World Init took: (0.001291s)

LogAudio: Display: Creating Audio Device: Id: 46, Scope: Unique, Realtime: True

LogAudioMixer: Display: Audio Mixer Platform Settings:

LogAudioMixer: Display: Sample Rate: 48000

LogAudioMixer: Display: Callback Buffer Frame Size Requested: 1024

LogAudioMixer: Display: Callback Buffer Frame Size To Use: 1024

LogAudioMixer: Display: Number of buffers to queue: 2

LogAudioMixer: Display: Max Channels (voices): 32

LogAudioMixer: Display: Number of Async Source Workers: 0

LogAudio: Display: AudioDevice MaxSources: 32

LogAudio: Display: Audio Spatialization Plugin: None (built-in).

LogAudio: Display: Audio Reverb Plugin: None (built-in).

LogAudio: Display: Audio Occlusion Plugin: None (built-in).

LogAudioMixer: Display: Initializing audio mixer using platform API: 'XAudio2'

LogAudioMixer: Display: Using Audio Hardware Device 扬声器 (Realtek(R) Audio)

LogAudioMixer: Display: Initializing Sound Submixes...

LogAudioMixer: Display: Creating Master Submix 'MasterSubmixDefault'

LogAudioMixer: Display: Creating Master Submix 'MasterReverbSubmixDefault'

LogAudioMixer: FMixerPlatformXAudio2::StartAudioStream() called. InstanceID=46

LogAudioMixer: Display: Output buffers initialized: Frames=1024, Channels=2, Samples=2048, InstanceID=46

LogAudioMixer: Display: Starting AudioMixerPlatformInterface::RunInternal(), InstanceID=46

LogAudioMixer: Display: FMixerPlatformXAudio2::SubmitBuffer() called for the first time. InstanceID=46

LogInit: FAudioDevice initialized with ID 46.

LogAudio: Display: Audio Device (ID: 46) registered with world 'shili5'.

LogAudioMixer: Initializing Audio Bus Subsystem for audio device with ID 46

LogLoad: Game class is 'GameModeBase'

LogWorld: Bringing World /Game/UEDPIE_0_shili5.shili5 up for play (max tick rate 60) at 2025.12.10-19.25.11

LogWorld: Bringing up level for play took: 0.025191

LogOnline: OSS: Created online subsystem instance for: :Context_49

LogGameMode: FindPlayerStart: PATHS NOT DEFINED or NO PLAYERSTART with positive rating

PIE: 登陆的服务器

PIE: PIE总开始时间：0.199秒。

LogSlate: Updating window title bar state: overlay mode, drag disabled, window buttons hidden, title bar hidden

LogWorld: BeginTearingDown for /Game/UEDPIE_0_shili5

LogWorld: UWorld::CleanupWorld for shili5, bSessionEnded=true, bCleanupResources=true

LogSlate: InvalidateAllWidgets triggered. All widgets were invalidated

LogWorldMetrics: [UWorldMetricsSubsystem::Deinitialize]

LogWorldMetrics: [UWorldMetricsSubsystem::Clear]

LogPlayLevel: Display: Shutting down PIE online subsystems

LogSlate: InvalidateAllWidgets triggered. All widgets were invalidated

LogAudio: Display: Audio Device unregistered from world 'None'.

LogAudioMixer: Deinitializing Audio Bus Subsystem for audio device with ID 46

LogAudioMixer: FMixerPlatformXAudio2::StopAudioStream() called. InstanceID=46

LogAudioMixer: FMixerPlatformXAudio2::StopAudioStream() called. InstanceID=46

LogSlate: Updating window title bar state: overlay mode, drag disabled, window buttons hidden, title bar hidden

LogUObjectHash: Compacting FUObjectHashTables data took 1.33ms

LogPlayLevel: Display: Destroying online subsystem :Context_49

LogPlayLevel: PlayLevel: No blueprints needed recompiling

LogPlayLevel: Creating play world package: /Game/UEDPIE_0_shili5

LogPlayLevel: PIE: StaticDuplicateObject took: (0.054550s)

LogPlayLevel: PIE: Created PIE world by copying editor world from /Game/shili5.shili5 to /Game/UEDPIE_0_shili5.shili5 (0.054586s)

LogUObjectHash: Compacting FUObjectHashTables data took 1.48ms

LogWorldMetrics: [UWorldMetricsSubsystem::Initialize]

LogRenderer: SceneCulling instance hierarchy is disabled as UseNanite(PCD3D_SM5) returned false, for scene: 'World /Game/UEDPIE_0_shili5.shili5'.

LogPlayLevel: PIE: World Init took: (0.001168s)

LogAudio: Display: Creating Audio Device: Id: 47, Scope: Unique, Realtime: True

LogAudioMixer: Display: Audio Mixer Platform Settings:

LogAudioMixer: Display: Sample Rate: 48000

LogAudioMixer: Display: Callback Buffer Frame Size Requested: 1024

LogAudioMixer: Display: Callback Buffer Frame Size To Use: 1024

LogAudioMixer: Display: Number of buffers to queue: 2

LogAudioMixer: Display: Max Channels (voices): 32

LogAudioMixer: Display: Number of Async Source Workers: 0

LogAudio: Display: AudioDevice MaxSources: 32

LogAudio: Display: Audio Spatialization Plugin: None (built-in).

LogAudio: Display: Audio Reverb Plugin: None (built-in).

LogAudio: Display: Audio Occlusion Plugin: None (built-in).

LogAudioMixer: Display: Initializing audio mixer using platform API: 'XAudio2'

LogAudioMixer: Display: Using Audio Hardware Device 扬声器 (Realtek(R) Audio)

LogAudioMixer: Display: Initializing Sound Submixes...

LogAudioMixer: Display: Creating Master Submix 'MasterSubmixDefault'

LogAudioMixer: Display: Creating Master Submix 'MasterReverbSubmixDefault'

LogAudioMixer: FMixerPlatformXAudio2::StartAudioStream() called. InstanceID=47

LogAudioMixer: Display: Output buffers initialized: Frames=1024, Channels=2, Samples=2048, InstanceID=47

LogAudioMixer: Display: Starting AudioMixerPlatformInterface::RunInternal(), InstanceID=47

LogAudioMixer: Display: FMixerPlatformXAudio2::SubmitBuffer() called for the first time. InstanceID=47

LogInit: FAudioDevice initialized with ID 47.

LogAudio: Display: Audio Device (ID: 47) registered with world 'shili5'.

LogAudioMixer: Initializing Audio Bus Subsystem for audio device with ID 47

LogLoad: Game class is 'GameModeBase'

LogWorld: Bringing World /Game/UEDPIE_0_shili5.shili5 up for play (max tick rate 60) at 2025.12.10-19.25.32

LogWorld: Bringing up level for play took: 0.024017

LogOnline: OSS: Created online subsystem instance for: :Context_50

PIE: 登陆的服务器

PIE: PIE总开始时间：0.187秒。

LogSlate: Updating window title bar state: overlay mode, drag disabled, window buttons hidden, title bar hidden

LogWorld: BeginTearingDown for /Game/UEDPIE_0_shili5

LogWorld: UWorld::CleanupWorld for shili5, bSessionEnded=true, bCleanupResources=true

LogSlate: InvalidateAllWidgets triggered. All widgets were invalidated

LogWorldMetrics: [UWorldMetricsSubsystem::Deinitialize]

LogWorldMetrics: [UWorldMetricsSubsystem::Clear]

LogPlayLevel: Display: Shutting down PIE online subsystems

LogSlate: InvalidateAllWidgets triggered. All widgets were invalidated

LogSlate: Updating window title bar state: overlay mode, drag disabled, window buttons hidden, title bar hidden

LogAudioMixer: Deinitializing Audio Bus Subsystem for audio device with ID 47

LogAudioMixer: FMixerPlatformXAudio2::StopAudioStream() called. InstanceID=47

LogAudioMixer: FMixerPlatformXAudio2::StopAudioStream() called. InstanceID=47

LogUObjectHash: Compacting FUObjectHashTables data took 1.42ms

LogPlayLevel: Display: Destroying online subsystem :Context_50

LogDebuggerCommands: Repeating last play command: 选中的视口

LogPlayLevel: PlayLevel: No blueprints needed recompiling

LogPlayLevel: Creating play world package: /Game/UEDPIE_0_shili5

LogPlayLevel: PIE: StaticDuplicateObject took: (0.052246s)

LogPlayLevel: PIE: Created PIE world by copying editor world from /Game/shili5.shili5 to /Game/UEDPIE_0_shili5.shili5 (0.052280s)

LogUObjectHash: Compacting FUObjectHashTables data took 1.52ms

LogWorldMetrics: [UWorldMetricsSubsystem::Initialize]

LogRenderer: SceneCulling instance hierarchy is disabled as UseNanite(PCD3D_SM5) returned false, for scene: 'World /Game/UEDPIE_0_shili5.shili5'.

LogPlayLevel: PIE: World Init took: (0.001303s)

LogAudio: Display: Creating Audio Device: Id: 48, Scope: Unique, Realtime: True

LogAudioMixer: Display: Audio Mixer Platform Settings:

LogAudioMixer: Display: Sample Rate: 48000

LogAudioMixer: Display: Callback Buffer Frame Size Requested: 1024

LogAudioMixer: Display: Callback Buffer Frame Size To Use: 1024

LogAudioMixer: Display: Number of buffers to queue: 2

LogAudioMixer: Display: Max Channels (voices): 32

LogAudioMixer: Display: Number of Async Source Workers: 0

LogAudio: Display: AudioDevice MaxSources: 32

LogAudio: Display: Audio Spatialization Plugin: None (built-in).

LogAudio: Display: Audio Reverb Plugin: None (built-in).

LogAudio: Display: Audio Occlusion Plugin: None (built-in).

LogAudioMixer: Display: Initializing audio mixer using platform API: 'XAudio2'

LogAudioMixer: Display: Using Audio Hardware Device 扬声器 (Realtek(R) Audio)

LogAudioMixer: Display: Initializing Sound Submixes...

LogAudioMixer: Display: Creating Master Submix 'MasterSubmixDefault'

LogAudioMixer: Display: Creating Master Submix 'MasterReverbSubmixDefault'

LogAudioMixer: FMixerPlatformXAudio2::StartAudioStream() called. InstanceID=48

LogAudioMixer: Display: Output buffers initialized: Frames=1024, Channels=2, Samples=2048, InstanceID=48

LogAudioMixer: Display: Starting AudioMixerPlatformInterface::RunInternal(), InstanceID=48

LogAudioMixer: Display: FMixerPlatformXAudio2::SubmitBuffer() called for the first time. InstanceID=48

LogInit: FAudioDevice initialized with ID 48.

LogAudio: Display: Audio Device (ID: 48) registered with world 'shili5'.

LogAudioMixer: Initializing Audio Bus Subsystem for audio device with ID 48

LogLoad: Game class is 'GameModeBase'

LogWorld: Bringing World /Game/UEDPIE_0_shili5.shili5 up for play (max tick rate 60) at 2025.12.10-19.25.35

LogWorld: Bringing up level for play took: 0.025402

LogOnline: OSS: Created online subsystem instance for: :Context_51

PIE: 登陆的服务器

PIE: PIE总开始时间：0.188秒。

LogSlate: Updating window title bar state: overlay mode, drag disabled, window buttons hidden, title bar hidden

LogWorld: BeginTearingDown for /Game/UEDPIE_0_shili5

LogWorld: UWorld::CleanupWorld for shili5, bSessionEnded=true, bCleanupResources=true

LogSlate: InvalidateAllWidgets triggered. All widgets were invalidated

LogWorldMetrics: [UWorldMetricsSubsystem::Deinitialize]

LogWorldMetrics: [UWorldMetricsSubsystem::Clear]

LogPlayLevel: Display: Shutting down PIE online subsystems

LogSlate: InvalidateAllWidgets triggered. All widgets were invalidated

LogSlate: Updating window title bar state: overlay mode, drag disabled, window buttons hidden, title bar hidden

LogAudioMixer: Deinitializing Audio Bus Subsystem for audio device with ID 48

LogAudioMixer: FMixerPlatformXAudio2::StopAudioStream() called. InstanceID=48

LogAudioMixer: FMixerPlatformXAudio2::StopAudioStream() called. InstanceID=48

LogUObjectHash: Compacting FUObjectHashTables data took 1.50ms

LogPlayLevel: Display: Destroying online subsystem :Context_51

LogDebuggerCommands: Repeating last play command: 选中的视口

LogPlayLevel: PlayLevel: No blueprints needed recompiling

LogPlayLevel: Creating play world package: /Game/UEDPIE_0_shili5

LogPlayLevel: PIE: StaticDuplicateObject took: (0.053412s)

LogPlayLevel: PIE: Created PIE world by copying editor world from /Game/shili5.shili5 to /Game/UEDPIE_0_shili5.shili5 (0.053445s)

LogUObjectHash: Compacting FUObjectHashTables data took 1.52ms

LogWorldMetrics: [UWorldMetricsSubsystem::Initialize]

LogRenderer: SceneCulling instance hierarchy is disabled as UseNanite(PCD3D_SM5) returned false, for scene: 'World /Game/UEDPIE_0_shili5.shili5'.

LogPlayLevel: PIE: World Init took: (0.001146s)

LogAudio: Display: Creating Audio Device: Id: 49, Scope: Unique, Realtime: True

LogAudioMixer: Display: Audio Mixer Platform Settings:

LogAudioMixer: Display: Sample Rate: 48000

LogAudioMixer: Display: Callback Buffer Frame Size Requested: 1024

LogAudioMixer: Display: Callback Buffer Frame Size To Use: 1024

LogAudioMixer: Display: Number of buffers to queue: 2

LogAudioMixer: Display: Max Channels (voices): 32

LogAudioMixer: Display: Number of Async Source Workers: 0

LogAudio: Display: AudioDevice MaxSources: 32

LogAudio: Display: Audio Spatialization Plugin: None (built-in).

LogAudio: Display: Audio Reverb Plugin: None (built-in).

LogAudio: Display: Audio Occlusion Plugin: None (built-in).

LogAudioMixer: Display: Initializing audio mixer using platform API: 'XAudio2'

LogAudioMixer: Display: Using Audio Hardware Device 扬声器 (Realtek(R) Audio)

LogAudioMixer: Display: Initializing Sound Submixes...

LogAudioMixer: Display: Creating Master Submix 'MasterSubmixDefault'

LogAudioMixer: Display: Creating Master Submix 'MasterReverbSubmixDefault'

LogAudioMixer: FMixerPlatformXAudio2::StartAudioStream() called. InstanceID=49

LogAudioMixer: Display: Output buffers initialized: Frames=1024, Channels=2, Samples=2048, InstanceID=49

LogAudioMixer: Display: Starting AudioMixerPlatformInterface::RunInternal(), InstanceID=49

LogAudioMixer: Display: FMixerPlatformXAudio2::SubmitBuffer() called for the first time. InstanceID=49

LogInit: FAudioDevice initialized with ID 49.

LogAudio: Display: Audio Device (ID: 49) registered with world 'shili5'.

LogAudioMixer: Initializing Audio Bus Subsystem for audio device with ID 49

LogLoad: Game class is 'GameModeBase'

LogWorld: Bringing World /Game/UEDPIE_0_shili5.shili5 up for play (max tick rate 60) at 2025.12.10-19.25.44

LogWorld: Bringing up level for play took: 0.024146

LogOnline: OSS: Created online subsystem instance for: :Context_52

PIE: 登陆的服务器

PIE: PIE总开始时间：0.195秒。

Cmd: SELECT NONE

LogSlate: Updating window title bar state: overlay mode, drag disabled, window buttons hidden, title bar hidden

LogWorld: BeginTearingDown for /Game/UEDPIE_0_shili5

LogWorld: UWorld::CleanupWorld for shili5, bSessionEnded=true, bCleanupResources=true

LogSlate: InvalidateAllWidgets triggered. All widgets were invalidated

LogWorldMetrics: [UWorldMetricsSubsystem::Deinitialize]

LogWorldMetrics: [UWorldMetricsSubsystem::Clear]

LogPlayLevel: Display: Shutting down PIE online subsystems

LogSlate: InvalidateAllWidgets triggered. All widgets were invalidated

LogSlate: Updating window title bar state: overlay mode, drag disabled, window buttons hidden, title bar hidden

LogAudioMixer: Deinitializing Audio Bus Subsystem for audio device with ID 49

LogAudioMixer: FMixerPlatformXAudio2::StopAudioStream() called. InstanceID=49

LogAudioMixer: FMixerPlatformXAudio2::StopAudioStream() called. InstanceID=49

LogUObjectHash: Compacting FUObjectHashTables data took 1.42ms

LogPlayLevel: Display: Destroying online subsystem :Context_52

LogDebuggerCommands: Repeating last play command: 选中的视口

LogPlayLevel: PlayLevel: No blueprints needed recompiling

LogPlayLevel: Creating play world package: /Game/UEDPIE_0_shili5

LogPlayLevel: PIE: StaticDuplicateObject took: (0.050759s)

LogPlayLevel: PIE: Created PIE world by copying editor world from /Game/shili5.shili5 to /Game/UEDPIE_0_shili5.shili5 (0.050825s)

LogUObjectHash: Compacting FUObjectHashTables data took 1.47ms

LogWorldMetrics: [UWorldMetricsSubsystem::Initialize]

LogRenderer: SceneCulling instance hierarchy is disabled as UseNanite(PCD3D_SM5) returned false, for scene: 'World /Game/UEDPIE_0_shili5.shili5'.

LogPlayLevel: PIE: World Init took: (0.001876s)

LogAudio: Display: Creating Audio Device: Id: 50, Scope: Unique, Realtime: True

LogAudioMixer: Display: Audio Mixer Platform Settings:

LogAudioMixer: Display: Sample Rate: 48000

LogAudioMixer: Display: Callback Buffer Frame Size Requested: 1024

LogAudioMixer: Display: Callback Buffer Frame Size To Use: 1024

LogAudioMixer: Display: Number of buffers to queue: 2

LogAudioMixer: Display: Max Channels (voices): 32

LogAudioMixer: Display: Number of Async Source Workers: 0

LogAudio: Display: AudioDevice MaxSources: 32

LogAudio: Display: Audio Spatialization Plugin: None (built-in).

LogAudio: Display: Audio Reverb Plugin: None (built-in).

LogAudio: Display: Audio Occlusion Plugin: None (built-in).

LogAudioMixer: Display: Initializing audio mixer using platform API: 'XAudio2'

LogAudioMixer: Display: Using Audio Hardware Device 扬声器 (Realtek(R) Audio)

LogAudioMixer: Display: Initializing Sound Submixes...

LogAudioMixer: Display: Creating Master Submix 'MasterSubmixDefault'

LogAudioMixer: Display: Creating Master Submix 'MasterReverbSubmixDefault'

LogAudioMixer: FMixerPlatformXAudio2::StartAudioStream() called. InstanceID=50

LogAudioMixer: Display: Output buffers initialized: Frames=1024, Channels=2, Samples=2048, InstanceID=50

LogAudioMixer: Display: Starting AudioMixerPlatformInterface::RunInternal(), InstanceID=50

LogAudioMixer: Display: FMixerPlatformXAudio2::SubmitBuffer() called for the first time. InstanceID=50

LogInit: FAudioDevice initialized with ID 50.

LogAudio: Display: Audio Device (ID: 50) registered with world 'shili5'.

LogAudioMixer: Initializing Audio Bus Subsystem for audio device with ID 50

LogLoad: Game class is 'GameModeBase'

LogWorld: Bringing World /Game/UEDPIE_0_shili5.shili5 up for play (max tick rate 60) at 2025.12.10-19.25.52

LogWorld: Bringing up level for play took: 0.024098

LogOnline: OSS: Created online subsystem instance for: :Context_53

PIE: 登陆的服务器

PIE: PIE总开始时间：0.197秒。

LogSlate: Updating window title bar state: overlay mode, drag disabled, window buttons hidden, title bar hidden

LogWorld: BeginTearingDown for /Game/UEDPIE_0_shili5

LogWorld: UWorld::CleanupWorld for shili5, bSessionEnded=true, bCleanupResources=true

LogSlate: InvalidateAllWidgets triggered. All widgets were invalidated

LogWorldMetrics: [UWorldMetricsSubsystem::Deinitialize]

LogWorldMetrics: [UWorldMetricsSubsystem::Clear]

LogPlayLevel: Display: Shutting down PIE online subsystems

LogSlate: InvalidateAllWidgets triggered. All widgets were invalidated

LogSlate: Updating window title bar state: overlay mode, drag disabled, window buttons hidden, title bar hidden

LogAudioMixer: Deinitializing Audio Bus Subsystem for audio device with ID 50

LogAudioMixer: FMixerPlatformXAudio2::StopAudioStream() called. InstanceID=50

LogAudioMixer: FMixerPlatformXAudio2::StopAudioStream() called. InstanceID=50

LogUObjectHash: Compacting FUObjectHashTables data took 1.46ms

LogPlayLevel: Display: Destroying online subsystem :Context_53

LogPlayLevel: PlayLevel: No blueprints needed recompiling

LogPlayLevel: Creating play world package: /Game/UEDPIE_0_shili5

LogPlayLevel: PIE: StaticDuplicateObject took: (0.054195s)

LogPlayLevel: PIE: Created PIE world by copying editor world from /Game/shili5.shili5 to /Game/UEDPIE_0_shili5.shili5 (0.054230s)

LogUObjectHash: Compacting FUObjectHashTables data took 1.54ms

LogWorldMetrics: [UWorldMetricsSubsystem::Initialize]

LogRenderer: SceneCulling instance hierarchy is disabled as UseNanite(PCD3D_SM5) returned false, for scene: 'World /Game/UEDPIE_0_shili5.shili5'.

LogPlayLevel: PIE: World Init took: (0.001222s)

LogAudio: Display: Creating Audio Device: Id: 51, Scope: Unique, Realtime: True

LogAudioMixer: Display: Audio Mixer Platform Settings:

LogAudioMixer: Display: Sample Rate: 48000

LogAudioMixer: Display: Callback Buffer Frame Size Requested: 1024

LogAudioMixer: Display: Callback Buffer Frame Size To Use: 1024

LogAudioMixer: Display: Number of buffers to queue: 2

LogAudioMixer: Display: Max Channels (voices): 32

LogAudioMixer: Display: Number of Async Source Workers: 0

LogAudio: Display: AudioDevice MaxSources: 32

LogAudio: Display: Audio Spatialization Plugin: None (built-in).

LogAudio: Display: Audio Reverb Plugin: None (built-in).

LogAudio: Display: Audio Occlusion Plugin: None (built-in).

LogAudioMixer: Display: Initializing audio mixer using platform API: 'XAudio2'

LogAudioMixer: Display: Using Audio Hardware Device 扬声器 (Realtek(R) Audio)

LogAudioMixer: Display: Initializing Sound Submixes...

LogAudioMixer: Display: Creating Master Submix 'MasterSubmixDefault'

LogAudioMixer: Display: Creating Master Submix 'MasterReverbSubmixDefault'

LogAudioMixer: FMixerPlatformXAudio2::StartAudioStream() called. InstanceID=51

LogAudioMixer: Display: Output buffers initialized: Frames=1024, Channels=2, Samples=2048, InstanceID=51

LogAudioMixer: Display: Starting AudioMixerPlatformInterface::RunInternal(), InstanceID=51

LogAudioMixer: Display: FMixerPlatformXAudio2::SubmitBuffer() called for the first time. InstanceID=51

LogInit: FAudioDevice initialized with ID 51.

LogAudio: Display: Audio Device (ID: 51) registered with world 'shili5'.

LogAudioMixer: Initializing Audio Bus Subsystem for audio device with ID 51

LogSlate: Updating window title bar state: overlay mode, drag disabled, window buttons hidden, title bar hidden

LogLoad: Game class is 'GameModeBase'

LogWorld: Bringing World /Game/UEDPIE_0_shili5.shili5 up for play (max tick rate 60) at 2025.12.10-19.26.01

LogWorld: Bringing up level for play took: 0.030416

LogOnline: OSS: Created online subsystem instance for: :Context_54

PIE: 登陆的服务器

PIE: PIE总开始时间：0.258秒。

LogSlate: Updating window title bar state: overlay mode, drag disabled, window buttons hidden, title bar hidden

LogWorld: BeginTearingDown for /Game/UEDPIE_0_shili5

LogSlate: Window 'wodexiangmu2 预览 [NetMode: Standalone 0] （64-bit/PC D3D SM5）' being destroyed

LogWorld: UWorld::CleanupWorld for shili5, bSessionEnded=true, bCleanupResources=true

LogSlate: InvalidateAllWidgets triggered. All widgets were invalidated

LogWorldMetrics: [UWorldMetricsSubsystem::Deinitialize]

LogWorldMetrics: [UWorldMetricsSubsystem::Clear]

LogPlayLevel: Display: Shutting down PIE online subsystems

LogSlate: InvalidateAllWidgets triggered. All widgets were invalidated

LogAudioMixer: Deinitializing Audio Bus Subsystem for audio device with ID 51

LogAudioMixer: FMixerPlatformXAudio2::StopAudioStream() called. InstanceID=51

LogAudioMixer: FMixerPlatformXAudio2::StopAudioStream() called. InstanceID=51

LogUObjectHash: Compacting FUObjectHashTables data took 2.60ms

LogPlayLevel: Display: Destroying online subsystem :Context_54

LogDebuggerCommands: Repeating last play command: 新建编辑器窗口（PIE）

LogPlayLevel: PlayLevel: No blueprints needed recompiling

LogPlayLevel: Creating play world package: /Game/UEDPIE_0_shili5

LogPlayLevel: PIE: StaticDuplicateObject took: (0.048711s)

LogPlayLevel: PIE: Created PIE world by copying editor world from /Game/shili5.shili5 to /Game/UEDPIE_0_shili5.shili5 (0.048743s)

LogUObjectHash: Compacting FUObjectHashTables data took 1.47ms

LogWorldMetrics: [UWorldMetricsSubsystem::Initialize]

LogRenderer: SceneCulling instance hierarchy is disabled as UseNanite(PCD3D_SM5) returned false, for scene: 'World /Game/UEDPIE_0_shili5.shili5'.

LogPlayLevel: PIE: World Init took: (0.001168s)

LogAudio: Display: Creating Audio Device: Id: 52, Scope: Unique, Realtime: True

LogAudioMixer: Display: Audio Mixer Platform Settings:

LogAudioMixer: Display: Sample Rate: 48000

LogAudioMixer: Display: Callback Buffer Frame Size Requested: 1024

LogAudioMixer: Display: Callback Buffer Frame Size To Use: 1024

LogAudioMixer: Display: Number of buffers to queue: 2

LogAudioMixer: Display: Max Channels (voices): 32

LogAudioMixer: Display: Number of Async Source Workers: 0

LogAudio: Display: AudioDevice MaxSources: 32

LogAudio: Display: Audio Spatialization Plugin: None (built-in).

LogAudio: Display: Audio Reverb Plugin: None (built-in).

LogAudio: Display: Audio Occlusion Plugin: None (built-in).

LogAudioMixer: Display: Initializing audio mixer using platform API: 'XAudio2'

LogAudioMixer: Display: Using Audio Hardware Device 扬声器 (Realtek(R) Audio)

LogAudioMixer: Display: Initializing Sound Submixes...

LogAudioMixer: Display: Creating Master Submix 'MasterSubmixDefault'

LogAudioMixer: Display: Creating Master Submix 'MasterReverbSubmixDefault'

LogAudioMixer: FMixerPlatformXAudio2::StartAudioStream() called. InstanceID=52

LogAudioMixer: Display: Output buffers initialized: Frames=1024, Channels=2, Samples=2048, InstanceID=52

LogAudioMixer: Display: Starting AudioMixerPlatformInterface::RunInternal(), InstanceID=52

LogAudioMixer: Display: FMixerPlatformXAudio2::SubmitBuffer() called for the first time. InstanceID=52

LogInit: FAudioDevice initialized with ID 52.

LogAudio: Display: Audio Device (ID: 52) registered with world 'shili5'.

LogAudioMixer: Initializing Audio Bus Subsystem for audio device with ID 52

LogSlate: Updating window title bar state: overlay mode, drag disabled, window buttons hidden, title bar hidden

LogLoad: Game class is 'GameModeBase'

LogWorld: Bringing World /Game/UEDPIE_0_shili5.shili5 up for play (max tick rate 60) at 2025.12.10-19.26.06

LogWorld: Bringing up level for play took: 0.027138

LogOnline: OSS: Created online subsystem instance for: :Context_55

PIE: 登陆的服务器

PIE: PIE总开始时间：0.233秒。

LogSlate: Updating window title bar state: overlay mode, drag disabled, window buttons hidden, title bar hidden

LogWorld: BeginTearingDown for /Game/UEDPIE_0_shili5

LogSlate: Window 'wodexiangmu2 预览 [NetMode: Standalone 0] （64-bit/PC D3D SM5）' being destroyed

LogWorld: UWorld::CleanupWorld for shili5, bSessionEnded=true, bCleanupResources=true

LogSlate: InvalidateAllWidgets triggered. All widgets were invalidated

LogWorldMetrics: [UWorldMetricsSubsystem::Deinitialize]

LogWorldMetrics: [UWorldMetricsSubsystem::Clear]

LogPlayLevel: Display: Shutting down PIE online subsystems

LogSlate: InvalidateAllWidgets triggered. All widgets were invalidated

LogAudioMixer: Deinitializing Audio Bus Subsystem for audio device with ID 52

LogAudioMixer: FMixerPlatformXAudio2::StopAudioStream() called. InstanceID=52

LogAudioMixer: FMixerPlatformXAudio2::StopAudioStream() called. InstanceID=52

LogUObjectHash: Compacting FUObjectHashTables data took 1.59ms

LogPlayLevel: Display: Destroying online subsystem :Context_55

LogPlayLevel: PlayLevel: No blueprints needed recompiling

LogPlayLevel: Creating play world package: /Game/UEDPIE_0_shili5

LogPlayLevel: PIE: StaticDuplicateObject took: (0.049619s)

LogPlayLevel: PIE: Created PIE world by copying editor world from /Game/shili5.shili5 to /Game/UEDPIE_0_shili5.shili5 (0.049652s)

LogUObjectHash: Compacting FUObjectHashTables data took 1.50ms

LogWorldMetrics: [UWorldMetricsSubsystem::Initialize]

LogRenderer: SceneCulling instance hierarchy is disabled as UseNanite(PCD3D_SM5) returned false, for scene: 'World /Game/UEDPIE_0_shili5.shili5'.

LogPlayLevel: PIE: World Init took: (0.001146s)

LogAudio: Display: Creating Audio Device: Id: 53, Scope: Unique, Realtime: True

LogAudioMixer: Display: Audio Mixer Platform Settings:

LogAudioMixer: Display: Sample Rate: 48000

LogAudioMixer: Display: Callback Buffer Frame Size Requested: 1024

LogAudioMixer: Display: Callback Buffer Frame Size To Use: 1024

LogAudioMixer: Display: Number of buffers to queue: 2

LogAudioMixer: Display: Max Channels (voices): 32

LogAudioMixer: Display: Number of Async Source Workers: 0

LogAudio: Display: AudioDevice MaxSources: 32

LogAudio: Display: Audio Spatialization Plugin: None (built-in).

LogAudio: Display: Audio Reverb Plugin: None (built-in).

LogAudio: Display: Audio Occlusion Plugin: None (built-in).

LogAudioMixer: Display: Initializing audio mixer using platform API: 'XAudio2'

LogAudioMixer: Display: Using Audio Hardware Device 扬声器 (Realtek(R) Audio)

LogAudioMixer: Display: Initializing Sound Submixes...

LogAudioMixer: Display: Creating Master Submix 'MasterSubmixDefault'

LogAudioMixer: Display: Creating Master Submix 'MasterReverbSubmixDefault'

LogAudioMixer: FMixerPlatformXAudio2::StartAudioStream() called. InstanceID=53

LogAudioMixer: Display: Output buffers initialized: Frames=1024, Channels=2, Samples=2048, InstanceID=53

LogAudioMixer: Display: Starting AudioMixerPlatformInterface::RunInternal(), InstanceID=53

LogAudioMixer: Display: FMixerPlatformXAudio2::SubmitBuffer() called for the first time. InstanceID=53

LogInit: FAudioDevice initialized with ID 53.

LogAudio: Display: Audio Device (ID: 53) registered with world 'shili5'.

LogAudioMixer: Initializing Audio Bus Subsystem for audio device with ID 53

LogLoad: Game class is 'GameModeBase'

LogWorld: Bringing World /Game/UEDPIE_0_shili5.shili5 up for play (max tick rate 60) at 2025.12.10-19.26.16

LogWorld: Bringing up level for play took: 0.024361

LogOnline: OSS: Created online subsystem instance for: :Context_56

LogGameMode: FindPlayerStart: PATHS NOT DEFINED or NO PLAYERSTART with positive rating

PIE: 登陆的服务器

PIE: PIE总开始时间：0.19秒。

LogSlate: Updating window title bar state: overlay mode, drag disabled, window buttons hidden, title bar hidden

LogWorld: BeginTearingDown for /Game/UEDPIE_0_shili5

LogWorld: UWorld::CleanupWorld for shili5, bSessionEnded=true, bCleanupResources=true

LogSlate: InvalidateAllWidgets triggered. All widgets were invalidated

LogWorldMetrics: [UWorldMetricsSubsystem::Deinitialize]

LogWorldMetrics: [UWorldMetricsSubsystem::Clear]

LogPlayLevel: Display: Shutting down PIE online subsystems

LogSlate: InvalidateAllWidgets triggered. All widgets were invalidated

LogSlate: Updating window title bar state: overlay mode, drag disabled, window buttons hidden, title bar hidden

LogAudioMixer: Deinitializing Audio Bus Subsystem for audio device with ID 53

LogAudioMixer: FMixerPlatformXAudio2::StopAudioStream() called. InstanceID=53

LogAudioMixer: FMixerPlatformXAudio2::StopAudioStream() called. InstanceID=53

LogUObjectHash: Compacting FUObjectHashTables data took 1.43ms

LogPlayLevel: Display: Destroying online subsystem :Context_56

LogDebuggerCommands: Repeating last play command: 模拟

LogPlayLevel: PlayLevel: No blueprints needed recompiling

LogPlayLevel: Creating play world package: /Game/UEDPIE_0_shili5

LogPlayLevel: PIE: StaticDuplicateObject took: (0.049667s)

LogPlayLevel: PIE: Created PIE world by copying editor world from /Game/shili5.shili5 to /Game/UEDPIE_0_shili5.shili5 (0.049699s)

LogUObjectHash: Compacting FUObjectHashTables data took 1.42ms

LogWorldMetrics: [UWorldMetricsSubsystem::Initialize]

LogRenderer: SceneCulling instance hierarchy is disabled as UseNanite(PCD3D_SM5) returned false, for scene: 'World /Game/UEDPIE_0_shili5.shili5'.

LogPlayLevel: PIE: World Init took: (0.001182s)

LogAudio: Display: Creating Audio Device: Id: 54, Scope: Unique, Realtime: True

LogAudioMixer: Display: Audio Mixer Platform Settings:

LogAudioMixer: Display: Sample Rate: 48000

LogAudioMixer: Display: Callback Buffer Frame Size Requested: 1024

LogAudioMixer: Display: Callback Buffer Frame Size To Use: 1024

LogAudioMixer: Display: Number of buffers to queue: 2

LogAudioMixer: Display: Max Channels (voices): 32

LogAudioMixer: Display: Number of Async Source Workers: 0

LogAudio: Display: AudioDevice MaxSources: 32

LogAudio: Display: Audio Spatialization Plugin: None (built-in).

LogAudio: Display: Audio Reverb Plugin: None (built-in).

LogAudio: Display: Audio Occlusion Plugin: None (built-in).

LogAudioMixer: Display: Initializing audio mixer using platform API: 'XAudio2'

LogAudioMixer: Display: Using Audio Hardware Device 扬声器 (Realtek(R) Audio)

LogAudioMixer: Display: Initializing Sound Submixes...

LogAudioMixer: Display: Creating Master Submix 'MasterSubmixDefault'

LogAudioMixer: Display: Creating Master Submix 'MasterReverbSubmixDefault'

LogAudioMixer: FMixerPlatformXAudio2::StartAudioStream() called. InstanceID=54

LogAudioMixer: Display: Output buffers initialized: Frames=1024, Channels=2, Samples=2048, InstanceID=54

LogAudioMixer: Display: Starting AudioMixerPlatformInterface::RunInternal(), InstanceID=54

LogAudioMixer: Display: FMixerPlatformXAudio2::SubmitBuffer() called for the first time. InstanceID=54

LogInit: FAudioDevice initialized with ID 54.

LogAudio: Display: Audio Device (ID: 54) registered with world 'shili5'.

LogAudioMixer: Initializing Audio Bus Subsystem for audio device with ID 54

LogLoad: Game class is 'GameModeBase'

LogWorld: Bringing World /Game/UEDPIE_0_shili5.shili5 up for play (max tick rate 60) at 2025.12.10-19.26.19

LogWorld: Bringing up level for play took: 0.024462

LogOnline: OSS: Created online subsystem instance for: :Context_57

LogGameMode: FindPlayerStart: PATHS NOT DEFINED or NO PLAYERSTART with positive rating

PIE: 登陆的服务器

PIE: PIE总开始时间：0.184秒。

LogSlate: Updating window title bar state: overlay mode, drag disabled, window buttons hidden, title bar hidden

LogWorld: BeginTearingDown for /Game/UEDPIE_0_shili5

LogWorld: UWorld::CleanupWorld for shili5, bSessionEnded=true, bCleanupResources=true

LogSlate: InvalidateAllWidgets triggered. All widgets were invalidated

LogWorldMetrics: [UWorldMetricsSubsystem::Deinitialize]

LogWorldMetrics: [UWorldMetricsSubsystem::Clear]

LogPlayLevel: Display: Shutting down PIE online subsystems

LogSlate: InvalidateAllWidgets triggered. All widgets were invalidated

LogSlate: Updating window title bar state: overlay mode, drag disabled, window buttons hidden, title bar hidden

LogAudioMixer: Deinitializing Audio Bus Subsystem for audio device with ID 54

LogAudioMixer: FMixerPlatformXAudio2::StopAudioStream() called. InstanceID=54

LogAudioMixer: FMixerPlatformXAudio2::StopAudioStream() called. InstanceID=54

LogUObjectHash: Compacting FUObjectHashTables data took 1.34ms

LogPlayLevel: Display: Destroying online subsystem :Context_57

LogDebuggerCommands: Repeating last play command: 模拟

LogPlayLevel: PlayLevel: No blueprints needed recompiling

LogPlayLevel: Creating play world package: /Game/UEDPIE_0_shili5

LogPlayLevel: PIE: StaticDuplicateObject took: (0.048183s)

LogPlayLevel: PIE: Created PIE world by copying editor world from /Game/shili5.shili5 to /Game/UEDPIE_0_shili5.shili5 (0.048214s)

LogUObjectHash: Compacting FUObjectHashTables data took 1.40ms

LogWorldMetrics: [UWorldMetricsSubsystem::Initialize]

LogRenderer: SceneCulling instance hierarchy is disabled as UseNanite(PCD3D_SM5) returned false, for scene: 'World /Game/UEDPIE_0_shili5.shili5'.

LogPlayLevel: PIE: World Init took: (0.001197s)

LogAudio: Display: Creating Audio Device: Id: 55, Scope: Unique, Realtime: True

LogAudioMixer: Display: Audio Mixer Platform Settings:

LogAudioMixer: Display: Sample Rate: 48000

LogAudioMixer: Display: Callback Buffer Frame Size Requested: 1024

LogAudioMixer: Display: Callback Buffer Frame Size To Use: 1024

LogAudioMixer: Display: Number of buffers to queue: 2

LogAudioMixer: Display: Max Channels (voices): 32

LogAudioMixer: Display: Number of Async Source Workers: 0

LogAudio: Display: AudioDevice MaxSources: 32

LogAudio: Display: Audio Spatialization Plugin: None (built-in).

LogAudio: Display: Audio Reverb Plugin: None (built-in).

LogAudio: Display: Audio Occlusion Plugin: None (built-in).

LogAudioMixer: Display: Initializing audio mixer using platform API: 'XAudio2'

LogAudioMixer: Display: Using Audio Hardware Device 扬声器 (Realtek(R) Audio)

LogAudioMixer: Display: Initializing Sound Submixes...

LogAudioMixer: Display: Creating Master Submix 'MasterSubmixDefault'

LogAudioMixer: Display: Creating Master Submix 'MasterReverbSubmixDefault'

LogAudioMixer: FMixerPlatformXAudio2::StartAudioStream() called. InstanceID=55

LogAudioMixer: Display: Output buffers initialized: Frames=1024, Channels=2, Samples=2048, InstanceID=55

LogAudioMixer: Display: Starting AudioMixerPlatformInterface::RunInternal(), InstanceID=55

LogAudioMixer: Display: FMixerPlatformXAudio2::SubmitBuffer() called for the first time. InstanceID=55

LogInit: FAudioDevice initialized with ID 55.

LogAudio: Display: Audio Device (ID: 55) registered with world 'shili5'.

LogAudioMixer: Initializing Audio Bus Subsystem for audio device with ID 55

LogLoad: Game class is 'GameModeBase'

LogWorld: Bringing World /Game/UEDPIE_0_shili5.shili5 up for play (max tick rate 60) at 2025.12.10-19.26.49

LogWorld: Bringing up level for play took: 0.022602

LogOnline: OSS: Created online subsystem instance for: :Context_58

LogGameMode: FindPlayerStart: PATHS NOT DEFINED or NO PLAYERSTART with positive rating

PIE: 登陆的服务器

PIE: PIE总开始时间：0.184秒。

LogSlate: Updating window title bar state: overlay mode, drag disabled, window buttons hidden, title bar hidden

LogWorld: BeginTearingDown for /Game/UEDPIE_0_shili5

LogWorld: UWorld::CleanupWorld for shili5, bSessionEnded=true, bCleanupResources=true

LogSlate: InvalidateAllWidgets triggered. All widgets were invalidated

LogWorldMetrics: [UWorldMetricsSubsystem::Deinitialize]

LogWorldMetrics: [UWorldMetricsSubsystem::Clear]

LogPlayLevel: Display: Shutting down PIE online subsystems

LogSlate: InvalidateAllWidgets triggered. All widgets were invalidated

LogSlate: Updating window title bar state: overlay mode, drag disabled, window buttons hidden, title bar hidden

LogAudioMixer: Deinitializing Audio Bus Subsystem for audio device with ID 55

LogAudioMixer: FMixerPlatformXAudio2::StopAudioStream() called. InstanceID=55

LogAudioMixer: FMixerPlatformXAudio2::StopAudioStream() called. InstanceID=55

LogUObjectHash: Compacting FUObjectHashTables data took 1.39ms

LogPlayLevel: Display: Destroying online subsystem :Context_58

LogEditorViewport: Clicking Background

LogDebuggerCommands: Repeating last play command: 模拟

LogPlayLevel: PlayLevel: No blueprints needed recompiling

LogPlayLevel: Creating play world package: /Game/UEDPIE_0_shili5

LogPlayLevel: PIE: StaticDuplicateObject took: (0.051902s)

LogPlayLevel: PIE: Created PIE world by copying editor world from /Game/shili5.shili5 to /Game/UEDPIE_0_shili5.shili5 (0.051936s)

LogUObjectHash: Compacting FUObjectHashTables data took 1.44ms

LogWorldMetrics: [UWorldMetricsSubsystem::Initialize]

LogRenderer: SceneCulling instance hierarchy is disabled as UseNanite(PCD3D_SM5) returned false, for scene: 'World /Game/UEDPIE_0_shili5.shili5'.

LogPlayLevel: PIE: World Init took: (0.001185s)

LogAudio: Display: Creating Audio Device: Id: 56, Scope: Unique, Realtime: True

LogAudioMixer: Display: Audio Mixer Platform Settings:

LogAudioMixer: Display: Sample Rate: 48000

LogAudioMixer: Display: Callback Buffer Frame Size Requested: 1024

LogAudioMixer: Display: Callback Buffer Frame Size To Use: 1024

LogAudioMixer: Display: Number of buffers to queue: 2

LogAudioMixer: Display: Max Channels (voices): 32

LogAudioMixer: Display: Number of Async Source Workers: 0

LogAudio: Display: AudioDevice MaxSources: 32

LogAudio: Display: Audio Spatialization Plugin: None (built-in).

LogAudio: Display: Audio Reverb Plugin: None (built-in).

LogAudio: Display: Audio Occlusion Plugin: None (built-in).

LogAudioMixer: Display: Initializing audio mixer using platform API: 'XAudio2'

LogAudioMixer: Display: Using Audio Hardware Device 扬声器 (Realtek(R) Audio)

LogAudioMixer: Display: Initializing Sound Submixes...

LogAudioMixer: Display: Creating Master Submix 'MasterSubmixDefault'

LogAudioMixer: Display: Creating Master Submix 'MasterReverbSubmixDefault'

LogAudioMixer: FMixerPlatformXAudio2::StartAudioStream() called. InstanceID=56

LogAudioMixer: Display: Output buffers initialized: Frames=1024, Channels=2, Samples=2048, InstanceID=56

LogAudioMixer: Display: Starting AudioMixerPlatformInterface::RunInternal(), InstanceID=56

LogAudioMixer: Display: FMixerPlatformXAudio2::SubmitBuffer() called for the first time. InstanceID=56

LogInit: FAudioDevice initialized with ID 56.

LogAudio: Display: Audio Device (ID: 56) registered with world 'shili5'.

LogAudioMixer: Initializing Audio Bus Subsystem for audio device with ID 56

LogLoad: Game class is 'GameModeBase'

LogWorld: Bringing World /Game/UEDPIE_0_shili5.shili5 up for play (max tick rate 60) at 2025.12.10-19.26.59

LogWorld: Bringing up level for play took: 0.025236

LogOnline: OSS: Created online subsystem instance for: :Context_59

LogGameMode: FindPlayerStart: PATHS NOT DEFINED or NO PLAYERSTART with positive rating

PIE: 登陆的服务器

PIE: PIE总开始时间：0.192秒。

LogSlate: Updating window title bar state: overlay mode, drag disabled, window buttons hidden, title bar hidden

LogWorld: BeginTearingDown for /Game/UEDPIE_0_shili5

LogWorld: UWorld::CleanupWorld for shili5, bSessionEnded=true, bCleanupResources=true

LogSlate: InvalidateAllWidgets triggered. All widgets were invalidated

LogWorldMetrics: [UWorldMetricsSubsystem::Deinitialize]

LogWorldMetrics: [UWorldMetricsSubsystem::Clear]

LogPlayLevel: Display: Shutting down PIE online subsystems

LogSlate: InvalidateAllWidgets triggered. All widgets were invalidated

LogSlate: Updating window title bar state: overlay mode, drag disabled, window buttons hidden, title bar hidden

LogAudioMixer: Deinitializing Audio Bus Subsystem for audio device with ID 56

LogAudioMixer: FMixerPlatformXAudio2::StopAudioStream() called. InstanceID=56

LogAudioMixer: FMixerPlatformXAudio2::StopAudioStream() called. InstanceID=56

LogUObjectHash: Compacting FUObjectHashTables data took 2.02ms

LogPlayLevel: Display: Destroying online subsystem :Context_59

LogDebuggerCommands: Repeating last play command: 模拟

LogPlayLevel: PlayLevel: No blueprints needed recompiling

LogPlayLevel: Creating play world package: /Game/UEDPIE_0_shili5

LogPlayLevel: PIE: StaticDuplicateObject took: (0.046250s)

LogPlayLevel: PIE: Created PIE world by copying editor world from /Game/shili5.shili5 to /Game/UEDPIE_0_shili5.shili5 (0.046284s)

LogUObjectHash: Compacting FUObjectHashTables data took 1.36ms

LogWorldMetrics: [UWorldMetricsSubsystem::Initialize]

LogRenderer: SceneCulling instance hierarchy is disabled as UseNanite(PCD3D_SM5) returned false, for scene: 'World /Game/UEDPIE_0_shili5.shili5'.

LogPlayLevel: PIE: World Init took: (0.001129s)

LogAudio: Display: Creating Audio Device: Id: 57, Scope: Unique, Realtime: True

LogAudioMixer: Display: Audio Mixer Platform Settings:

LogAudioMixer: Display: Sample Rate: 48000

LogAudioMixer: Display: Callback Buffer Frame Size Requested: 1024

LogAudioMixer: Display: Callback Buffer Frame Size To Use: 1024

LogAudioMixer: Display: Number of buffers to queue: 2

LogAudioMixer: Display: Max Channels (voices): 32

LogAudioMixer: Display: Number of Async Source Workers: 0

LogAudio: Display: AudioDevice MaxSources: 32

LogAudio: Display: Audio Spatialization Plugin: None (built-in).

LogAudio: Display: Audio Reverb Plugin: None (built-in).

LogAudio: Display: Audio Occlusion Plugin: None (built-in).

LogAudioMixer: Display: Initializing audio mixer using platform API: 'XAudio2'

LogAudioMixer: Display: Using Audio Hardware Device 扬声器 (Realtek(R) Audio)

LogAudioMixer: Display: Initializing Sound Submixes...

LogAudioMixer: Display: Creating Master Submix 'MasterSubmixDefault'

LogAudioMixer: Display: Creating Master Submix 'MasterReverbSubmixDefault'

LogAudioMixer: FMixerPlatformXAudio2::StartAudioStream() called. InstanceID=57

LogAudioMixer: Display: Output buffers initialized: Frames=1024, Channels=2, Samples=2048, InstanceID=57

LogAudioMixer: Display: Starting AudioMixerPlatformInterface::RunInternal(), InstanceID=57

LogInit: FAudioDevice initialized with ID 57.

LogAudioMixer: Display: FMixerPlatformXAudio2::SubmitBuffer() called for the first time. InstanceID=57

LogAudio: Display: Audio Device (ID: 57) registered with world 'shili5'.

LogAudioMixer: Initializing Audio Bus Subsystem for audio device with ID 57

LogLoad: Game class is 'GameModeBase'

LogWorld: Bringing World /Game/UEDPIE_0_shili5.shili5 up for play (max tick rate 60) at 2025.12.10-19.27.52

LogWorld: Bringing up level for play took: 0.021744

LogOnline: OSS: Created online subsystem instance for: :Context_60

LogGameMode: FindPlayerStart: PATHS NOT DEFINED or NO PLAYERSTART with positive rating

PIE: 登陆的服务器

PIE: PIE总开始时间：0.177秒。

LogSlate: Updating window title bar state: overlay mode, drag disabled, window buttons hidden, title bar hidden

LogWorld: BeginTearingDown for /Game/UEDPIE_0_shili5

LogWorld: UWorld::CleanupWorld for shili5, bSessionEnded=true, bCleanupResources=true

LogSlate: InvalidateAllWidgets triggered. All widgets were invalidated

LogWorldMetrics: [UWorldMetricsSubsystem::Deinitialize]

LogWorldMetrics: [UWorldMetricsSubsystem::Clear]

LogPlayLevel: Display: Shutting down PIE online subsystems

LogSlate: InvalidateAllWidgets triggered. All widgets were invalidated

LogSlate: Updating window title bar state: overlay mode, drag disabled, window buttons hidden, title bar hidden

LogAudioMixer: Deinitializing Audio Bus Subsystem for audio device with ID 57

LogAudioMixer: FMixerPlatformXAudio2::StopAudioStream() called. InstanceID=57

LogAudioMixer: FMixerPlatformXAudio2::StopAudioStream() called. InstanceID=57

LogUObjectHash: Compacting FUObjectHashTables data took 1.39ms

LogPlayLevel: Display: Destroying online subsystem :Context_60

LogDebuggerCommands: Repeating last play command: 模拟

LogPlayLevel: PlayLevel: No blueprints needed recompiling

LogPlayLevel: Creating play world package: /Game/UEDPIE_0_shili5

LogPlayLevel: PIE: StaticDuplicateObject took: (0.045365s)

LogPlayLevel: PIE: Created PIE world by copying editor world from /Game/shili5.shili5 to /Game/UEDPIE_0_shili5.shili5 (0.045395s)

LogUObjectHash: Compacting FUObjectHashTables data took 1.37ms

LogWorldMetrics: [UWorldMetricsSubsystem::Initialize]

LogRenderer: SceneCulling instance hierarchy is disabled as UseNanite(PCD3D_SM5) returned false, for scene: 'World /Game/UEDPIE_0_shili5.shili5'.

LogPlayLevel: PIE: World Init took: (0.001048s)

LogAudio: Display: Creating Audio Device: Id: 58, Scope: Unique, Realtime: True

LogAudioMixer: Display: Audio Mixer Platform Settings:

LogAudioMixer: Display: Sample Rate: 48000

LogAudioMixer: Display: Callback Buffer Frame Size Requested: 1024

LogAudioMixer: Display: Callback Buffer Frame Size To Use: 1024

LogAudioMixer: Display: Number of buffers to queue: 2

LogAudioMixer: Display: Max Channels (voices): 32

LogAudioMixer: Display: Number of Async Source Workers: 0

LogAudio: Display: AudioDevice MaxSources: 32

LogAudio: Display: Audio Spatialization Plugin: None (built-in).

LogAudio: Display: Audio Reverb Plugin: None (built-in).

LogAudio: Display: Audio Occlusion Plugin: None (built-in).

LogAudioMixer: Display: Initializing audio mixer using platform API: 'XAudio2'

LogAudioMixer: Display: Using Audio Hardware Device 扬声器 (Realtek(R) Audio)

LogAudioMixer: Display: Initializing Sound Submixes...

LogAudioMixer: Display: Creating Master Submix 'MasterSubmixDefault'

LogAudioMixer: Display: Creating Master Submix 'MasterReverbSubmixDefault'

LogAudioMixer: FMixerPlatformXAudio2::StartAudioStream() called. InstanceID=58

LogAudioMixer: Display: Output buffers initialized: Frames=1024, Channels=2, Samples=2048, InstanceID=58

LogAudioMixer: Display: Starting AudioMixerPlatformInterface::RunInternal(), InstanceID=58

LogAudioMixer: Display: FMixerPlatformXAudio2::SubmitBuffer() called for the first time. InstanceID=58

LogInit: FAudioDevice initialized with ID 58.

LogAudio: Display: Audio Device (ID: 58) registered with world 'shili5'.

LogAudioMixer: Initializing Audio Bus Subsystem for audio device with ID 58

LogLoad: Game class is 'GameModeBase'

LogWorld: Bringing World /Game/UEDPIE_0_shili5.shili5 up for play (max tick rate 60) at 2025.12.10-19.28.19

LogWorld: Bringing up level for play took: 0.021210

LogOnline: OSS: Created online subsystem instance for: :Context_61

LogGameMode: FindPlayerStart: PATHS NOT DEFINED or NO PLAYERSTART with positive rating

PIE: 登陆的服务器

PIE: PIE总开始时间：0.174秒。

LogSlate: Updating window title bar state: overlay mode, drag disabled, window buttons hidden, title bar hidden

LogWorld: BeginTearingDown for /Game/UEDPIE_0_shili5

LogWorld: UWorld::CleanupWorld for shili5, bSessionEnded=true, bCleanupResources=true

LogSlate: InvalidateAllWidgets triggered. All widgets were invalidated

LogWorldMetrics: [UWorldMetricsSubsystem::Deinitialize]

LogWorldMetrics: [UWorldMetricsSubsystem::Clear]

LogPlayLevel: Display: Shutting down PIE online subsystems

LogSlate: InvalidateAllWidgets triggered. All widgets were invalidated

LogSlate: Updating window title bar state: overlay mode, drag disabled, window buttons hidden, title bar hidden

LogAudioMixer: Deinitializing Audio Bus Subsystem for audio device with ID 58

LogAudioMixer: FMixerPlatformXAudio2::StopAudioStream() called. InstanceID=58

LogAudioMixer: FMixerPlatformXAudio2::StopAudioStream() called. InstanceID=58

LogUObjectHash: Compacting FUObjectHashTables data took 1.40ms

LogPlayLevel: Display: Destroying online subsystem :Context_61

LogDebuggerCommands: Repeating last play command: 模拟

LogPlayLevel: PlayLevel: No blueprints needed recompiling

LogPlayLevel: Creating play world package: /Game/UEDPIE_0_shili5

LogPlayLevel: PIE: StaticDuplicateObject took: (0.045892s)

LogPlayLevel: PIE: Created PIE world by copying editor world from /Game/shili5.shili5 to /Game/UEDPIE_0_shili5.shili5 (0.045923s)

LogUObjectHash: Compacting FUObjectHashTables data took 1.28ms

LogWorldMetrics: [UWorldMetricsSubsystem::Initialize]

LogRenderer: SceneCulling instance hierarchy is disabled as UseNanite(PCD3D_SM5) returned false, for scene: 'World /Game/UEDPIE_0_shili5.shili5'.

LogPlayLevel: PIE: World Init took: (0.001068s)

LogAudio: Display: Creating Audio Device: Id: 59, Scope: Unique, Realtime: True

LogAudioMixer: Display: Audio Mixer Platform Settings:

LogAudioMixer: Display: Sample Rate: 48000

LogAudioMixer: Display: Callback Buffer Frame Size Requested: 1024

LogAudioMixer: Display: Callback Buffer Frame Size To Use: 1024

LogAudioMixer: Display: Number of buffers to queue: 2

LogAudioMixer: Display: Max Channels (voices): 32

LogAudioMixer: Display: Number of Async Source Workers: 0

LogAudio: Display: AudioDevice MaxSources: 32

LogAudio: Display: Audio Spatialization Plugin: None (built-in).

LogAudio: Display: Audio Reverb Plugin: None (built-in).

LogAudio: Display: Audio Occlusion Plugin: None (built-in).

LogAudioMixer: Display: Initializing audio mixer using platform API: 'XAudio2'

LogAudioMixer: Display: Using Audio Hardware Device 扬声器 (Realtek(R) Audio)

LogAudioMixer: Display: Initializing Sound Submixes...

LogAudioMixer: Display: Creating Master Submix 'MasterSubmixDefault'

LogAudioMixer: Display: Creating Master Submix 'MasterReverbSubmixDefault'

LogAudioMixer: FMixerPlatformXAudio2::StartAudioStream() called. InstanceID=59

LogAudioMixer: Display: Output buffers initialized: Frames=1024, Channels=2, Samples=2048, InstanceID=59

LogAudioMixer: Display: Starting AudioMixerPlatformInterface::RunInternal(), InstanceID=59

LogAudioMixer: Display: FMixerPlatformXAudio2::SubmitBuffer() called for the first time. InstanceID=59

LogInit: FAudioDevice initialized with ID 59.

LogAudio: Display: Audio Device (ID: 59) registered with world 'shili5'.

LogAudioMixer: Initializing Audio Bus Subsystem for audio device with ID 59

LogLoad: Game class is 'GameModeBase'

LogWorld: Bringing World /Game/UEDPIE_0_shili5.shili5 up for play (max tick rate 60) at 2025.12.10-19.29.01

LogWorld: Bringing up level for play took: 0.021560

LogOnline: OSS: Created online subsystem instance for: :Context_62

LogGameMode: FindPlayerStart: PATHS NOT DEFINED or NO PLAYERSTART with positive rating

PIE: 登陆的服务器

PIE: PIE总开始时间：0.182秒。

LogSlate: Updating window title bar state: overlay mode, drag disabled, window buttons hidden, title bar hidden

LogWorld: BeginTearingDown for /Game/UEDPIE_0_shili5

LogWorld: UWorld::CleanupWorld for shili5, bSessionEnded=true, bCleanupResources=true

LogSlate: InvalidateAllWidgets triggered. All widgets were invalidated

LogWorldMetrics: [UWorldMetricsSubsystem::Deinitialize]

LogWorldMetrics: [UWorldMetricsSubsystem::Clear]

LogPlayLevel: Display: Shutting down PIE online subsystems

LogSlate: InvalidateAllWidgets triggered. All widgets were invalidated

LogSlate: Updating window title bar state: overlay mode, drag disabled, window buttons hidden, title bar hidden

LogAudioMixer: Deinitializing Audio Bus Subsystem for audio device with ID 59

LogAudioMixer: FMixerPlatformXAudio2::StopAudioStream() called. InstanceID=59

LogAudioMixer: FMixerPlatformXAudio2::StopAudioStream() called. InstanceID=59

LogUObjectHash: Compacting FUObjectHashTables data took 1.38ms

LogPlayLevel: Display: Destroying online subsystem :Context_62

LogDebuggerCommands: Repeating last play command: 模拟

LogPlayLevel: PlayLevel: No blueprints needed recompiling

LogPlayLevel: Creating play world package: /Game/UEDPIE_0_shili5

LogPlayLevel: PIE: StaticDuplicateObject took: (0.043747s)

LogPlayLevel: PIE: Created PIE world by copying editor world from /Game/shili5.shili5 to /Game/UEDPIE_0_shili5.shili5 (0.043777s)

LogUObjectHash: Compacting FUObjectHashTables data took 1.34ms

LogWorldMetrics: [UWorldMetricsSubsystem::Initialize]

LogRenderer: SceneCulling instance hierarchy is disabled as UseNanite(PCD3D_SM5) returned false, for scene: 'World /Game/UEDPIE_0_shili5.shili5'.

LogPlayLevel: PIE: World Init took: (0.001109s)

LogAudio: Display: Creating Audio Device: Id: 60, Scope: Unique, Realtime: True

LogAudioMixer: Display: Audio Mixer Platform Settings:

LogAudioMixer: Display: Sample Rate: 48000

LogAudioMixer: Display: Callback Buffer Frame Size Requested: 1024

LogAudioMixer: Display: Callback Buffer Frame Size To Use: 1024

LogAudioMixer: Display: Number of buffers to queue: 2

LogAudioMixer: Display: Max Channels (voices): 32

LogAudioMixer: Display: Number of Async Source Workers: 0

LogAudio: Display: AudioDevice MaxSources: 32

LogAudio: Display: Audio Spatialization Plugin: None (built-in).

LogAudio: Display: Audio Reverb Plugin: None (built-in).

LogAudio: Display: Audio Occlusion Plugin: None (built-in).

LogAudioMixer: Display: Initializing audio mixer using platform API: 'XAudio2'

LogAudioMixer: Display: Using Audio Hardware Device 扬声器 (Realtek(R) Audio)

LogAudioMixer: Display: Initializing Sound Submixes...

LogAudioMixer: Display: Creating Master Submix 'MasterSubmixDefault'

LogAudioMixer: Display: Creating Master Submix 'MasterReverbSubmixDefault'

LogAudioMixer: FMixerPlatformXAudio2::StartAudioStream() called. InstanceID=60

LogAudioMixer: Display: Output buffers initialized: Frames=1024, Channels=2, Samples=2048, InstanceID=60

LogAudioMixer: Display: Starting AudioMixerPlatformInterface::RunInternal(), InstanceID=60

LogAudioMixer: Display: FMixerPlatformXAudio2::SubmitBuffer() called for the first time. InstanceID=60

LogInit: FAudioDevice initialized with ID 60.

LogAudio: Display: Audio Device (ID: 60) registered with world 'shili5'.

LogAudioMixer: Initializing Audio Bus Subsystem for audio device with ID 60

LogLoad: Game class is 'GameModeBase'

LogWorld: Bringing World /Game/UEDPIE_0_shili5.shili5 up for play (max tick rate 60) at 2025.12.10-19.30.24

LogWorld: Bringing up level for play took: 0.021812

LogOnline: OSS: Created online subsystem instance for: :Context_63

LogGameMode: FindPlayerStart: PATHS NOT DEFINED or NO PLAYERSTART with positive rating

PIE: 登陆的服务器

PIE: PIE总开始时间：0.178秒。

LogEOSSDK: LogEOS: Updating Product SDK Config, Time: 3218.835938

LogEOSSDK: LogEOS: SDK Config Product Update Request Completed - No Change

LogEOSSDK: LogEOS: ScheduleNextSDKConfigDataUpdate - Time: 3219.501953, Update Interval: 359.221771

LogAssetEditorSubsystem: Opening Asset editor for NiagaraSystem /Game/NG_Rain.NG_Rain

LogRenderer: SceneCulling instance hierarchy is disabled as UseNanite(PCD3D_SM5) returned false, for scene: 'World /Engine/Transient.World_4'.

LogRenderer: SceneCulling instance hierarchy is disabled as UseNanite(PCD3D_SM5) returned false, for scene: 'World /Engine/Transient.World_5'.

53363_NG_Rain_MessageLog: System已成功编译。

53363_NG_Rain_MessageLog: System已成功编译。

LogSlate: Updating window title bar state: overlay mode, drag disabled, window buttons hidden, title bar hidden

LogWorld: BeginTearingDown for /Game/UEDPIE_0_shili5

LogWorld: UWorld::CleanupWorld for shili5, bSessionEnded=true, bCleanupResources=true

LogSlate: InvalidateAllWidgets triggered. All widgets were invalidated

LogWorldMetrics: [UWorldMetricsSubsystem::Deinitialize]

LogWorldMetrics: [UWorldMetricsSubsystem::Clear]

LogPlayLevel: Display: Shutting down PIE online subsystems

LogSlate: InvalidateAllWidgets triggered. All widgets were invalidated

LogSlate: Updating window title bar state: overlay mode, drag disabled, window buttons hidden, title bar hidden

LogAudioMixer: Deinitializing Audio Bus Subsystem for audio device with ID 60

LogAudioMixer: FMixerPlatformXAudio2::StopAudioStream() called. InstanceID=60

LogAudioMixer: FMixerPlatformXAudio2::StopAudioStream() called. InstanceID=60

LogUObjectHash: Compacting FUObjectHashTables data took 1.38ms

LogPlayLevel: Display: Destroying online subsystem :Context_63

LogDebuggerCommands: Repeating last play command: 模拟

LogPlayLevel: PlayLevel: No blueprints needed recompiling

LogPlayLevel: Creating play world package: /Game/UEDPIE_0_shili5

LogPlayLevel: PIE: StaticDuplicateObject took: (0.044606s)

LogPlayLevel: PIE: Created PIE world by copying editor world from /Game/shili5.shili5 to /Game/UEDPIE_0_shili5.shili5 (0.044636s)

LogUObjectHash: Compacting FUObjectHashTables data took 1.50ms

LogWorldMetrics: [UWorldMetricsSubsystem::Initialize]

LogRenderer: SceneCulling instance hierarchy is disabled as UseNanite(PCD3D_SM5) returned false, for scene: 'World /Game/UEDPIE_0_shili5.shili5'.

LogPlayLevel: PIE: World Init took: (0.001145s)

LogAudio: Display: Creating Audio Device: Id: 61, Scope: Unique, Realtime: True

LogAudioMixer: Display: Audio Mixer Platform Settings:

LogAudioMixer: Display: Sample Rate: 48000

LogAudioMixer: Display: Callback Buffer Frame Size Requested: 1024

LogAudioMixer: Display: Callback Buffer Frame Size To Use: 1024

LogAudioMixer: Display: Number of buffers to queue: 2

LogAudioMixer: Display: Max Channels (voices): 32

LogAudioMixer: Display: Number of Async Source Workers: 0

LogAudio: Display: AudioDevice MaxSources: 32

LogAudio: Display: Audio Spatialization Plugin: None (built-in).

LogAudio: Display: Audio Reverb Plugin: None (built-in).

LogAudio: Display: Audio Occlusion Plugin: None (built-in).

LogAudioMixer: Display: Initializing audio mixer using platform API: 'XAudio2'

LogAudioMixer: Display: Using Audio Hardware Device 扬声器 (Realtek(R) Audio)

LogAudioMixer: Display: Initializing Sound Submixes...

LogAudioMixer: Display: Creating Master Submix 'MasterSubmixDefault'

LogAudioMixer: Display: Creating Master Submix 'MasterReverbSubmixDefault'

LogAudioMixer: FMixerPlatformXAudio2::StartAudioStream() called. InstanceID=61

LogAudioMixer: Display: Output buffers initialized: Frames=1024, Channels=2, Samples=2048, InstanceID=61

LogAudioMixer: Display: Starting AudioMixerPlatformInterface::RunInternal(), InstanceID=61

LogAudioMixer: Display: FMixerPlatformXAudio2::SubmitBuffer() called for the first time. InstanceID=61

LogInit: FAudioDevice initialized with ID 61.

LogAudio: Display: Audio Device (ID: 61) registered with world 'shili5'.

LogAudioMixer: Initializing Audio Bus Subsystem for audio device with ID 61

LogLoad: Game class is 'GameModeBase'

LogWorld: Bringing World /Game/UEDPIE_0_shili5.shili5 up for play (max tick rate 60) at 2025.12.10-19.31.05

LogWorld: Bringing up level for play took: 0.021833

LogOnline: OSS: Created online subsystem instance for: :Context_66

LogGameMode: FindPlayerStart: PATHS NOT DEFINED or NO PLAYERSTART with positive rating

PIE: 登陆的服务器

PIE: PIE总开始时间：0.187秒。

LogSlate: Updating window title bar state: overlay mode, drag disabled, window buttons hidden, title bar hidden

LogWorld: BeginTearingDown for /Game/UEDPIE_0_shili5

LogWorld: UWorld::CleanupWorld for shili5, bSessionEnded=true, bCleanupResources=true

LogSlate: InvalidateAllWidgets triggered. All widgets were invalidated

LogWorldMetrics: [UWorldMetricsSubsystem::Deinitialize]

LogWorldMetrics: [UWorldMetricsSubsystem::Clear]

LogPlayLevel: Display: Shutting down PIE online subsystems

LogSlate: InvalidateAllWidgets triggered. All widgets were invalidated

LogSlate: Updating window title bar state: overlay mode, drag disabled, window buttons hidden, title bar hidden

LogAudioMixer: Deinitializing Audio Bus Subsystem for audio device with ID 61

LogAudioMixer: FMixerPlatformXAudio2::StopAudioStream() called. InstanceID=61

LogAudioMixer: FMixerPlatformXAudio2::StopAudioStream() called. InstanceID=61

LogUObjectHash: Compacting FUObjectHashTables data took 1.55ms

LogPlayLevel: Display: Destroying online subsystem :Context_66

LogDebuggerCommands: Repeating last play command: 模拟

LogPlayLevel: PlayLevel: No blueprints needed recompiling

LogPlayLevel: Creating play world package: /Game/UEDPIE_0_shili5

LogPlayLevel: PIE: StaticDuplicateObject took: (0.045283s)

LogPlayLevel: PIE: Created PIE world by copying editor world from /Game/shili5.shili5 to /Game/UEDPIE_0_shili5.shili5 (0.045313s)

LogUObjectHash: Compacting FUObjectHashTables data took 1.31ms

LogWorldMetrics: [UWorldMetricsSubsystem::Initialize]

LogRenderer: SceneCulling instance hierarchy is disabled as UseNanite(PCD3D_SM5) returned false, for scene: 'World /Game/UEDPIE_0_shili5.shili5'.

LogPlayLevel: PIE: World Init took: (0.001045s)

LogAudio: Display: Creating Audio Device: Id: 62, Scope: Unique, Realtime: True

LogAudioMixer: Display: Audio Mixer Platform Settings:

LogAudioMixer: Display: Sample Rate: 48000

LogAudioMixer: Display: Callback Buffer Frame Size Requested: 1024

LogAudioMixer: Display: Callback Buffer Frame Size To Use: 1024

LogAudioMixer: Display: Number of buffers to queue: 2

LogAudioMixer: Display: Max Channels (voices): 32

LogAudioMixer: Display: Number of Async Source Workers: 0

LogAudio: Display: AudioDevice MaxSources: 32

LogAudio: Display: Audio Spatialization Plugin: None (built-in).

LogAudio: Display: Audio Reverb Plugin: None (built-in).

LogAudio: Display: Audio Occlusion Plugin: None (built-in).

LogAudioMixer: Display: Initializing audio mixer using platform API: 'XAudio2'

LogAudioMixer: Display: Using Audio Hardware Device 扬声器 (Realtek(R) Audio)

LogAudioMixer: Display: Initializing Sound Submixes...

LogAudioMixer: Display: Creating Master Submix 'MasterSubmixDefault'

LogAudioMixer: Display: Creating Master Submix 'MasterReverbSubmixDefault'

LogAudioMixer: FMixerPlatformXAudio2::StartAudioStream() called. InstanceID=62

LogAudioMixer: Display: Output buffers initialized: Frames=1024, Channels=2, Samples=2048, InstanceID=62

LogAudioMixer: Display: Starting AudioMixerPlatformInterface::RunInternal(), InstanceID=62

LogAudioMixer: Display: FMixerPlatformXAudio2::SubmitBuffer() called for the first time. InstanceID=62

LogInit: FAudioDevice initialized with ID 62.

LogAudio: Display: Audio Device (ID: 62) registered with world 'shili5'.

LogAudioMixer: Initializing Audio Bus Subsystem for audio device with ID 62

LogLoad: Game class is 'GameModeBase'

LogWorld: Bringing World /Game/UEDPIE_0_shili5.shili5 up for play (max tick rate 60) at 2025.12.10-19.31.38

LogWorld: Bringing up level for play took: 0.022837

LogOnline: OSS: Created online subsystem instance for: :Context_67

LogGameMode: FindPlayerStart: PATHS NOT DEFINED or NO PLAYERSTART with positive rating

PIE: 登陆的服务器

PIE: PIE总开始时间：0.178秒。

LogSlate: Updating window title bar state: overlay mode, drag disabled, window buttons hidden, title bar hidden

LogWorld: BeginTearingDown for /Game/UEDPIE_0_shili5

LogWorld: UWorld::CleanupWorld for shili5, bSessionEnded=true, bCleanupResources=true

LogSlate: InvalidateAllWidgets triggered. All widgets were invalidated

LogWorldMetrics: [UWorldMetricsSubsystem::Deinitialize]

LogWorldMetrics: [UWorldMetricsSubsystem::Clear]

LogPlayLevel: Display: Shutting down PIE online subsystems

LogSlate: InvalidateAllWidgets triggered. All widgets were invalidated

LogSlate: Updating window title bar state: overlay mode, drag disabled, window buttons hidden, title bar hidden

LogAudioMixer: Deinitializing Audio Bus Subsystem for audio device with ID 62

LogAudioMixer: FMixerPlatformXAudio2::StopAudioStream() called. InstanceID=62

LogAudioMixer: FMixerPlatformXAudio2::StopAudioStream() called. InstanceID=62

LogUObjectHash: Compacting FUObjectHashTables data took 1.44ms

LogPlayLevel: Display: Destroying online subsystem :Context_67

LogDebuggerCommands: Repeating last play command: 模拟

LogPlayLevel: PlayLevel: No blueprints needed recompiling

LogPlayLevel: Creating play world package: /Game/UEDPIE_0_shili5

LogPlayLevel: PIE: StaticDuplicateObject took: (0.052010s)

LogPlayLevel: PIE: Created PIE world by copying editor world from /Game/shili5.shili5 to /Game/UEDPIE_0_shili5.shili5 (0.052045s)

LogUObjectHash: Compacting FUObjectHashTables data took 1.40ms

LogWorldMetrics: [UWorldMetricsSubsystem::Initialize]

LogRenderer: SceneCulling instance hierarchy is disabled as UseNanite(PCD3D_SM5) returned false, for scene: 'World /Game/UEDPIE_0_shili5.shili5'.

LogPlayLevel: PIE: World Init took: (0.001083s)

LogAudio: Display: Creating Audio Device: Id: 63, Scope: Unique, Realtime: True

LogAudioMixer: Display: Audio Mixer Platform Settings:

LogAudioMixer: Display: Sample Rate: 48000

LogAudioMixer: Display: Callback Buffer Frame Size Requested: 1024

LogAudioMixer: Display: Callback Buffer Frame Size To Use: 1024

LogAudioMixer: Display: Number of buffers to queue: 2

LogAudioMixer: Display: Max Channels (voices): 32

LogAudioMixer: Display: Number of Async Source Workers: 0

LogAudio: Display: AudioDevice MaxSources: 32

LogAudio: Display: Audio Spatialization Plugin: None (built-in).

LogAudio: Display: Audio Reverb Plugin: None (built-in).

LogAudio: Display: Audio Occlusion Plugin: None (built-in).

LogAudioMixer: Display: Initializing audio mixer using platform API: 'XAudio2'

LogAudioMixer: Display: Using Audio Hardware Device 扬声器 (Realtek(R) Audio)

LogAudioMixer: Display: Initializing Sound Submixes...

LogAudioMixer: Display: Creating Master Submix 'MasterSubmixDefault'

LogAudioMixer: Display: Creating Master Submix 'MasterReverbSubmixDefault'

LogAudioMixer: FMixerPlatformXAudio2::StartAudioStream() called. InstanceID=63

LogAudioMixer: Display: Output buffers initialized: Frames=1024, Channels=2, Samples=2048, InstanceID=63

LogAudioMixer: Display: Starting AudioMixerPlatformInterface::RunInternal(), InstanceID=63

LogAudioMixer: Display: FMixerPlatformXAudio2::SubmitBuffer() called for the first time. InstanceID=63

LogInit: FAudioDevice initialized with ID 63.

LogAudio: Display: Audio Device (ID: 63) registered with world 'shili5'.

LogAudioMixer: Initializing Audio Bus Subsystem for audio device with ID 63

LogLoad: Game class is 'GameModeBase'

LogWorld: Bringing World /Game/UEDPIE_0_shili5.shili5 up for play (max tick rate 60) at 2025.12.10-19.32.14

LogWorld: Bringing up level for play took: 0.023519

LogOnline: OSS: Created online subsystem instance for: :Context_68

LogGameMode: FindPlayerStart: PATHS NOT DEFINED or NO PLAYERSTART with positive rating

PIE: 登陆的服务器

PIE: PIE总开始时间：0.189秒。

LogSlate: Updating window title bar state: overlay mode, drag disabled, window buttons hidden, title bar hidden

LogWorld: BeginTearingDown for /Game/UEDPIE_0_shili5

LogWorld: UWorld::CleanupWorld for shili5, bSessionEnded=true, bCleanupResources=true

LogSlate: InvalidateAllWidgets triggered. All widgets were invalidated

LogWorldMetrics: [UWorldMetricsSubsystem::Deinitialize]

LogWorldMetrics: [UWorldMetricsSubsystem::Clear]

LogPlayLevel: Display: Shutting down PIE online subsystems

LogSlate: InvalidateAllWidgets triggered. All widgets were invalidated

LogSlate: Updating window title bar state: overlay mode, drag disabled, window buttons hidden, title bar hidden

LogAudioMixer: Deinitializing Audio Bus Subsystem for audio device with ID 63

LogAudioMixer: FMixerPlatformXAudio2::StopAudioStream() called. InstanceID=63

LogAudioMixer: FMixerPlatformXAudio2::StopAudioStream() called. InstanceID=63

LogUObjectHash: Compacting FUObjectHashTables data took 1.51ms

LogPlayLevel: Display: Destroying online subsystem :Context_68

LogDebuggerCommands: Repeating last play command: 模拟

LogPlayLevel: PlayLevel: No blueprints needed recompiling

LogPlayLevel: Creating play world package: /Game/UEDPIE_0_shili5

LogPlayLevel: PIE: StaticDuplicateObject took: (0.043327s)

LogPlayLevel: PIE: Created PIE world by copying editor world from /Game/shili5.shili5 to /Game/UEDPIE_0_shili5.shili5 (0.043373s)

LogUObjectHash: Compacting FUObjectHashTables data took 1.26ms

LogWorldMetrics: [UWorldMetricsSubsystem::Initialize]

LogRenderer: SceneCulling instance hierarchy is disabled as UseNanite(PCD3D_SM5) returned false, for scene: 'World /Game/UEDPIE_0_shili5.shili5'.

LogPlayLevel: PIE: World Init took: (0.001095s)

LogAudio: Display: Creating Audio Device: Id: 64, Scope: Unique, Realtime: True

LogAudioMixer: Display: Audio Mixer Platform Settings:

LogAudioMixer: Display: Sample Rate: 48000

LogAudioMixer: Display: Callback Buffer Frame Size Requested: 1024

LogAudioMixer: Display: Callback Buffer Frame Size To Use: 1024

LogAudioMixer: Display: Number of buffers to queue: 2

LogAudioMixer: Display: Max Channels (voices): 32

LogAudioMixer: Display: Number of Async Source Workers: 0

LogAudio: Display: AudioDevice MaxSources: 32

LogAudio: Display: Audio Spatialization Plugin: None (built-in).

LogAudio: Display: Audio Reverb Plugin: None (built-in).

LogAudio: Display: Audio Occlusion Plugin: None (built-in).

LogAudioMixer: Display: Initializing audio mixer using platform API: 'XAudio2'

LogAudioMixer: Display: Using Audio Hardware Device 扬声器 (Realtek(R) Audio)

LogAudioMixer: Display: Initializing Sound Submixes...

LogAudioMixer: Display: Creating Master Submix 'MasterSubmixDefault'

LogAudioMixer: Display: Creating Master Submix 'MasterReverbSubmixDefault'

LogAudioMixer: FMixerPlatformXAudio2::StartAudioStream() called. InstanceID=64

LogAudioMixer: Display: Output buffers initialized: Frames=1024, Channels=2, Samples=2048, InstanceID=64

LogAudioMixer: Display: Starting AudioMixerPlatformInterface::RunInternal(), InstanceID=64

LogAudioMixer: Display: FMixerPlatformXAudio2::SubmitBuffer() called for the first time. InstanceID=64

LogInit: FAudioDevice initialized with ID 64.

LogAudio: Display: Audio Device (ID: 64) registered with world 'shili5'.

LogAudioMixer: Initializing Audio Bus Subsystem for audio device with ID 64

LogLoad: Game class is 'GameModeBase'

LogWorld: Bringing World /Game/UEDPIE_0_shili5.shili5 up for play (max tick rate 60) at 2025.12.10-19.32.17

LogWorld: Bringing up level for play took: 0.022067

LogOnline: OSS: Created online subsystem instance for: :Context_69

LogGameMode: FindPlayerStart: PATHS NOT DEFINED or NO PLAYERSTART with positive rating

PIE: 登陆的服务器

PIE: PIE总开始时间：0.175秒。

LogSlate: Updating window title bar state: overlay mode, drag disabled, window buttons hidden, title bar hidden

LogWorld: BeginTearingDown for /Game/UEDPIE_0_shili5

LogWorld: UWorld::CleanupWorld for shili5, bSessionEnded=true, bCleanupResources=true

LogSlate: InvalidateAllWidgets triggered. All widgets were invalidated

LogWorldMetrics: [UWorldMetricsSubsystem::Deinitialize]

LogWorldMetrics: [UWorldMetricsSubsystem::Clear]

LogPlayLevel: Display: Shutting down PIE online subsystems

LogSlate: InvalidateAllWidgets triggered. All widgets were invalidated

LogSlate: Updating window title bar state: overlay mode, drag disabled, window buttons hidden, title bar hidden

LogAudioMixer: Deinitializing Audio Bus Subsystem for audio device with ID 64

LogAudioMixer: FMixerPlatformXAudio2::StopAudioStream() called. InstanceID=64

LogAudioMixer: FMixerPlatformXAudio2::StopAudioStream() called. InstanceID=64

LogUObjectHash: Compacting FUObjectHashTables data took 1.75ms

LogPlayLevel: Display: Destroying online subsystem :Context_69

LogDebuggerCommands: Repeating last play command: 模拟

LogPlayLevel: PlayLevel: No blueprints needed recompiling

LogPlayLevel: Creating play world package: /Game/UEDPIE_0_shili5

LogPlayLevel: PIE: StaticDuplicateObject took: (0.046978s)

LogPlayLevel: PIE: Created PIE world by copying editor world from /Game/shili5.shili5 to /Game/UEDPIE_0_shili5.shili5 (0.047023s)

LogUObjectHash: Compacting FUObjectHashTables data took 1.49ms

LogWorldMetrics: [UWorldMetricsSubsystem::Initialize]

LogRenderer: SceneCulling instance hierarchy is disabled as UseNanite(PCD3D_SM5) returned false, for scene: 'World /Game/UEDPIE_0_shili5.shili5'.

LogPlayLevel: PIE: World Init took: (0.001075s)

LogAudio: Display: Creating Audio Device: Id: 65, Scope: Unique, Realtime: True

LogAudioMixer: Display: Audio Mixer Platform Settings:

LogAudioMixer: Display: Sample Rate: 48000

LogAudioMixer: Display: Callback Buffer Frame Size Requested: 1024

LogAudioMixer: Display: Callback Buffer Frame Size To Use: 1024

LogAudioMixer: Display: Number of buffers to queue: 2

LogAudioMixer: Display: Max Channels (voices): 32

LogAudioMixer: Display: Number of Async Source Workers: 0

LogAudio: Display: AudioDevice MaxSources: 32

LogAudio: Display: Audio Spatialization Plugin: None (built-in).

LogAudio: Display: Audio Reverb Plugin: None (built-in).

LogAudio: Display: Audio Occlusion Plugin: None (built-in).

LogAudioMixer: Display: Initializing audio mixer using platform API: 'XAudio2'

LogAudioMixer: Display: Using Audio Hardware Device 扬声器 (Realtek(R) Audio)

LogAudioMixer: Display: Initializing Sound Submixes...

LogAudioMixer: Display: Creating Master Submix 'MasterSubmixDefault'

LogAudioMixer: Display: Creating Master Submix 'MasterReverbSubmixDefault'

LogAudioMixer: FMixerPlatformXAudio2::StartAudioStream() called. InstanceID=65

LogAudioMixer: Display: Output buffers initialized: Frames=1024, Channels=2, Samples=2048, InstanceID=65

LogAudioMixer: Display: Starting AudioMixerPlatformInterface::RunInternal(), InstanceID=65

LogAudioMixer: Display: FMixerPlatformXAudio2::SubmitBuffer() called for the first time. InstanceID=65

LogInit: FAudioDevice initialized with ID 65.

LogAudio: Display: Audio Device (ID: 65) registered with world 'shili5'.

LogAudioMixer: Initializing Audio Bus Subsystem for audio device with ID 65

LogLoad: Game class is 'GameModeBase'

LogWorld: Bringing World /Game/UEDPIE_0_shili5.shili5 up for play (max tick rate 60) at 2025.12.10-19.32.51

LogWorld: Bringing up level for play took: 0.023403

LogOnline: OSS: Created online subsystem instance for: :Context_70

LogGameMode: FindPlayerStart: PATHS NOT DEFINED or NO PLAYERSTART with positive rating

PIE: 登陆的服务器

PIE: PIE总开始时间：0.178秒。

LogSlate: Updating window title bar state: overlay mode, drag disabled, window buttons hidden, title bar hidden

LogWorld: BeginTearingDown for /Game/UEDPIE_0_shili5

LogWorld: UWorld::CleanupWorld for shili5, bSessionEnded=true, bCleanupResources=true

LogSlate: InvalidateAllWidgets triggered. All widgets were invalidated

LogWorldMetrics: [UWorldMetricsSubsystem::Deinitialize]

LogWorldMetrics: [UWorldMetricsSubsystem::Clear]

LogPlayLevel: Display: Shutting down PIE online subsystems

LogSlate: InvalidateAllWidgets triggered. All widgets were invalidated

LogSlate: Updating window title bar state: overlay mode, drag disabled, window buttons hidden, title bar hidden

LogAudioMixer: Deinitializing Audio Bus Subsystem for audio device with ID 65

LogAudioMixer: FMixerPlatformXAudio2::StopAudioStream() called. InstanceID=65

LogAudioMixer: FMixerPlatformXAudio2::StopAudioStream() called. InstanceID=65

LogUObjectHash: Compacting FUObjectHashTables data took 1.52ms

LogPlayLevel: Display: Destroying online subsystem :Context_70

LogDebuggerCommands: Repeating last play command: 模拟

LogPlayLevel: PlayLevel: No blueprints needed recompiling

LogPlayLevel: Creating play world package: /Game/UEDPIE_0_shili5

LogPlayLevel: PIE: StaticDuplicateObject took: (0.044849s)

LogPlayLevel: PIE: Created PIE world by copying editor world from /Game/shili5.shili5 to /Game/UEDPIE_0_shili5.shili5 (0.044881s)

LogUObjectHash: Compacting FUObjectHashTables data took 1.35ms

LogWorldMetrics: [UWorldMetricsSubsystem::Initialize]

LogRenderer: SceneCulling instance hierarchy is disabled as UseNanite(PCD3D_SM5) returned false, for scene: 'World /Game/UEDPIE_0_shili5.shili5'.

LogPlayLevel: PIE: World Init took: (0.001094s)

LogAudio: Display: Creating Audio Device: Id: 66, Scope: Unique, Realtime: True

LogAudioMixer: Display: Audio Mixer Platform Settings:

LogAudioMixer: Display: Sample Rate: 48000

LogAudioMixer: Display: Callback Buffer Frame Size Requested: 1024

LogAudioMixer: Display: Callback Buffer Frame Size To Use: 1024

LogAudioMixer: Display: Number of buffers to queue: 2

LogAudioMixer: Display: Max Channels (voices): 32

LogAudioMixer: Display: Number of Async Source Workers: 0

LogAudio: Display: AudioDevice MaxSources: 32

LogAudio: Display: Audio Spatialization Plugin: None (built-in).

LogAudio: Display: Audio Reverb Plugin: None (built-in).

LogAudio: Display: Audio Occlusion Plugin: None (built-in).

LogAudioMixer: Display: Initializing audio mixer using platform API: 'XAudio2'

LogAudioMixer: Display: Using Audio Hardware Device 扬声器 (Realtek(R) Audio)

LogAudioMixer: Display: Initializing Sound Submixes...

LogAudioMixer: Display: Creating Master Submix 'MasterSubmixDefault'

LogAudioMixer: Display: Creating Master Submix 'MasterReverbSubmixDefault'

LogAudioMixer: FMixerPlatformXAudio2::StartAudioStream() called. InstanceID=66

LogAudioMixer: Display: Output buffers initialized: Frames=1024, Channels=2, Samples=2048, InstanceID=66

LogAudioMixer: Display: Starting AudioMixerPlatformInterface::RunInternal(), InstanceID=66

LogAudioMixer: Display: FMixerPlatformXAudio2::SubmitBuffer() called for the first time. InstanceID=66

LogInit: FAudioDevice initialized with ID 66.

LogAudio: Display: Audio Device (ID: 66) registered with world 'shili5'.

LogAudioMixer: Initializing Audio Bus Subsystem for audio device with ID 66

LogLoad: Game class is 'GameModeBase'

LogWorld: Bringing World /Game/UEDPIE_0_shili5.shili5 up for play (max tick rate 60) at 2025.12.10-19.33.15

LogWorld: Bringing up level for play took: 0.024058

LogOnline: OSS: Created online subsystem instance for: :Context_71

LogGameMode: FindPlayerStart: PATHS NOT DEFINED or NO PLAYERSTART with positive rating

PIE: 登陆的服务器

PIE: PIE总开始时间：0.178秒。

LogSlate: Updating window title bar state: overlay mode, drag disabled, window buttons hidden, title bar hidden

LogWorld: BeginTearingDown for /Game/UEDPIE_0_shili5

LogWorld: UWorld::CleanupWorld for shili5, bSessionEnded=true, bCleanupResources=true

LogSlate: InvalidateAllWidgets triggered. All widgets were invalidated

LogWorldMetrics: [UWorldMetricsSubsystem::Deinitialize]

LogWorldMetrics: [UWorldMetricsSubsystem::Clear]

LogPlayLevel: Display: Shutting down PIE online subsystems

LogSlate: InvalidateAllWidgets triggered. All widgets were invalidated

LogSlate: Updating window title bar state: overlay mode, drag disabled, window buttons hidden, title bar hidden

LogAudioMixer: Deinitializing Audio Bus Subsystem for audio device with ID 66

LogAudioMixer: FMixerPlatformXAudio2::StopAudioStream() called. InstanceID=66

LogAudioMixer: FMixerPlatformXAudio2::StopAudioStream() called. InstanceID=66

LogUObjectHash: Compacting FUObjectHashTables data took 1.35ms

LogPlayLevel: Display: Destroying online subsystem :Context_71

LogDebuggerCommands: Repeating last play command: 模拟

LogPlayLevel: PlayLevel: No blueprints needed recompiling

LogPlayLevel: Creating play world package: /Game/UEDPIE_0_shili5

LogPlayLevel: PIE: StaticDuplicateObject took: (0.044073s)

LogPlayLevel: PIE: Created PIE world by copying editor world from /Game/shili5.shili5 to /Game/UEDPIE_0_shili5.shili5 (0.044105s)

LogUObjectHash: Compacting FUObjectHashTables data took 1.38ms

LogWorldMetrics: [UWorldMetricsSubsystem::Initialize]

LogRenderer: SceneCulling instance hierarchy is disabled as UseNanite(PCD3D_SM5) returned false, for scene: 'World /Game/UEDPIE_0_shili5.shili5'.

LogPlayLevel: PIE: World Init took: (0.001072s)

LogAudio: Display: Creating Audio Device: Id: 67, Scope: Unique, Realtime: True

LogAudioMixer: Display: Audio Mixer Platform Settings:

LogAudioMixer: Display: Sample Rate: 48000

LogAudioMixer: Display: Callback Buffer Frame Size Requested: 1024

LogAudioMixer: Display: Callback Buffer Frame Size To Use: 1024

LogAudioMixer: Display: Number of buffers to queue: 2

LogAudioMixer: Display: Max Channels (voices): 32

LogAudioMixer: Display: Number of Async Source Workers: 0

LogAudio: Display: AudioDevice MaxSources: 32

LogAudio: Display: Audio Spatialization Plugin: None (built-in).

LogAudio: Display: Audio Reverb Plugin: None (built-in).

LogAudio: Display: Audio Occlusion Plugin: None (built-in).

LogAudioMixer: Display: Initializing audio mixer using platform API: 'XAudio2'

LogAudioMixer: Display: Using Audio Hardware Device 扬声器 (Realtek(R) Audio)

LogAudioMixer: Display: Initializing Sound Submixes...

LogAudioMixer: Display: Creating Master Submix 'MasterSubmixDefault'

LogAudioMixer: Display: Creating Master Submix 'MasterReverbSubmixDefault'

LogAudioMixer: FMixerPlatformXAudio2::StartAudioStream() called. InstanceID=67

LogAudioMixer: Display: Output buffers initialized: Frames=1024, Channels=2, Samples=2048, InstanceID=67

LogAudioMixer: Display: Starting AudioMixerPlatformInterface::RunInternal(), InstanceID=67

LogAudioMixer: Display: FMixerPlatformXAudio2::SubmitBuffer() called for the first time. InstanceID=67

LogInit: FAudioDevice initialized with ID 67.

LogAudio: Display: Audio Device (ID: 67) registered with world 'shili5'.

LogAudioMixer: Initializing Audio Bus Subsystem for audio device with ID 67

LogLoad: Game class is 'GameModeBase'

LogWorld: Bringing World /Game/UEDPIE_0_shili5.shili5 up for play (max tick rate 60) at 2025.12.10-19.33.58

LogWorld: Bringing up level for play took: 0.022879

LogOnline: OSS: Created online subsystem instance for: :Context_72

LogGameMode: FindPlayerStart: PATHS NOT DEFINED or NO PLAYERSTART with positive rating

PIE: 登陆的服务器

PIE: PIE总开始时间：0.181秒。

LogSlate: Updating window title bar state: overlay mode, drag disabled, window buttons hidden, title bar hidden

LogWorld: BeginTearingDown for /Game/UEDPIE_0_shili5

LogWorld: UWorld::CleanupWorld for shili5, bSessionEnded=true, bCleanupResources=true

LogSlate: InvalidateAllWidgets triggered. All widgets were invalidated

LogWorldMetrics: [UWorldMetricsSubsystem::Deinitialize]

LogWorldMetrics: [UWorldMetricsSubsystem::Clear]

LogPlayLevel: Display: Shutting down PIE online subsystems

LogSlate: InvalidateAllWidgets triggered. All widgets were invalidated

LogSlate: Updating window title bar state: overlay mode, drag disabled, window buttons hidden, title bar hidden

LogAudioMixer: Deinitializing Audio Bus Subsystem for audio device with ID 67

LogAudioMixer: FMixerPlatformXAudio2::StopAudioStream() called. InstanceID=67

LogAudioMixer: FMixerPlatformXAudio2::StopAudioStream() called. InstanceID=67

LogUObjectHash: Compacting FUObjectHashTables data took 1.38ms

LogPlayLevel: Display: Destroying online subsystem :Context_72

LogDebuggerCommands: Repeating last play command: 模拟

LogPlayLevel: PlayLevel: No blueprints needed recompiling

LogPlayLevel: Creating play world package: /Game/UEDPIE_0_shili5

LogPlayLevel: PIE: StaticDuplicateObject took: (0.044462s)

LogPlayLevel: PIE: Created PIE world by copying editor world from /Game/shili5.shili5 to /Game/UEDPIE_0_shili5.shili5 (0.044494s)

LogUObjectHash: Compacting FUObjectHashTables data took 1.41ms

LogWorldMetrics: [UWorldMetricsSubsystem::Initialize]

LogRenderer: SceneCulling instance hierarchy is disabled as UseNanite(PCD3D_SM5) returned false, for scene: 'World /Game/UEDPIE_0_shili5.shili5'.

LogPlayLevel: PIE: World Init took: (0.001121s)

LogAudio: Display: Creating Audio Device: Id: 68, Scope: Unique, Realtime: True

LogAudioMixer: Display: Audio Mixer Platform Settings:

LogAudioMixer: Display: Sample Rate: 48000

LogAudioMixer: Display: Callback Buffer Frame Size Requested: 1024

LogAudioMixer: Display: Callback Buffer Frame Size To Use: 1024

LogAudioMixer: Display: Number of buffers to queue: 2

LogAudioMixer: Display: Max Channels (voices): 32

LogAudioMixer: Display: Number of Async Source Workers: 0

LogAudio: Display: AudioDevice MaxSources: 32

LogAudio: Display: Audio Spatialization Plugin: None (built-in).

LogAudio: Display: Audio Reverb Plugin: None (built-in).

LogAudio: Display: Audio Occlusion Plugin: None (built-in).

LogAudioMixer: Display: Initializing audio mixer using platform API: 'XAudio2'

LogAudioMixer: Display: Using Audio Hardware Device 扬声器 (Realtek(R) Audio)

LogAudioMixer: Display: Initializing Sound Submixes...

LogAudioMixer: Display: Creating Master Submix 'MasterSubmixDefault'

LogAudioMixer: Display: Creating Master Submix 'MasterReverbSubmixDefault'

LogAudioMixer: FMixerPlatformXAudio2::StartAudioStream() called. InstanceID=68

LogAudioMixer: Display: Output buffers initialized: Frames=1024, Channels=2, Samples=2048, InstanceID=68

LogAudioMixer: Display: Starting AudioMixerPlatformInterface::RunInternal(), InstanceID=68

LogAudioMixer: Display: FMixerPlatformXAudio2::SubmitBuffer() called for the first time. InstanceID=68

LogInit: FAudioDevice initialized with ID 68.

LogAudio: Display: Audio Device (ID: 68) registered with world 'shili5'.

LogAudioMixer: Initializing Audio Bus Subsystem for audio device with ID 68

LogLoad: Game class is 'GameModeBase'

LogWorld: Bringing World /Game/UEDPIE_0_shili5.shili5 up for play (max tick rate 60) at 2025.12.10-19.34.36

LogWorld: Bringing up level for play took: 0.021278

LogOnline: OSS: Created online subsystem instance for: :Context_73

LogGameMode: FindPlayerStart: PATHS NOT DEFINED or NO PLAYERSTART with positive rating

PIE: 登陆的服务器

PIE: PIE总开始时间：0.175秒。

LogSlate: Updating window title bar state: overlay mode, drag disabled, window buttons hidden, title bar hidden

LogWorld: BeginTearingDown for /Game/UEDPIE_0_shili5

LogWorld: UWorld::CleanupWorld for shili5, bSessionEnded=true, bCleanupResources=true

LogSlate: InvalidateAllWidgets triggered. All widgets were invalidated

LogWorldMetrics: [UWorldMetricsSubsystem::Deinitialize]

LogWorldMetrics: [UWorldMetricsSubsystem::Clear]

LogPlayLevel: Display: Shutting down PIE online subsystems

LogSlate: InvalidateAllWidgets triggered. All widgets were invalidated

LogSlate: Updating window title bar state: overlay mode, drag disabled, window buttons hidden, title bar hidden

LogAudioMixer: Deinitializing Audio Bus Subsystem for audio device with ID 68

LogAudioMixer: FMixerPlatformXAudio2::StopAudioStream() called. InstanceID=68

LogAudioMixer: FMixerPlatformXAudio2::StopAudioStream() called. InstanceID=68

LogUObjectHash: Compacting FUObjectHashTables data took 1.39ms

LogPlayLevel: Display: Destroying online subsystem :Context_73

LogDebuggerCommands: Repeating last play command: 模拟

LogPlayLevel: PlayLevel: No blueprints needed recompiling

LogPlayLevel: Creating play world package: /Game/UEDPIE_0_shili5

LogPlayLevel: PIE: StaticDuplicateObject took: (0.044038s)

LogPlayLevel: PIE: Created PIE world by copying editor world from /Game/shili5.shili5 to /Game/UEDPIE_0_shili5.shili5 (0.044073s)

LogUObjectHash: Compacting FUObjectHashTables data took 1.20ms

LogWorldMetrics: [UWorldMetricsSubsystem::Initialize]

LogRenderer: SceneCulling instance hierarchy is disabled as UseNanite(PCD3D_SM5) returned false, for scene: 'World /Game/UEDPIE_0_shili5.shili5'.

LogPlayLevel: PIE: World Init took: (0.001071s)

LogAudio: Display: Creating Audio Device: Id: 69, Scope: Unique, Realtime: True

LogAudioMixer: Display: Audio Mixer Platform Settings:

LogAudioMixer: Display: Sample Rate: 48000

LogAudioMixer: Display: Callback Buffer Frame Size Requested: 1024

LogAudioMixer: Display: Callback Buffer Frame Size To Use: 1024

LogAudioMixer: Display: Number of buffers to queue: 2

LogAudioMixer: Display: Max Channels (voices): 32

LogAudioMixer: Display: Number of Async Source Workers: 0

LogAudio: Display: AudioDevice MaxSources: 32

LogAudio: Display: Audio Spatialization Plugin: None (built-in).

LogAudio: Display: Audio Reverb Plugin: None (built-in).

LogAudio: Display: Audio Occlusion Plugin: None (built-in).

LogAudioMixer: Display: Initializing audio mixer using platform API: 'XAudio2'

LogAudioMixer: Display: Using Audio Hardware Device 扬声器 (Realtek(R) Audio)

LogAudioMixer: Display: Initializing Sound Submixes...

LogAudioMixer: Display: Creating Master Submix 'MasterSubmixDefault'

LogAudioMixer: Display: Creating Master Submix 'MasterReverbSubmixDefault'

LogAudioMixer: FMixerPlatformXAudio2::StartAudioStream() called. InstanceID=69

LogAudioMixer: Display: Output buffers initialized: Frames=1024, Channels=2, Samples=2048, InstanceID=69

LogAudioMixer: Display: Starting AudioMixerPlatformInterface::RunInternal(), InstanceID=69

LogAudioMixer: Display: FMixerPlatformXAudio2::SubmitBuffer() called for the first time. InstanceID=69

LogInit: FAudioDevice initialized with ID 69.

LogAudio: Display: Audio Device (ID: 69) registered with world 'shili5'.

LogAudioMixer: Initializing Audio Bus Subsystem for audio device with ID 69

LogLoad: Game class is 'GameModeBase'

LogWorld: Bringing World /Game/UEDPIE_0_shili5.shili5 up for play (max tick rate 60) at 2025.12.10-19.35.19

LogWorld: Bringing up level for play took: 0.023157

LogOnline: OSS: Created online subsystem instance for: :Context_74

LogGameMode: FindPlayerStart: PATHS NOT DEFINED or NO PLAYERSTART with positive rating

PIE: 登陆的服务器

PIE: PIE总开始时间：0.184秒。

LogSlate: Updating window title bar state: overlay mode, drag disabled, window buttons hidden, title bar hidden

LogWorld: BeginTearingDown for /Game/UEDPIE_0_shili5

LogWorld: UWorld::CleanupWorld for shili5, bSessionEnded=true, bCleanupResources=true

LogSlate: InvalidateAllWidgets triggered. All widgets were invalidated

LogWorldMetrics: [UWorldMetricsSubsystem::Deinitialize]

LogWorldMetrics: [UWorldMetricsSubsystem::Clear]

LogPlayLevel: Display: Shutting down PIE online subsystems

LogSlate: InvalidateAllWidgets triggered. All widgets were invalidated

LogSlate: Updating window title bar state: overlay mode, drag disabled, window buttons hidden, title bar hidden

LogAudioMixer: Deinitializing Audio Bus Subsystem for audio device with ID 69

LogAudioMixer: FMixerPlatformXAudio2::StopAudioStream() called. InstanceID=69

LogAudioMixer: FMixerPlatformXAudio2::StopAudioStream() called. InstanceID=69

LogUObjectHash: Compacting FUObjectHashTables data took 1.31ms

LogPlayLevel: Display: Destroying online subsystem :Context_74

LogDebuggerCommands: Repeating last play command: 模拟

LogPlayLevel: PlayLevel: No blueprints needed recompiling

LogPlayLevel: Creating play world package: /Game/UEDPIE_0_shili5

LogPlayLevel: PIE: StaticDuplicateObject took: (0.044625s)

LogPlayLevel: PIE: Created PIE world by copying editor world from /Game/shili5.shili5 to /Game/UEDPIE_0_shili5.shili5 (0.044659s)

LogUObjectHash: Compacting FUObjectHashTables data took 1.34ms

LogWorldMetrics: [UWorldMetricsSubsystem::Initialize]

LogRenderer: SceneCulling instance hierarchy is disabled as UseNanite(PCD3D_SM5) returned false, for scene: 'World /Game/UEDPIE_0_shili5.shili5'.

LogPlayLevel: PIE: World Init took: (0.001087s)

LogAudio: Display: Creating Audio Device: Id: 70, Scope: Unique, Realtime: True

LogAudioMixer: Display: Audio Mixer Platform Settings:

LogAudioMixer: Display: Sample Rate: 48000

LogAudioMixer: Display: Callback Buffer Frame Size Requested: 1024

LogAudioMixer: Display: Callback Buffer Frame Size To Use: 1024

LogAudioMixer: Display: Number of buffers to queue: 2

LogAudioMixer: Display: Max Channels (voices): 32

LogAudioMixer: Display: Number of Async Source Workers: 0

LogAudio: Display: AudioDevice MaxSources: 32

LogAudio: Display: Audio Spatialization Plugin: None (built-in).

LogAudio: Display: Audio Reverb Plugin: None (built-in).

LogAudio: Display: Audio Occlusion Plugin: None (built-in).

LogAudioMixer: Display: Initializing audio mixer using platform API: 'XAudio2'

LogAudioMixer: Display: Using Audio Hardware Device 扬声器 (Realtek(R) Audio)

LogAudioMixer: Display: Initializing Sound Submixes...

LogAudioMixer: Display: Creating Master Submix 'MasterSubmixDefault'

LogAudioMixer: Display: Creating Master Submix 'MasterReverbSubmixDefault'

LogAudioMixer: FMixerPlatformXAudio2::StartAudioStream() called. InstanceID=70

LogAudioMixer: Display: Output buffers initialized: Frames=1024, Channels=2, Samples=2048, InstanceID=70

LogAudioMixer: Display: Starting AudioMixerPlatformInterface::RunInternal(), InstanceID=70

LogAudioMixer: Display: FMixerPlatformXAudio2::SubmitBuffer() called for the first time. InstanceID=70

LogInit: FAudioDevice initialized with ID 70.

LogAudio: Display: Audio Device (ID: 70) registered with world 'shili5'.

LogAudioMixer: Initializing Audio Bus Subsystem for audio device with ID 70

LogLoad: Game class is 'GameModeBase'

LogWorld: Bringing World /Game/UEDPIE_0_shili5.shili5 up for play (max tick rate 60) at 2025.12.10-19.35.22

LogWorld: Bringing up level for play took: 0.021538

LogOnline: OSS: Created online subsystem instance for: :Context_75

LogGameMode: FindPlayerStart: PATHS NOT DEFINED or NO PLAYERSTART with positive rating

PIE: 登陆的服务器

PIE: PIE总开始时间：0.185秒。

LogSlate: Updating window title bar state: overlay mode, drag disabled, window buttons hidden, title bar hidden

LogWorld: BeginTearingDown for /Game/UEDPIE_0_shili5

LogWorld: UWorld::CleanupWorld for shili5, bSessionEnded=true, bCleanupResources=true

LogSlate: InvalidateAllWidgets triggered. All widgets were invalidated

LogWorldMetrics: [UWorldMetricsSubsystem::Deinitialize]

LogWorldMetrics: [UWorldMetricsSubsystem::Clear]

LogPlayLevel: Display: Shutting down PIE online subsystems

LogSlate: InvalidateAllWidgets triggered. All widgets were invalidated

LogSlate: Updating window title bar state: overlay mode, drag disabled, window buttons hidden, title bar hidden

LogAudioMixer: Deinitializing Audio Bus Subsystem for audio device with ID 70

LogAudioMixer: FMixerPlatformXAudio2::StopAudioStream() called. InstanceID=70

LogAudioMixer: FMixerPlatformXAudio2::StopAudioStream() called. InstanceID=70

LogUObjectHash: Compacting FUObjectHashTables data took 1.32ms

LogPlayLevel: Display: Destroying online subsystem :Context_75

LogDebuggerCommands: Repeating last play command: 模拟

LogPlayLevel: PlayLevel: No blueprints needed recompiling

LogPlayLevel: Creating play world package: /Game/UEDPIE_0_shili5

LogPlayLevel: PIE: StaticDuplicateObject took: (0.044538s)

LogPlayLevel: PIE: Created PIE world by copying editor world from /Game/shili5.shili5 to /Game/UEDPIE_0_shili5.shili5 (0.044570s)

LogUObjectHash: Compacting FUObjectHashTables data took 1.20ms

LogWorldMetrics: [UWorldMetricsSubsystem::Initialize]

LogRenderer: SceneCulling instance hierarchy is disabled as UseNanite(PCD3D_SM5) returned false, for scene: 'World /Game/UEDPIE_0_shili5.shili5'.

LogPlayLevel: PIE: World Init took: (0.001130s)

LogAudio: Display: Creating Audio Device: Id: 71, Scope: Unique, Realtime: True

LogAudioMixer: Display: Audio Mixer Platform Settings:

LogAudioMixer: Display: Sample Rate: 48000

LogAudioMixer: Display: Callback Buffer Frame Size Requested: 1024

LogAudioMixer: Display: Callback Buffer Frame Size To Use: 1024

LogAudioMixer: Display: Number of buffers to queue: 2

LogAudioMixer: Display: Max Channels (voices): 32

LogAudioMixer: Display: Number of Async Source Workers: 0

LogAudio: Display: AudioDevice MaxSources: 32

LogAudio: Display: Audio Spatialization Plugin: None (built-in).

LogAudio: Display: Audio Reverb Plugin: None (built-in).

LogAudio: Display: Audio Occlusion Plugin: None (built-in).

LogAudioMixer: Display: Initializing audio mixer using platform API: 'XAudio2'

LogAudioMixer: Display: Using Audio Hardware Device 扬声器 (Realtek(R) Audio)

LogAudioMixer: Display: Initializing Sound Submixes...

LogAudioMixer: Display: Creating Master Submix 'MasterSubmixDefault'

LogAudioMixer: Display: Creating Master Submix 'MasterReverbSubmixDefault'

LogAudioMixer: FMixerPlatformXAudio2::StartAudioStream() called. InstanceID=71

LogAudioMixer: Display: Output buffers initialized: Frames=1024, Channels=2, Samples=2048, InstanceID=71

LogAudioMixer: Display: Starting AudioMixerPlatformInterface::RunInternal(), InstanceID=71

LogAudioMixer: Display: FMixerPlatformXAudio2::SubmitBuffer() called for the first time. InstanceID=71

LogInit: FAudioDevice initialized with ID 71.

LogAudio: Display: Audio Device (ID: 71) registered with world 'shili5'.

LogAudioMixer: Initializing Audio Bus Subsystem for audio device with ID 71

LogLoad: Game class is 'GameModeBase'

LogWorld: Bringing World /Game/UEDPIE_0_shili5.shili5 up for play (max tick rate 60) at 2025.12.10-19.36.01

LogWorld: Bringing up level for play took: 0.023002

LogOnline: OSS: Created online subsystem instance for: :Context_76

LogGameMode: FindPlayerStart: PATHS NOT DEFINED or NO PLAYERSTART with positive rating

PIE: 登陆的服务器

PIE: PIE总开始时间：0.182秒。

LogSlate: Updating window title bar state: overlay mode, drag disabled, window buttons hidden, title bar hidden

LogWorld: BeginTearingDown for /Game/UEDPIE_0_shili5

LogWorld: UWorld::CleanupWorld for shili5, bSessionEnded=true, bCleanupResources=true

LogSlate: InvalidateAllWidgets triggered. All widgets were invalidated

LogWorldMetrics: [UWorldMetricsSubsystem::Deinitialize]

LogWorldMetrics: [UWorldMetricsSubsystem::Clear]

LogPlayLevel: Display: Shutting down PIE online subsystems

LogSlate: InvalidateAllWidgets triggered. All widgets were invalidated

LogSlate: Updating window title bar state: overlay mode, drag disabled, window buttons hidden, title bar hidden

LogAudioMixer: Deinitializing Audio Bus Subsystem for audio device with ID 71

LogAudioMixer: FMixerPlatformXAudio2::StopAudioStream() called. InstanceID=71

LogAudioMixer: FMixerPlatformXAudio2::StopAudioStream() called. InstanceID=71

LogUObjectHash: Compacting FUObjectHashTables data took 1.39ms

LogPlayLevel: Display: Destroying online subsystem :Context_76

LogDebuggerCommands: Repeating last play command: 模拟

LogPlayLevel: PlayLevel: No blueprints needed recompiling

LogPlayLevel: Creating play world package: /Game/UEDPIE_0_shili5

LogPlayLevel: PIE: StaticDuplicateObject took: (0.044149s)

LogPlayLevel: PIE: Created PIE world by copying editor world from /Game/shili5.shili5 to /Game/UEDPIE_0_shili5.shili5 (0.044202s)

LogUObjectHash: Compacting FUObjectHashTables data took 1.24ms

LogWorldMetrics: [UWorldMetricsSubsystem::Initialize]

LogRenderer: SceneCulling instance hierarchy is disabled as UseNanite(PCD3D_SM5) returned false, for scene: 'World /Game/UEDPIE_0_shili5.shili5'.

LogPlayLevel: PIE: World Init took: (0.001036s)

LogAudio: Display: Creating Audio Device: Id: 72, Scope: Unique, Realtime: True

LogAudioMixer: Display: Audio Mixer Platform Settings:

LogAudioMixer: Display: Sample Rate: 48000

LogAudioMixer: Display: Callback Buffer Frame Size Requested: 1024

LogAudioMixer: Display: Callback Buffer Frame Size To Use: 1024

LogAudioMixer: Display: Number of buffers to queue: 2

LogAudioMixer: Display: Max Channels (voices): 32

LogAudioMixer: Display: Number of Async Source Workers: 0

LogAudio: Display: AudioDevice MaxSources: 32

LogAudio: Display: Audio Spatialization Plugin: None (built-in).

LogAudio: Display: Audio Reverb Plugin: None (built-in).

LogAudio: Display: Audio Occlusion Plugin: None (built-in).

LogAudioMixer: Display: Initializing audio mixer using platform API: 'XAudio2'

LogAudioMixer: Display: Using Audio Hardware Device 扬声器 (Realtek(R) Audio)

LogAudioMixer: Display: Initializing Sound Submixes...

LogAudioMixer: Display: Creating Master Submix 'MasterSubmixDefault'

LogAudioMixer: Display: Creating Master Submix 'MasterReverbSubmixDefault'

LogAudioMixer: FMixerPlatformXAudio2::StartAudioStream() called. InstanceID=72

LogAudioMixer: Display: Output buffers initialized: Frames=1024, Channels=2, Samples=2048, InstanceID=72

LogAudioMixer: Display: Starting AudioMixerPlatformInterface::RunInternal(), InstanceID=72

LogAudioMixer: Display: FMixerPlatformXAudio2::SubmitBuffer() called for the first time. InstanceID=72

LogInit: FAudioDevice initialized with ID 72.

LogAudio: Display: Audio Device (ID: 72) registered with world 'shili5'.

LogAudioMixer: Initializing Audio Bus Subsystem for audio device with ID 72

LogLoad: Game class is 'GameModeBase'

LogWorld: Bringing World /Game/UEDPIE_0_shili5.shili5 up for play (max tick rate 60) at 2025.12.10-19.36.51

LogWorld: Bringing up level for play took: 0.021813

LogOnline: OSS: Created online subsystem instance for: :Context_77

LogGameMode: FindPlayerStart: PATHS NOT DEFINED or NO PLAYERSTART with positive rating

PIE: 登陆的服务器

PIE: PIE总开始时间：0.183秒。

LogEOSSDK: LogEOS: Updating Product SDK Config, Time: 3612.278076

LogEOSSDK: LogEOS: SDK Config Product Update Request Completed - No Change

LogEOSSDK: LogEOS: ScheduleNextSDKConfigDataUpdate - Time: 3612.944824, Update Interval: 353.470276

LogSlate: Updating window title bar state: overlay mode, drag disabled, window buttons hidden, title bar hidden

LogWorld: BeginTearingDown for /Game/UEDPIE_0_shili5

LogWorld: UWorld::CleanupWorld for shili5, bSessionEnded=true, bCleanupResources=true

LogSlate: InvalidateAllWidgets triggered. All widgets were invalidated

LogWorldMetrics: [UWorldMetricsSubsystem::Deinitialize]

LogWorldMetrics: [UWorldMetricsSubsystem::Clear]

LogPlayLevel: Display: Shutting down PIE online subsystems

LogSlate: InvalidateAllWidgets triggered. All widgets were invalidated

LogSlate: Updating window title bar state: overlay mode, drag disabled, window buttons hidden, title bar hidden

LogAudioMixer: Deinitializing Audio Bus Subsystem for audio device with ID 72

LogAudioMixer: FMixerPlatformXAudio2::StopAudioStream() called. InstanceID=72

LogAudioMixer: FMixerPlatformXAudio2::StopAudioStream() called. InstanceID=72

LogUObjectHash: Compacting FUObjectHashTables data took 1.38ms

LogPlayLevel: Display: Destroying online subsystem :Context_77

LogDebuggerCommands: Repeating last play command: 模拟

LogPlayLevel: PlayLevel: No blueprints needed recompiling

LogPlayLevel: Creating play world package: /Game/UEDPIE_0_shili5

LogPlayLevel: PIE: StaticDuplicateObject took: (0.043146s)

LogPlayLevel: PIE: Created PIE world by copying editor world from /Game/shili5.shili5 to /Game/UEDPIE_0_shili5.shili5 (0.043178s)

LogUObjectHash: Compacting FUObjectHashTables data took 1.29ms

LogWorldMetrics: [UWorldMetricsSubsystem::Initialize]

LogRenderer: SceneCulling instance hierarchy is disabled as UseNanite(PCD3D_SM5) returned false, for scene: 'World /Game/UEDPIE_0_shili5.shili5'.

LogPlayLevel: PIE: World Init took: (0.001096s)

LogAudio: Display: Creating Audio Device: Id: 73, Scope: Unique, Realtime: True

LogAudioMixer: Display: Audio Mixer Platform Settings:

LogAudioMixer: Display: Sample Rate: 48000

LogAudioMixer: Display: Callback Buffer Frame Size Requested: 1024

LogAudioMixer: Display: Callback Buffer Frame Size To Use: 1024

LogAudioMixer: Display: Number of buffers to queue: 2

LogAudioMixer: Display: Max Channels (voices): 32

LogAudioMixer: Display: Number of Async Source Workers: 0

LogAudio: Display: AudioDevice MaxSources: 32

LogAudio: Display: Audio Spatialization Plugin: None (built-in).

LogAudio: Display: Audio Reverb Plugin: None (built-in).

LogAudio: Display: Audio Occlusion Plugin: None (built-in).

LogAudioMixer: Display: Initializing audio mixer using platform API: 'XAudio2'

LogAudioMixer: Display: Using Audio Hardware Device 扬声器 (Realtek(R) Audio)

LogAudioMixer: Display: Initializing Sound Submixes...

LogAudioMixer: Display: Creating Master Submix 'MasterSubmixDefault'

LogAudioMixer: Display: Creating Master Submix 'MasterReverbSubmixDefault'

LogAudioMixer: FMixerPlatformXAudio2::StartAudioStream() called. InstanceID=73

LogAudioMixer: Display: Output buffers initialized: Frames=1024, Channels=2, Samples=2048, InstanceID=73

LogAudioMixer: Display: Starting AudioMixerPlatformInterface::RunInternal(), InstanceID=73

LogAudioMixer: Display: FMixerPlatformXAudio2::SubmitBuffer() called for the first time. InstanceID=73

LogInit: FAudioDevice initialized with ID 73.

LogAudio: Display: Audio Device (ID: 73) registered with world 'shili5'.

LogAudioMixer: Initializing Audio Bus Subsystem for audio device with ID 73

LogLoad: Game class is 'GameModeBase'

LogWorld: Bringing World /Game/UEDPIE_0_shili5.shili5 up for play (max tick rate 60) at 2025.12.10-19.37.22

LogWorld: Bringing up level for play took: 0.022591

LogOnline: OSS: Created online subsystem instance for: :Context_78

LogGameMode: FindPlayerStart: PATHS NOT DEFINED or NO PLAYERSTART with positive rating

PIE: 登陆的服务器

PIE: PIE总开始时间：0.179秒。

LogSlate: Updating window title bar state: overlay mode, drag disabled, window buttons hidden, title bar hidden

LogWorld: BeginTearingDown for /Game/UEDPIE_0_shili5

LogWorld: UWorld::CleanupWorld for shili5, bSessionEnded=true, bCleanupResources=true

LogSlate: InvalidateAllWidgets triggered. All widgets were invalidated

LogWorldMetrics: [UWorldMetricsSubsystem::Deinitialize]

LogWorldMetrics: [UWorldMetricsSubsystem::Clear]

LogPlayLevel: Display: Shutting down PIE online subsystems

LogSlate: InvalidateAllWidgets triggered. All widgets were invalidated

LogSlate: Updating window title bar state: overlay mode, drag disabled, window buttons hidden, title bar hidden

LogAudioMixer: Deinitializing Audio Bus Subsystem for audio device with ID 73

LogAudioMixer: FMixerPlatformXAudio2::StopAudioStream() called. InstanceID=73

LogAudioMixer: FMixerPlatformXAudio2::StopAudioStream() called. InstanceID=73

LogUObjectHash: Compacting FUObjectHashTables data took 1.40ms

LogPlayLevel: Display: Destroying online subsystem :Context_78

LogDebuggerCommands: Repeating last play command: 模拟

LogPlayLevel: PlayLevel: No blueprints needed recompiling

LogPlayLevel: Creating play world package: /Game/UEDPIE_0_shili5

LogPlayLevel: PIE: StaticDuplicateObject took: (0.043828s)

LogPlayLevel: PIE: Created PIE world by copying editor world from /Game/shili5.shili5 to /Game/UEDPIE_0_shili5.shili5 (0.043859s)

LogUObjectHash: Compacting FUObjectHashTables data took 1.36ms

LogWorldMetrics: [UWorldMetricsSubsystem::Initialize]

LogRenderer: SceneCulling instance hierarchy is disabled as UseNanite(PCD3D_SM5) returned false, for scene: 'World /Game/UEDPIE_0_shili5.shili5'.

LogPlayLevel: PIE: World Init took: (0.001105s)

LogAudio: Display: Creating Audio Device: Id: 74, Scope: Unique, Realtime: True

LogAudioMixer: Display: Audio Mixer Platform Settings:

LogAudioMixer: Display: Sample Rate: 48000

LogAudioMixer: Display: Callback Buffer Frame Size Requested: 1024

LogAudioMixer: Display: Callback Buffer Frame Size To Use: 1024

LogAudioMixer: Display: Number of buffers to queue: 2

LogAudioMixer: Display: Max Channels (voices): 32

LogAudioMixer: Display: Number of Async Source Workers: 0

LogAudio: Display: AudioDevice MaxSources: 32

LogAudio: Display: Audio Spatialization Plugin: None (built-in).

LogAudio: Display: Audio Reverb Plugin: None (built-in).

LogAudio: Display: Audio Occlusion Plugin: None (built-in).

LogAudioMixer: Display: Initializing audio mixer using platform API: 'XAudio2'

LogAudioMixer: Display: Using Audio Hardware Device 扬声器 (Realtek(R) Audio)

LogAudioMixer: Display: Initializing Sound Submixes...

LogAudioMixer: Display: Creating Master Submix 'MasterSubmixDefault'

LogAudioMixer: Display: Creating Master Submix 'MasterReverbSubmixDefault'

LogAudioMixer: FMixerPlatformXAudio2::StartAudioStream() called. InstanceID=74

LogAudioMixer: Display: Output buffers initialized: Frames=1024, Channels=2, Samples=2048, InstanceID=74

LogAudioMixer: Display: Starting AudioMixerPlatformInterface::RunInternal(), InstanceID=74

LogAudioMixer: Display: FMixerPlatformXAudio2::SubmitBuffer() called for the first time. InstanceID=74

LogInit: FAudioDevice initialized with ID 74.

LogAudio: Display: Audio Device (ID: 74) registered with world 'shili5'.

LogAudioMixer: Initializing Audio Bus Subsystem for audio device with ID 74

LogLoad: Game class is 'GameModeBase'

LogWorld: Bringing World /Game/UEDPIE_0_shili5.shili5 up for play (max tick rate 60) at 2025.12.10-19.37.51

LogWorld: Bringing up level for play took: 0.023202

LogOnline: OSS: Created online subsystem instance for: :Context_79

LogGameMode: FindPlayerStart: PATHS NOT DEFINED or NO PLAYERSTART with positive rating

PIE: 登陆的服务器

PIE: PIE总开始时间：0.183秒。

LogSlate: Updating window title bar state: overlay mode, drag disabled, window buttons hidden, title bar hidden

LogWorld: BeginTearingDown for /Game/UEDPIE_0_shili5

LogWorld: UWorld::CleanupWorld for shili5, bSessionEnded=true, bCleanupResources=true

LogSlate: InvalidateAllWidgets triggered. All widgets were invalidated

LogWorldMetrics: [UWorldMetricsSubsystem::Deinitialize]

LogWorldMetrics: [UWorldMetricsSubsystem::Clear]

LogPlayLevel: Display: Shutting down PIE online subsystems

LogSlate: InvalidateAllWidgets triggered. All widgets were invalidated

LogSlate: Updating window title bar state: overlay mode, drag disabled, window buttons hidden, title bar hidden

LogAudioMixer: Deinitializing Audio Bus Subsystem for audio device with ID 74

LogAudioMixer: FMixerPlatformXAudio2::StopAudioStream() called. InstanceID=74

LogAudioMixer: FMixerPlatformXAudio2::StopAudioStream() called. InstanceID=74

LogUObjectHash: Compacting FUObjectHashTables data took 1.37ms

LogPlayLevel: Display: Destroying online subsystem :Context_79

LogDebuggerCommands: Repeating last play command: 模拟

LogPlayLevel: PlayLevel: No blueprints needed recompiling

LogPlayLevel: Creating play world package: /Game/UEDPIE_0_shili5

LogPlayLevel: PIE: StaticDuplicateObject took: (0.051059s)

LogPlayLevel: PIE: Created PIE world by copying editor world from /Game/shili5.shili5 to /Game/UEDPIE_0_shili5.shili5 (0.051115s)

LogUObjectHash: Compacting FUObjectHashTables data took 1.19ms

LogWorldMetrics: [UWorldMetricsSubsystem::Initialize]

LogRenderer: SceneCulling instance hierarchy is disabled as UseNanite(PCD3D_SM5) returned false, for scene: 'World /Game/UEDPIE_0_shili5.shili5'.

LogPlayLevel: PIE: World Init took: (0.001060s)

LogAudio: Display: Creating Audio Device: Id: 75, Scope: Unique, Realtime: True

LogAudioMixer: Display: Audio Mixer Platform Settings:

LogAudioMixer: Display: Sample Rate: 48000

LogAudioMixer: Display: Callback Buffer Frame Size Requested: 1024

LogAudioMixer: Display: Callback Buffer Frame Size To Use: 1024

LogAudioMixer: Display: Number of buffers to queue: 2

LogAudioMixer: Display: Max Channels (voices): 32

LogAudioMixer: Display: Number of Async Source Workers: 0

LogAudio: Display: AudioDevice MaxSources: 32

LogAudio: Display: Audio Spatialization Plugin: None (built-in).

LogAudio: Display: Audio Reverb Plugin: None (built-in).

LogAudio: Display: Audio Occlusion Plugin: None (built-in).

LogAudioMixer: Display: Initializing audio mixer using platform API: 'XAudio2'

LogAudioMixer: Display: Using Audio Hardware Device 扬声器 (Realtek(R) Audio)

LogAudioMixer: Display: Initializing Sound Submixes...

LogAudioMixer: Display: Creating Master Submix 'MasterSubmixDefault'

LogAudioMixer: Display: Creating Master Submix 'MasterReverbSubmixDefault'

LogAudioMixer: FMixerPlatformXAudio2::StartAudioStream() called. InstanceID=75

LogAudioMixer: Display: Output buffers initialized: Frames=1024, Channels=2, Samples=2048, InstanceID=75

LogAudioMixer: Display: Starting AudioMixerPlatformInterface::RunInternal(), InstanceID=75

LogAudioMixer: Display: FMixerPlatformXAudio2::SubmitBuffer() called for the first time. InstanceID=75

LogInit: FAudioDevice initialized with ID 75.

LogAudio: Display: Audio Device (ID: 75) registered with world 'shili5'.

LogAudioMixer: Initializing Audio Bus Subsystem for audio device with ID 75

LogLoad: Game class is 'GameModeBase'

LogWorld: Bringing World /Game/UEDPIE_0_shili5.shili5 up for play (max tick rate 60) at 2025.12.10-19.38.25

LogWorld: Bringing up level for play took: 0.021407

LogOnline: OSS: Created online subsystem instance for: :Context_80

LogGameMode: FindPlayerStart: PATHS NOT DEFINED or NO PLAYERSTART with positive rating

PIE: 登陆的服务器

PIE: PIE总开始时间：0.182秒。

LogDerivedDataCache: C:/Users/若水/AppData/Local/UnrealEngine/Common/DerivedDataCache: Maintenance finished in +00:00:00.007 and deleted 0 files with total size 0 MiB and 29 empty folders. Scanned 0 files in 33 folders with total size 0 MiB.

LogSlate: Updating window title bar state: overlay mode, drag disabled, window buttons hidden, title bar hidden

LogWorld: BeginTearingDown for /Game/UEDPIE_0_shili5

LogWorld: UWorld::CleanupWorld for shili5, bSessionEnded=true, bCleanupResources=true

LogSlate: InvalidateAllWidgets triggered. All widgets were invalidated

LogWorldMetrics: [UWorldMetricsSubsystem::Deinitialize]

LogWorldMetrics: [UWorldMetricsSubsystem::Clear]

LogPlayLevel: Display: Shutting down PIE online subsystems

LogSlate: InvalidateAllWidgets triggered. All widgets were invalidated

LogSlate: Updating window title bar state: overlay mode, drag disabled, window buttons hidden, title bar hidden

LogAudioMixer: Deinitializing Audio Bus Subsystem for audio device with ID 75

LogAudioMixer: FMixerPlatformXAudio2::StopAudioStream() called. InstanceID=75

LogAudioMixer: FMixerPlatformXAudio2::StopAudioStream() called. InstanceID=75

LogUObjectHash: Compacting FUObjectHashTables data took 1.44ms

LogPlayLevel: Display: Destroying online subsystem :Context_80

LogDebuggerCommands: Repeating last play command: 模拟

LogPlayLevel: PlayLevel: No blueprints needed recompiling

LogPlayLevel: Creating play world package: /Game/UEDPIE_0_shili5

LogPlayLevel: PIE: StaticDuplicateObject took: (0.050844s)

LogPlayLevel: PIE: Created PIE world by copying editor world from /Game/shili5.shili5 to /Game/UEDPIE_0_shili5.shili5 (0.050880s)

LogUObjectHash: Compacting FUObjectHashTables data took 1.21ms

LogWorldMetrics: [UWorldMetricsSubsystem::Initialize]

LogRenderer: SceneCulling instance hierarchy is disabled as UseNanite(PCD3D_SM5) returned false, for scene: 'World /Game/UEDPIE_0_shili5.shili5'.

LogPlayLevel: PIE: World Init took: (0.001086s)

LogAudio: Display: Creating Audio Device: Id: 76, Scope: Unique, Realtime: True

LogAudioMixer: Display: Audio Mixer Platform Settings:

LogAudioMixer: Display: Sample Rate: 48000

LogAudioMixer: Display: Callback Buffer Frame Size Requested: 1024

LogAudioMixer: Display: Callback Buffer Frame Size To Use: 1024

LogAudioMixer: Display: Number of buffers to queue: 2

LogAudioMixer: Display: Max Channels (voices): 32

LogAudioMixer: Display: Number of Async Source Workers: 0

LogAudio: Display: AudioDevice MaxSources: 32

LogAudio: Display: Audio Spatialization Plugin: None (built-in).

LogAudio: Display: Audio Reverb Plugin: None (built-in).

LogAudio: Display: Audio Occlusion Plugin: None (built-in).

LogAudioMixer: Display: Initializing audio mixer using platform API: 'XAudio2'

LogAudioMixer: Display: Using Audio Hardware Device 扬声器 (Realtek(R) Audio)

LogAudioMixer: Display: Initializing Sound Submixes...

LogAudioMixer: Display: Creating Master Submix 'MasterSubmixDefault'

LogAudioMixer: Display: Creating Master Submix 'MasterReverbSubmixDefault'

LogAudioMixer: FMixerPlatformXAudio2::StartAudioStream() called. InstanceID=76

LogAudioMixer: Display: Output buffers initialized: Frames=1024, Channels=2, Samples=2048, InstanceID=76

LogAudioMixer: Display: Starting AudioMixerPlatformInterface::RunInternal(), InstanceID=76

LogAudioMixer: Display: FMixerPlatformXAudio2::SubmitBuffer() called for the first time. InstanceID=76

LogInit: FAudioDevice initialized with ID 76.

LogAudio: Display: Audio Device (ID: 76) registered with world 'shili5'.

LogAudioMixer: Initializing Audio Bus Subsystem for audio device with ID 76

LogLoad: Game class is 'GameModeBase'

LogWorld: Bringing World /Game/UEDPIE_0_shili5.shili5 up for play (max tick rate 60) at 2025.12.10-19.40.02

LogWorld: Bringing up level for play took: 0.023787

LogOnline: OSS: Created online subsystem instance for: :Context_81

LogGameMode: FindPlayerStart: PATHS NOT DEFINED or NO PLAYERSTART with positive rating

PIE: 登陆的服务器

PIE: PIE总开始时间：0.196秒。

LogSlate: Updating window title bar state: overlay mode, drag disabled, window buttons hidden, title bar hidden

LogWorld: BeginTearingDown for /Game/UEDPIE_0_shili5

LogWorld: UWorld::CleanupWorld for shili5, bSessionEnded=true, bCleanupResources=true

LogSlate: InvalidateAllWidgets triggered. All widgets were invalidated

LogWorldMetrics: [UWorldMetricsSubsystem::Deinitialize]

LogWorldMetrics: [UWorldMetricsSubsystem::Clear]

LogPlayLevel: Display: Shutting down PIE online subsystems

LogSlate: InvalidateAllWidgets triggered. All widgets were invalidated

LogSlate: Updating window title bar state: overlay mode, drag disabled, window buttons hidden, title bar hidden

LogAudioMixer: Deinitializing Audio Bus Subsystem for audio device with ID 76

LogAudioMixer: FMixerPlatformXAudio2::StopAudioStream() called. InstanceID=76

LogAudioMixer: FMixerPlatformXAudio2::StopAudioStream() called. InstanceID=76

LogUObjectHash: Compacting FUObjectHashTables data took 1.67ms

LogPlayLevel: Display: Destroying online subsystem :Context_81

LogDebuggerCommands: Repeating last play command: 模拟

LogPlayLevel: PlayLevel: No blueprints needed recompiling

LogPlayLevel: Creating play world package: /Game/UEDPIE_0_shili5

LogPlayLevel: PIE: StaticDuplicateObject took: (0.042963s)

LogPlayLevel: PIE: Created PIE world by copying editor world from /Game/shili5.shili5 to /Game/UEDPIE_0_shili5.shili5 (0.042994s)

LogUObjectHash: Compacting FUObjectHashTables data took 1.21ms

LogWorldMetrics: [UWorldMetricsSubsystem::Initialize]

LogRenderer: SceneCulling instance hierarchy is disabled as UseNanite(PCD3D_SM5) returned false, for scene: 'World /Game/UEDPIE_0_shili5.shili5'.

LogPlayLevel: PIE: World Init took: (0.001079s)

LogAudio: Display: Creating Audio Device: Id: 77, Scope: Unique, Realtime: True

LogAudioMixer: Display: Audio Mixer Platform Settings:

LogAudioMixer: Display: Sample Rate: 48000

LogAudioMixer: Display: Callback Buffer Frame Size Requested: 1024

LogAudioMixer: Display: Callback Buffer Frame Size To Use: 1024

LogAudioMixer: Display: Number of buffers to queue: 2

LogAudioMixer: Display: Max Channels (voices): 32

LogAudioMixer: Display: Number of Async Source Workers: 0

LogAudio: Display: AudioDevice MaxSources: 32

LogAudio: Display: Audio Spatialization Plugin: None (built-in).

LogAudio: Display: Audio Reverb Plugin: None (built-in).

LogAudio: Display: Audio Occlusion Plugin: None (built-in).

LogAudioMixer: Display: Initializing audio mixer using platform API: 'XAudio2'

LogAudioMixer: Display: Using Audio Hardware Device 扬声器 (Realtek(R) Audio)

LogAudioMixer: Display: Initializing Sound Submixes...

LogAudioMixer: Display: Creating Master Submix 'MasterSubmixDefault'

LogAudioMixer: Display: Creating Master Submix 'MasterReverbSubmixDefault'

LogAudioMixer: FMixerPlatformXAudio2::StartAudioStream() called. InstanceID=77

LogAudioMixer: Display: Output buffers initialized: Frames=1024, Channels=2, Samples=2048, InstanceID=77

LogAudioMixer: Display: Starting AudioMixerPlatformInterface::RunInternal(), InstanceID=77

LogAudioMixer: Display: FMixerPlatformXAudio2::SubmitBuffer() called for the first time. InstanceID=77

LogInit: FAudioDevice initialized with ID 77.

LogAudio: Display: Audio Device (ID: 77) registered with world 'shili5'.

LogAudioMixer: Initializing Audio Bus Subsystem for audio device with ID 77

LogLoad: Game class is 'GameModeBase'

LogWorld: Bringing World /Game/UEDPIE_0_shili5.shili5 up for play (max tick rate 60) at 2025.12.10-19.40.49

LogWorld: Bringing up level for play took: 0.021863

LogOnline: OSS: Created online subsystem instance for: :Context_82

LogGameMode: FindPlayerStart: PATHS NOT DEFINED or NO PLAYERSTART with positive rating

PIE: 登陆的服务器

PIE: PIE总开始时间：0.175秒。

LogEOSSDK: LogEOS: Updating Product SDK Config, Time: 4003.835938

LogEOSSDK: LogEOS: SDK Config Product Update Request Completed - No Change

LogEOSSDK: LogEOS: ScheduleNextSDKConfigDataUpdate - Time: 4004.503906, Update Interval: 357.125153

[2025.12.10-19.46.31:988][ 70]LogSlate: Updating window title bar state: overlay mode, drag disabled, window buttons hidden, title bar hidden

[2025.12.10-19.46.31:988][ 70]LogWorld: BeginTearingDown for /Game/UEDPIE_0_shili5

[2025.12.10-19.46.31:989][ 70]LogWorld: UWorld::CleanupWorld for shili5, bSessionEnded=true, bCleanupResources=true

[2025.12.10-19.46.31:992][ 70]LogSlate: InvalidateAllWidgets triggered. All widgets were invalidated

[2025.12.10-19.46.31:992][ 70]LogWorldMetrics: [UWorldMetricsSubsystem::Deinitialize]

[2025.12.10-19.46.31:992][ 70]LogWorldMetrics: [UWorldMetricsSubsystem::Clear]

[2025.12.10-19.46.31:995][ 70]LogPlayLevel: Display: Shutting down PIE online subsystems

[2025.12.10-19.46.32:001][ 70]LogSlate: InvalidateAllWidgets triggered. All widgets were invalidated

[2025.12.10-19.46.32:016][ 70]LogSlate: Updating window title bar state: overlay mode, drag disabled, window buttons hidden, title bar hidden

[2025.12.10-19.46.32:018][ 70]LogAudioMixer: Deinitializing Audio Bus Subsystem for audio device with ID 77

[2025.12.10-19.46.32:018][ 70]LogAudioMixer: FMixerPlatformXAudio2::StopAudioStream() called. InstanceID=77

[2025.12.10-19.46.32:020][ 70]LogAudioMixer: FMixerPlatformXAudio2::StopAudioStream() called. InstanceID=77

[2025.12.10-19.46.32:032][ 70]LogUObjectHash: Compacting FUObjectHashTables data took 1.41ms

[2025.12.10-19.46.32:111][ 71]LogPlayLevel: Display: Destroying online subsystem :Context_82

[2025.12.10-19.46.36:230][318]LogDebuggerCommands: Repeating last play command: 模拟

[2025.12.10-19.46.36:241][318]LogPlayLevel: PlayLevel: No blueprints needed recompiling

[2025.12.10-19.46.36:241][318]LogPlayLevel: Creating play world package: /Game/UEDPIE_0_shili5

[2025.12.10-19.46.36:287][318]LogPlayLevel: PIE: StaticDuplicateObject took: (0.045646s)

[2025.12.10-19.46.36:287][318]LogPlayLevel: PIE: Created PIE world by copying editor world from /Game/shili5.shili5 to /Game/UEDPIE_0_shili5.shili5 (0.045680s)

[2025.12.10-19.46.36:306][318]LogUObjectHash: Compacting FUObjectHashTables data took 1.32ms

[2025.12.10-19.46.36:307][318]LogWorldMetrics: [UWorldMetricsSubsystem::Initialize]

[2025.12.10-19.46.36:307][318]LogRenderer: SceneCulling instance hierarchy is disabled as UseNanite(PCD3D_SM5) returned false, for scene: 'World /Game/UEDPIE_0_shili5.shili5'.

[2025.12.10-19.46.36:308][318]LogPlayLevel: PIE: World Init took: (0.001170s)

[2025.12.10-19.46.36:330][318]LogAudio: Display: Creating Audio Device: Id: 78, Scope: Unique, Realtime: True

[2025.12.10-19.46.36:330][318]LogAudioMixer: Display: Audio Mixer Platform Settings:

[2025.12.10-19.46.36:330][318]LogAudioMixer: Display: Sample Rate: 48000

[2025.12.10-19.46.36:330][318]LogAudioMixer: Display: Callback Buffer Frame Size Requested: 1024

[2025.12.10-19.46.36:330][318]LogAudioMixer: Display: Callback Buffer Frame Size To Use: 1024

[2025.12.10-19.46.36:330][318]LogAudioMixer: Display: Number of buffers to queue: 2

[2025.12.10-19.46.36:330][318]LogAudioMixer: Display: Max Channels (voices): 32

[2025.12.10-19.46.36:330][318]LogAudioMixer: Display: Number of Async Source Workers: 0

[2025.12.10-19.46.36:330][318]LogAudio: Display: AudioDevice MaxSources: 32

[2025.12.10-19.46.36:330][318]LogAudio: Display: Audio Spatialization Plugin: None (built-in).

[2025.12.10-19.46.36:330][318]LogAudio: Display: Audio Reverb Plugin: None (built-in).

[2025.12.10-19.46.36:330][318]LogAudio: Display: Audio Occlusion Plugin: None (built-in).

[2025.12.10-19.46.36:332][318]LogAudioMixer: Display: Initializing audio mixer using platform API: 'XAudio2'

[2025.12.10-19.46.36:360][318]LogAudioMixer: Display: Using Audio Hardware Device 扬声器 (Realtek(R) Audio)

[2025.12.10-19.46.36:361][318]LogAudioMixer: Display: Initializing Sound Submixes...

[2025.12.10-19.46.36:361][318]LogAudioMixer: Display: Creating Master Submix 'MasterSubmixDefault'

[2025.12.10-19.46.36:361][318]LogAudioMixer: Display: Creating Master Submix 'MasterReverbSubmixDefault'

[2025.12.10-19.46.36:361][318]LogAudioMixer: FMixerPlatformXAudio2::StartAudioStream() called. InstanceID=78

[2025.12.10-19.46.36:361][318]LogAudioMixer: Display: Output buffers initialized: Frames=1024, Channels=2, Samples=2048, InstanceID=78

[2025.12.10-19.46.36:362][318]LogAudioMixer: Display: Starting AudioMixerPlatformInterface::RunInternal(), InstanceID=78

[2025.12.10-19.46.36:363][318]LogAudioMixer: Display: FMixerPlatformXAudio2::SubmitBuffer() called for the first time. InstanceID=78

[2025.12.10-19.46.36:363][318]LogInit: FAudioDevice initialized with ID 78.

[2025.12.10-19.46.36:363][318]LogAudio: Display: Audio Device (ID: 78) registered with world 'shili5'.

[2025.12.10-19.46.36:363][318]LogAudioMixer: Initializing Audio Bus Subsystem for audio device with ID 78

[2025.12.10-19.46.36:365][318]LogLoad: Game class is 'GameModeBase'

[2025.12.10-19.46.36:387][318]LogWorld: Bringing World /Game/UEDPIE_0_shili5.shili5 up for play (max tick rate 60) at 2025.12.10-19.46.36

[2025.12.10-19.46.36:387][318]LogWorld: Bringing up level for play took: 0.021652

[2025.12.10-19.46.36:389][318]LogOnline: OSS: Created online subsystem instance for: :Context_83

[2025.12.10-19.46.36:389][318]LogGameMode: FindPlayerStart: PATHS NOT DEFINED or NO PLAYERSTART with positive rating

[2025.12.10-19.46.36:415][318]PIE: 登陆的服务器

[2025.12.10-19.46.36:415][318]PIE: PIE总开始时间：0.18秒。

[2025.12.10-19.46.37:751][389]LogSlate: Updating window title bar state: overlay mode, drag disabled, window buttons hidden, title bar hidden

[2025.12.10-19.46.37:751][389]LogWorld: BeginTearingDown for /Game/UEDPIE_0_shili5

[2025.12.10-19.46.37:752][389]LogWorld: UWorld::CleanupWorld for shili5, bSessionEnded=true, bCleanupResources=true

[2025.12.10-19.46.37:756][389]LogSlate: InvalidateAllWidgets triggered. All widgets were invalidated

[2025.12.10-19.46.37:756][389]LogWorldMetrics: [UWorldMetricsSubsystem::Deinitialize]

[2025.12.10-19.46.37:756][389]LogWorldMetrics: [UWorldMetricsSubsystem::Clear]

[2025.12.10-19.46.37:758][389]LogPlayLevel: Display: Shutting down PIE online subsystems

[2025.12.10-19.46.37:764][389]LogSlate: InvalidateAllWidgets triggered. All widgets were invalidated

[2025.12.10-19.46.37:778][389]LogSlate: Updating window title bar state: overlay mode, drag disabled, window buttons hidden, title bar hidden

[2025.12.10-19.46.37:780][389]LogAudioMixer: Deinitializing Audio Bus Subsystem for audio device with ID 78

[2025.12.10-19.46.37:780][389]LogAudioMixer: FMixerPlatformXAudio2::StopAudioStream() called. InstanceID=78

[2025.12.10-19.46.37:782][389]LogAudioMixer: FMixerPlatformXAudio2::StopAudioStream() called. InstanceID=78

[2025.12.10-19.46.37:799][389]LogUObjectHash: Compacting FUObjectHashTables data took 1.39ms

[2025.12.10-19.46.37:877][390]LogPlayLevel: Display: Destroying online subsystem :Context_83

[2025.12.10-19.46.48:628][ 35]LogDebuggerCommands: Repeating last play command: 模拟

[2025.12.10-19.46.48:644][ 35]LogPlayLevel: PlayLevel: No blueprints needed recompiling

[2025.12.10-19.46.48:644][ 35]LogPlayLevel: Creating play world package: /Game/UEDPIE_0_shili5

[2025.12.10-19.46.48:689][ 35]LogPlayLevel: PIE: StaticDuplicateObject took: (0.045506s)

[2025.12.10-19.46.48:689][ 35]LogPlayLevel: PIE: Created PIE world by copying editor world from /Game/shili5.shili5 to /Game/UEDPIE_0_shili5.shili5 (0.045534s)

[2025.12.10-19.46.48:709][ 35]LogUObjectHash: Compacting FUObjectHashTables data took 1.74ms

[2025.12.10-19.46.48:711][ 35]LogWorldMetrics: [UWorldMetricsSubsystem::Initialize]

[2025.12.10-19.46.48:711][ 35]LogRenderer: SceneCulling instance hierarchy is disabled as UseNanite(PCD3D_SM5) returned false, for scene: 'World /Game/UEDPIE_0_shili5.shili5'.

[2025.12.10-19.46.48:712][ 35]LogPlayLevel: PIE: World Init took: (0.001617s)

[2025.12.10-19.46.48:735][ 35]LogAudio: Display: Creating Audio Device: Id: 79, Scope: Unique, Realtime: True

[2025.12.10-19.46.48:735][ 35]LogAudioMixer: Display: Audio Mixer Platform Settings:

[2025.12.10-19.46.48:735][ 35]LogAudioMixer: Display: Sample Rate: 48000

[2025.12.10-19.46.48:735][ 35]LogAudioMixer: Display: Callback Buffer Frame Size Requested: 1024

[2025.12.10-19.46.48:735][ 35]LogAudioMixer: Display: Callback Buffer Frame Size To Use: 1024

[2025.12.10-19.46.48:735][ 35]LogAudioMixer: Display: Number of buffers to queue: 2

[2025.12.10-19.46.48:735][ 35]LogAudioMixer: Display: Max Channels (voices): 32

[2025.12.10-19.46.48:735][ 35]LogAudioMixer: Display: Number of Async Source Workers: 0

[2025.12.10-19.46.48:736][ 35]LogAudio: Display: AudioDevice MaxSources: 32

[2025.12.10-19.46.48:736][ 35]LogAudio: Display: Audio Spatialization Plugin: None (built-in).

[2025.12.10-19.46.48:736][ 35]LogAudio: Display: Audio Reverb Plugin: None (built-in).

[2025.12.10-19.46.48:736][ 35]LogAudio: Display: Audio Occlusion Plugin: None (built-in).

[2025.12.10-19.46.48:737][ 35]LogAudioMixer: Display: Initializing audio mixer using platform API: 'XAudio2'

[2025.12.10-19.46.48:764][ 35]LogAudioMixer: Display: Using Audio Hardware Device 扬声器 (Realtek(R) Audio)

[2025.12.10-19.46.48:764][ 35]LogAudioMixer: Display: Initializing Sound Submixes...

[2025.12.10-19.46.48:764][ 35]LogAudioMixer: Display: Creating Master Submix 'MasterSubmixDefault'

[2025.12.10-19.46.48:764][ 35]LogAudioMixer: Display: Creating Master Submix 'MasterReverbSubmixDefault'

[2025.12.10-19.46.48:764][ 35]LogAudioMixer: FMixerPlatformXAudio2::StartAudioStream() called. InstanceID=79

[2025.12.10-19.46.48:764][ 35]LogAudioMixer: Display: Output buffers initialized: Frames=1024, Channels=2, Samples=2048, InstanceID=79

[2025.12.10-19.46.48:765][ 35]LogAudioMixer: Display: Starting AudioMixerPlatformInterface::RunInternal(), InstanceID=79

[2025.12.10-19.46.48:765][ 35]LogAudioMixer: Display: FMixerPlatformXAudio2::SubmitBuffer() called for the first time. InstanceID=79

[2025.12.10-19.46.48:767][ 35]LogInit: FAudioDevice initialized with ID 79.

[2025.12.10-19.46.48:767][ 35]LogAudio: Display: Audio Device (ID: 79) registered with world 'shili5'.

[2025.12.10-19.46.48:767][ 35]LogAudioMixer: Initializing Audio Bus Subsystem for audio device with ID 79

[2025.12.10-19.46.48:769][ 35]LogLoad: Game class is 'GameModeBase'

[2025.12.10-19.46.48:790][ 35]LogWorld: Bringing World /Game/UEDPIE_0_shili5.shili5 up for play (max tick rate 60) at 2025.12.10-19.46.48

[2025.12.10-19.46.48:790][ 35]LogWorld: Bringing up level for play took: 0.021619

[2025.12.10-19.46.48:792][ 35]LogOnline: OSS: Created online subsystem instance for: :Context_84

[2025.12.10-19.46.48:792][ 35]LogGameMode: FindPlayerStart: PATHS NOT DEFINED or NO PLAYERSTART with positive rating

[2025.12.10-19.46.48:816][ 35]PIE: 登陆的服务器

[2025.12.10-19.46.48:817][ 35]PIE: PIE总开始时间：0.183秒。

[2025.12.10-19.46.49:800][ 87]LogSlate: Updating window title bar state: overlay mode, drag disabled, window buttons hidden, title bar hidden

[2025.12.10-19.46.49:800][ 87]LogWorld: BeginTearingDown for /Game/UEDPIE_0_shili5

[2025.12.10-19.46.49:801][ 87]LogWorld: UWorld::CleanupWorld for shili5, bSessionEnded=true, bCleanupResources=true

[2025.12.10-19.46.49:805][ 87]LogSlate: InvalidateAllWidgets triggered. All widgets were invalidated

[2025.12.10-19.46.49:805][ 87]LogWorldMetrics: [UWorldMetricsSubsystem::Deinitialize]

[2025.12.10-19.46.49:805][ 87]LogWorldMetrics: [UWorldMetricsSubsystem::Clear]

[2025.12.10-19.46.49:807][ 87]LogPlayLevel: Display: Shutting down PIE online subsystems

[2025.12.10-19.46.49:813][ 87]LogSlate: InvalidateAllWidgets triggered. All widgets were invalidated

[2025.12.10-19.46.49:831][ 87]LogSlate: Updating window title bar state: overlay mode, drag disabled, window buttons hidden, title bar hidden

[2025.12.10-19.46.49:833][ 87]LogAudioMixer: Deinitializing Audio Bus Subsystem for audio device with ID 79

[2025.12.10-19.46.49:833][ 87]LogAudioMixer: FMixerPlatformXAudio2::StopAudioStream() called. InstanceID=79

[2025.12.10-19.46.49:834][ 87]LogAudioMixer: FMixerPlatformXAudio2::StopAudioStream() called. InstanceID=79

[2025.12.10-19.46.49:847][ 87]LogUObjectHash: Compacting FUObjectHashTables data took 1.34ms

[2025.12.10-19.46.49:927][ 88]LogPlayLevel: Display: Destroying online subsystem :Context_84

[2025.12.10-19.46.51:594][188]LogDebuggerCommands: Repeating last play command: 模拟

[2025.12.10-19.46.51:608][188]LogPlayLevel: PlayLevel: No blueprints needed recompiling

[2025.12.10-19.46.51:608][188]LogPlayLevel: Creating play world package: /Game/UEDPIE_0_shili5

[2025.12.10-19.46.51:653][188]LogPlayLevel: PIE: StaticDuplicateObject took: (0.044927s)

[2025.12.10-19.46.51:653][188]LogPlayLevel: PIE: Created PIE world by copying editor world from /Game/shili5.shili5 to /Game/UEDPIE_0_shili5.shili5 (0.044959s)

[2025.12.10-19.46.51:673][188]LogUObjectHash: Compacting FUObjectHashTables data took 1.31ms

[2025.12.10-19.46.51:674][188]LogWorldMetrics: [UWorldMetricsSubsystem::Initialize]

[2025.12.10-19.46.51:674][188]LogRenderer: SceneCulling instance hierarchy is disabled as UseNanite(PCD3D_SM5) returned false, for scene: 'World /Game/UEDPIE_0_shili5.shili5'.

[2025.12.10-19.46.51:675][188]LogPlayLevel: PIE: World Init took: (0.001114s)

[2025.12.10-19.46.51:698][188]LogAudio: Display: Creating Audio Device: Id: 80, Scope: Unique, Realtime: True

[2025.12.10-19.46.51:698][188]LogAudioMixer: Display: Audio Mixer Platform Settings:

[2025.12.10-19.46.51:698][188]LogAudioMixer: Display: Sample Rate: 48000

[2025.12.10-19.46.51:698][188]LogAudioMixer: Display: Callback Buffer Frame Size Requested: 1024

[2025.12.10-19.46.51:698][188]LogAudioMixer: Display: Callback Buffer Frame Size To Use: 1024

[2025.12.10-19.46.51:698][188]LogAudioMixer: Display: Number of buffers to queue: 2

[2025.12.10-19.46.51:698][188]LogAudioMixer: Display: Max Channels (voices): 32

[2025.12.10-19.46.51:698][188]LogAudioMixer: Display: Number of Async Source Workers: 0

[2025.12.10-19.46.51:698][188]LogAudio: Display: AudioDevice MaxSources: 32

[2025.12.10-19.46.51:698][188]LogAudio: Display: Audio Spatialization Plugin: None (built-in).

[2025.12.10-19.46.51:698][188]LogAudio: Display: Audio Reverb Plugin: None (built-in).

[2025.12.10-19.46.51:698][188]LogAudio: Display: Audio Occlusion Plugin: None (built-in).

[2025.12.10-19.46.51:700][188]LogAudioMixer: Display: Initializing audio mixer using platform API: 'XAudio2'

[2025.12.10-19.46.51:726][188]LogAudioMixer: Display: Using Audio Hardware Device 扬声器 (Realtek(R) Audio)

[2025.12.10-19.46.51:726][188]LogAudioMixer: Display: Initializing Sound Submixes...

[2025.12.10-19.46.51:726][188]LogAudioMixer: Display: Creating Master Submix 'MasterSubmixDefault'

[2025.12.10-19.46.51:726][188]LogAudioMixer: Display: Creating Master Submix 'MasterReverbSubmixDefault'

[2025.12.10-19.46.51:726][188]LogAudioMixer: FMixerPlatformXAudio2::StartAudioStream() called. InstanceID=80

[2025.12.10-19.46.51:726][188]LogAudioMixer: Display: Output buffers initialized: Frames=1024, Channels=2, Samples=2048, InstanceID=80

[2025.12.10-19.46.51:729][188]LogAudioMixer: Display: Starting AudioMixerPlatformInterface::RunInternal(), InstanceID=80

[2025.12.10-19.46.51:729][188]LogAudioMixer: Display: FMixerPlatformXAudio2::SubmitBuffer() called for the first time. InstanceID=80

[2025.12.10-19.46.51:729][188]LogInit: FAudioDevice initialized with ID 80.

[2025.12.10-19.46.51:729][188]LogAudio: Display: Audio Device (ID: 80) registered with world 'shili5'.

[2025.12.10-19.46.51:729][188]LogAudioMixer: Initializing Audio Bus Subsystem for audio device with ID 80

[2025.12.10-19.46.51:731][188]LogLoad: Game class is 'GameModeBase'

[2025.12.10-19.46.51:753][188]LogWorld: Bringing World /Game/UEDPIE_0_shili5.shili5 up for play (max tick rate 60) at 2025.12.10-19.46.51

[2025.12.10-19.46.51:753][188]LogWorld: Bringing up level for play took: 0.022861

[2025.12.10-19.46.51:756][188]LogOnline: OSS: Created online subsystem instance for: :Context_85

[2025.12.10-19.46.51:756][188]LogGameMode: FindPlayerStart: PATHS NOT DEFINED or NO PLAYERSTART with positive rating

[2025.12.10-19.46.51:779][188]PIE: 登陆的服务器

[2025.12.10-19.46.51:780][188]PIE: PIE总开始时间：0.18秒。

[2025.12.10-19.46.53:060][256]LogSlate: Updating window title bar state: overlay mode, drag disabled, window buttons hidden, title bar hidden

[2025.12.10-19.46.53:060][256]LogWorld: BeginTearingDown for /Game/UEDPIE_0_shili5

[2025.12.10-19.46.53:061][256]LogWorld: UWorld::CleanupWorld for shili5, bSessionEnded=true, bCleanupResources=true

[2025.12.10-19.46.53:065][256]LogSlate: InvalidateAllWidgets triggered. All widgets were invalidated

[2025.12.10-19.46.53:065][256]LogWorldMetrics: [UWorldMetricsSubsystem::Deinitialize]

[2025.12.10-19.46.53:065][256]LogWorldMetrics: [UWorldMetricsSubsystem::Clear]

[2025.12.10-19.46.53:067][256]LogPlayLevel: Display: Shutting down PIE online subsystems

[2025.12.10-19.46.53:072][256]LogSlate: InvalidateAllWidgets triggered. All widgets were invalidated

[2025.12.10-19.46.53:088][256]LogSlate: Updating window title bar state: overlay mode, drag disabled, window buttons hidden, title bar hidden

[2025.12.10-19.46.53:091][256]LogAudioMixer: Deinitializing Audio Bus Subsystem for audio device with ID 80

[2025.12.10-19.46.53:091][256]LogAudioMixer: FMixerPlatformXAudio2::StopAudioStream() called. InstanceID=80

[2025.12.10-19.46.53:093][256]LogAudioMixer: FMixerPlatformXAudio2::StopAudioStream() called. InstanceID=80

[2025.12.10-19.46.53:110][256]LogUObjectHash: Compacting FUObjectHashTables data took 1.46ms

[2025.12.10-19.46.53:196][257]LogPlayLevel: Display: Destroying online subsystem :Context_85

[2025.12.10-19.46.54:564][339]LogDebuggerCommands: Repeating last play command: 模拟

[2025.12.10-19.46.54:575][339]LogPlayLevel: PlayLevel: No blueprints needed recompiling

[2025.12.10-19.46.54:575][339]LogPlayLevel: Creating play world package: /Game/UEDPIE_0_shili5

[2025.12.10-19.46.54:627][339]LogPlayLevel: PIE: StaticDuplicateObject took: (0.051387s)

[2025.12.10-19.46.54:627][339]LogPlayLevel: PIE: Created PIE world by copying editor world from /Game/shili5.shili5 to /Game/UEDPIE_0_shili5.shili5 (0.051421s)

[2025.12.10-19.46.54:648][339]LogUObjectHash: Compacting FUObjectHashTables data took 1.37ms

[2025.12.10-19.46.54:649][339]LogWorldMetrics: [UWorldMetricsSubsystem::Initialize]

[2025.12.10-19.46.54:649][339]LogRenderer: SceneCulling instance hierarchy is disabled as UseNanite(PCD3D_SM5) returned false, for scene: 'World /Game/UEDPIE_0_shili5.shili5'.

[2025.12.10-19.46.54:650][339]LogPlayLevel: PIE: World Init took: (0.001186s)

[2025.12.10-19.46.54:672][339]LogAudio: Display: Creating Audio Device: Id: 81, Scope: Unique, Realtime: True

[2025.12.10-19.46.54:672][339]LogAudioMixer: Display: Audio Mixer Platform Settings:

[2025.12.10-19.46.54:672][339]LogAudioMixer: Display: Sample Rate: 48000

[2025.12.10-19.46.54:672][339]LogAudioMixer: Display: Callback Buffer Frame Size Requested: 1024

[2025.12.10-19.46.54:672][339]LogAudioMixer: Display: Callback Buffer Frame Size To Use: 1024

[2025.12.10-19.46.54:672][339]LogAudioMixer: Display: Number of buffers to queue: 2

[2025.12.10-19.46.54:672][339]LogAudioMixer: Display: Max Channels (voices): 32

[2025.12.10-19.46.54:672][339]LogAudioMixer: Display: Number of Async Source Workers: 0

[2025.12.10-19.46.54:672][339]LogAudio: Display: AudioDevice MaxSources: 32

[2025.12.10-19.46.54:672][339]LogAudio: Display: Audio Spatialization Plugin: None (built-in).

[2025.12.10-19.46.54:672][339]LogAudio: Display: Audio Reverb Plugin: None (built-in).

[2025.12.10-19.46.54:672][339]LogAudio: Display: Audio Occlusion Plugin: None (built-in).

[2025.12.10-19.46.54:674][339]LogAudioMixer: Display: Initializing audio mixer using platform API: 'XAudio2'

[2025.12.10-19.46.54:699][339]LogAudioMixer: Display: Using Audio Hardware Device 扬声器 (Realtek(R) Audio)

[2025.12.10-19.46.54:699][339]LogAudioMixer: Display: Initializing Sound Submixes...

[2025.12.10-19.46.54:699][339]LogAudioMixer: Display: Creating Master Submix 'MasterSubmixDefault'

[2025.12.10-19.46.54:699][339]LogAudioMixer: Display: Creating Master Submix 'MasterReverbSubmixDefault'

[2025.12.10-19.46.54:699][339]LogAudioMixer: FMixerPlatformXAudio2::StartAudioStream() called. InstanceID=81

[2025.12.10-19.46.54:699][339]LogAudioMixer: Display: Output buffers initialized: Frames=1024, Channels=2, Samples=2048, InstanceID=81

[2025.12.10-19.46.54:701][339]LogAudioMixer: Display: Starting AudioMixerPlatformInterface::RunInternal(), InstanceID=81

[2025.12.10-19.46.54:701][339]LogAudioMixer: Display: FMixerPlatformXAudio2::SubmitBuffer() called for the first time. InstanceID=81

[2025.12.10-19.46.54:701][339]LogInit: FAudioDevice initialized with ID 81.

[2025.12.10-19.46.54:701][339]LogAudio: Display: Audio Device (ID: 81) registered with world 'shili5'.

[2025.12.10-19.46.54:701][339]LogAudioMixer: Initializing Audio Bus Subsystem for audio device with ID 81

[2025.12.10-19.46.54:704][339]LogLoad: Game class is 'GameModeBase'

[2025.12.10-19.46.54:724][339]LogWorld: Bringing World /Game/UEDPIE_0_shili5.shili5 up for play (max tick rate 60) at 2025.12.10-19.46.54

[2025.12.10-19.46.54:725][339]LogWorld: Bringing up level for play took: 0.021102

[2025.12.10-19.46.54:727][339]LogOnline: OSS: Created online subsystem instance for: :Context_86

[2025.12.10-19.46.54:727][339]LogGameMode: FindPlayerStart: PATHS NOT DEFINED or NO PLAYERSTART with positive rating

[2025.12.10-19.46.54:750][339]PIE: 登陆的服务器

[2025.12.10-19.46.54:750][339]PIE: PIE总开始时间：0.18秒。

[2025.12.10-19.47.13:632][739]LogSlate: Updating window title bar state: overlay mode, drag disabled, window buttons hidden, title bar hidden

[2025.12.10-19.47.13:632][739]LogWorld: BeginTearingDown for /Game/UEDPIE_0_shili5

[2025.12.10-19.47.13:633][739]LogWorld: UWorld::CleanupWorld for shili5, bSessionEnded=true, bCleanupResources=true

[2025.12.10-19.47.13:637][739]LogSlate: InvalidateAllWidgets triggered. All widgets were invalidated

[2025.12.10-19.47.13:638][739]LogWorldMetrics: [UWorldMetricsSubsystem::Deinitialize]

[2025.12.10-19.47.13:638][739]LogWorldMetrics: [UWorldMetricsSubsystem::Clear]

[2025.12.10-19.47.13:641][739]LogPlayLevel: Display: Shutting down PIE online subsystems

[2025.12.10-19.47.13:647][739]LogSlate: InvalidateAllWidgets triggered. All widgets were invalidated

[2025.12.10-19.47.13:662][739]LogSlate: Updating window title bar state: overlay mode, drag disabled, window buttons hidden, title bar hidden

[2025.12.10-19.47.13:663][739]LogAudioMixer: Deinitializing Audio Bus Subsystem for audio device with ID 81

[2025.12.10-19.47.13:663][739]LogAudioMixer: FMixerPlatformXAudio2::StopAudioStream() called. InstanceID=81

[2025.12.10-19.47.13:665][739]LogAudioMixer: FMixerPlatformXAudio2::StopAudioStream() called. InstanceID=81

[2025.12.10-19.47.13:677][739]LogUObjectHash: Compacting FUObjectHashTables data took 1.29ms

[2025.12.10-19.47.13:757][740]LogPlayLevel: Display: Destroying online subsystem :Context_86

[2025.12.10-19.47.50:650][338]LogDebuggerCommands: Repeating last play command: 模拟

[2025.12.10-19.47.50:662][338]LogPlayLevel: PlayLevel: No blueprints needed recompiling

[2025.12.10-19.47.50:662][338]LogPlayLevel: Creating play world package: /Game/UEDPIE_0_shili5

[2025.12.10-19.47.50:706][338]LogPlayLevel: PIE: StaticDuplicateObject took: (0.043456s)

[2025.12.10-19.47.50:706][338]LogPlayLevel: PIE: Created PIE world by copying editor world from /Game/shili5.shili5 to /Game/UEDPIE_0_shili5.shili5 (0.043491s)

[2025.12.10-19.47.50:721][338]LogUObjectHash: Compacting FUObjectHashTables data took 1.23ms

[2025.12.10-19.47.50:722][338]LogWorldMetrics: [UWorldMetricsSubsystem::Initialize]

[2025.12.10-19.47.50:723][338]LogRenderer: SceneCulling instance hierarchy is disabled as UseNanite(PCD3D_SM5) returned false, for scene: 'World /Game/UEDPIE_0_shili5.shili5'.

[2025.12.10-19.47.50:723][338]LogPlayLevel: PIE: World Init took: (0.001119s)

[2025.12.10-19.47.50:747][338]LogAudio: Display: Creating Audio Device: Id: 82, Scope: Unique, Realtime: True

[2025.12.10-19.47.50:747][338]LogAudioMixer: Display: Audio Mixer Platform Settings:

[2025.12.10-19.47.50:747][338]LogAudioMixer: Display: Sample Rate: 48000

[2025.12.10-19.47.50:747][338]LogAudioMixer: Display: Callback Buffer Frame Size Requested: 1024

[2025.12.10-19.47.50:747][338]LogAudioMixer: Display: Callback Buffer Frame Size To Use: 1024

[2025.12.10-19.47.50:747][338]LogAudioMixer: Display: Number of buffers to queue: 2

[2025.12.10-19.47.50:747][338]LogAudioMixer: Display: Max Channels (voices): 32

[2025.12.10-19.47.50:747][338]LogAudioMixer: Display: Number of Async Source Workers: 0

[2025.12.10-19.47.50:747][338]LogAudio: Display: AudioDevice MaxSources: 32

[2025.12.10-19.47.50:747][338]LogAudio: Display: Audio Spatialization Plugin: None (built-in).

[2025.12.10-19.47.50:747][338]LogAudio: Display: Audio Reverb Plugin: None (built-in).

[2025.12.10-19.47.50:747][338]LogAudio: Display: Audio Occlusion Plugin: None (built-in).

[2025.12.10-19.47.50:749][338]LogAudioMixer: Display: Initializing audio mixer using platform API: 'XAudio2'

[2025.12.10-19.47.50:773][338]LogAudioMixer: Display: Using Audio Hardware Device 扬声器 (Realtek(R) Audio)

[2025.12.10-19.47.50:774][338]LogAudioMixer: Display: Initializing Sound Submixes...

[2025.12.10-19.47.50:774][338]LogAudioMixer: Display: Creating Master Submix 'MasterSubmixDefault'

[2025.12.10-19.47.50:774][338]LogAudioMixer: Display: Creating Master Submix 'MasterReverbSubmixDefault'

[2025.12.10-19.47.50:774][338]LogAudioMixer: FMixerPlatformXAudio2::StartAudioStream() called. InstanceID=82

[2025.12.10-19.47.50:774][338]LogAudioMixer: Display: Output buffers initialized: Frames=1024, Channels=2, Samples=2048, InstanceID=82

[2025.12.10-19.47.50:776][338]LogAudioMixer: Display: Starting AudioMixerPlatformInterface::RunInternal(), InstanceID=82

[2025.12.10-19.47.50:776][338]LogAudioMixer: Display: FMixerPlatformXAudio2::SubmitBuffer() called for the first time. InstanceID=82

[2025.12.10-19.47.50:776][338]LogInit: FAudioDevice initialized with ID 82.

[2025.12.10-19.47.50:776][338]LogAudio: Display: Audio Device (ID: 82) registered with world 'shili5'.

[2025.12.10-19.47.50:776][338]LogAudioMixer: Initializing Audio Bus Subsystem for audio device with ID 82

[2025.12.10-19.47.50:778][338]LogLoad: Game class is 'GameModeBase'

[2025.12.10-19.47.50:800][338]LogWorld: Bringing World /Game/UEDPIE_0_shili5.shili5 up for play (max tick rate 60) at 2025.12.10-19.47.50

[2025.12.10-19.47.50:801][338]LogWorld: Bringing up level for play took: 0.021638

[2025.12.10-19.47.50:802][338]LogOnline: OSS: Created online subsystem instance for: :Context_87

[2025.12.10-19.47.50:803][338]LogGameMode: FindPlayerStart: PATHS NOT DEFINED or NO PLAYERSTART with positive rating

[2025.12.10-19.47.50:832][338]PIE: 登陆的服务器

[2025.12.10-19.47.50:832][338]PIE: PIE总开始时间：0.177秒。

[2025.12.10-19.50.10:333][100]LogEOSSDK: LogEOS: Updating Product SDK Config, Time: 4395.936035

[2025.12.10-19.50.10:648][119]LogEOSSDK: LogEOS: SDK Config Product Update Request Completed - No Change

[2025.12.10-19.50.10:648][119]LogEOSSDK: LogEOS: ScheduleNextSDKConfigDataUpdate - Time: 4396.234863, Update Interval: 318.190247

[2025.12.10-19.56.20:290][282]LogEOSSDK: LogEOS: Updating Product SDK Config, Time: 4765.794434

[2025.12.10-19.56.20:657][304]LogEOSSDK: LogEOS: SDK Config Product Update Request Completed - No Change

[2025.12.10-19.56.20:658][304]LogEOSSDK: LogEOS: ScheduleNextSDKConfigDataUpdate - Time: 4766.145508, Update Interval: 357.883240

[2025.12.10-20.02.54:675][941]LogEOSSDK: LogEOS: Updating Product SDK Config, Time: 5160.056641

[2025.12.10-20.02.56:575][ 54]LogEOSSDK: LogEOS: SDK Config Product Update Request Completed - No Change

[2025.12.10-20.02.56:575][ 54]LogEOSSDK: LogEOS: ScheduleNextSDKConfigDataUpdate - Time: 5161.941895, Update Interval: 356.085083

[2025.12.10-20.09.35:357][972]LogEOSSDK: LogEOS: Updating Product SDK Config, Time: 5560.604980

[2025.12.10-20.09.35:740][995]LogEOSSDK: LogEOS: SDK Config Product Update Request Completed - No Change

[2025.12.10-20.09.35:740][995]LogEOSSDK: LogEOS: ScheduleNextSDKConfigDataUpdate - Time: 5560.972168, Update Interval: 325.490906
