# Supplementary figures and images for: Digital Twin Modeling for Landslide Risk Scenarios in Mountainous Regions
Source: Sensors (Basel). 2026 Jan 8;26(2):421. doi: 10.3390/s26020421 (PMC12845812; doi:10.3390/s26020421)

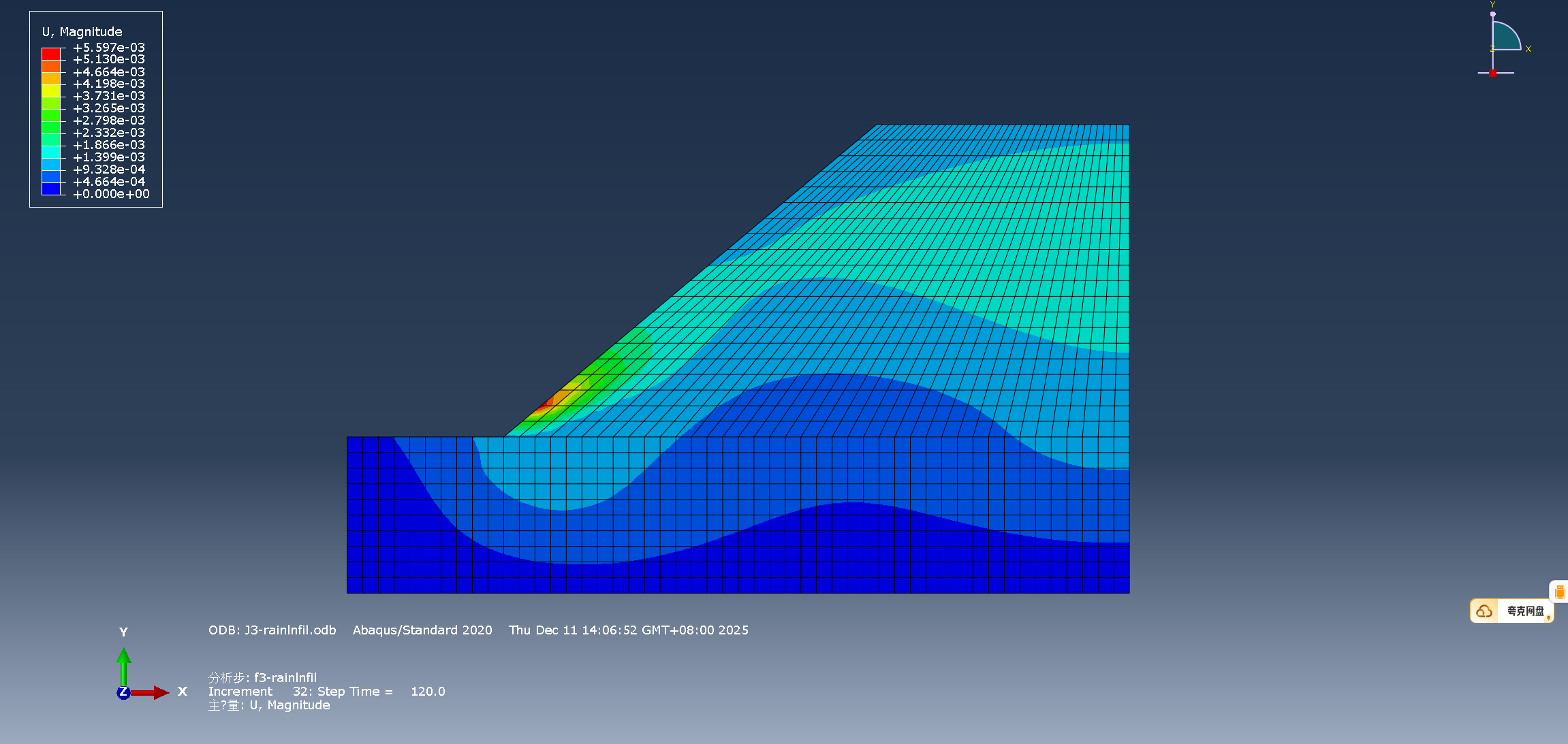

Supplement: Supplementary file 1 [file sensors-26-00421-s001.zip › Supplementary Materials/Materials in Sensitivity Analysis/Experiment on Poisson's Ratio/0.35Poisson's Ratio40°.png]

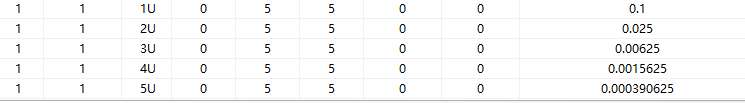

Supplement: Supplementary file 1 [file sensors-26-00421-s001.zip › Supplementary Materials/Materials in Sensitivity Analysis/Experiment on Poisson's Ratio/0.35Poisson's Ratio80°.jpg]

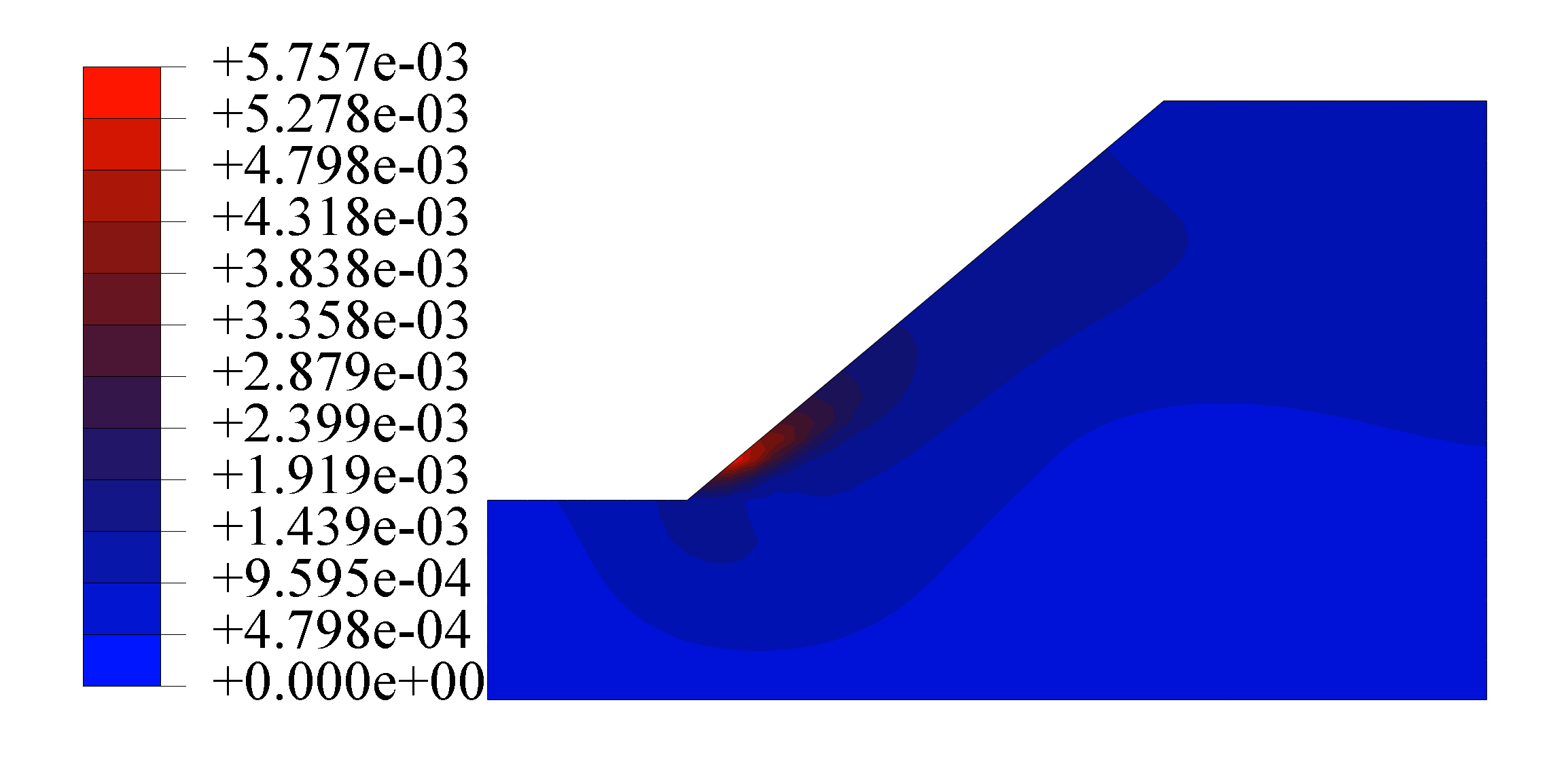

Supplement: Supplementary file 1 [file sensors-26-00421-s001.zip › Supplementary Materials/Materials in Sensitivity Analysis/Experiment on Poisson's Ratio/0.3Poisson's Ratio40°.png]

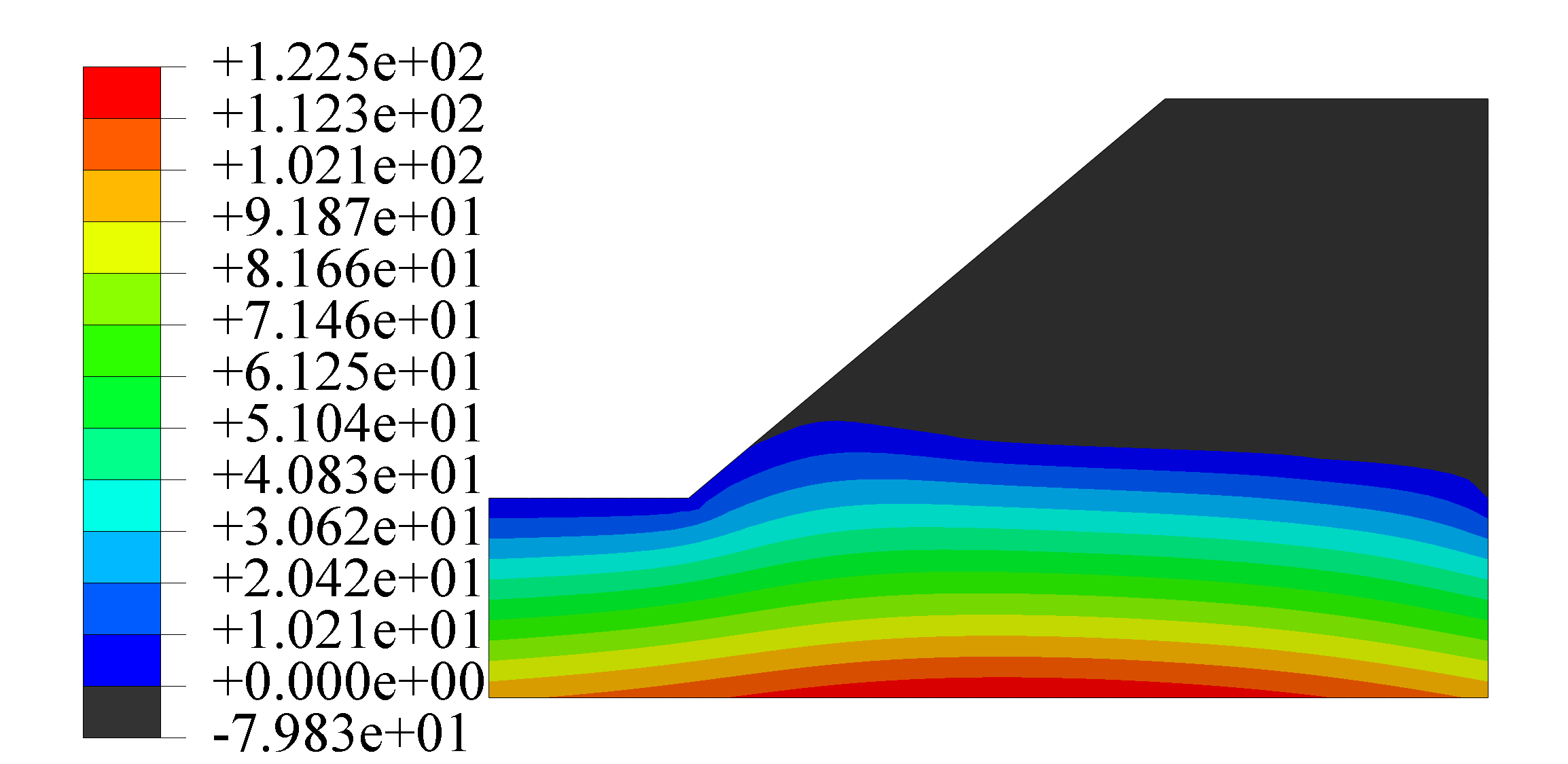

Supplement: Supplementary file 1 [file sensors-26-00421-s001.zip › Supplementary Materials/por40.png]

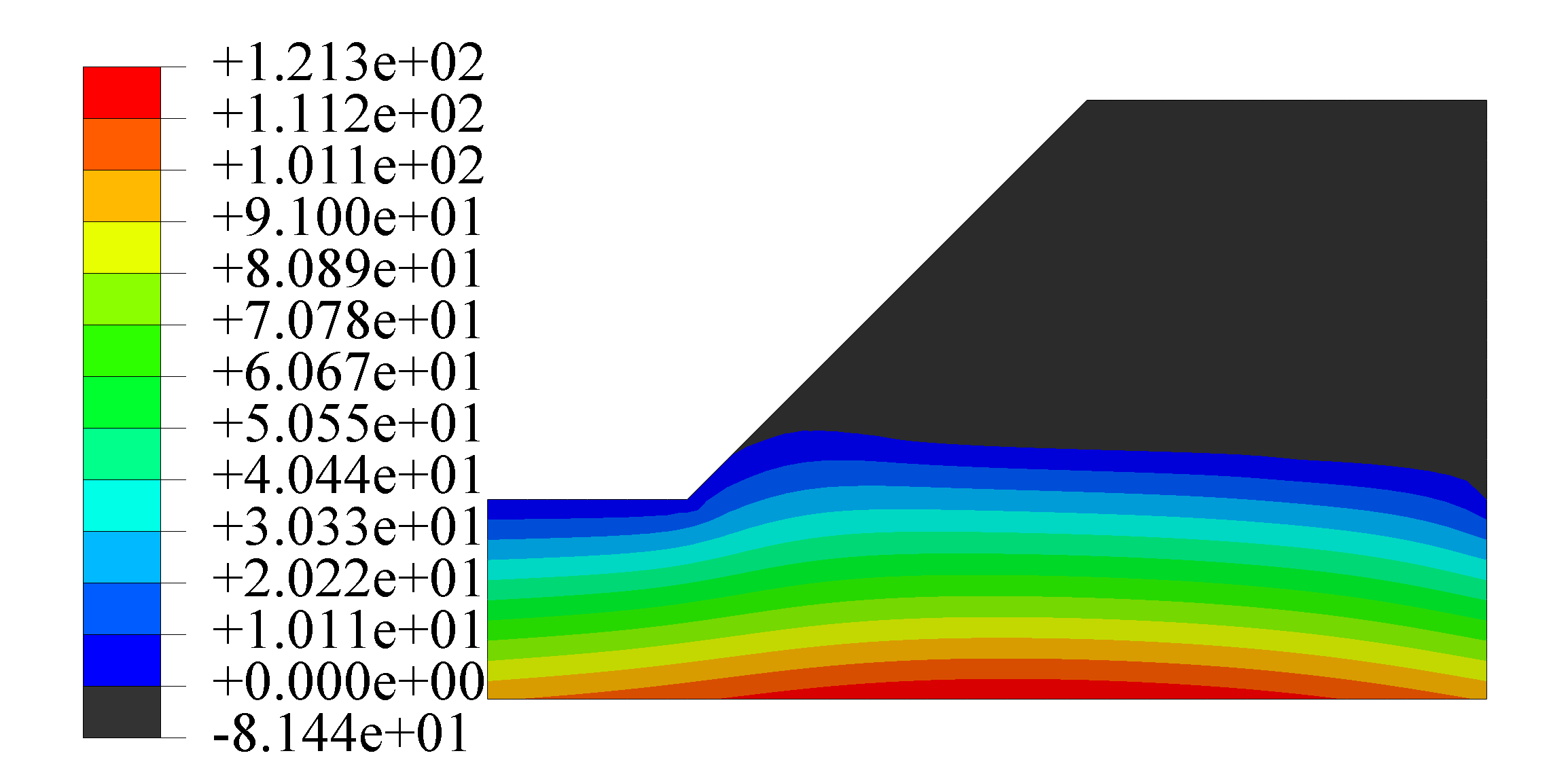

Supplement: Supplementary file 1 [file sensors-26-00421-s001.zip › Supplementary Materials/por45.png]

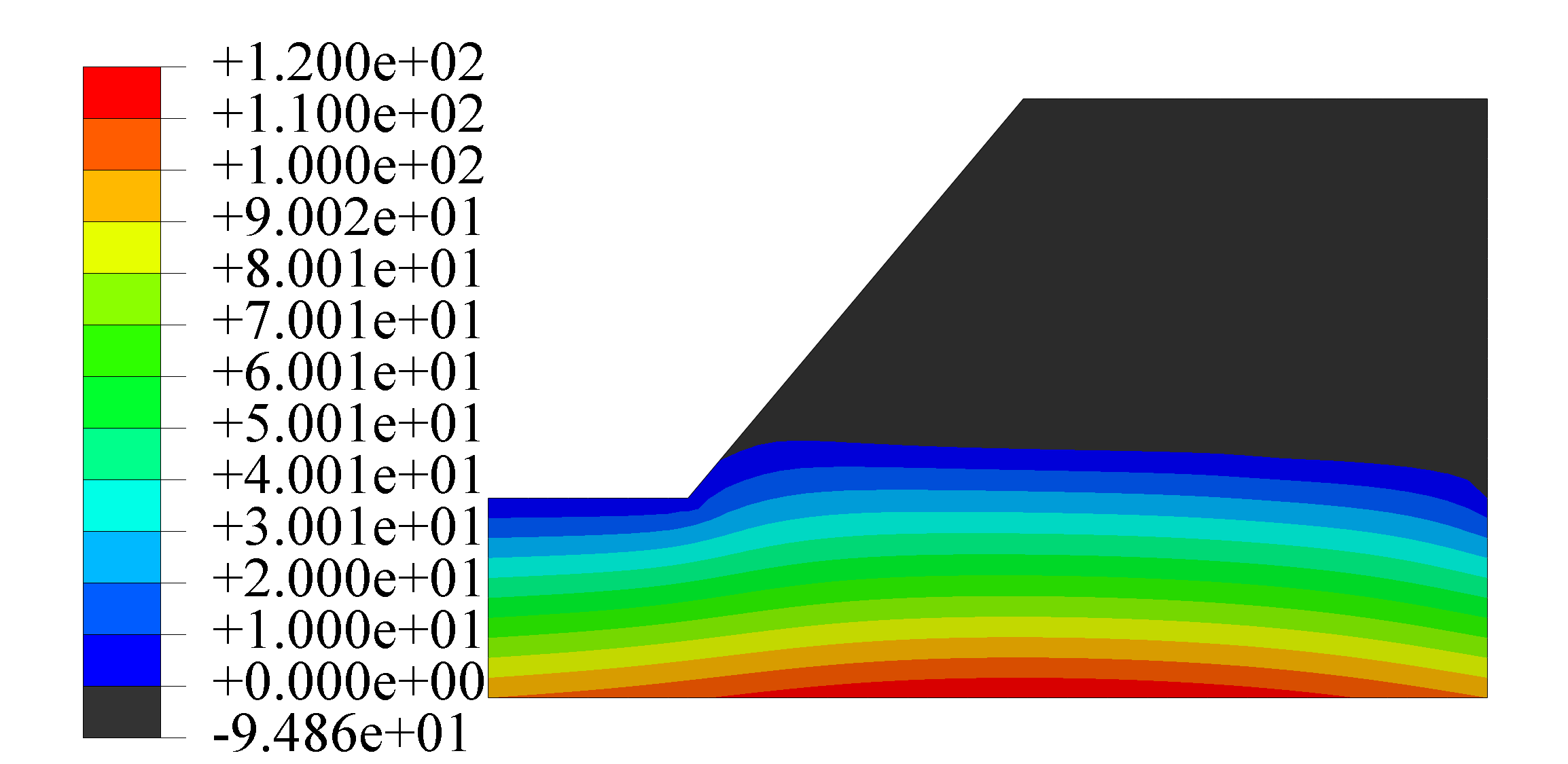

Supplement: Supplementary file 1 [file sensors-26-00421-s001.zip › Supplementary Materials/por50.png]

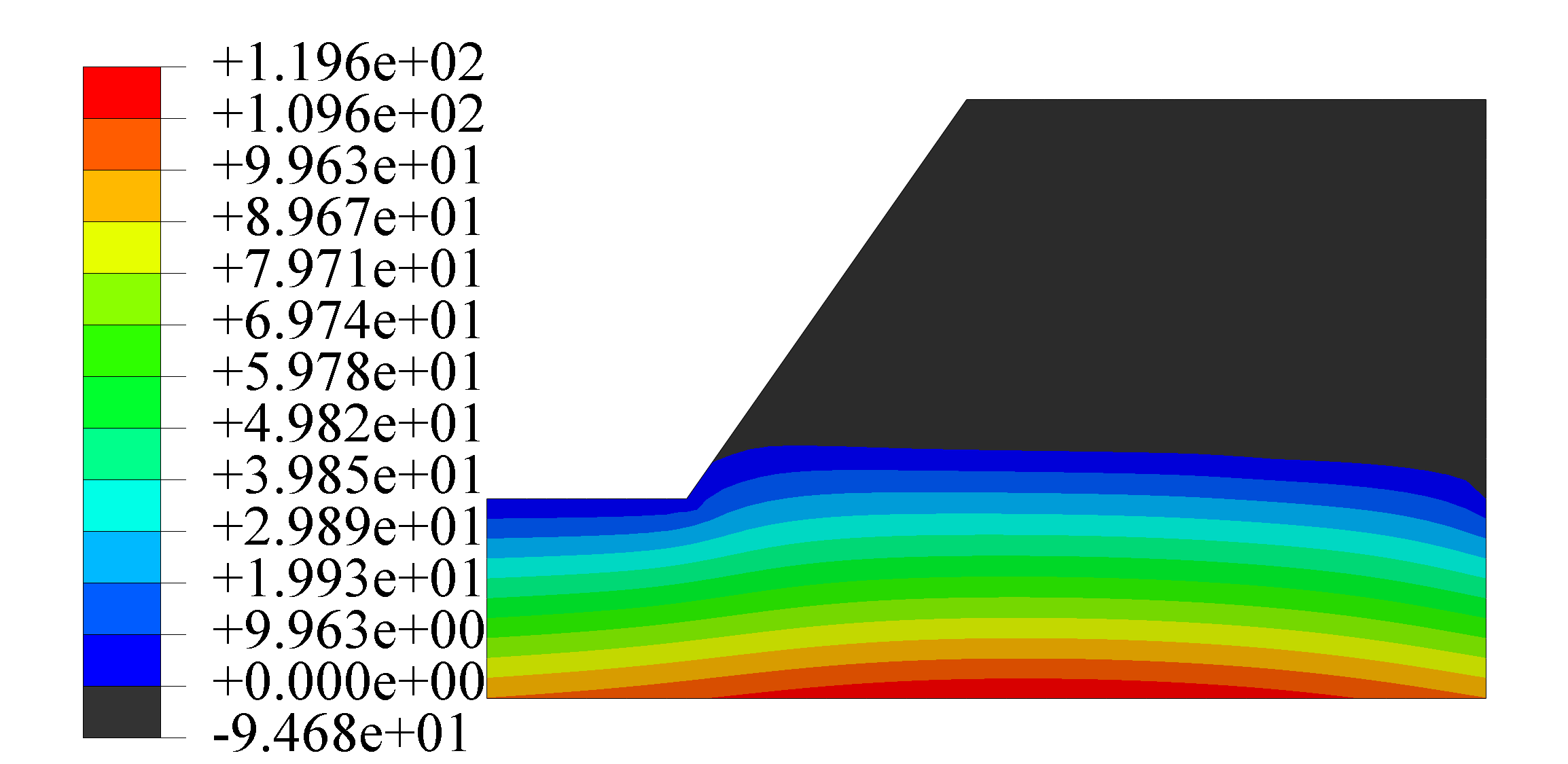

Supplement: Supplementary file 1 [file sensors-26-00421-s001.zip › Supplementary Materials/por55.png]

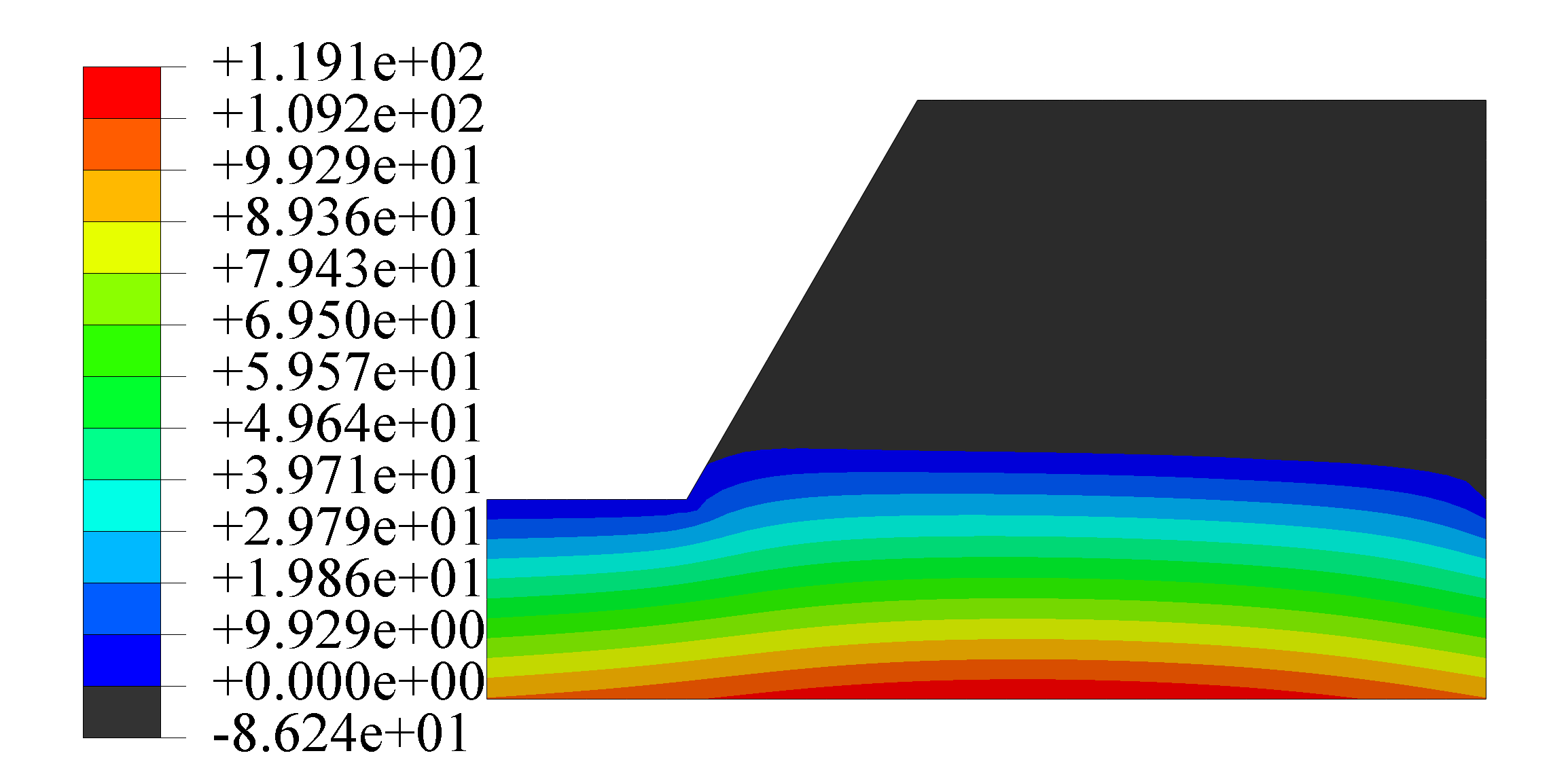

Supplement: Supplementary file 1 [file sensors-26-00421-s001.zip › Supplementary Materials/por60.png]

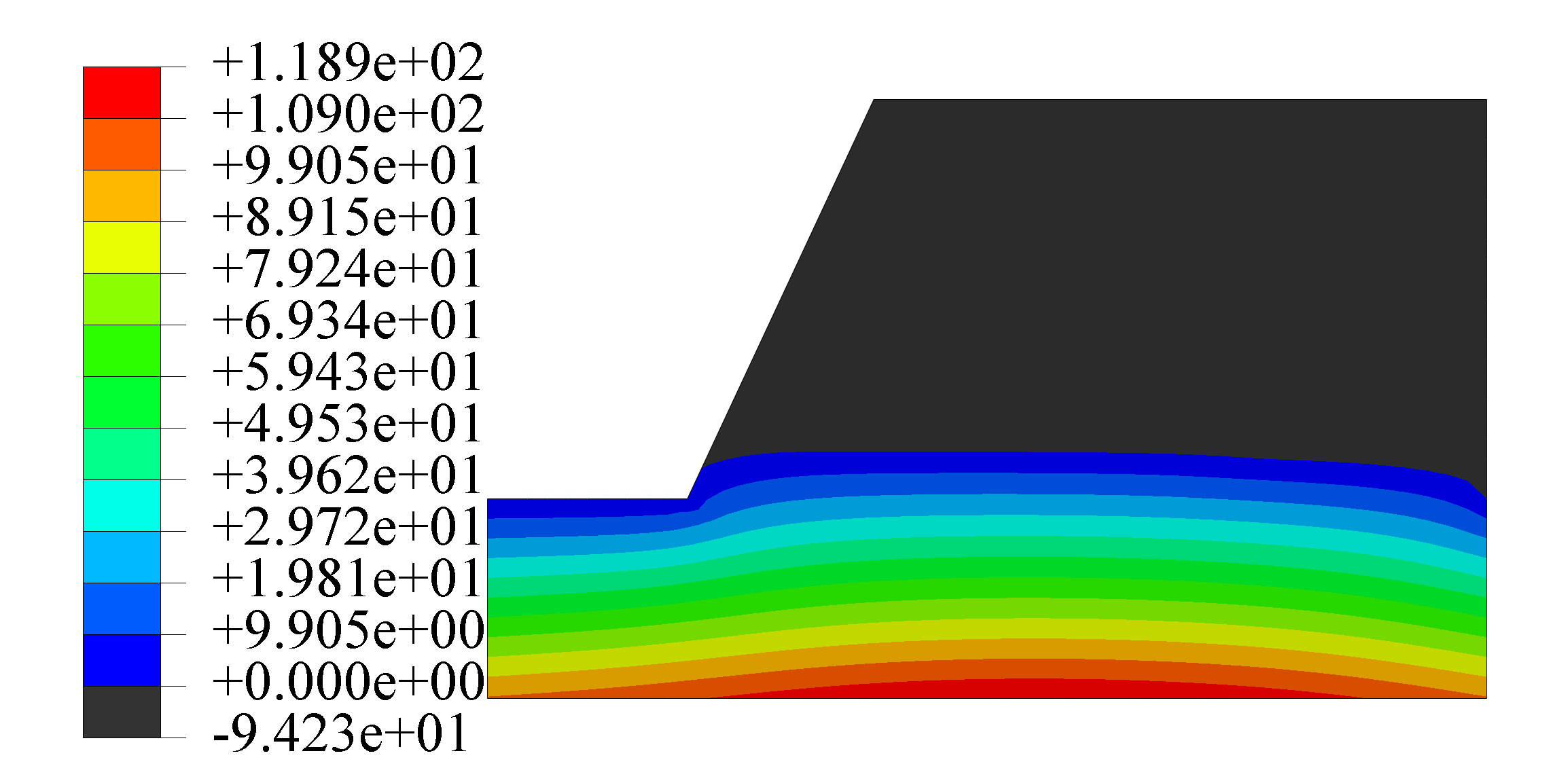

Supplement: Supplementary file 1 [file sensors-26-00421-s001.zip › Supplementary Materials/por65.png]

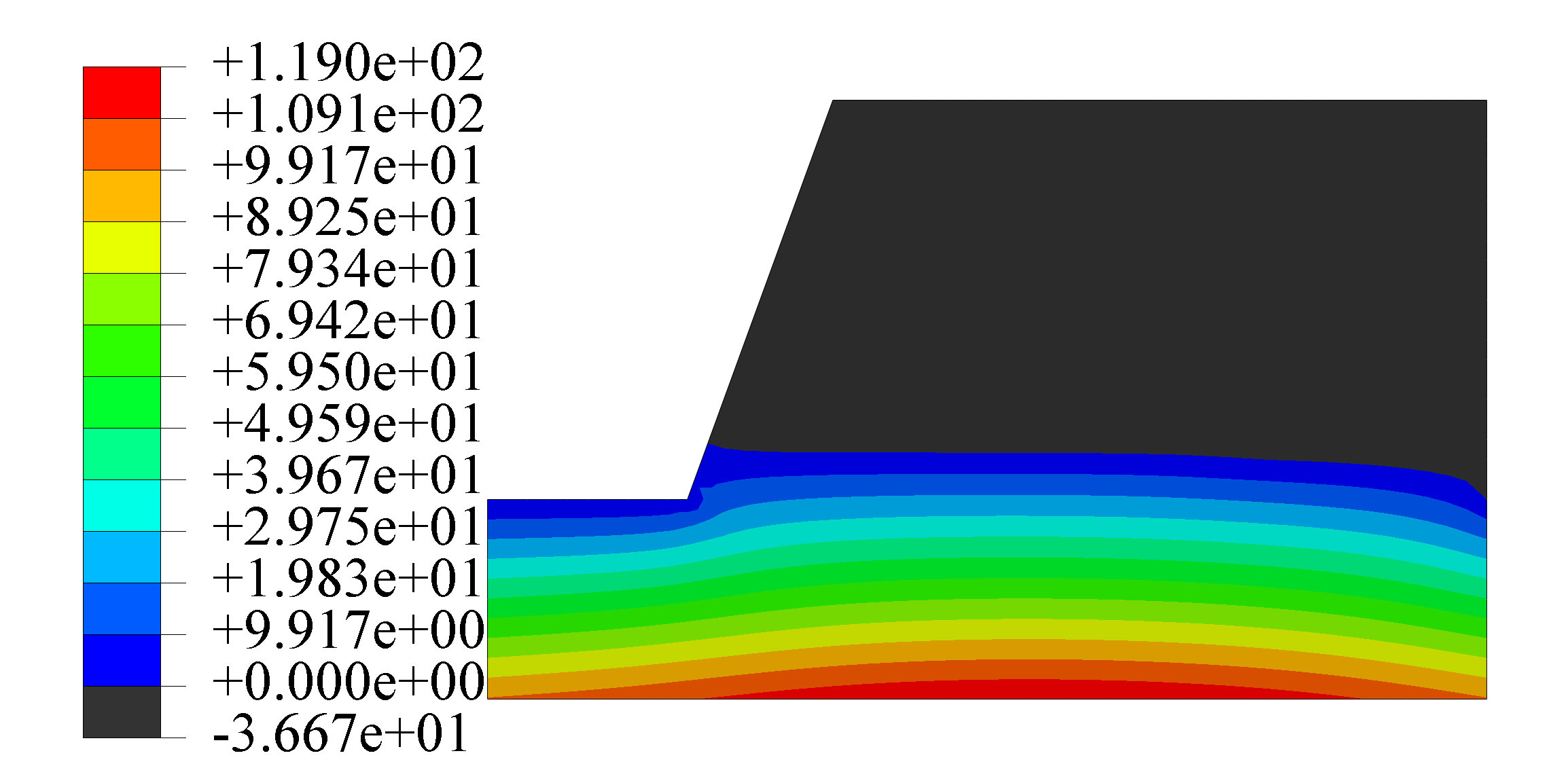

Supplement: Supplementary file 1 [file sensors-26-00421-s001.zip › Supplementary Materials/por70.png]

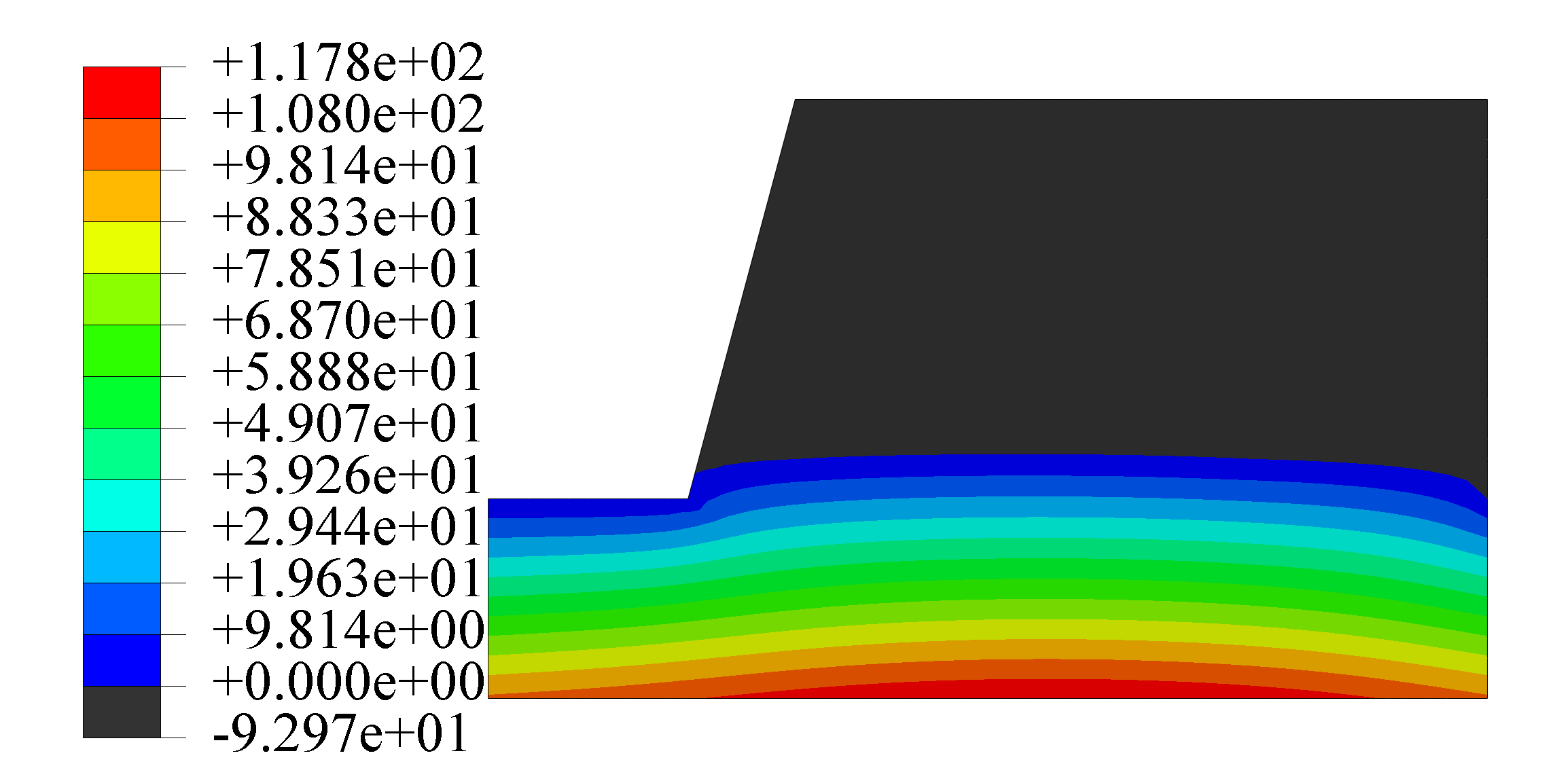

Supplement: Supplementary file 1 [file sensors-26-00421-s001.zip › Supplementary Materials/por75.png]

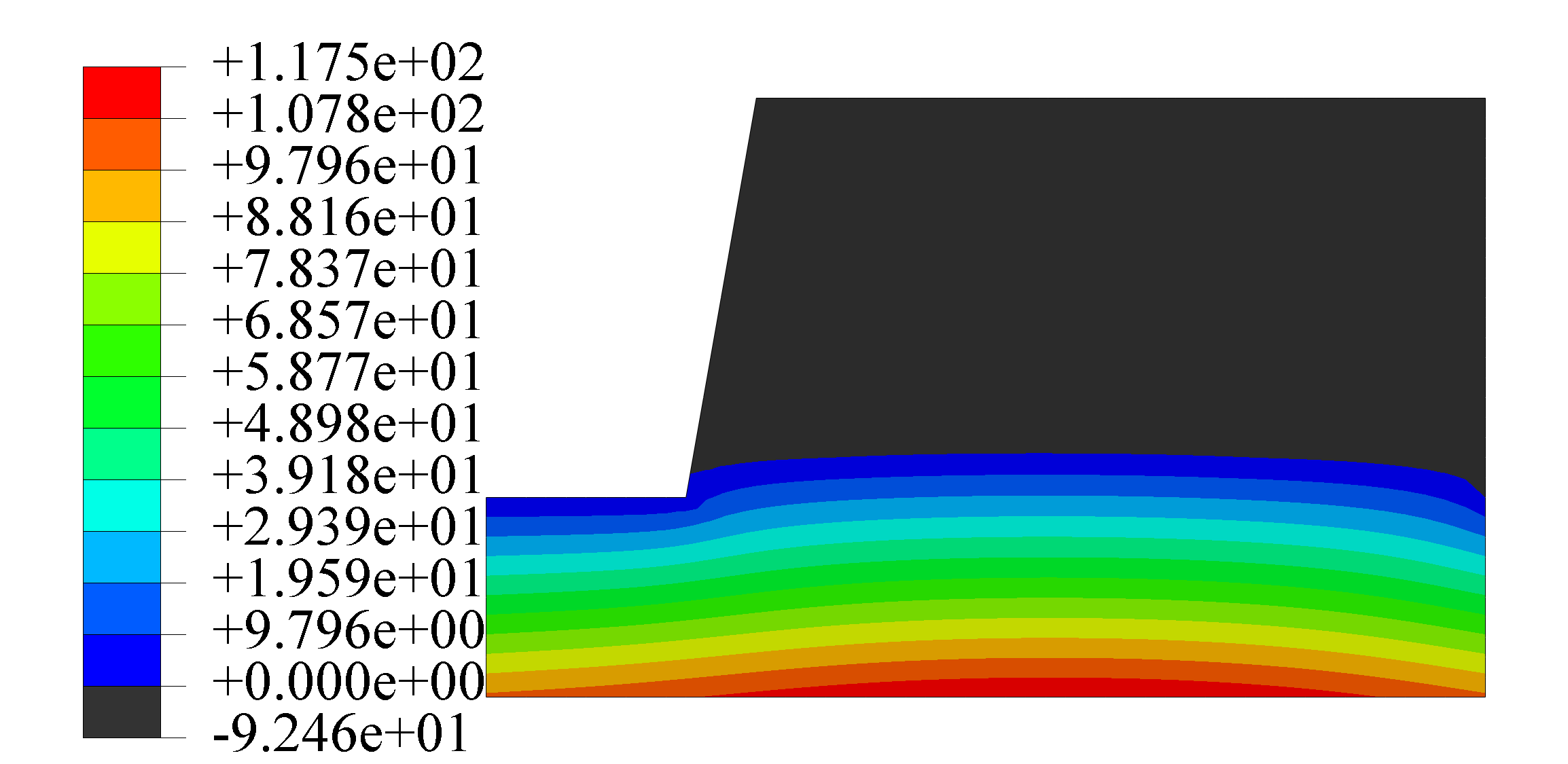

Supplement: Supplementary file 1 [file sensors-26-00421-s001.zip › Supplementary Materials/por80.png]

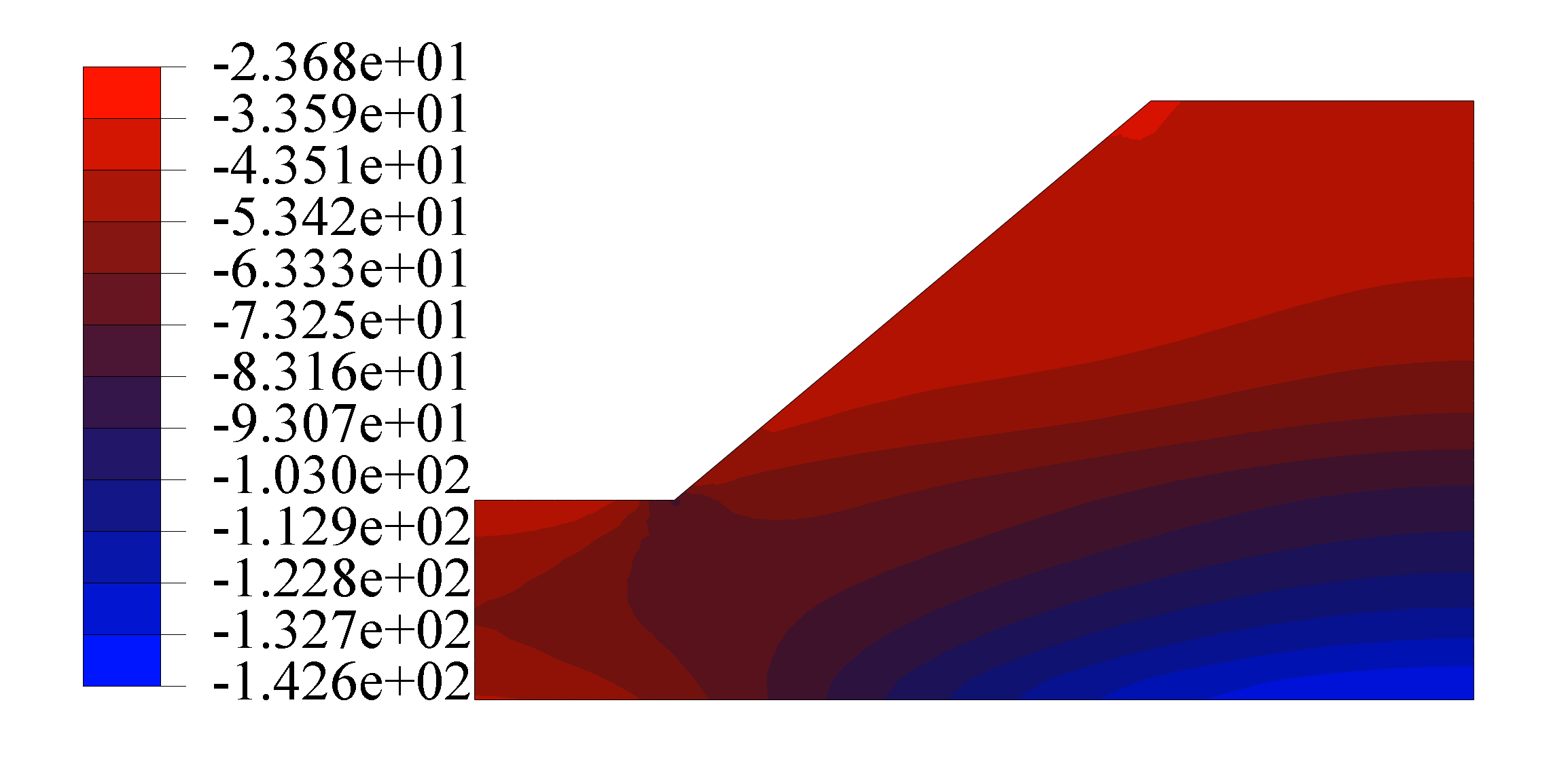

Supplement: Supplementary file 1 [file sensors-26-00421-s001.zip › Supplementary Materials/S1140.png]

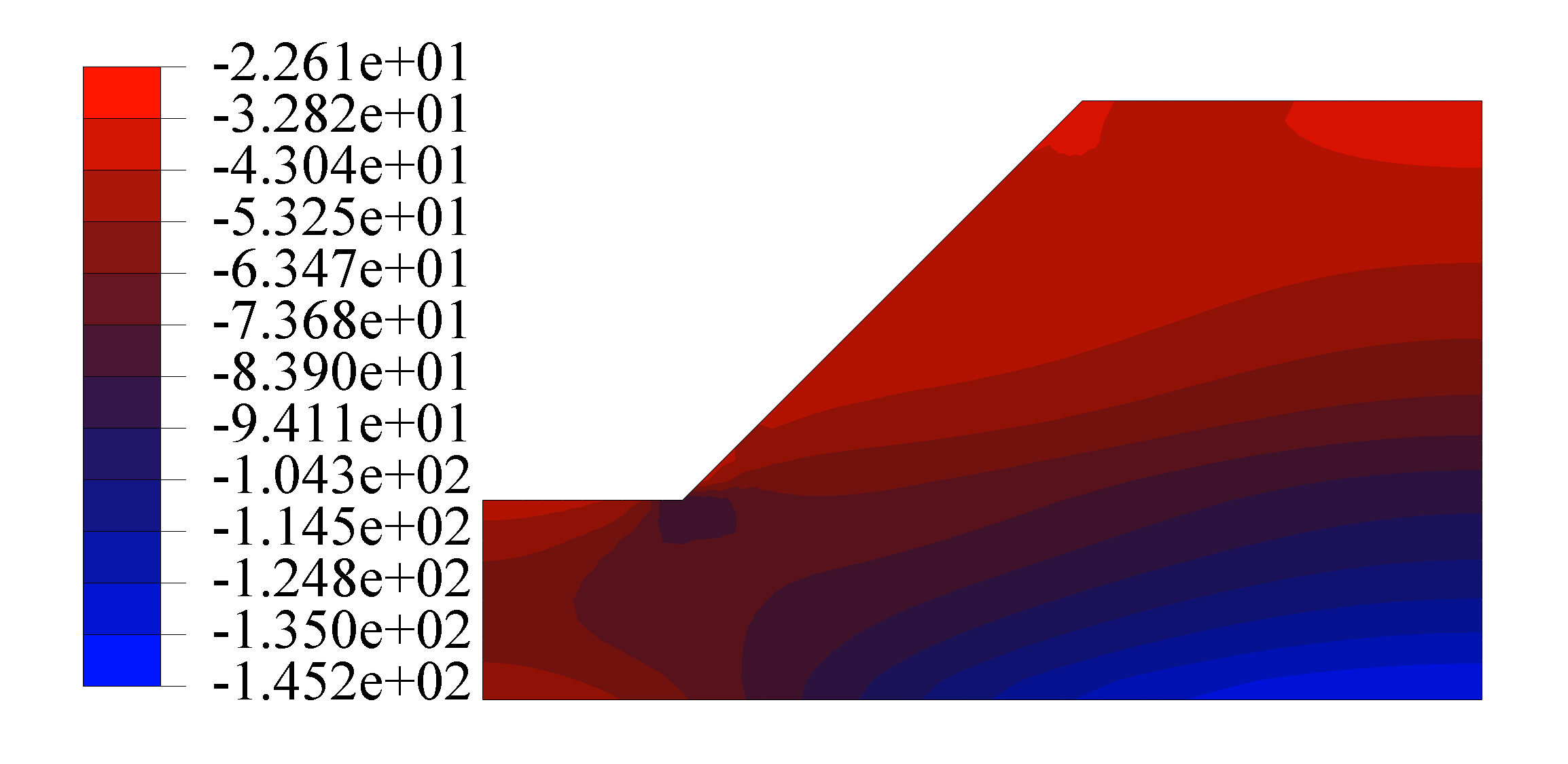

Supplement: Supplementary file 1 [file sensors-26-00421-s001.zip › Supplementary Materials/S1145.png]

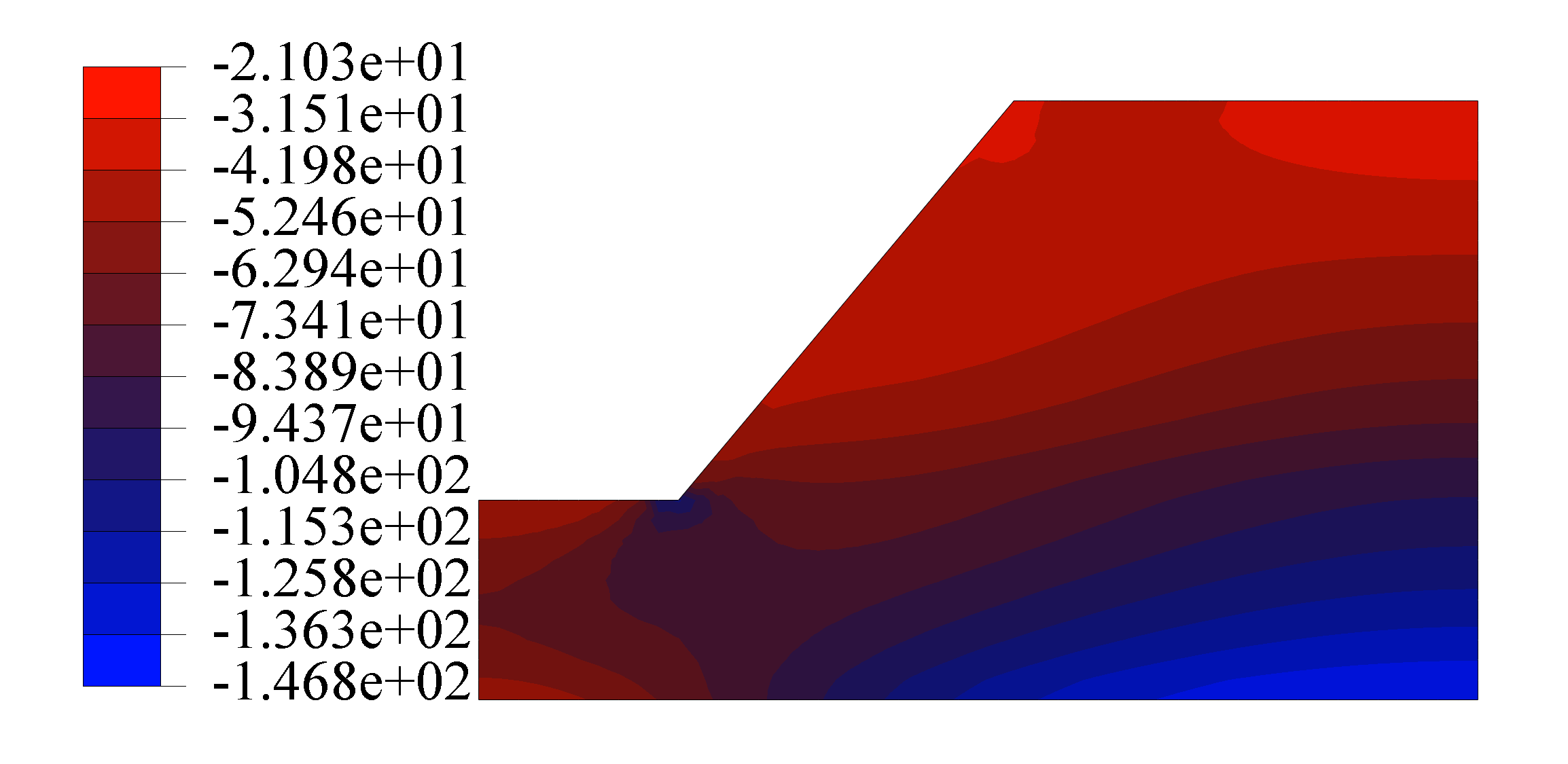

Supplement: Supplementary file 1 [file sensors-26-00421-s001.zip › Supplementary Materials/S1150.png]

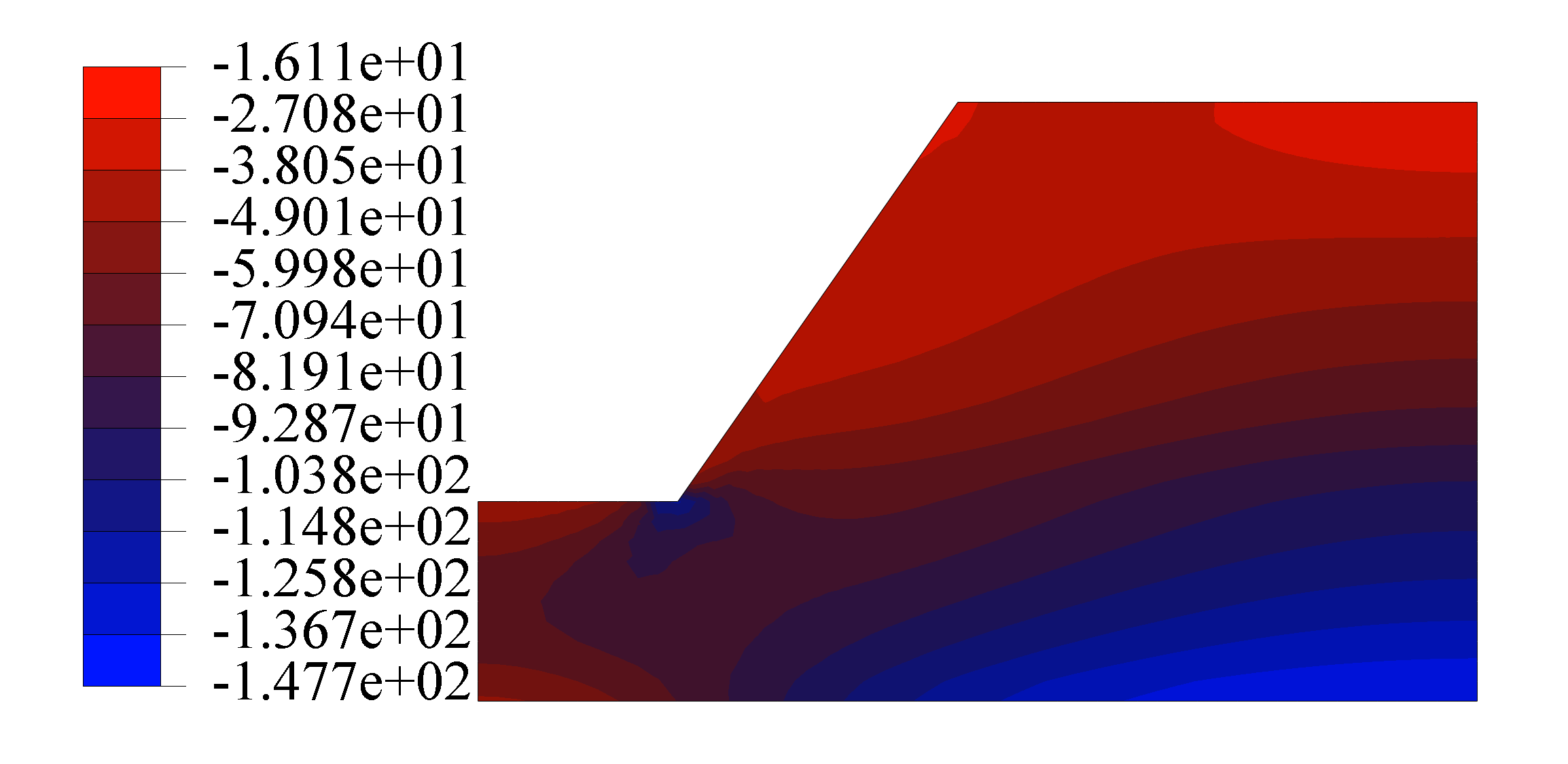

Supplement: Supplementary file 1 [file sensors-26-00421-s001.zip › Supplementary Materials/S1155.png]

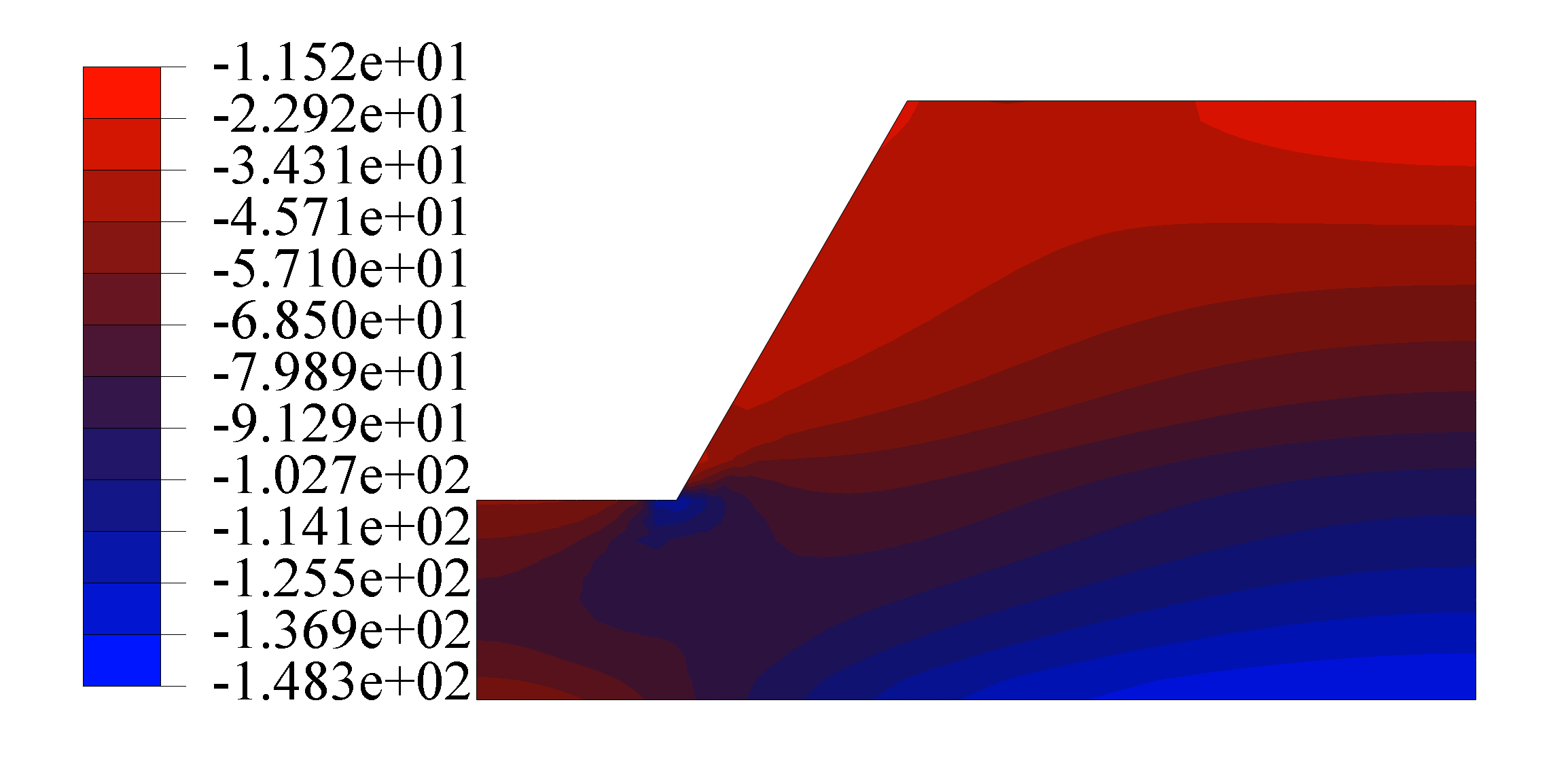

Supplement: Supplementary file 1 [file sensors-26-00421-s001.zip › Supplementary Materials/S1160.png]

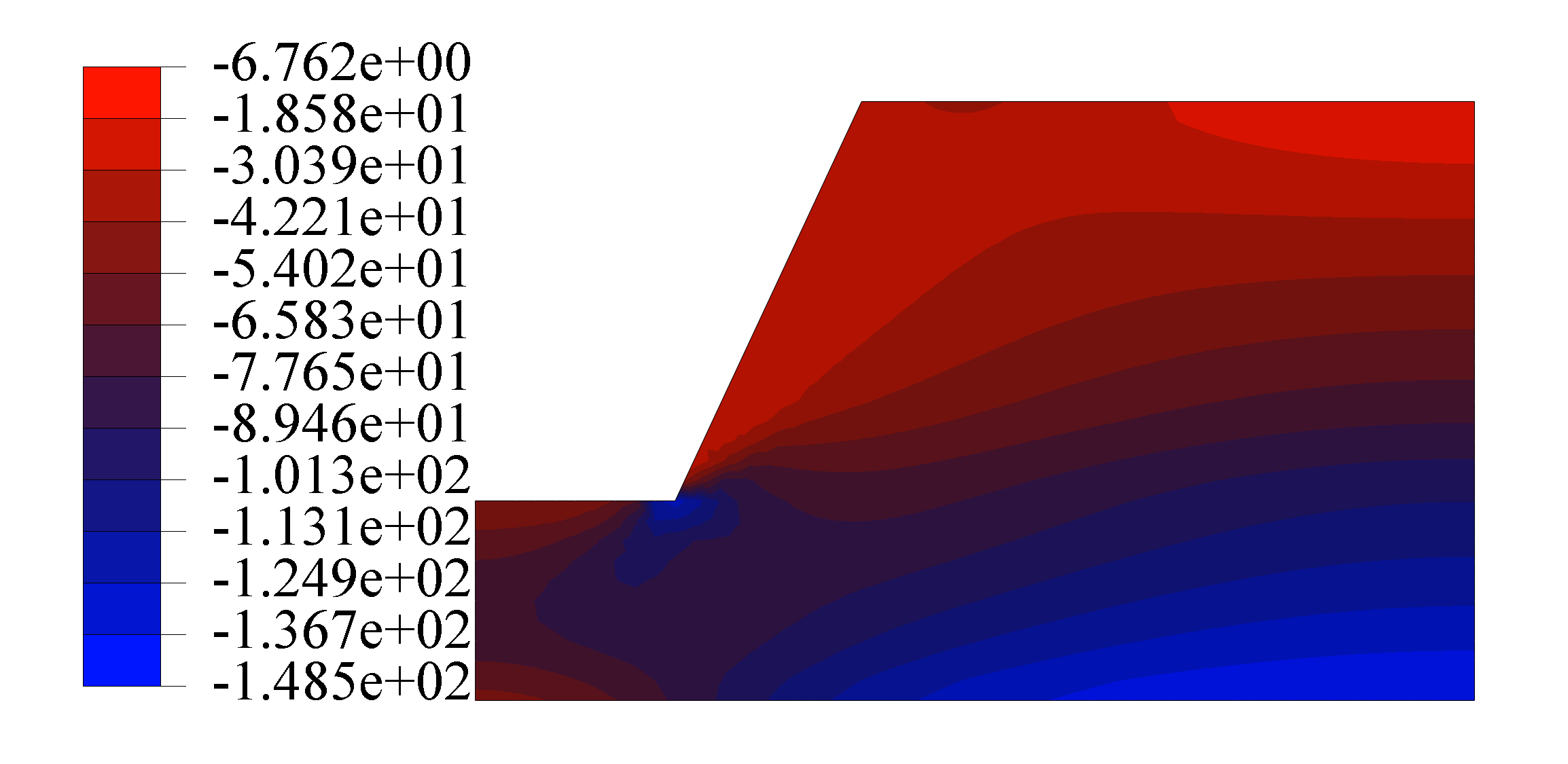

Supplement: Supplementary file 1 [file sensors-26-00421-s001.zip › Supplementary Materials/S1165.png]

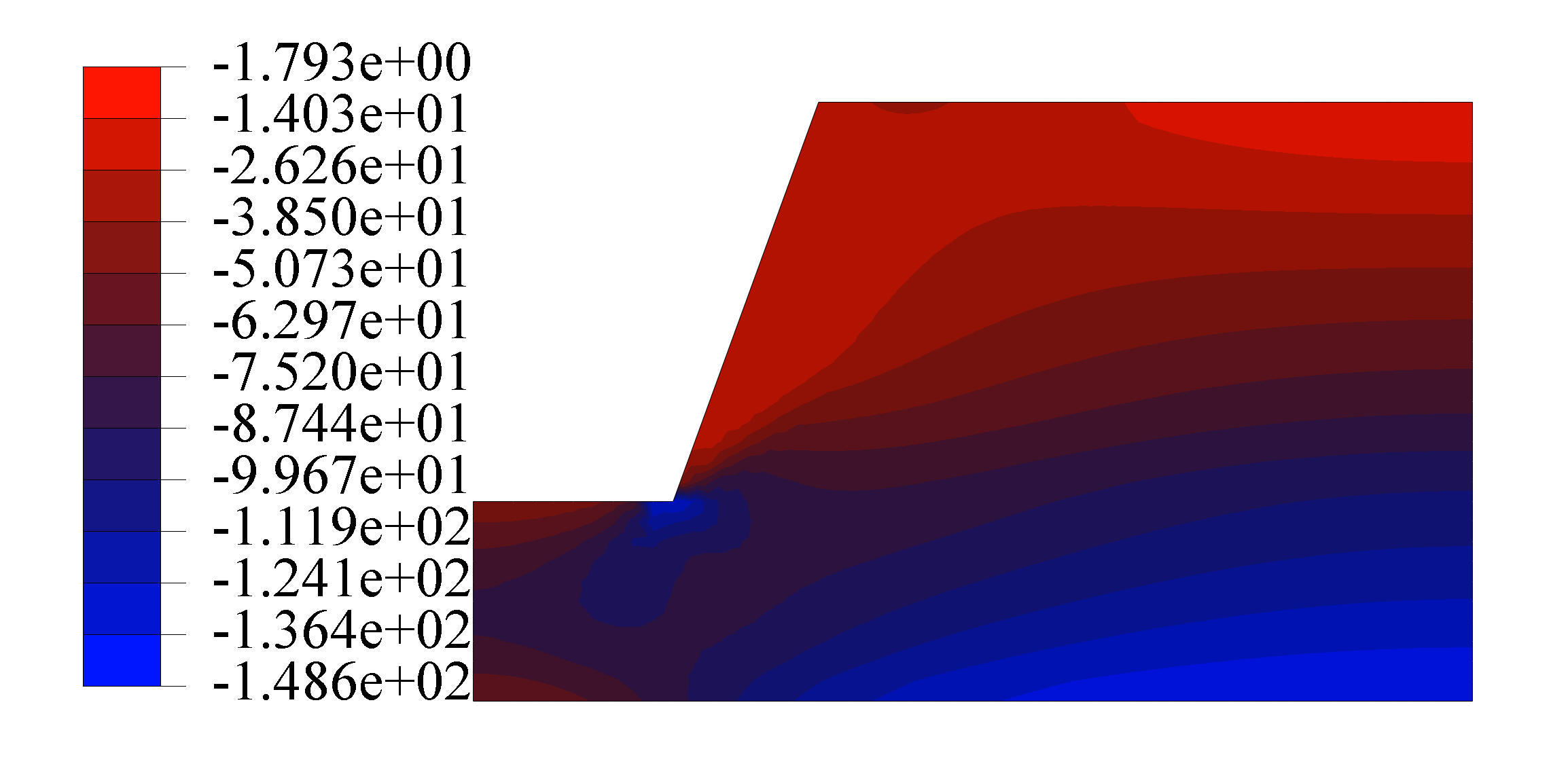

Supplement: Supplementary file 1 [file sensors-26-00421-s001.zip › Supplementary Materials/S1170.png]

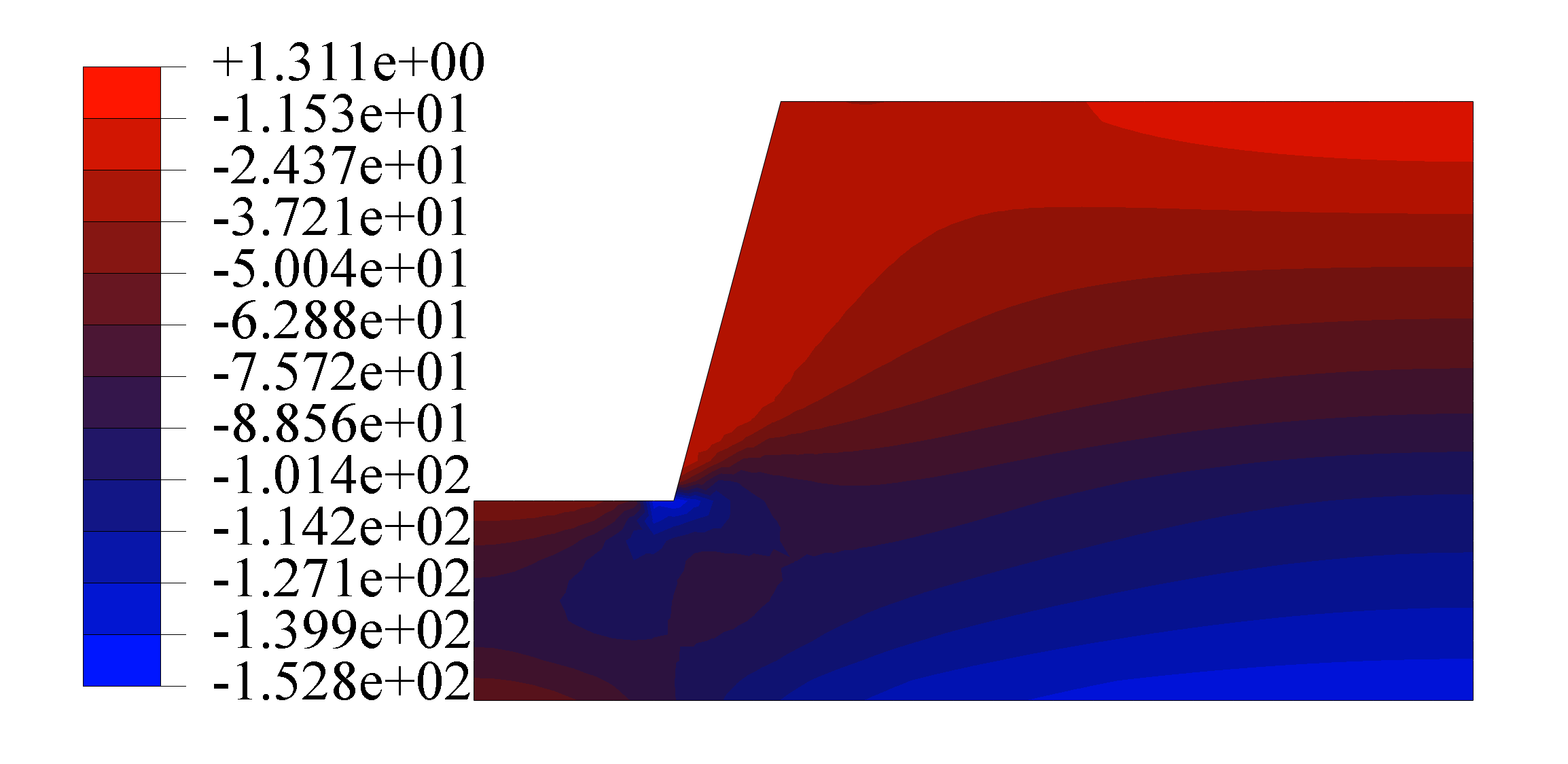

Supplement: Supplementary file 1 [file sensors-26-00421-s001.zip › Supplementary Materials/S1175.png]

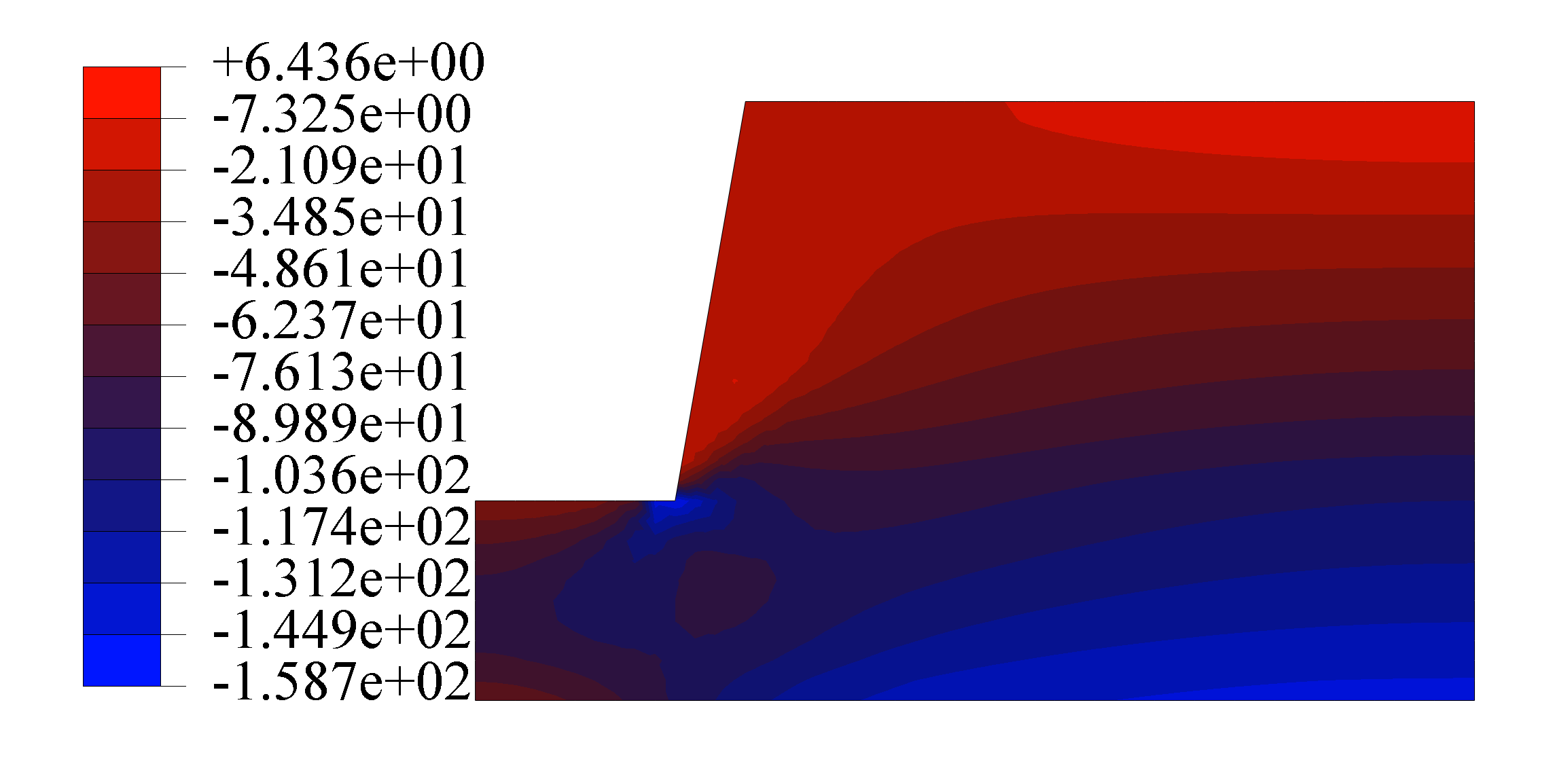

Supplement: Supplementary file 1 [file sensors-26-00421-s001.zip › Supplementary Materials/S1180.png]

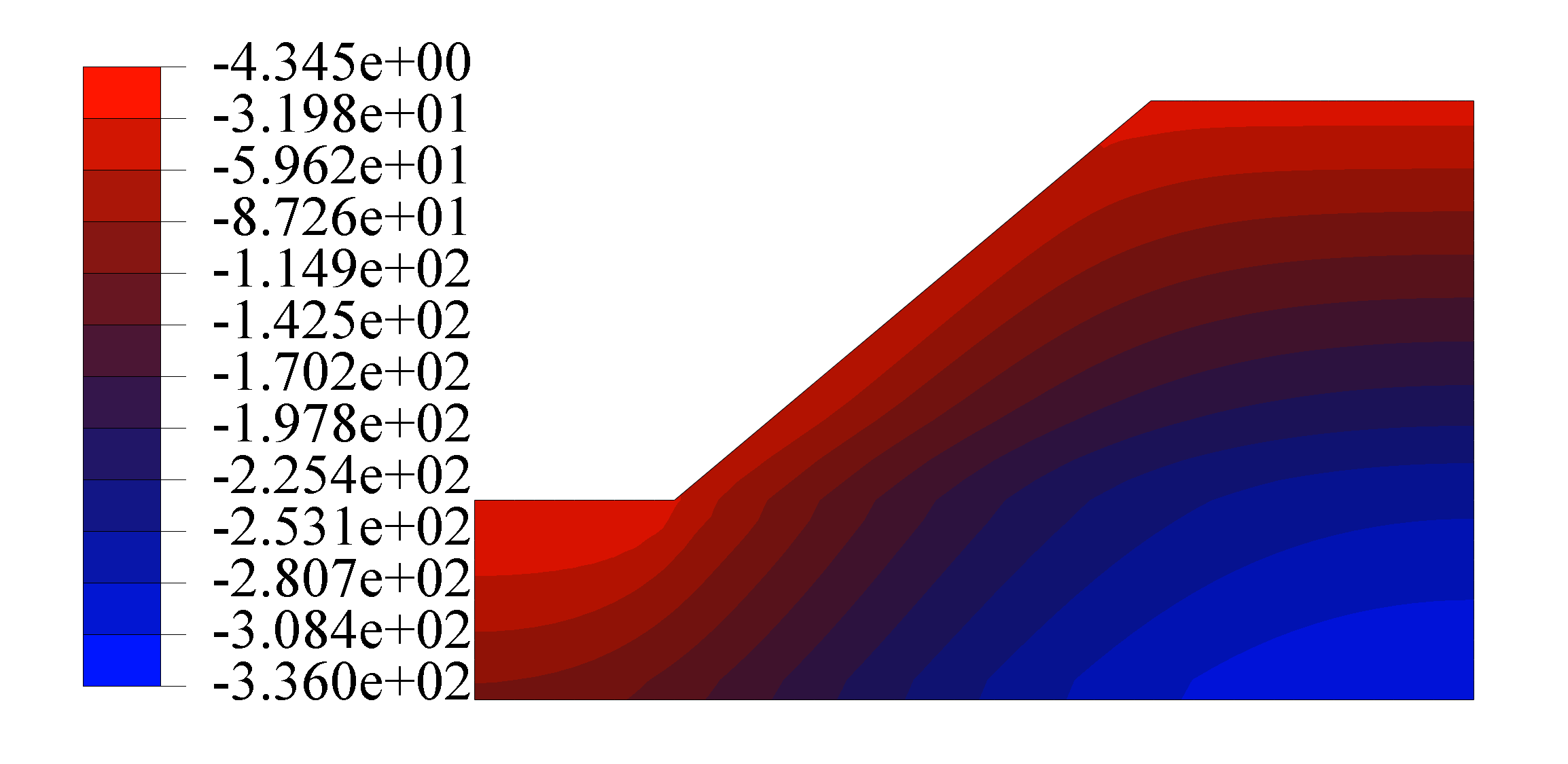

Supplement: Supplementary file 1 [file sensors-26-00421-s001.zip › Supplementary Materials/S2240.png]

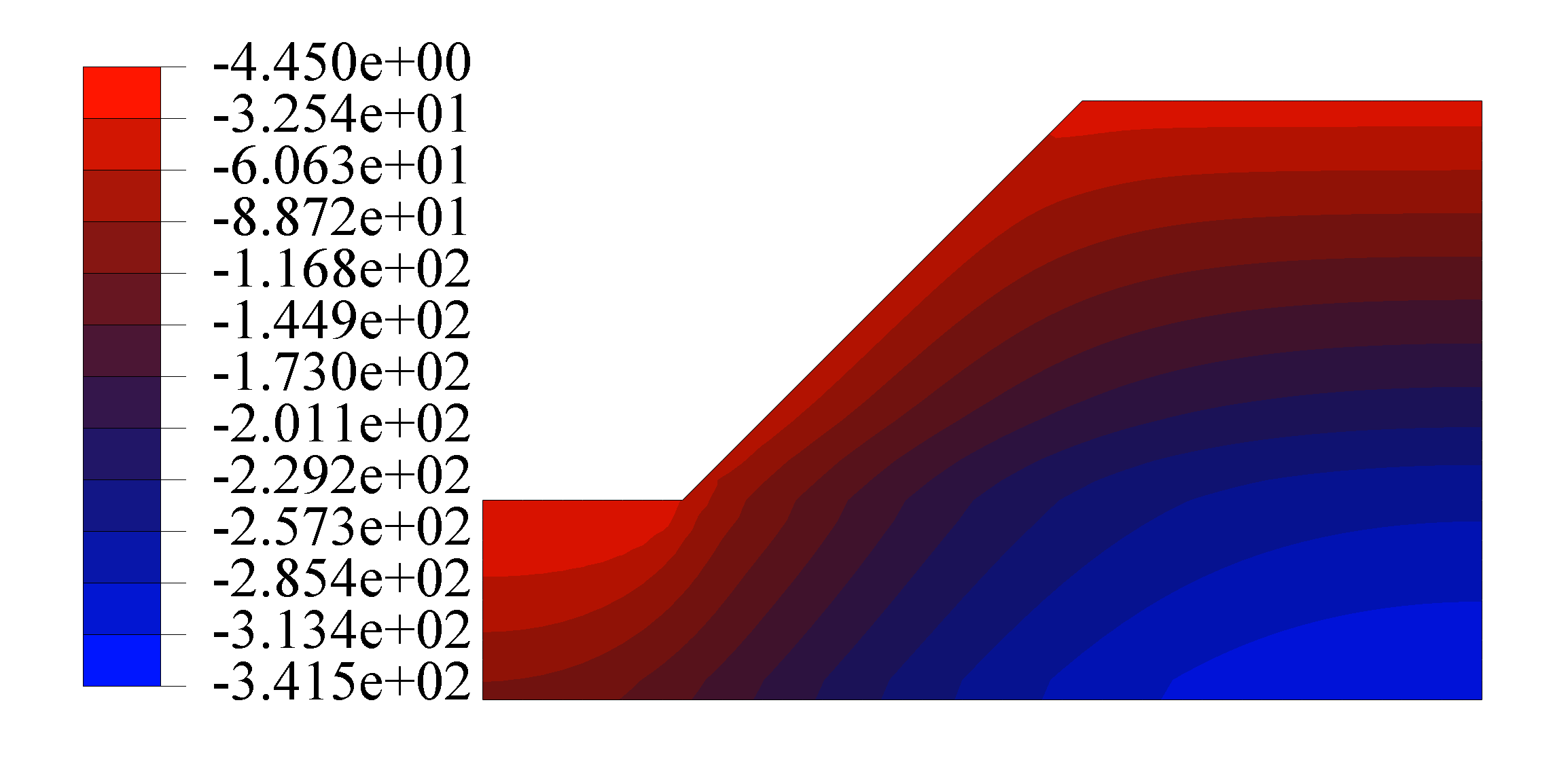

Supplement: Supplementary file 1 [file sensors-26-00421-s001.zip › Supplementary Materials/S2245.png]

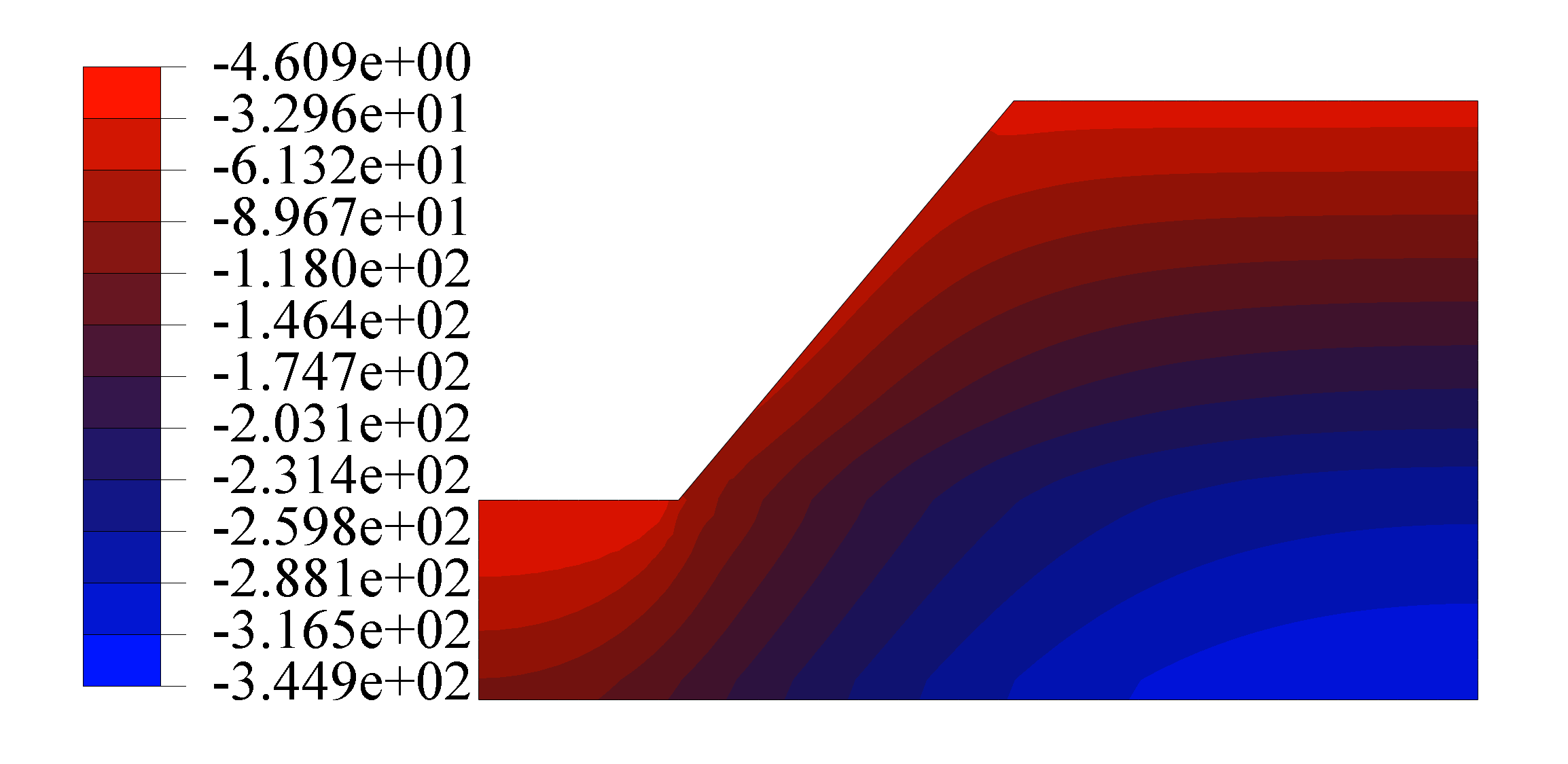

Supplement: Supplementary file 1 [file sensors-26-00421-s001.zip › Supplementary Materials/S2250.png]

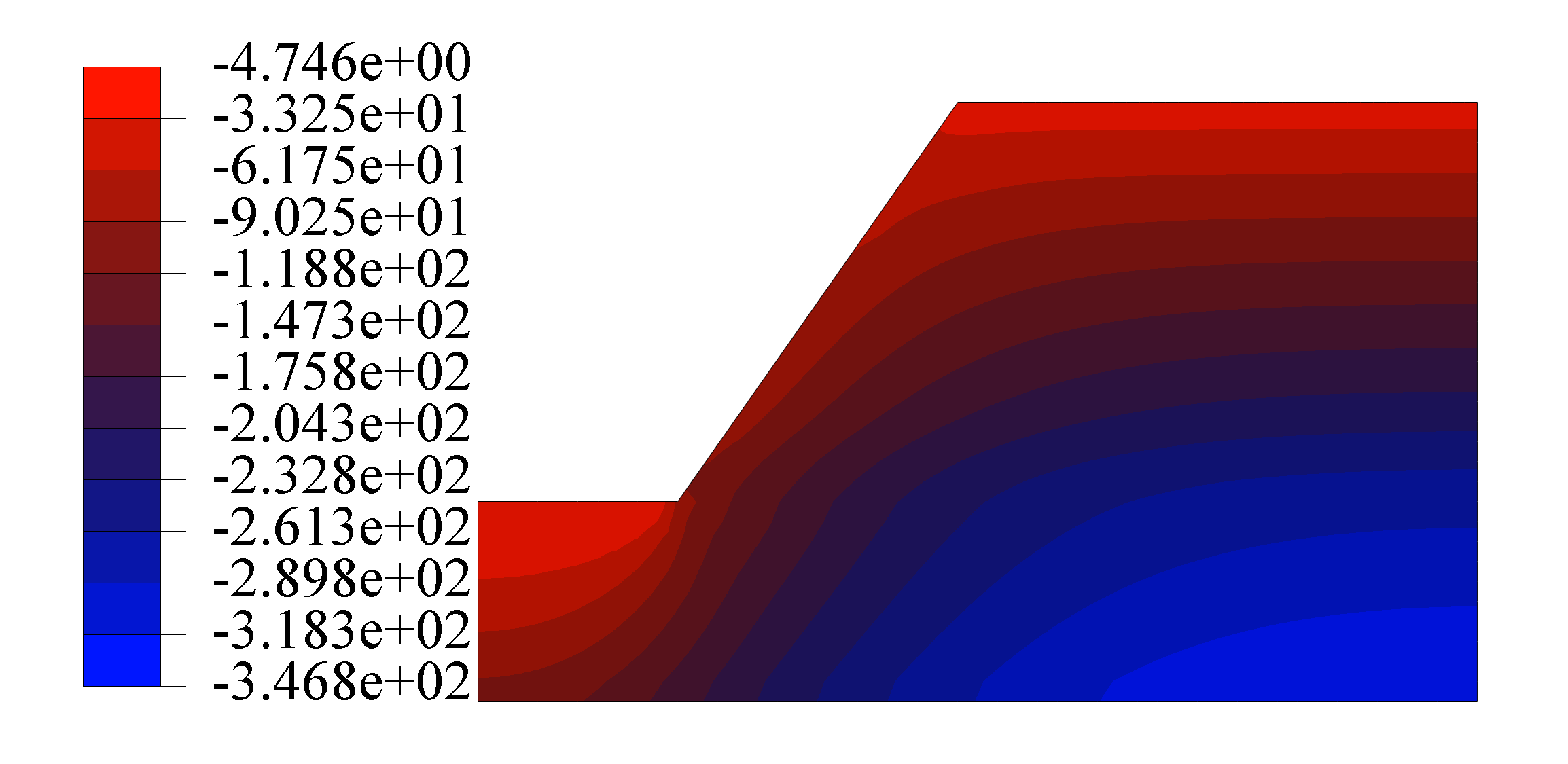

Supplement: Supplementary file 1 [file sensors-26-00421-s001.zip › Supplementary Materials/S2255.png]

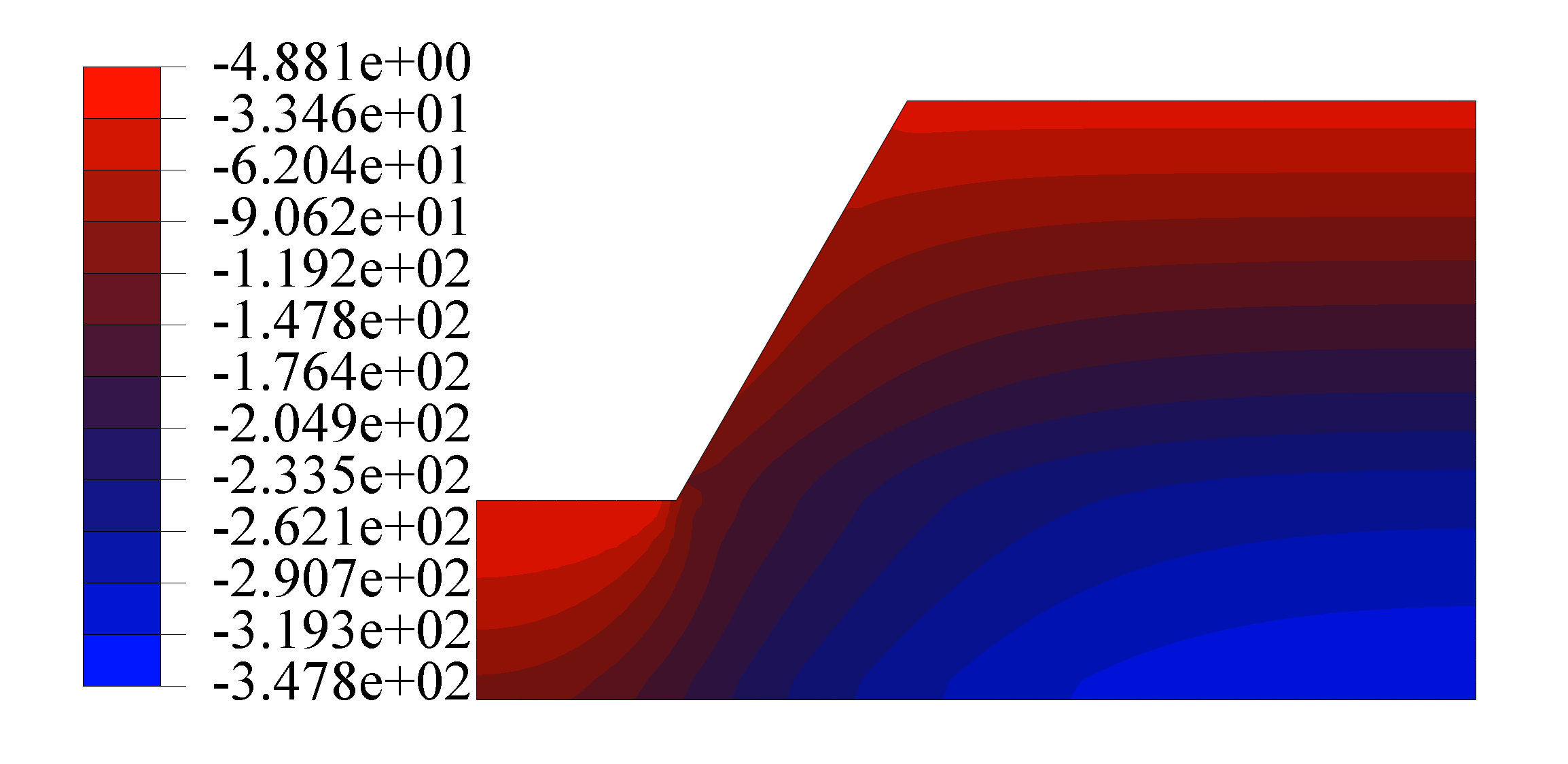

Supplement: Supplementary file 1 [file sensors-26-00421-s001.zip › Supplementary Materials/S2260.png]

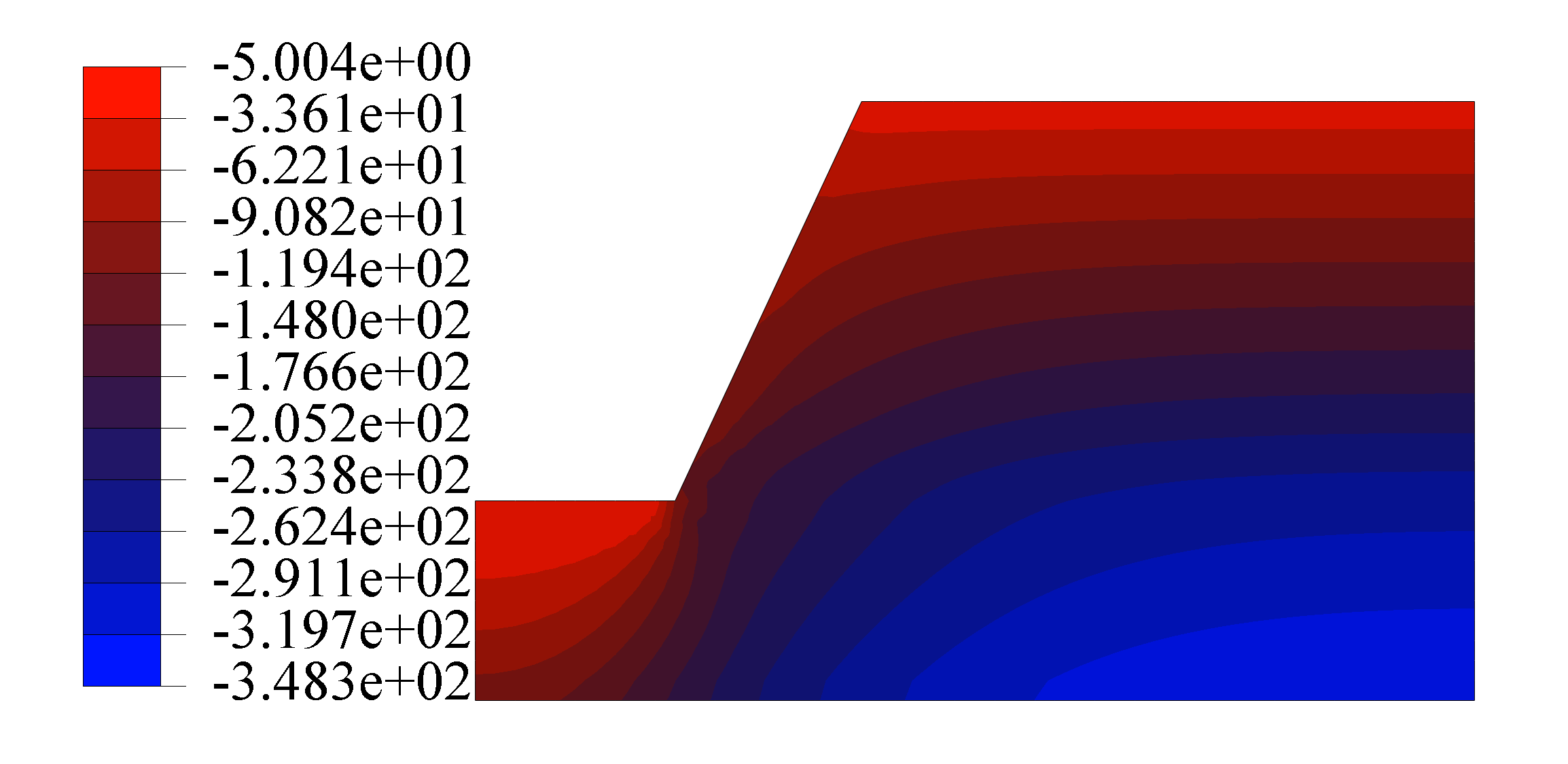

Supplement: Supplementary file 1 [file sensors-26-00421-s001.zip › Supplementary Materials/S2265.png]

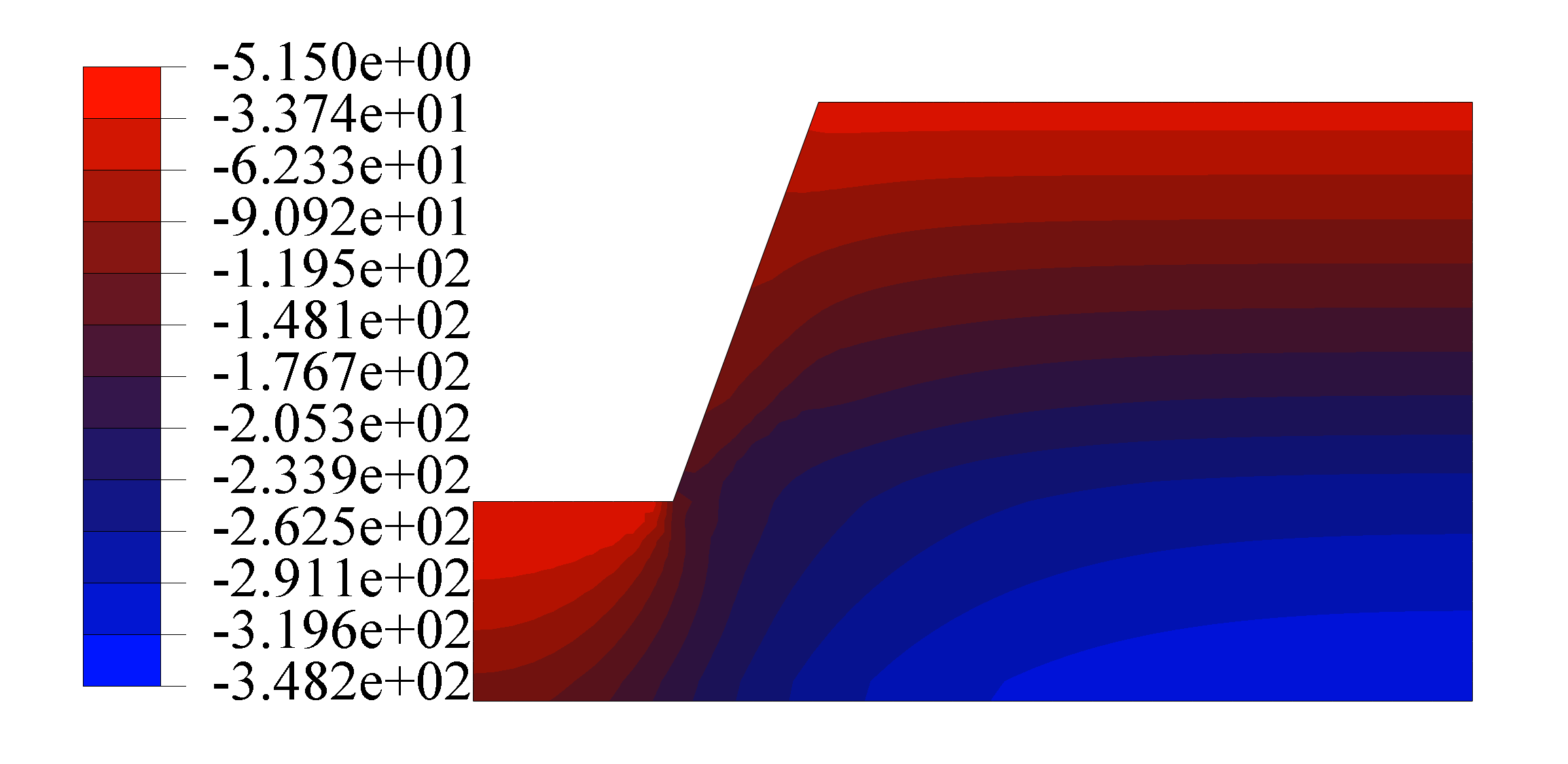

Supplement: Supplementary file 1 [file sensors-26-00421-s001.zip › Supplementary Materials/S2270.png]

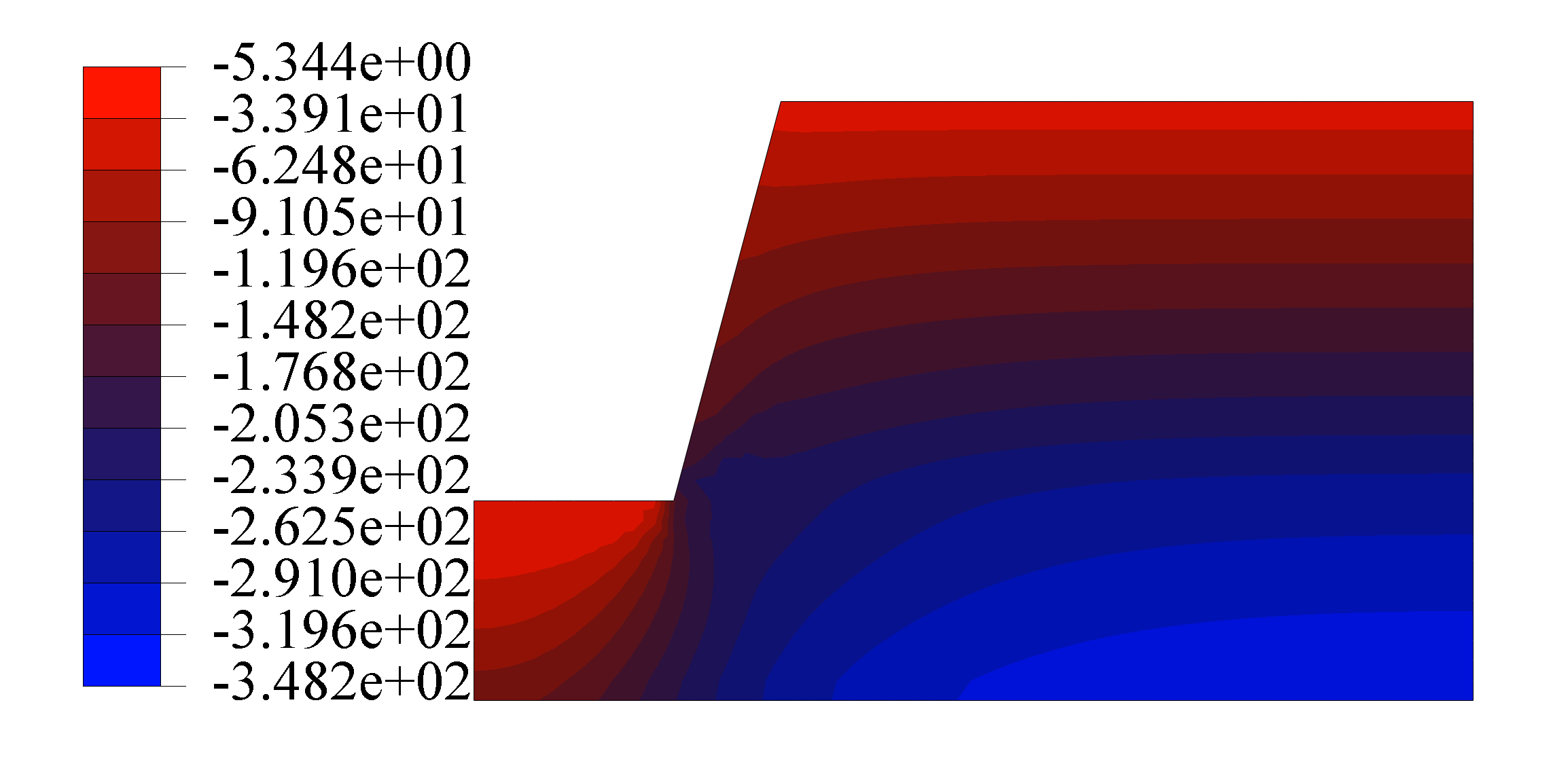

Supplement: Supplementary file 1 [file sensors-26-00421-s001.zip › Supplementary Materials/S2275.png]

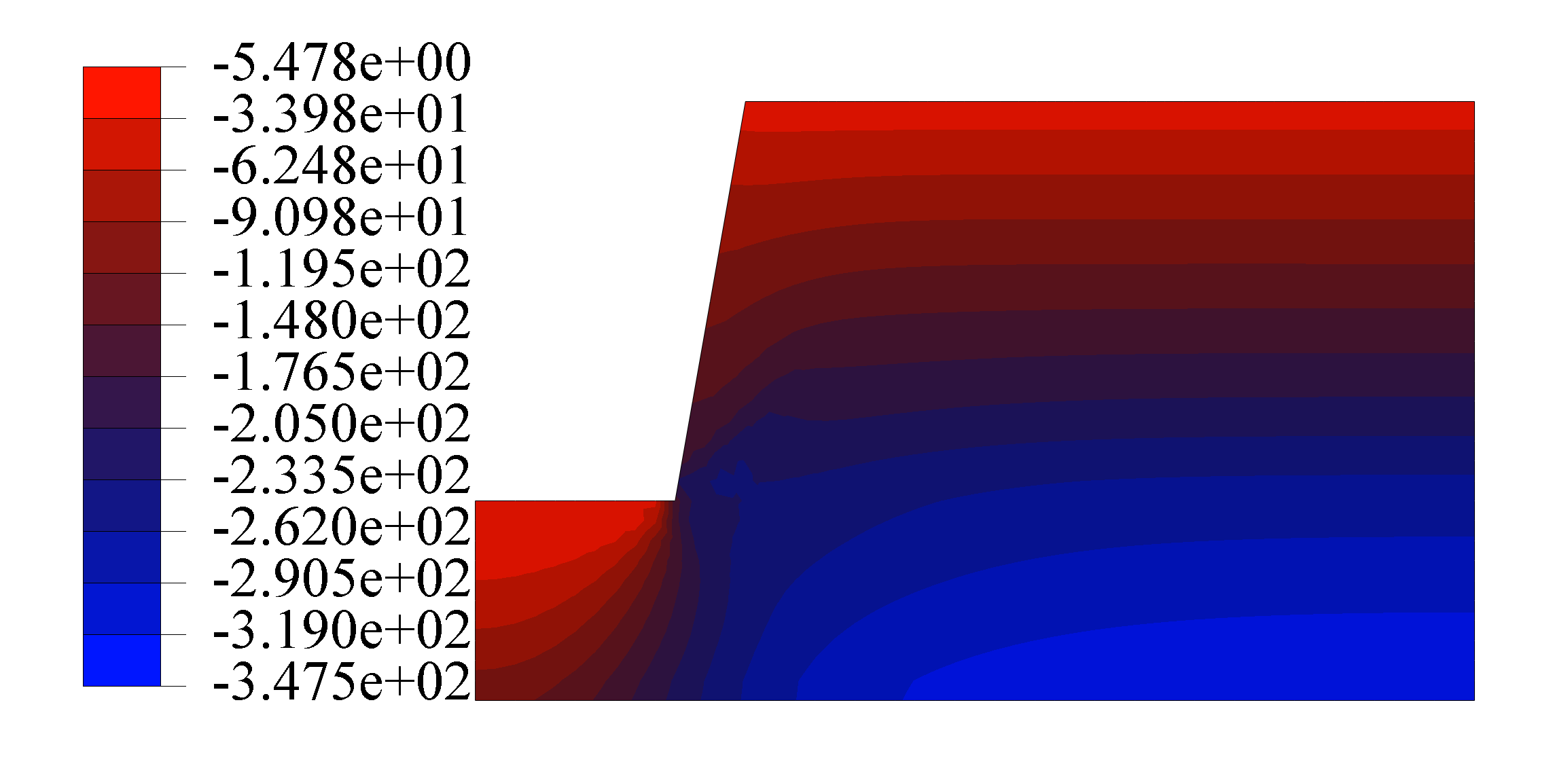

Supplement: Supplementary file 1 [file sensors-26-00421-s001.zip › Supplementary Materials/S2280.png]

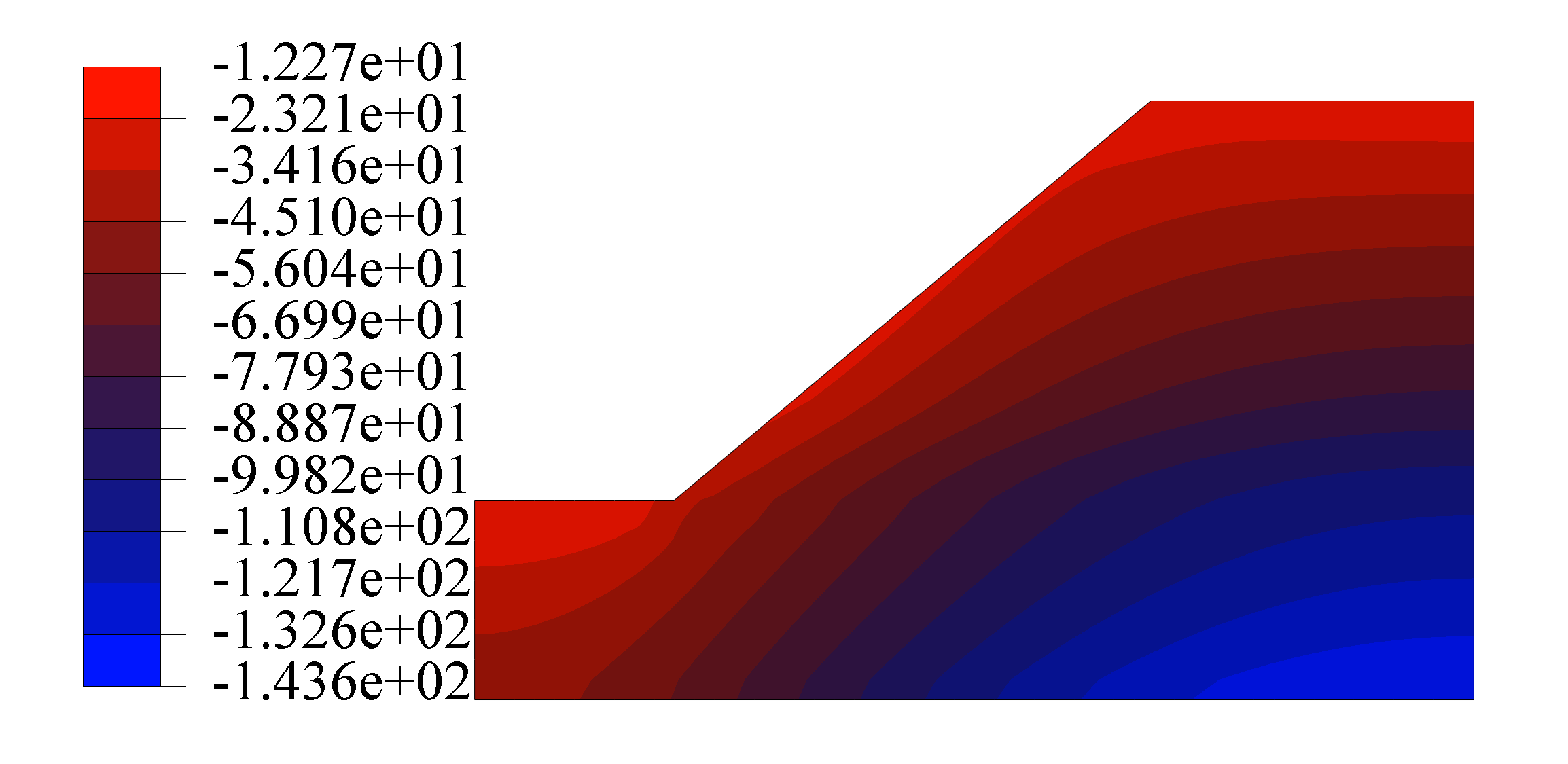

Supplement: Supplementary file 1 [file sensors-26-00421-s001.zip › Supplementary Materials/S3340.png]

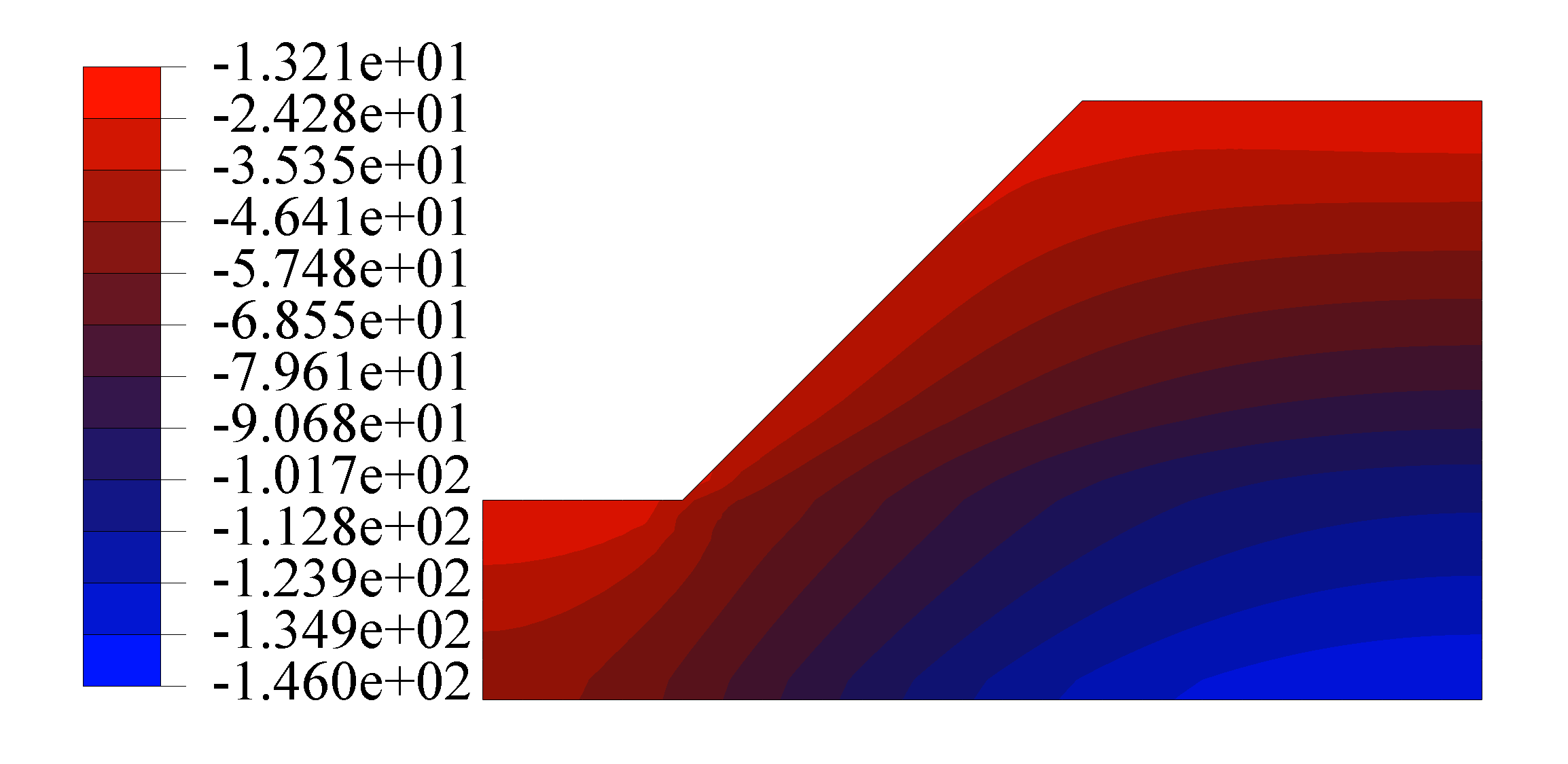

Supplement: Supplementary file 1 [file sensors-26-00421-s001.zip › Supplementary Materials/S3345.png]

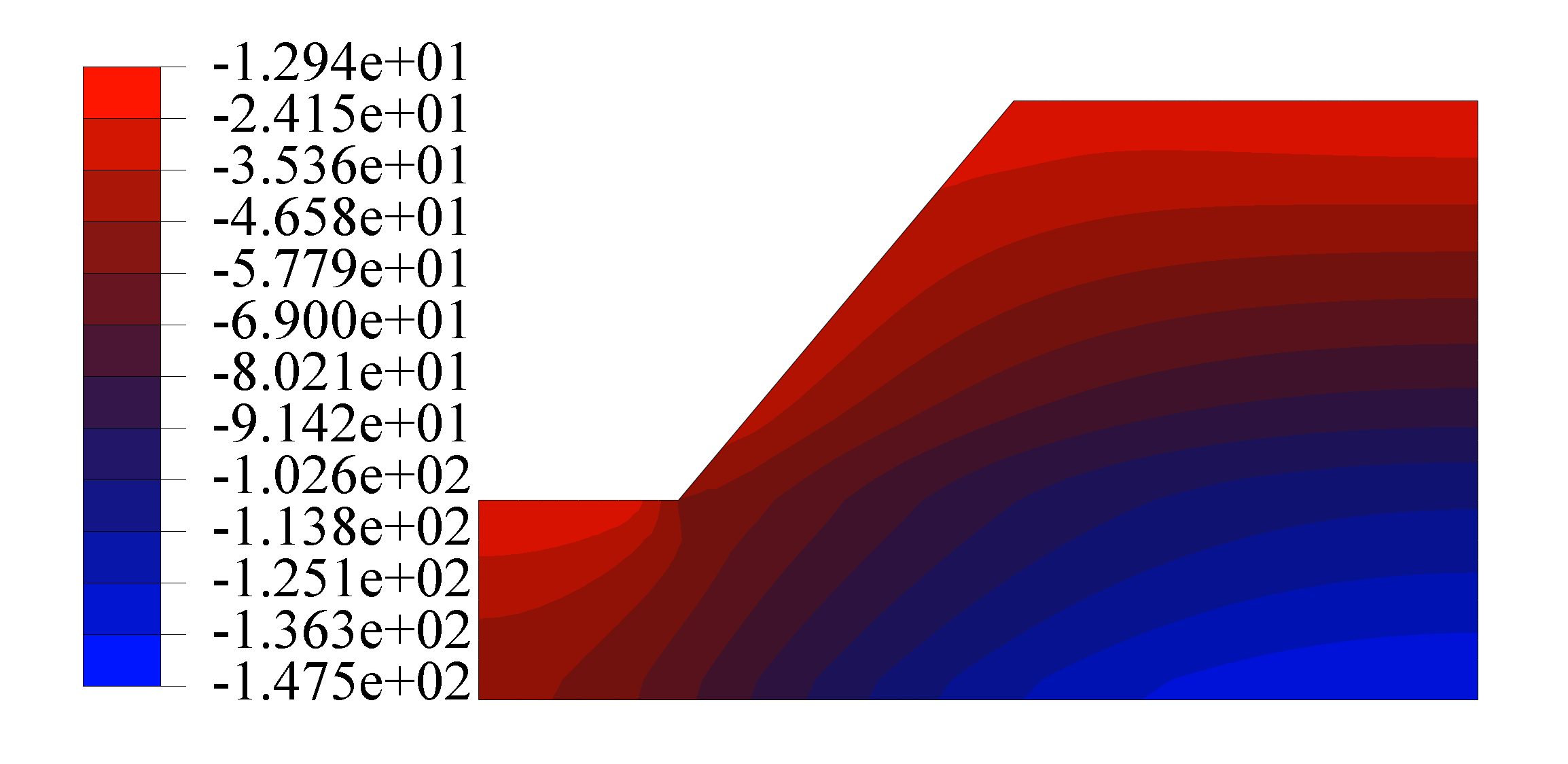

Supplement: Supplementary file 1 [file sensors-26-00421-s001.zip › Supplementary Materials/S3350.png]

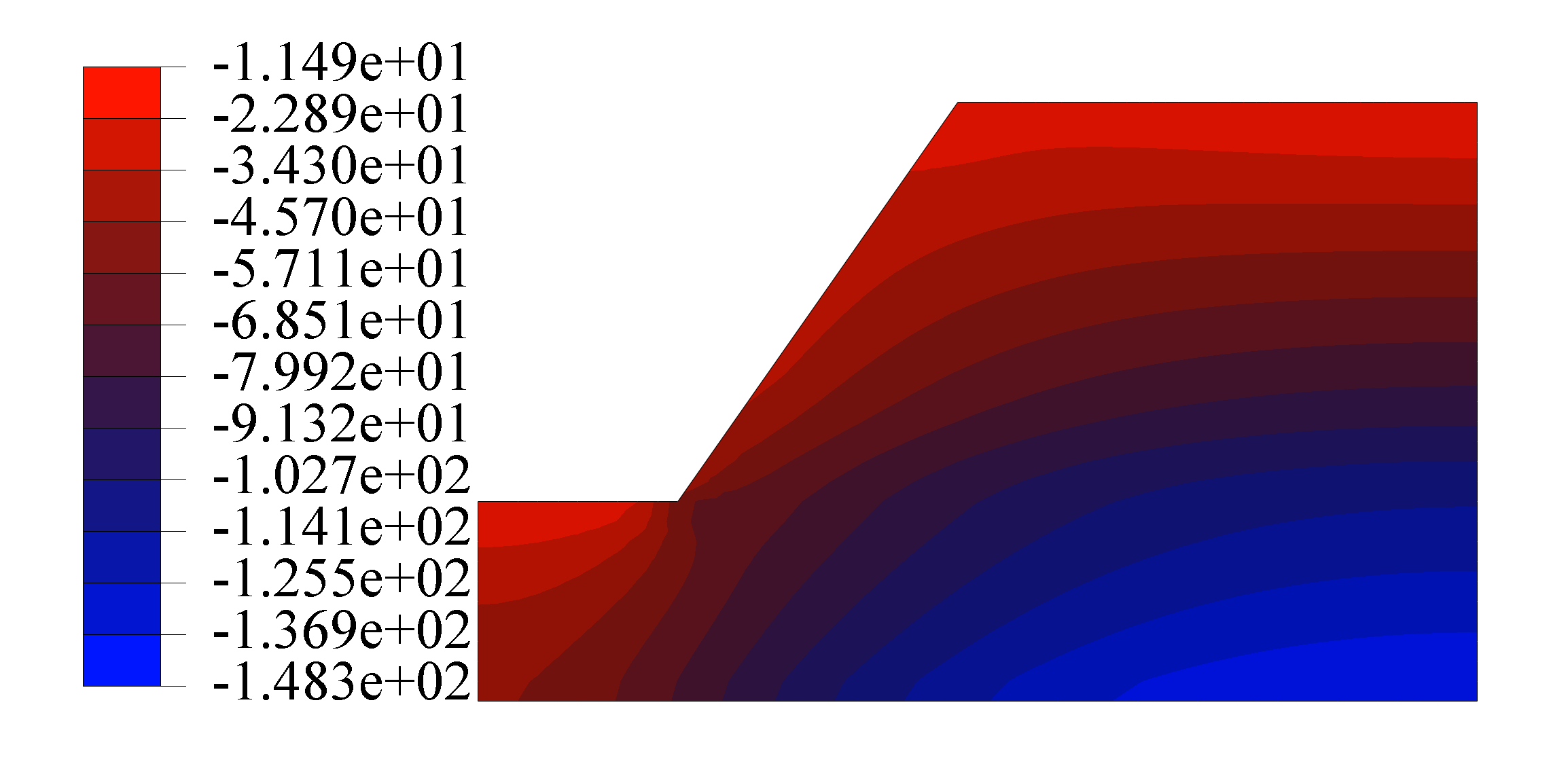

Supplement: Supplementary file 1 [file sensors-26-00421-s001.zip › Supplementary Materials/S3355.png]

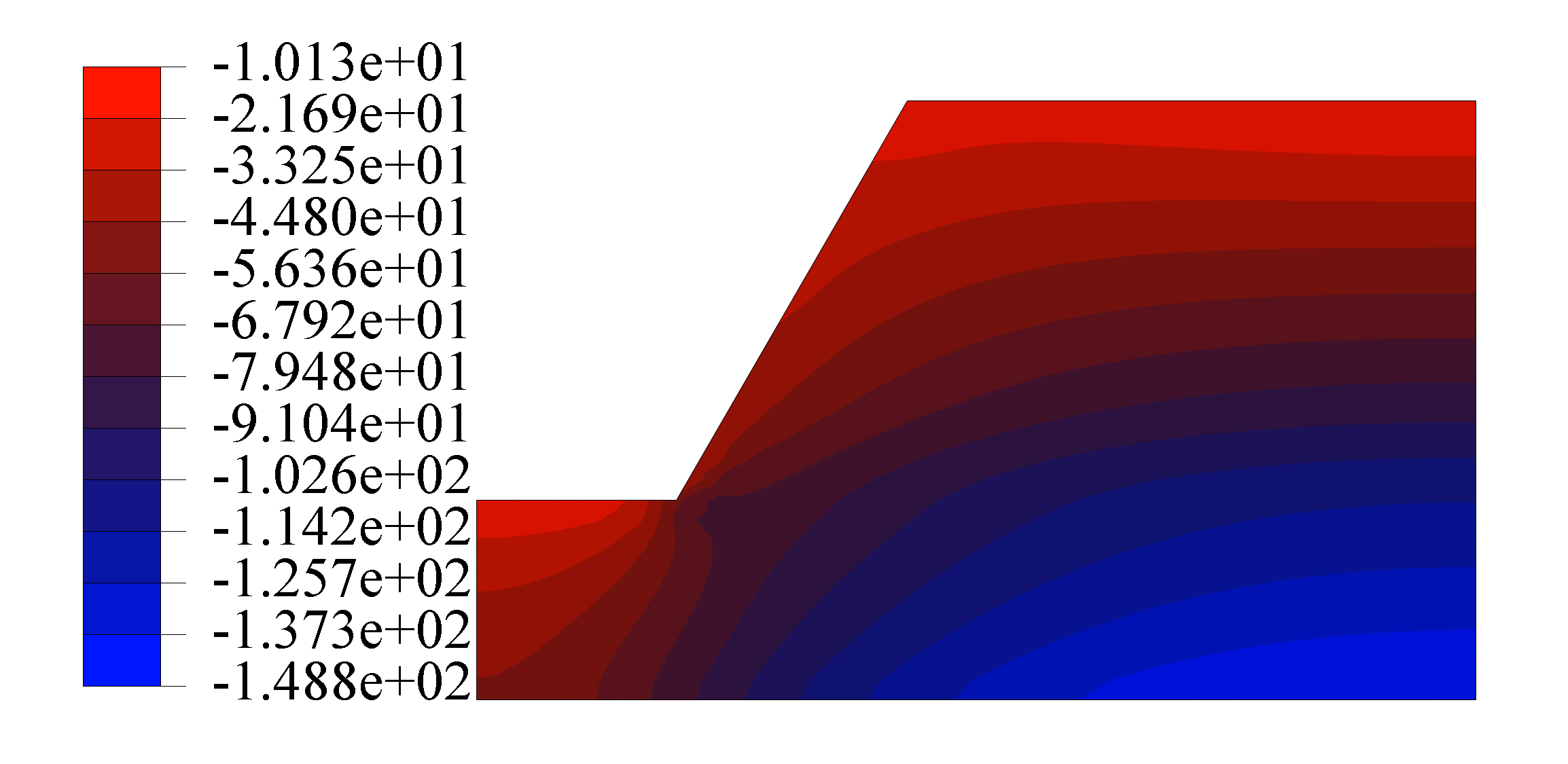

Supplement: Supplementary file 1 [file sensors-26-00421-s001.zip › Supplementary Materials/S3360.png]

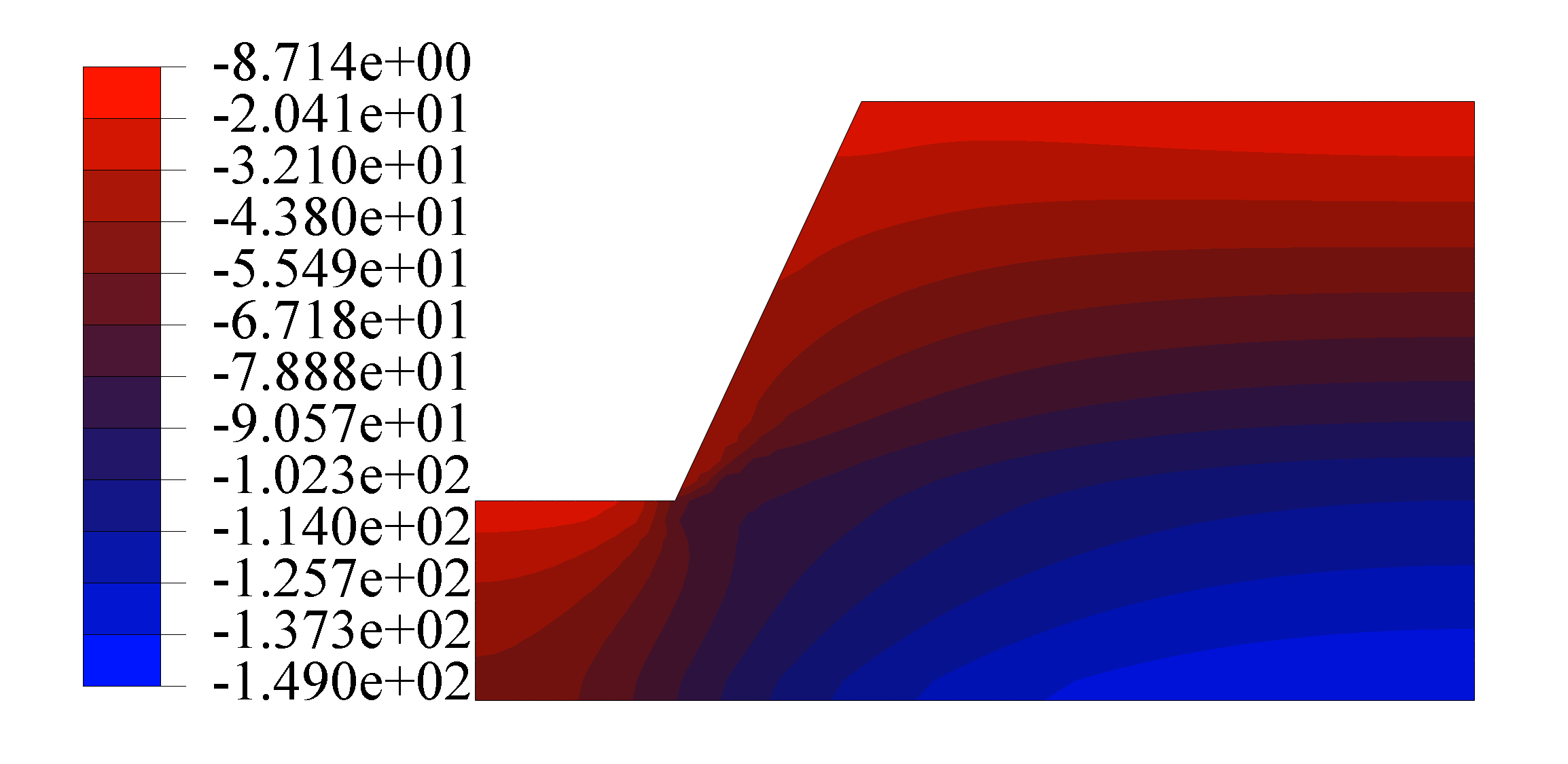

Supplement: Supplementary file 1 [file sensors-26-00421-s001.zip › Supplementary Materials/S3365.png]

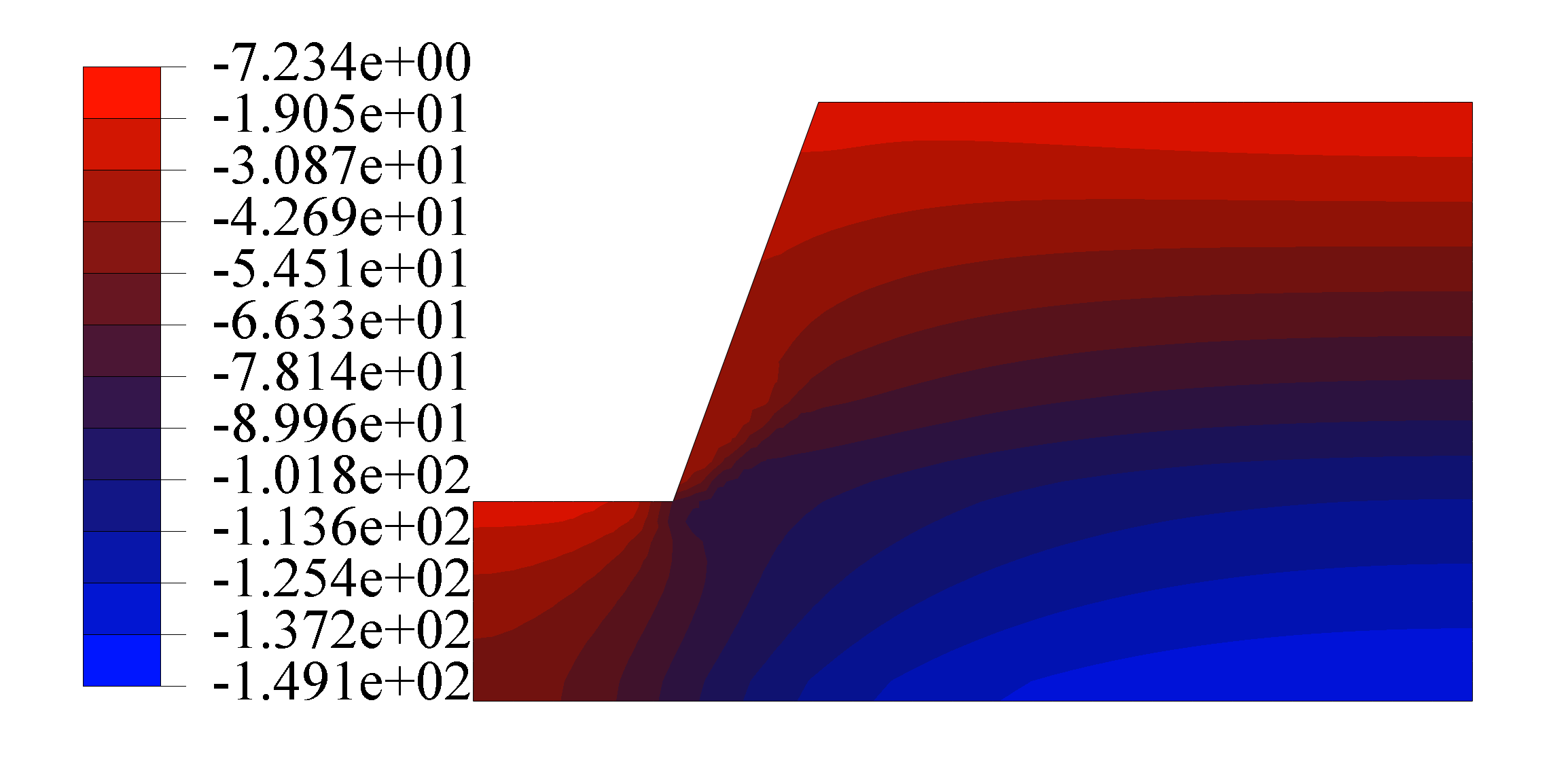

Supplement: Supplementary file 1 [file sensors-26-00421-s001.zip › Supplementary Materials/S3370.png]

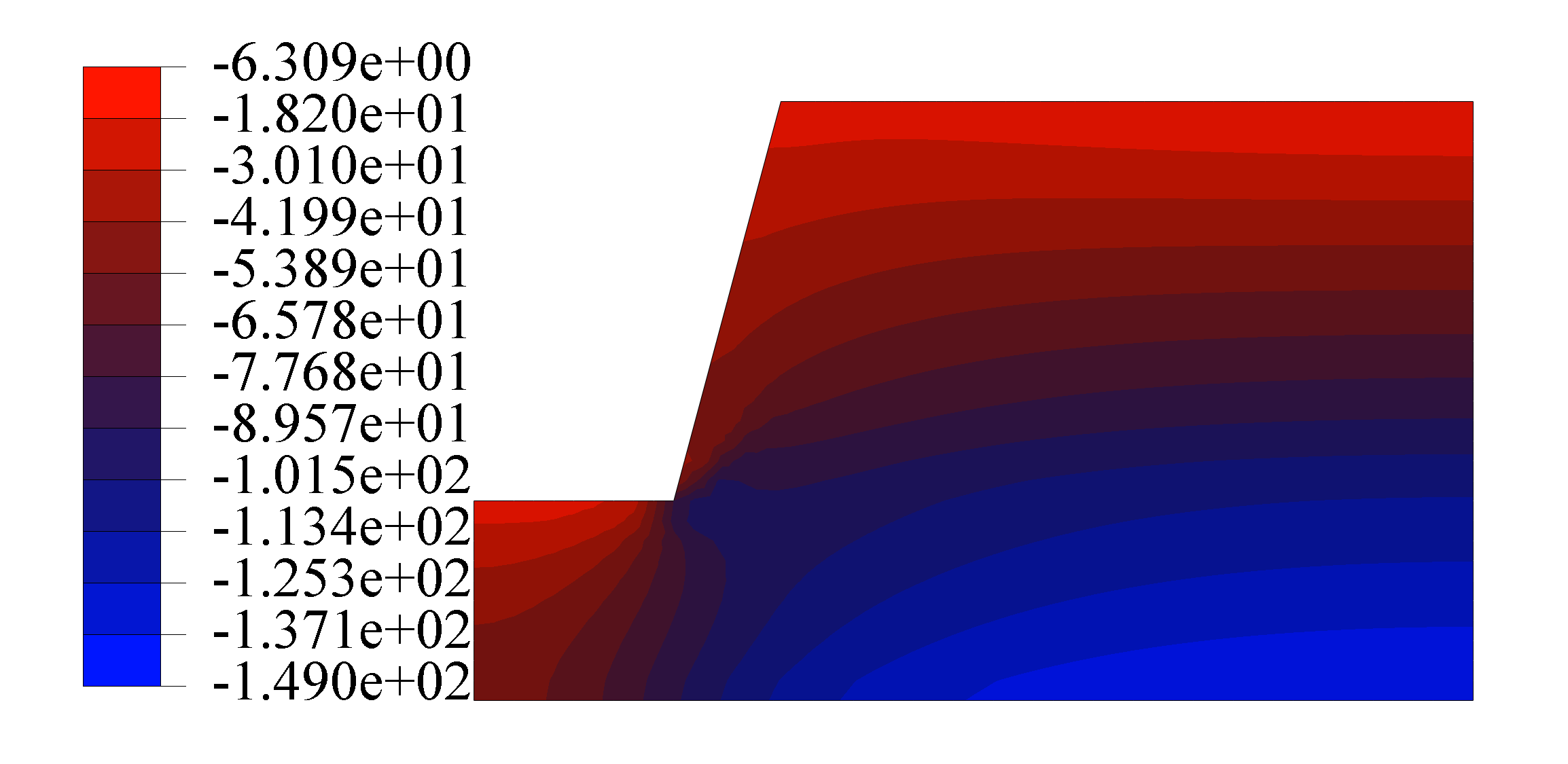

Supplement: Supplementary file 1 [file sensors-26-00421-s001.zip › Supplementary Materials/S3375.png]

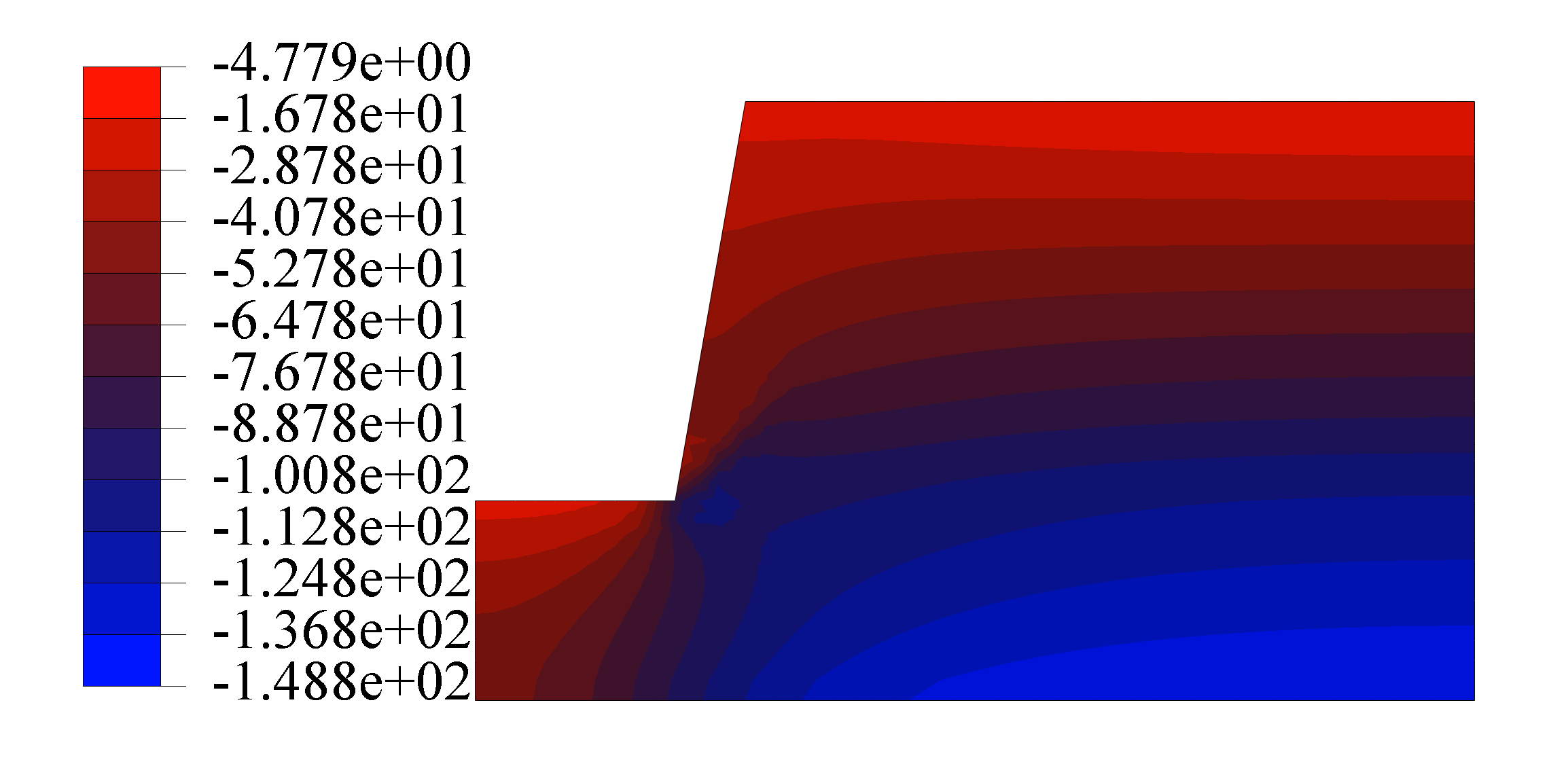

Supplement: Supplementary file 1 [file sensors-26-00421-s001.zip › Supplementary Materials/S3380.png]

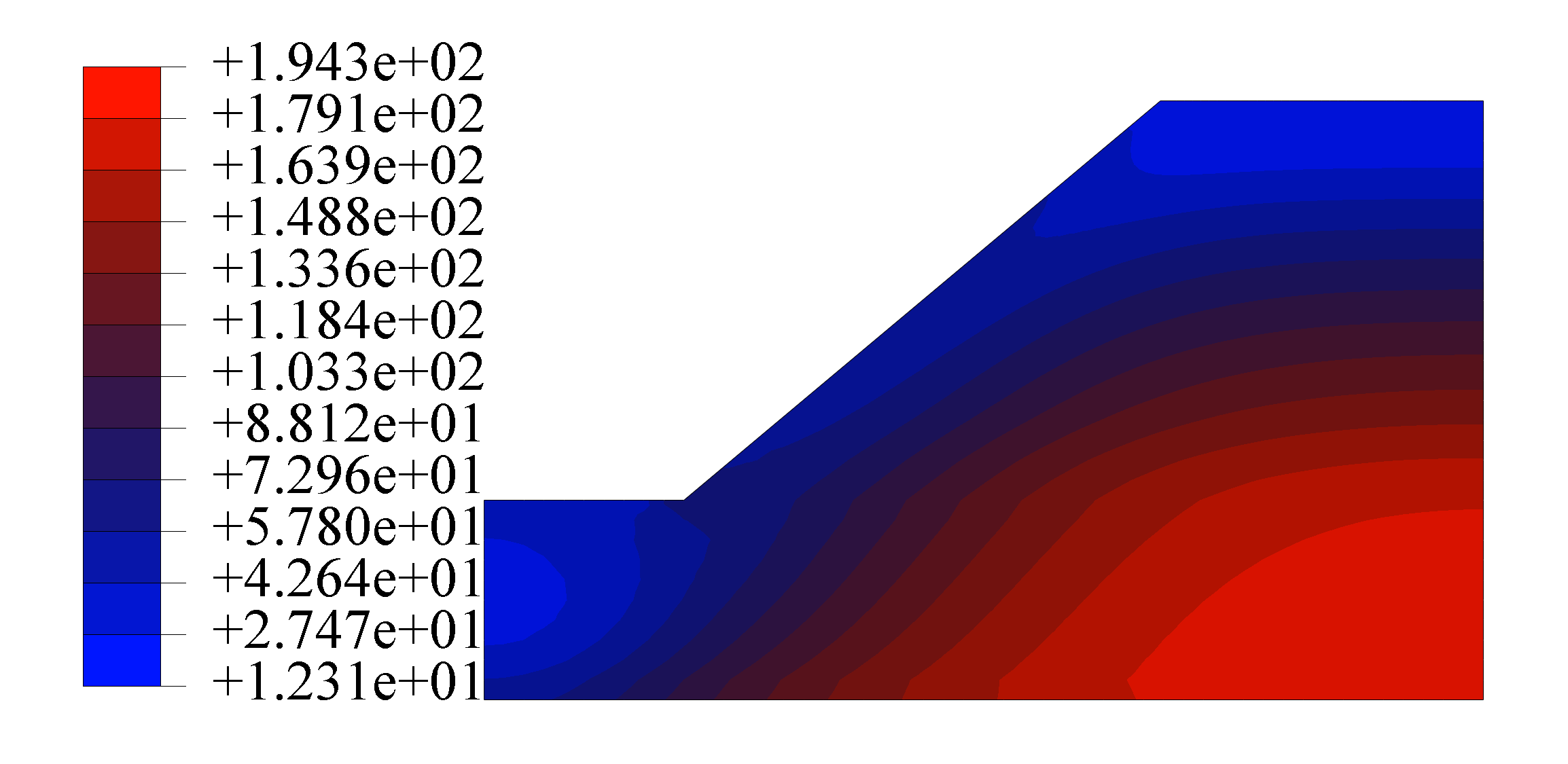

Supplement: Supplementary file 1 [file sensors-26-00421-s001.zip › Supplementary Materials/SM40.png]

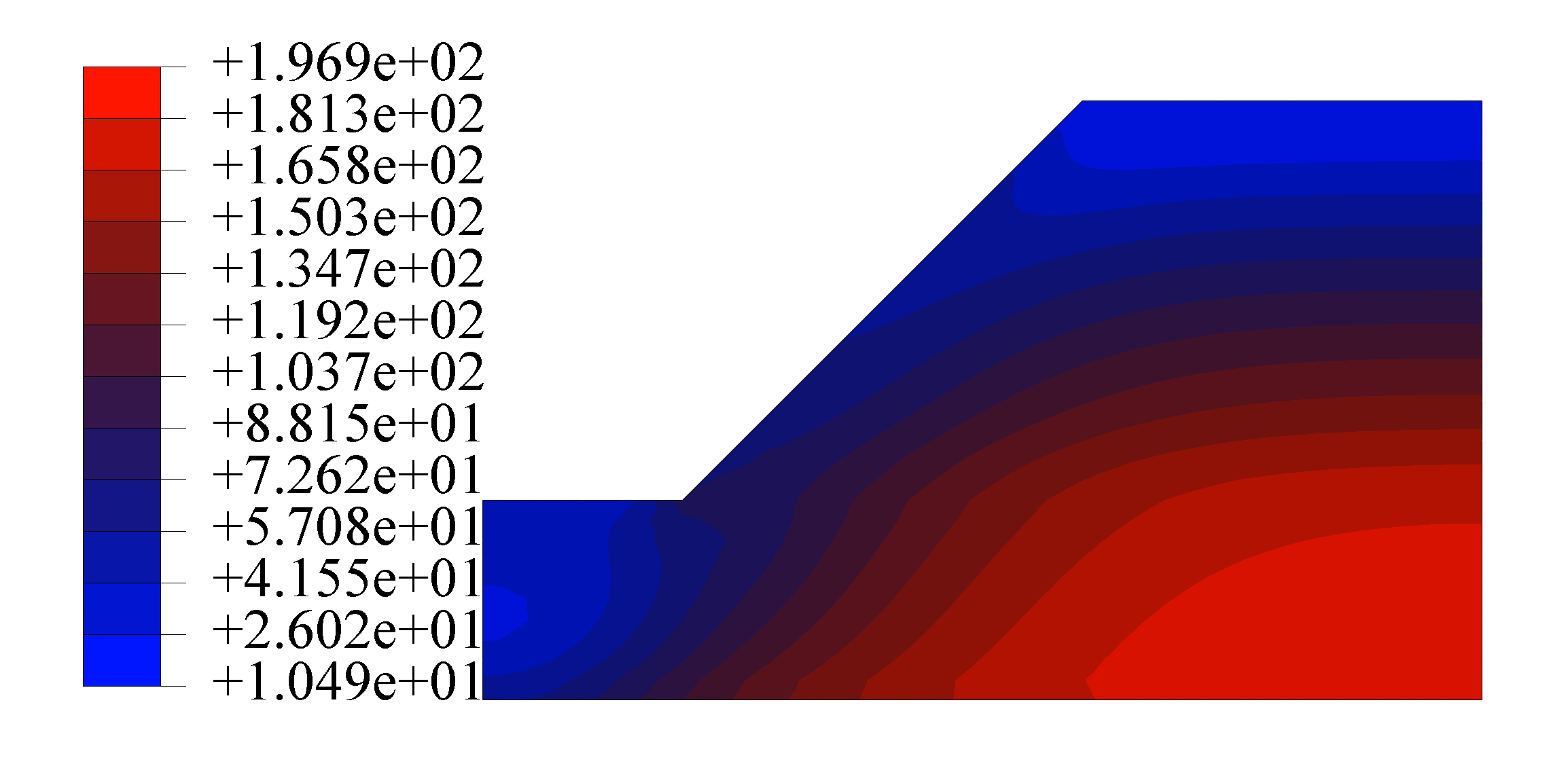

Supplement: Supplementary file 1 [file sensors-26-00421-s001.zip › Supplementary Materials/SM45.png]

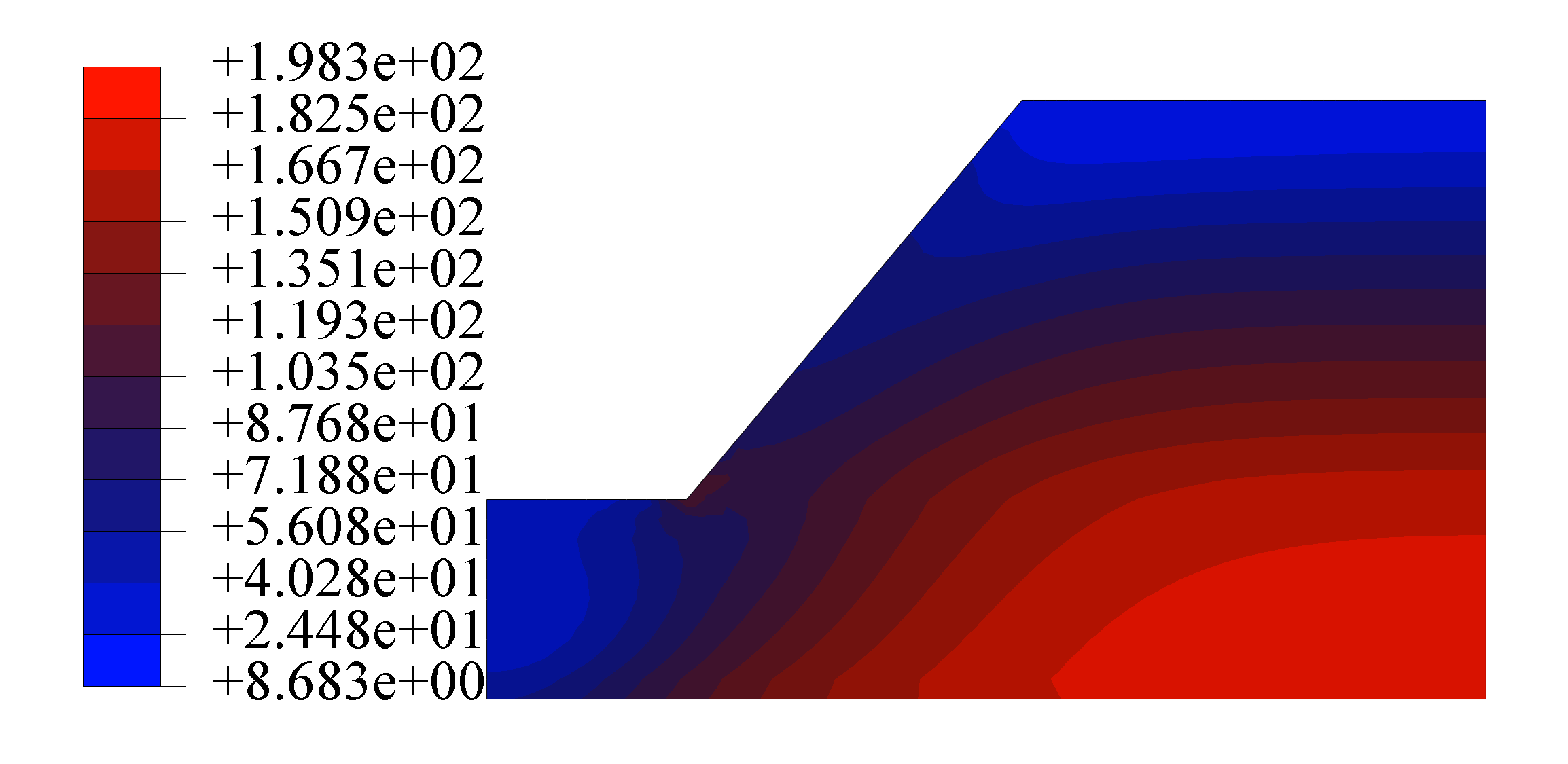

Supplement: Supplementary file 1 [file sensors-26-00421-s001.zip › Supplementary Materials/SM50.png]

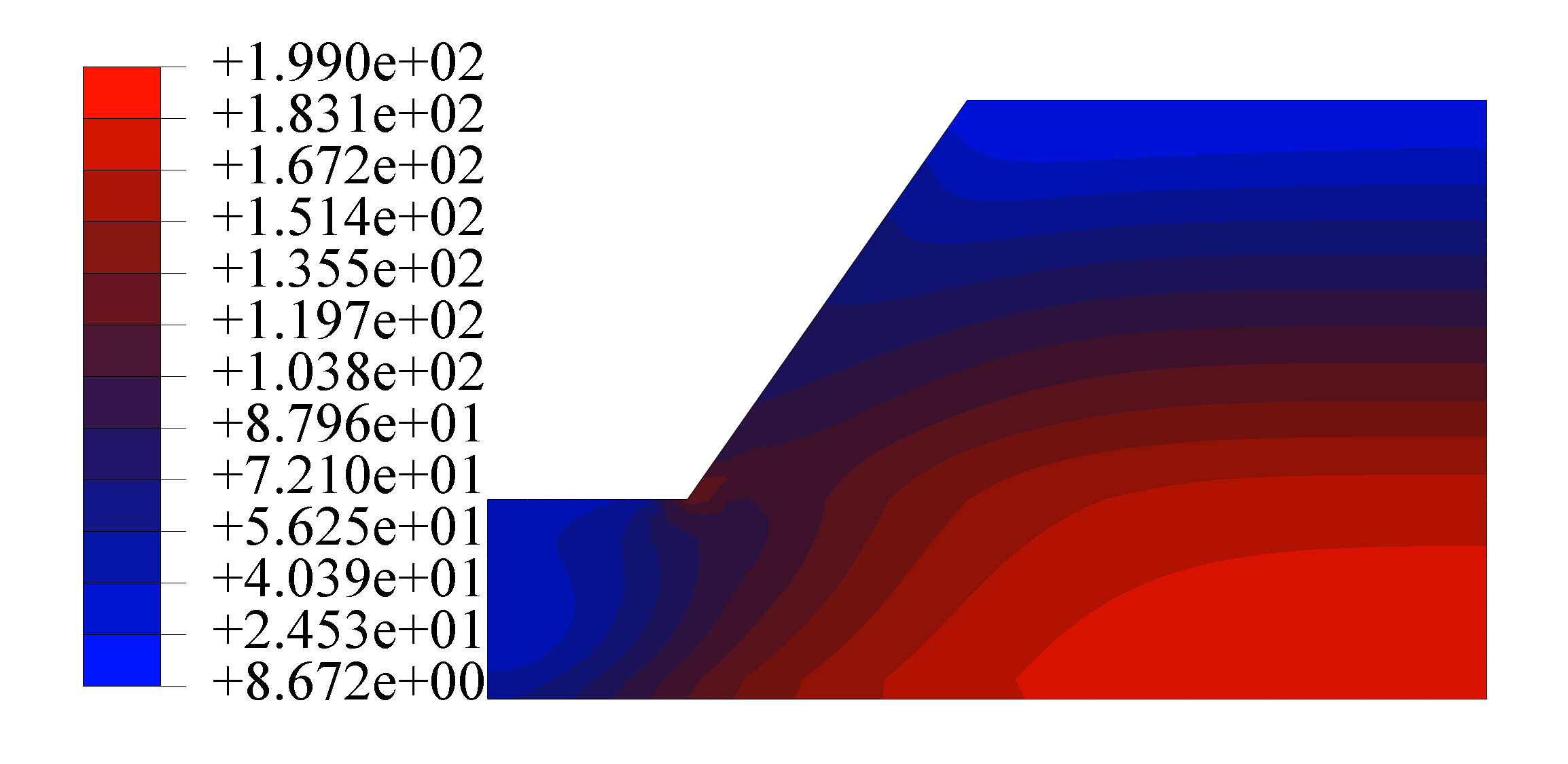

Supplement: Supplementary file 1 [file sensors-26-00421-s001.zip › Supplementary Materials/SM55.png]

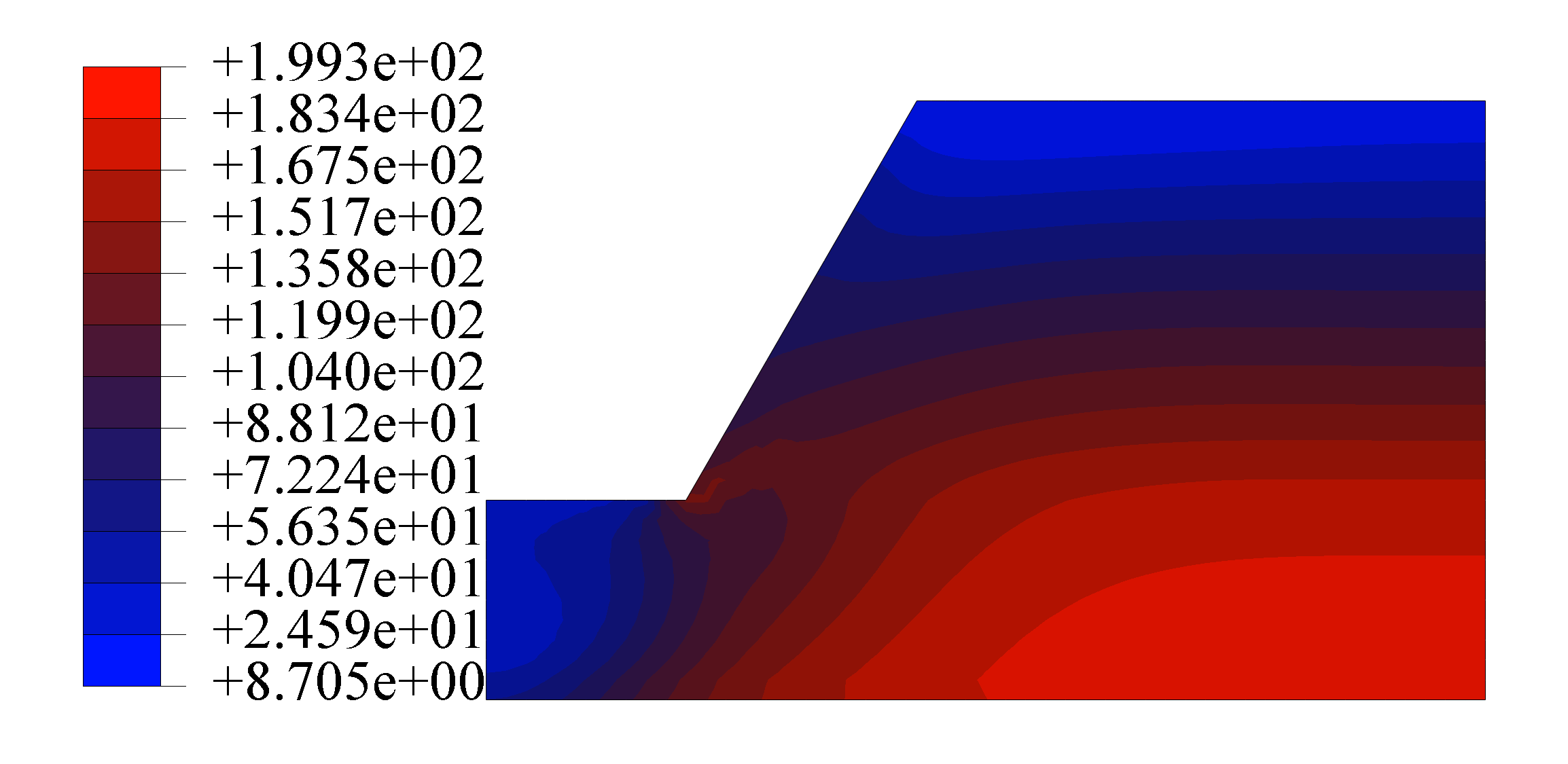

Supplement: Supplementary file 1 [file sensors-26-00421-s001.zip › Supplementary Materials/SM60.png]

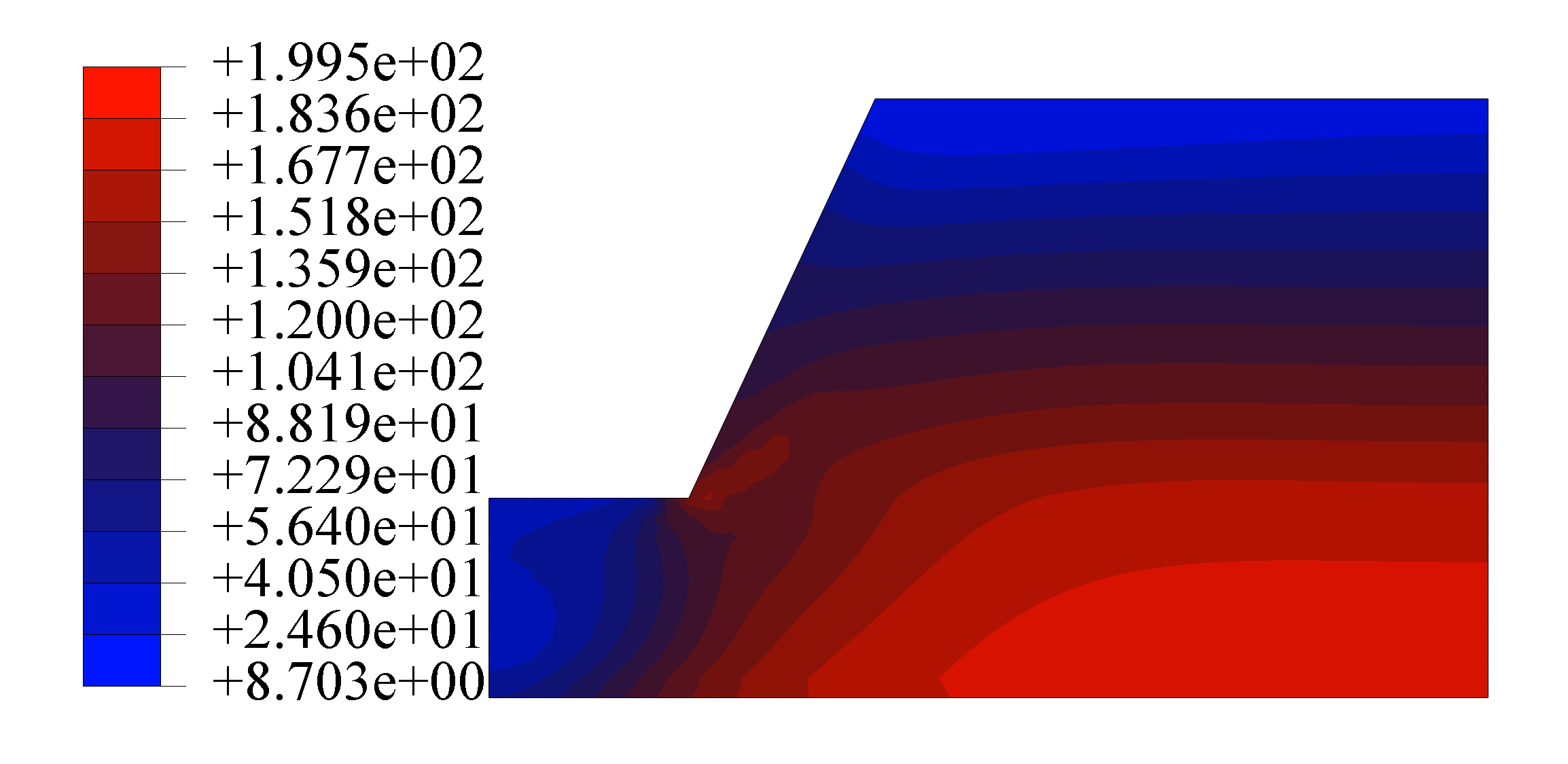

Supplement: Supplementary file 1 [file sensors-26-00421-s001.zip › Supplementary Materials/SM65.png]

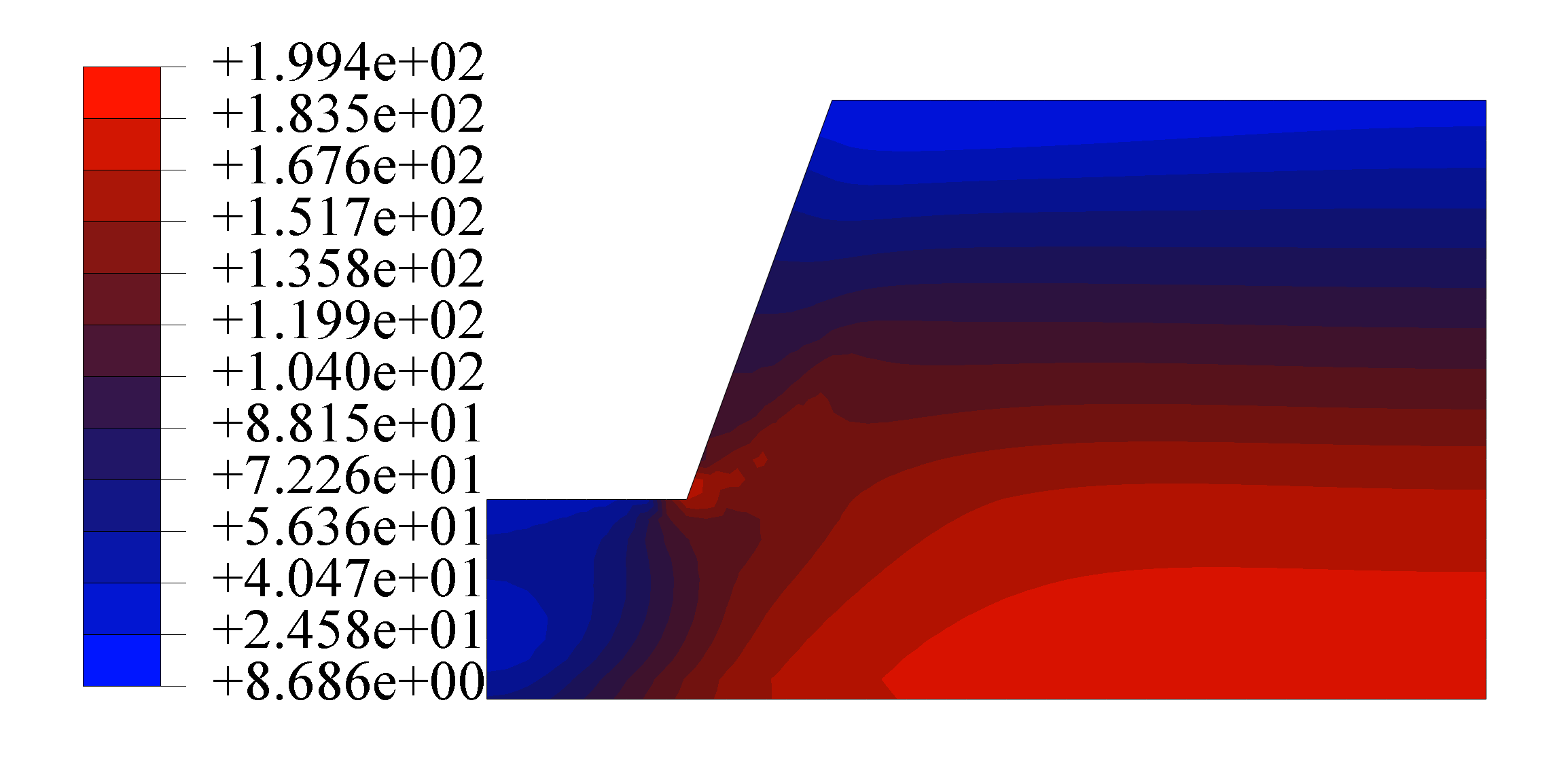

Supplement: Supplementary file 1 [file sensors-26-00421-s001.zip › Supplementary Materials/SM70.png]

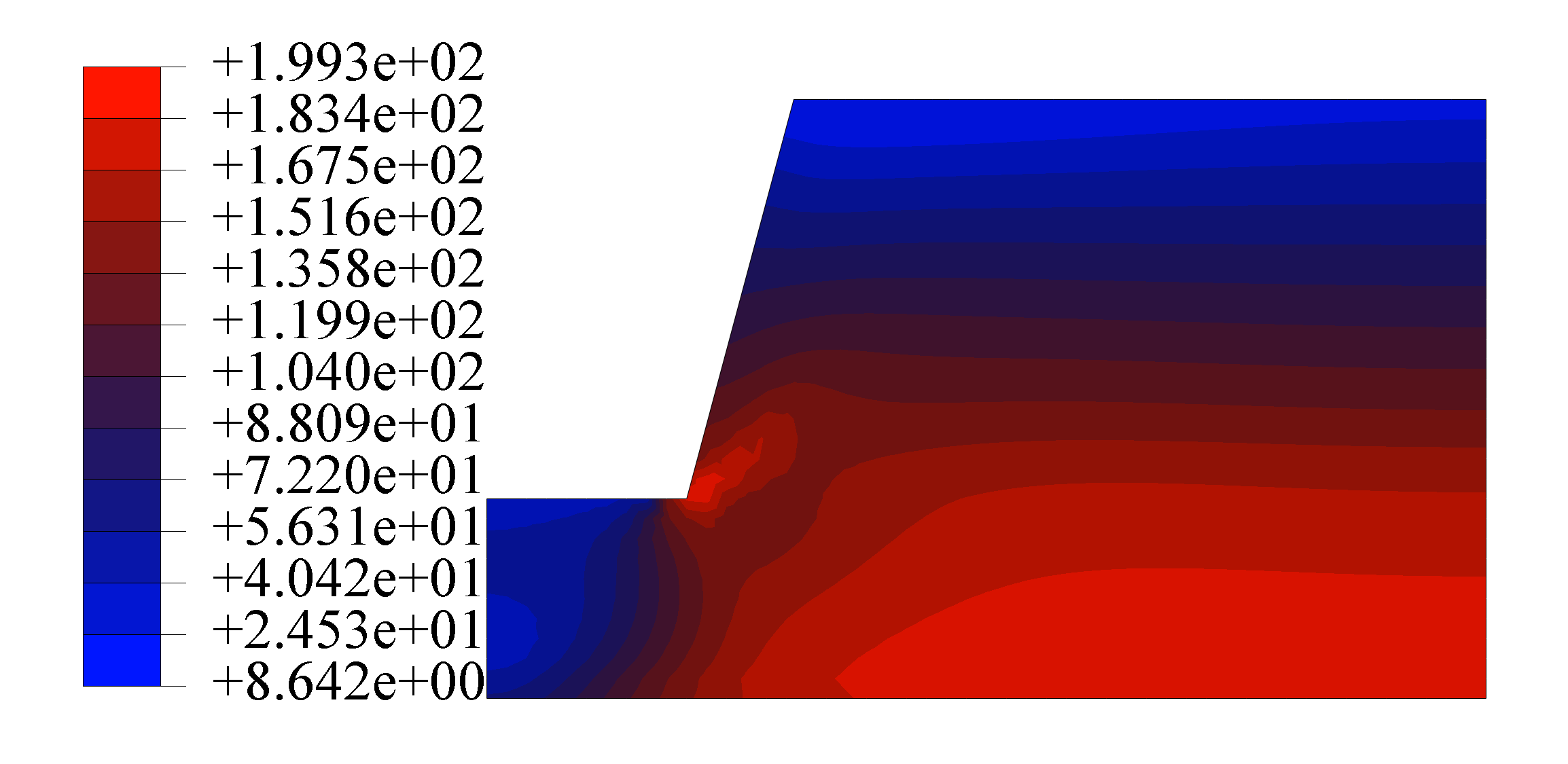

Supplement: Supplementary file 1 [file sensors-26-00421-s001.zip › Supplementary Materials/SM75.png]

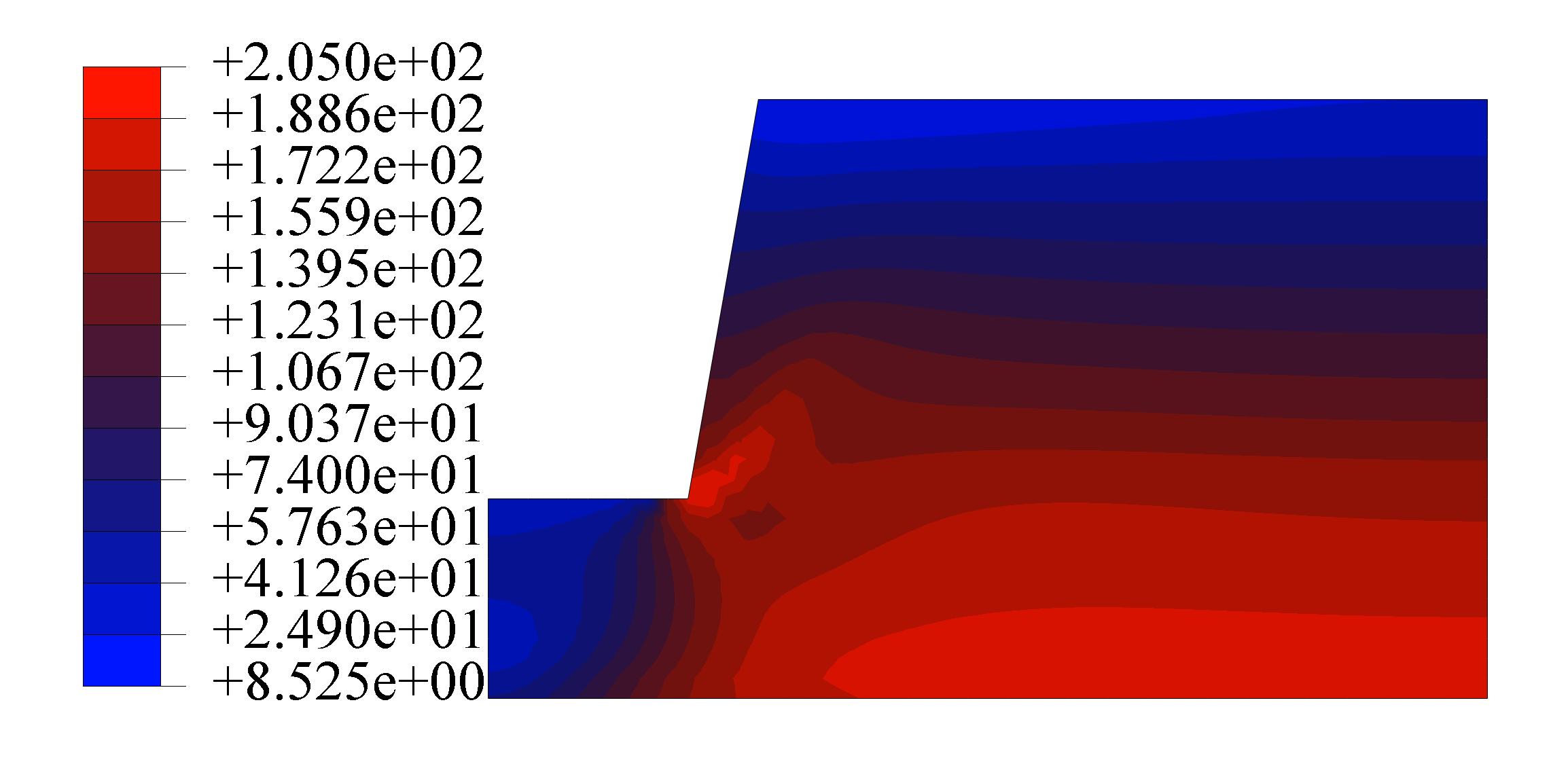

Supplement: Supplementary file 1 [file sensors-26-00421-s001.zip › Supplementary Materials/SM80.png]

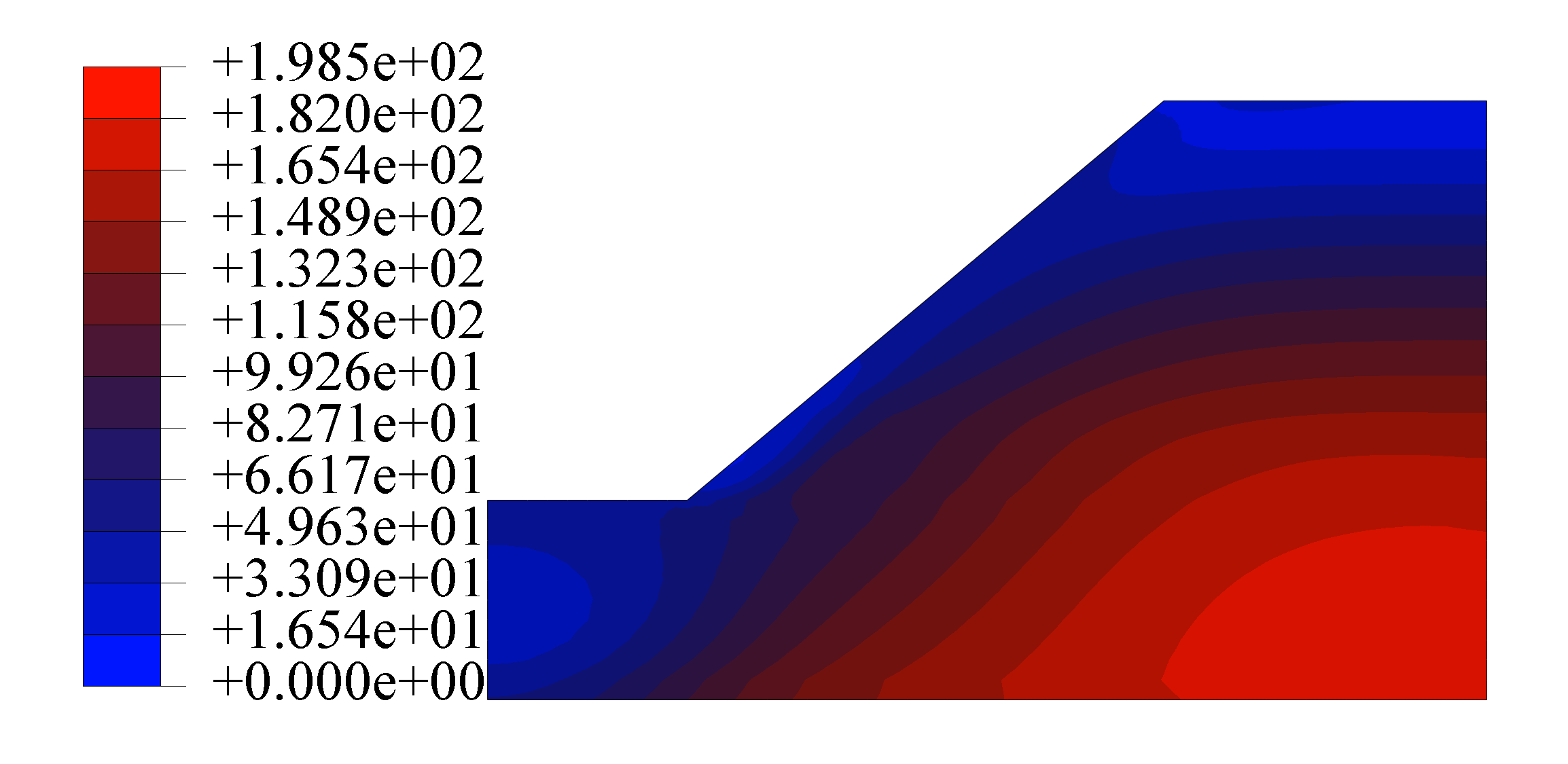

Supplement: Supplementary file 1 [file sensors-26-00421-s001.zip › Supplementary Materials/SMH40.png]

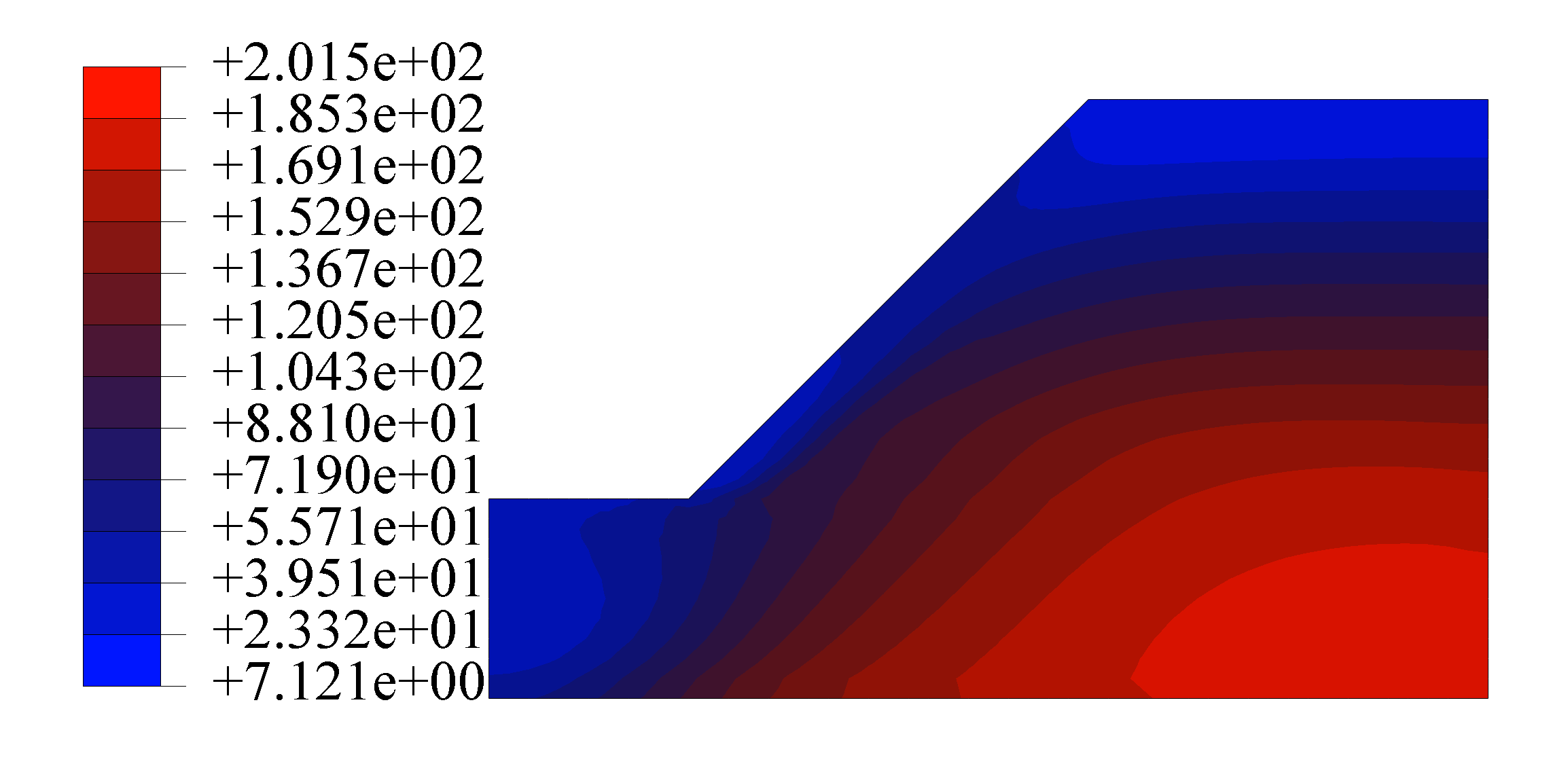

Supplement: Supplementary file 1 [file sensors-26-00421-s001.zip › Supplementary Materials/SMH45.png]

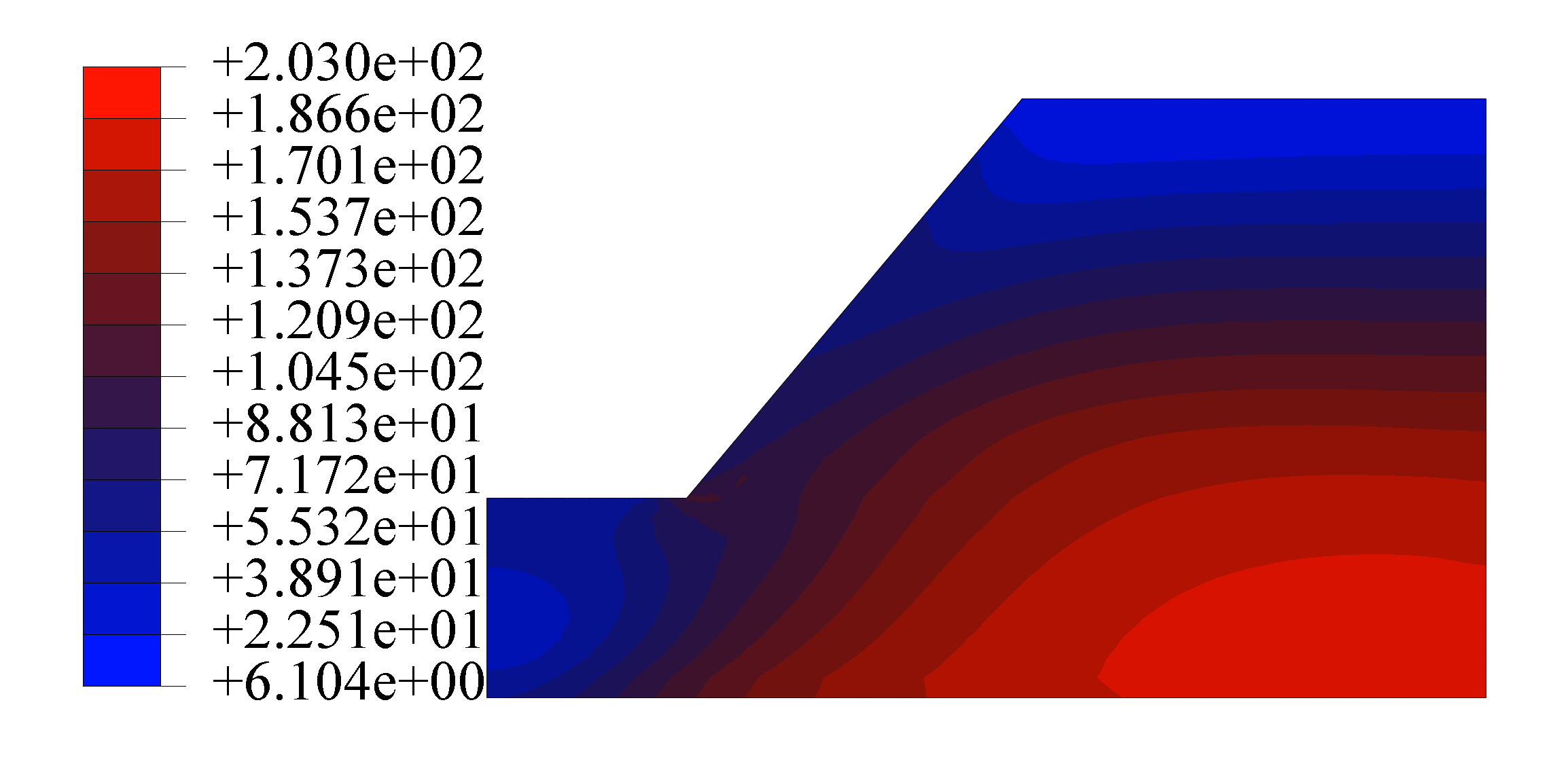

Supplement: Supplementary file 1 [file sensors-26-00421-s001.zip › Supplementary Materials/SMH50.png]

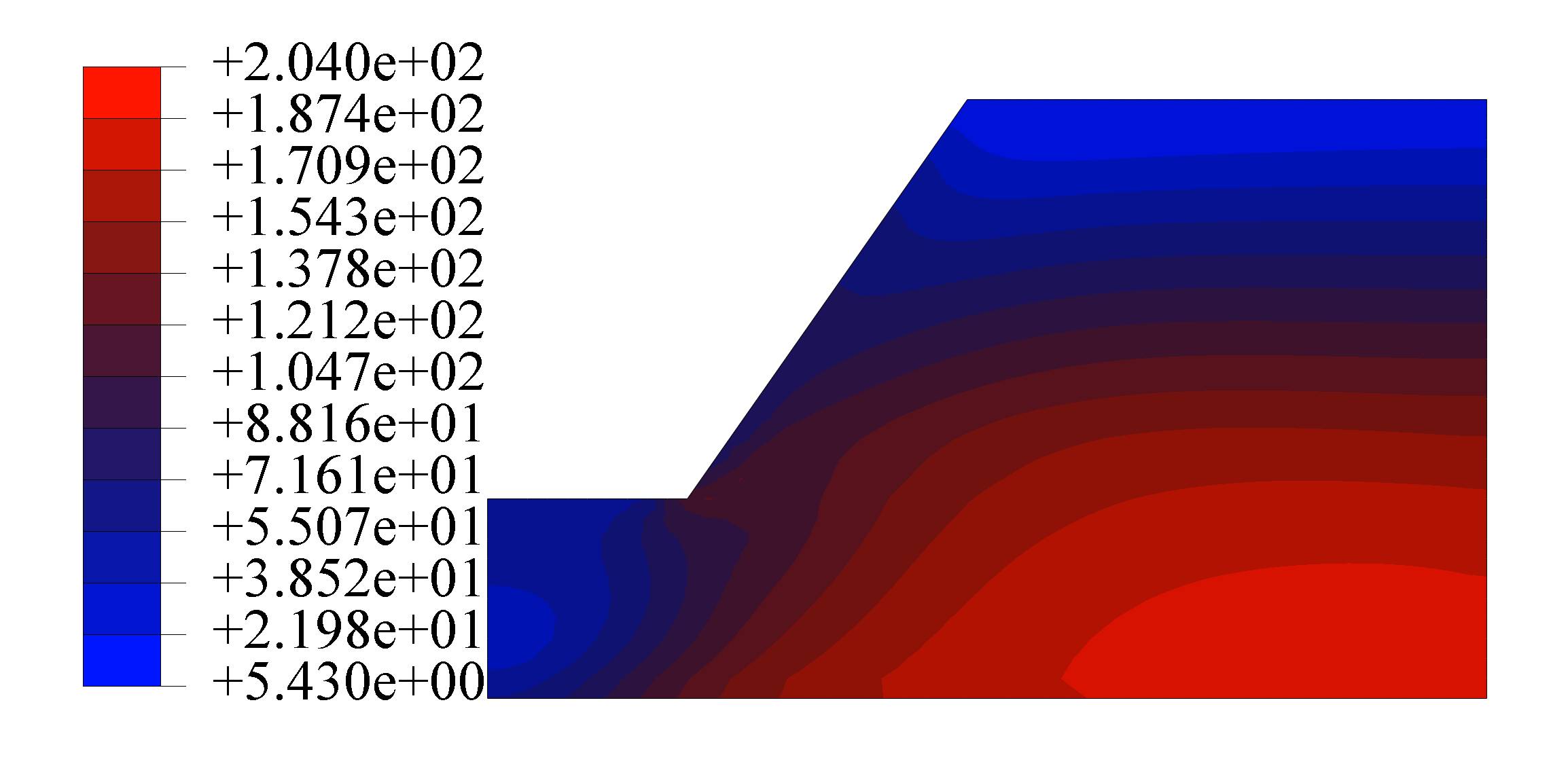

Supplement: Supplementary file 1 [file sensors-26-00421-s001.zip › Supplementary Materials/SMH55.png]

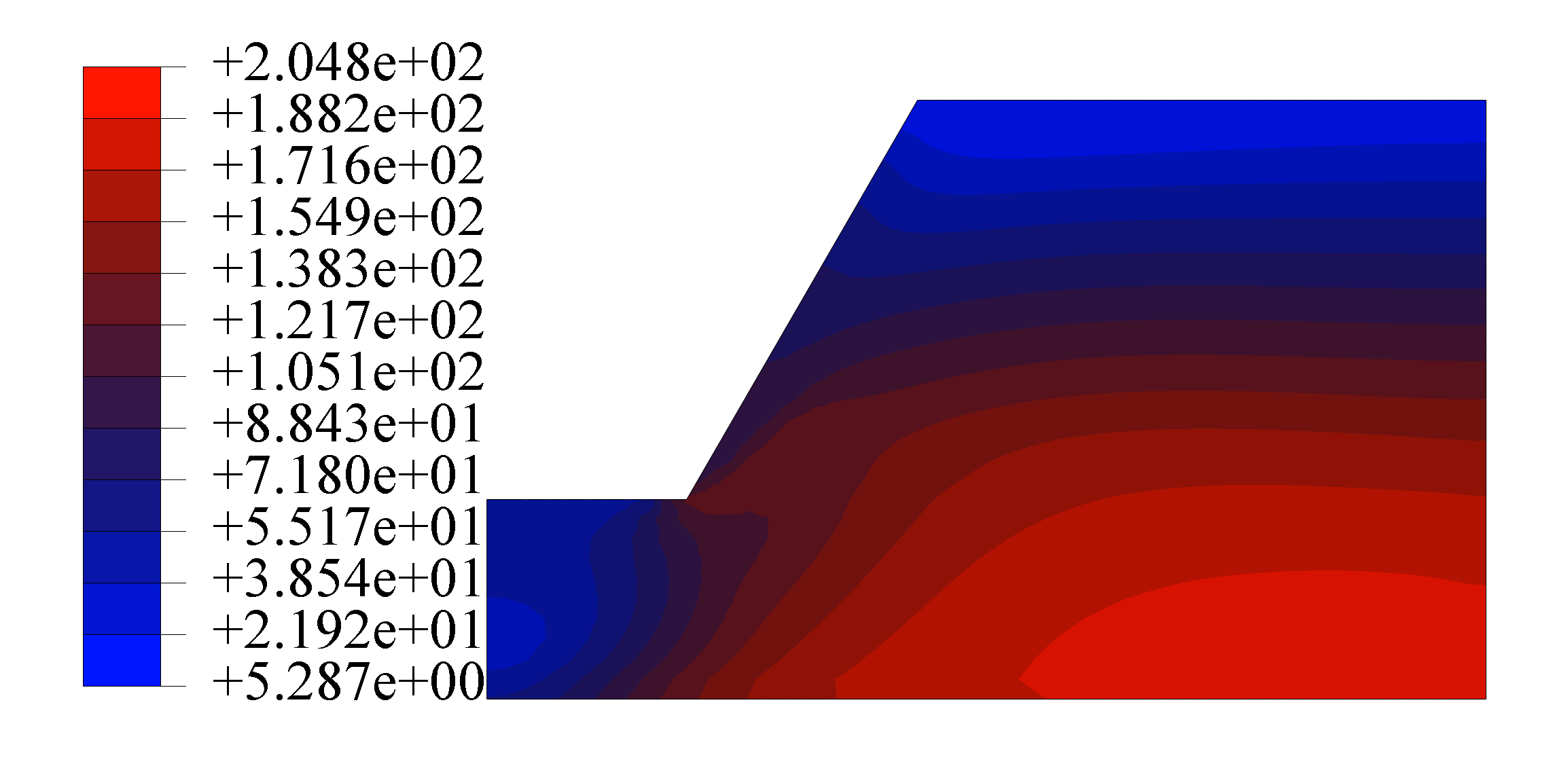

Supplement: Supplementary file 1 [file sensors-26-00421-s001.zip › Supplementary Materials/SMH60.png]

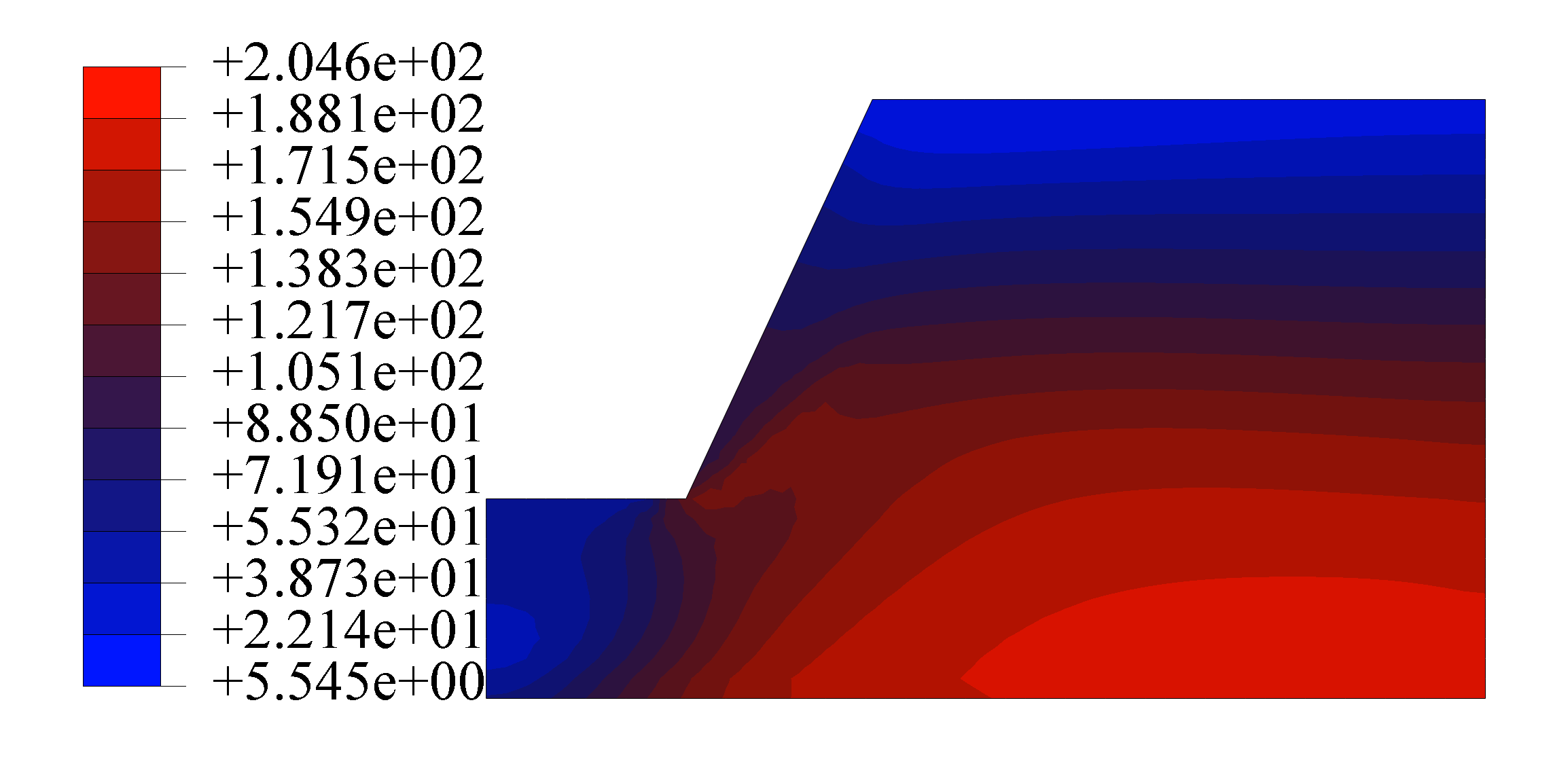

Supplement: Supplementary file 1 [file sensors-26-00421-s001.zip › Supplementary Materials/SMH65.png]

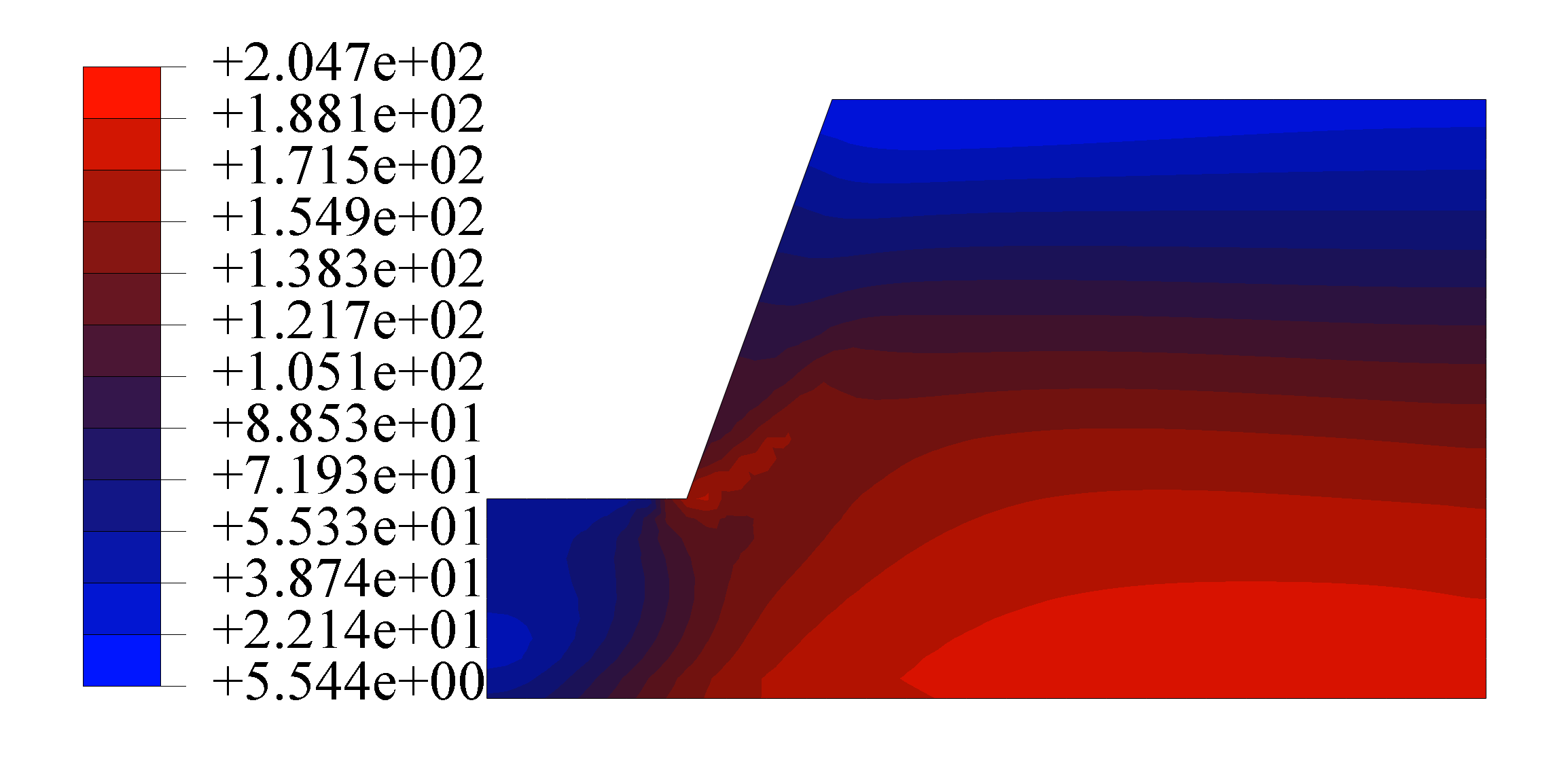

Supplement: Supplementary file 1 [file sensors-26-00421-s001.zip › Supplementary Materials/SMH70.png]

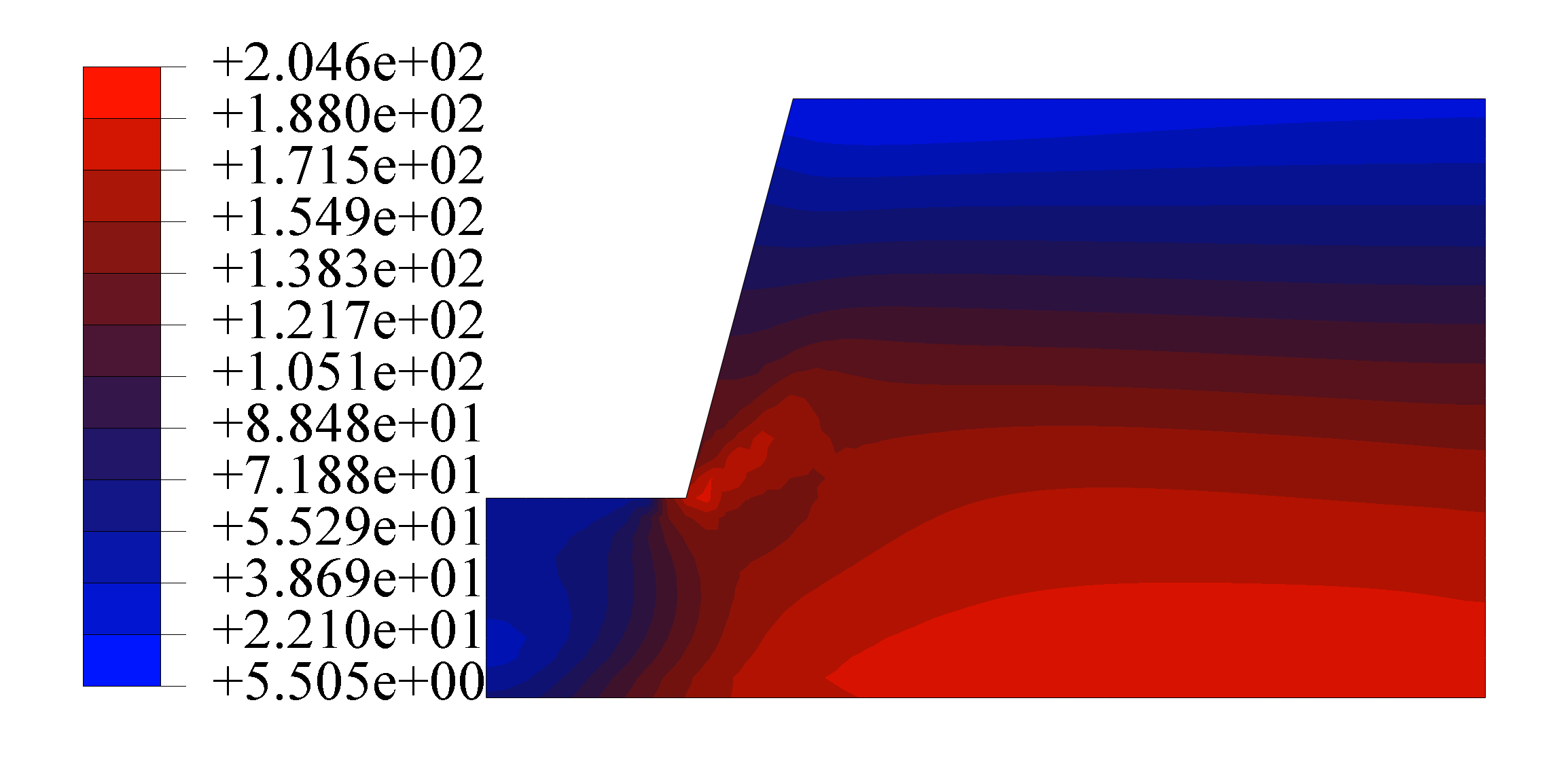

Supplement: Supplementary file 1 [file sensors-26-00421-s001.zip › Supplementary Materials/SMH75.png]

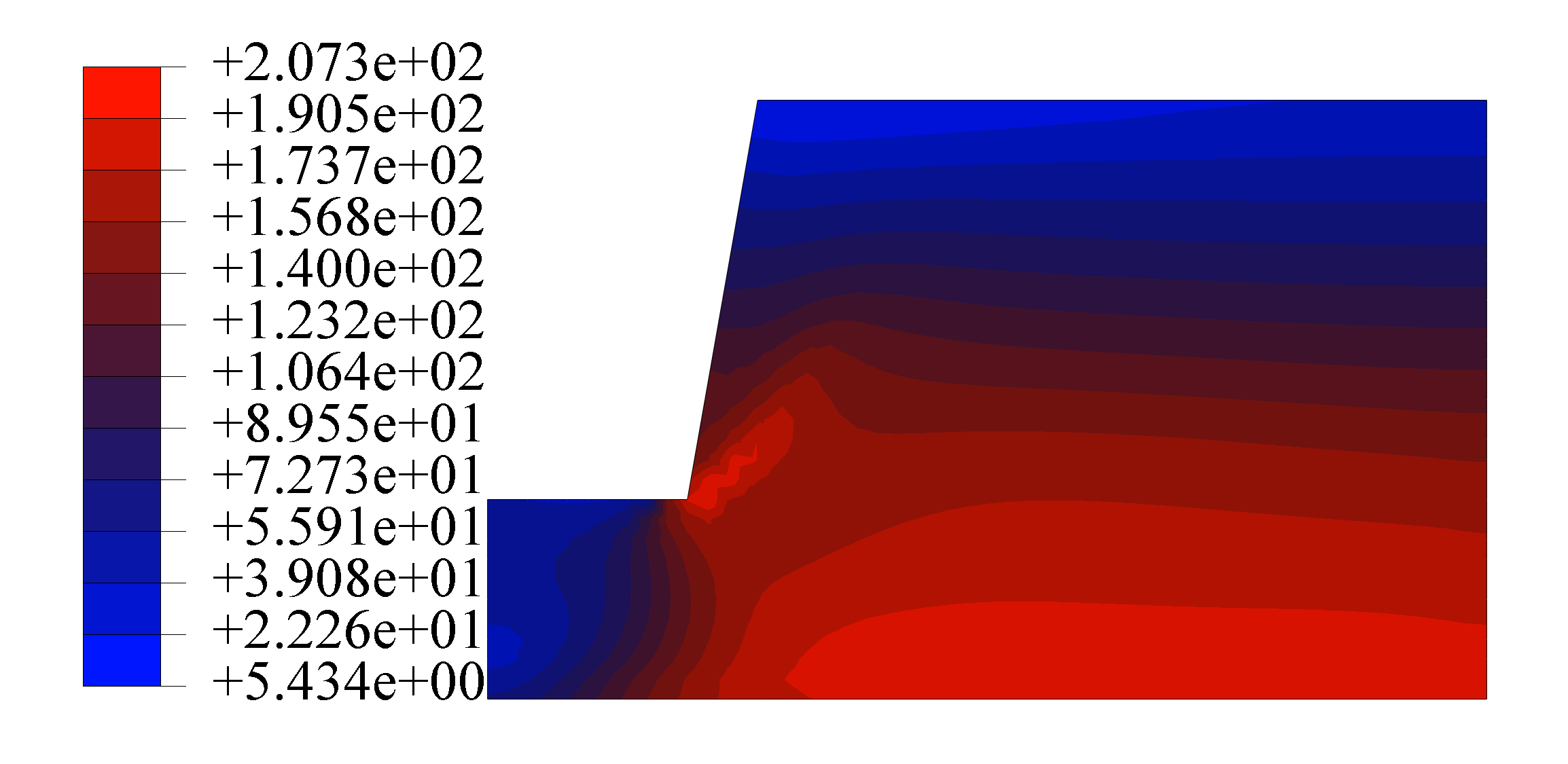

Supplement: Supplementary file 1 [file sensors-26-00421-s001.zip › Supplementary Materials/SMH80.png]

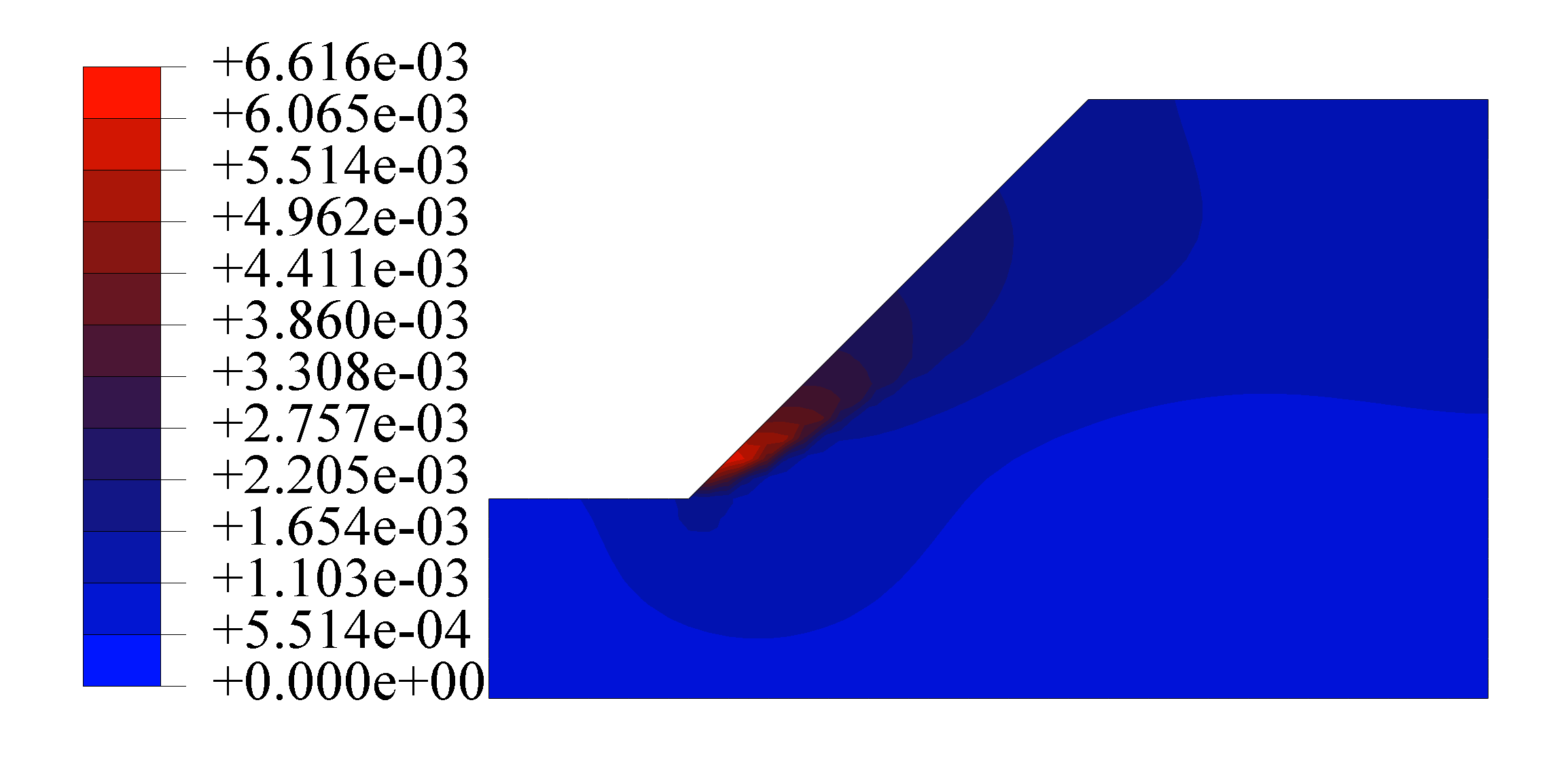

Supplement: Supplementary file 1 [file sensors-26-00421-s001.zip › Supplementary Materials/U45.png]

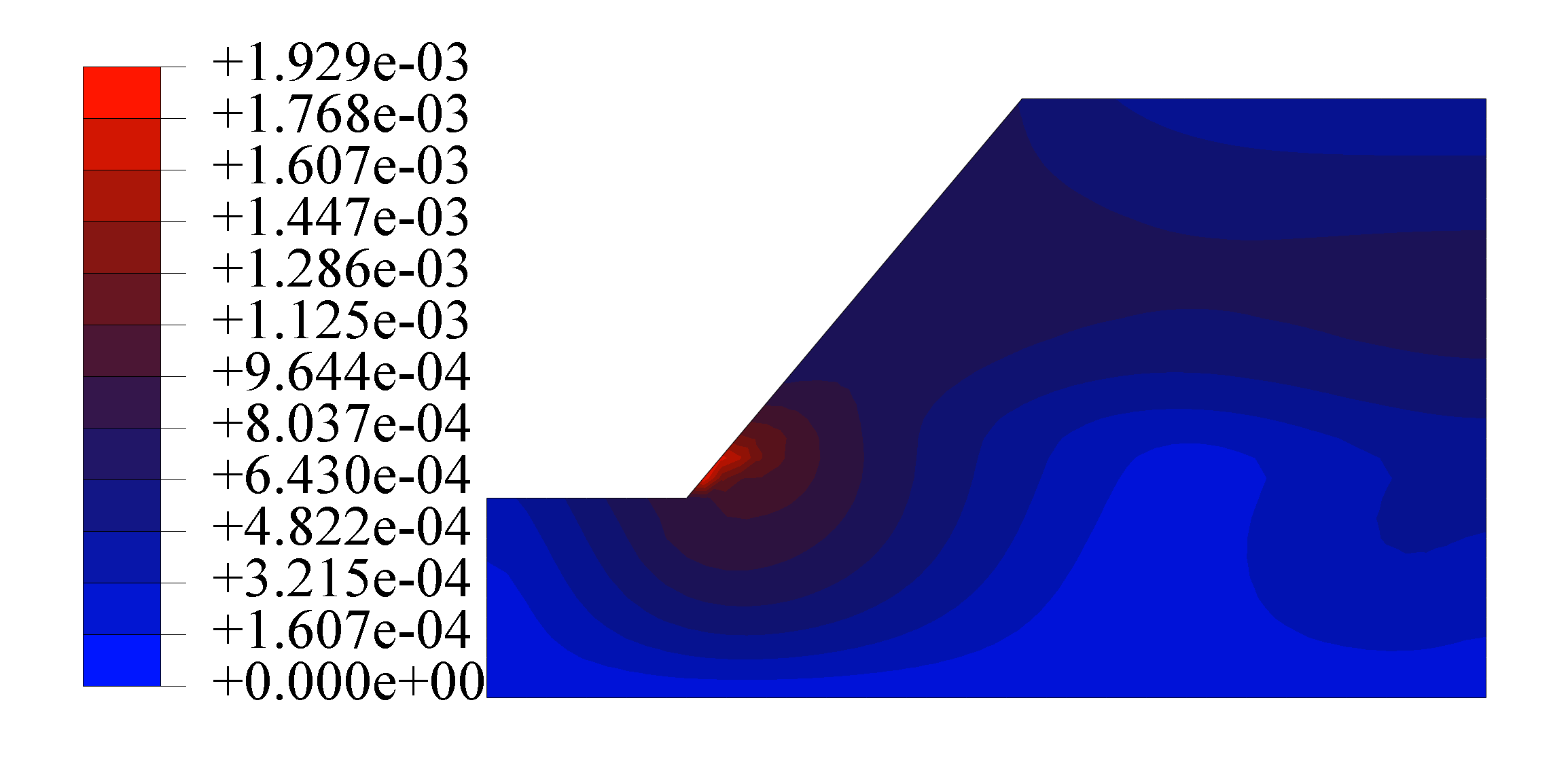

Supplement: Supplementary file 1 [file sensors-26-00421-s001.zip › Supplementary Materials/U50.png]

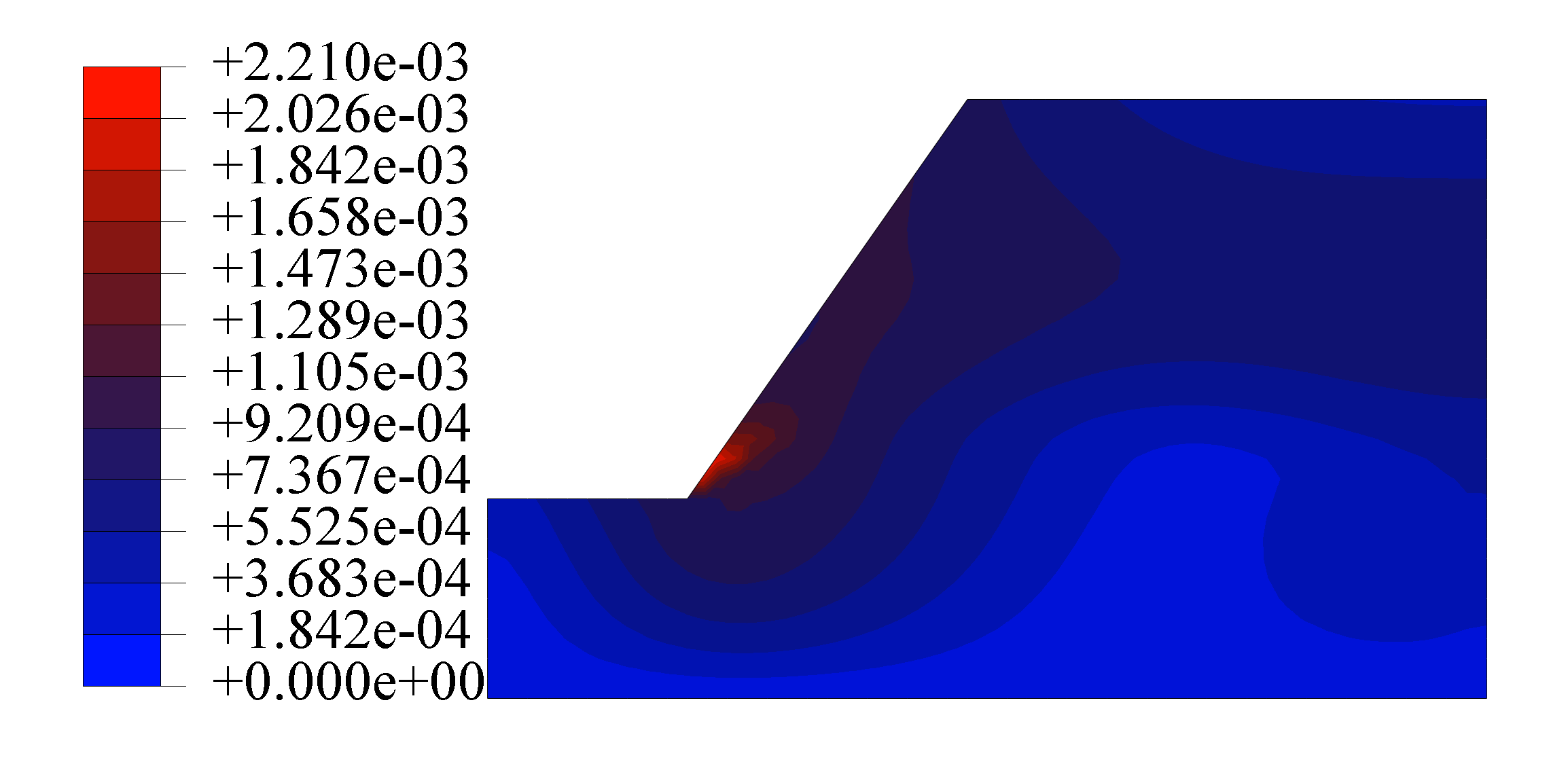

Supplement: Supplementary file 1 [file sensors-26-00421-s001.zip › Supplementary Materials/U55.png]

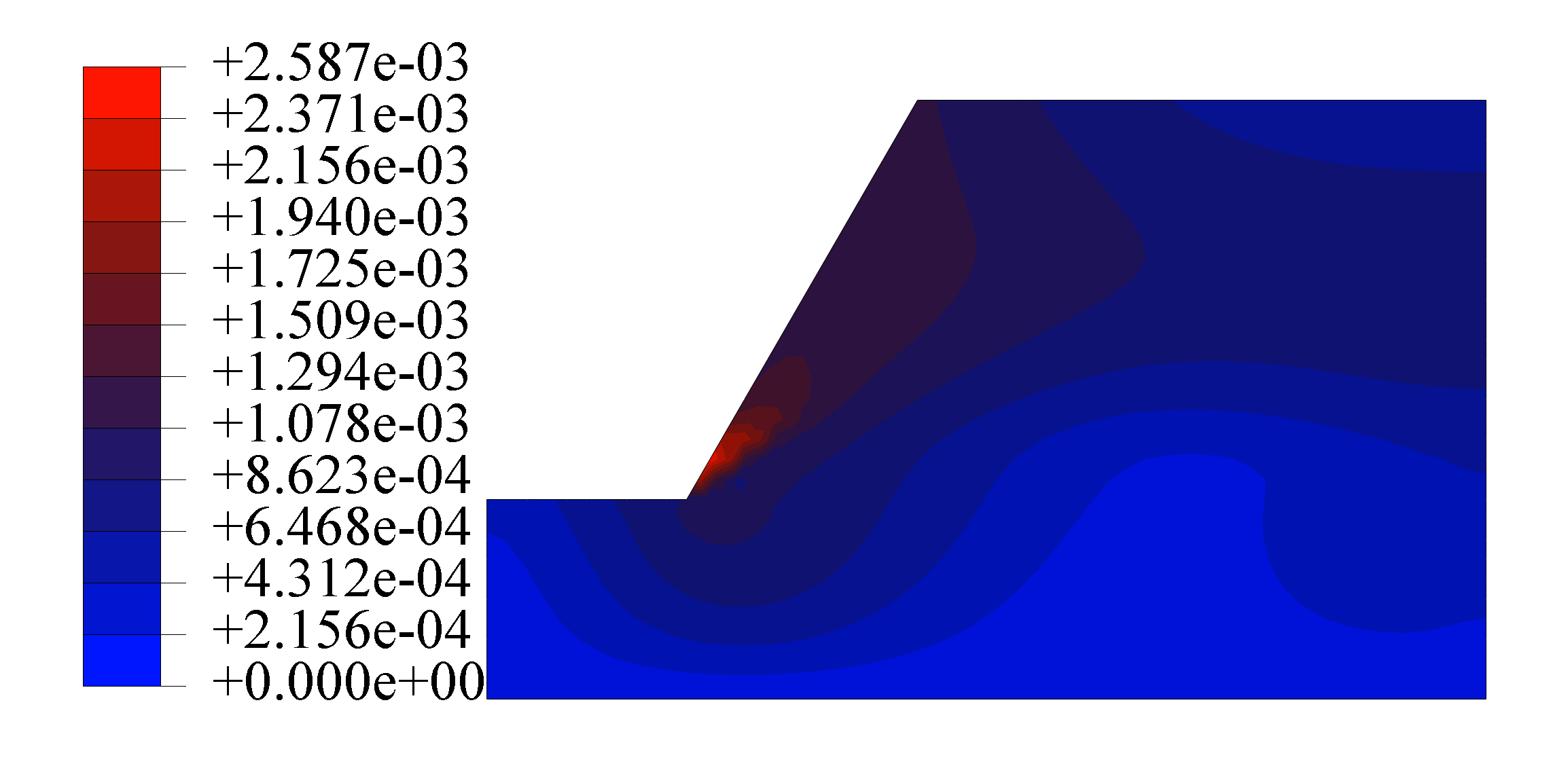

Supplement: Supplementary file 1 [file sensors-26-00421-s001.zip › Supplementary Materials/U60.png]

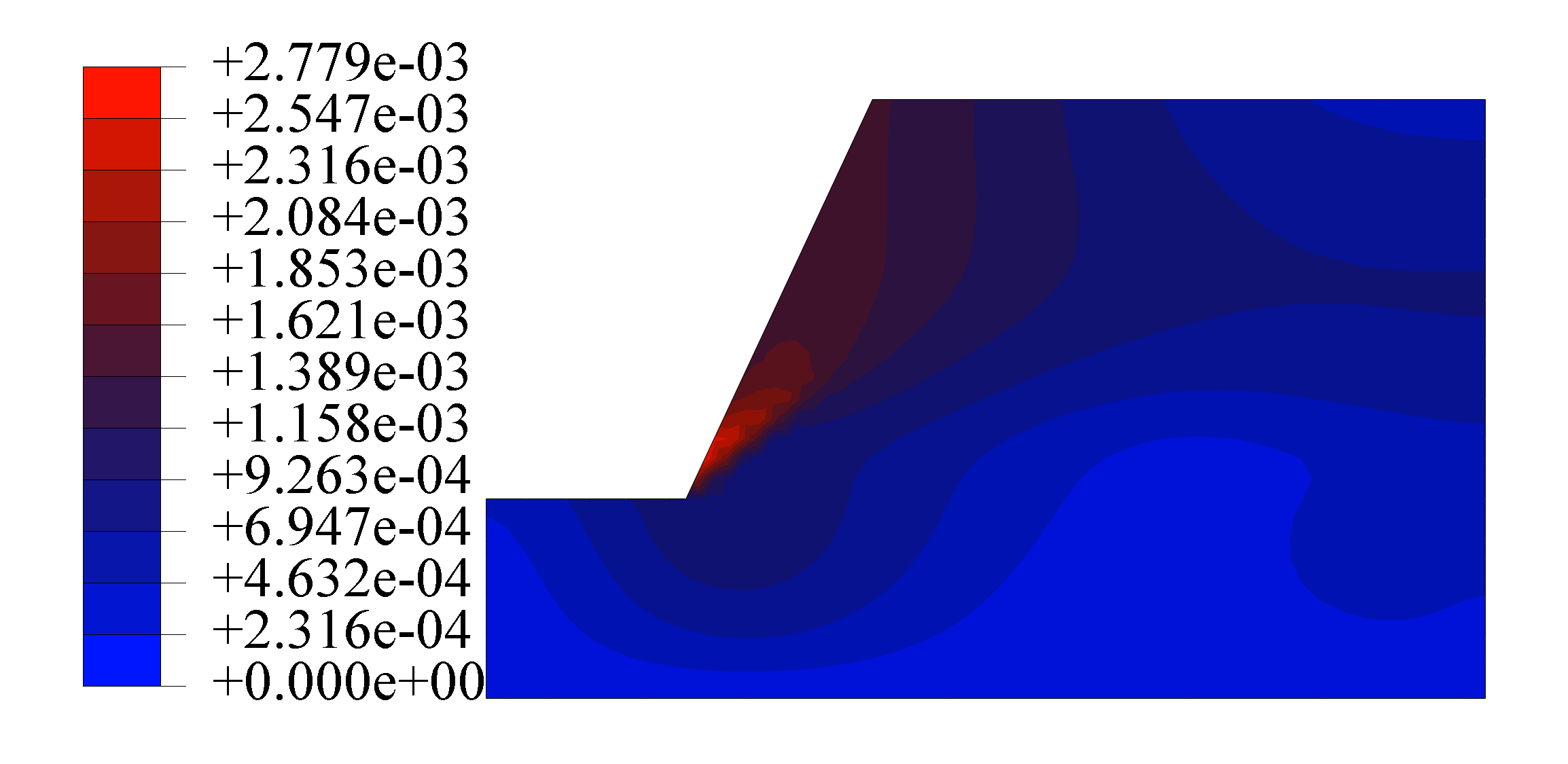

Supplement: Supplementary file 1 [file sensors-26-00421-s001.zip › Supplementary Materials/U65.png]

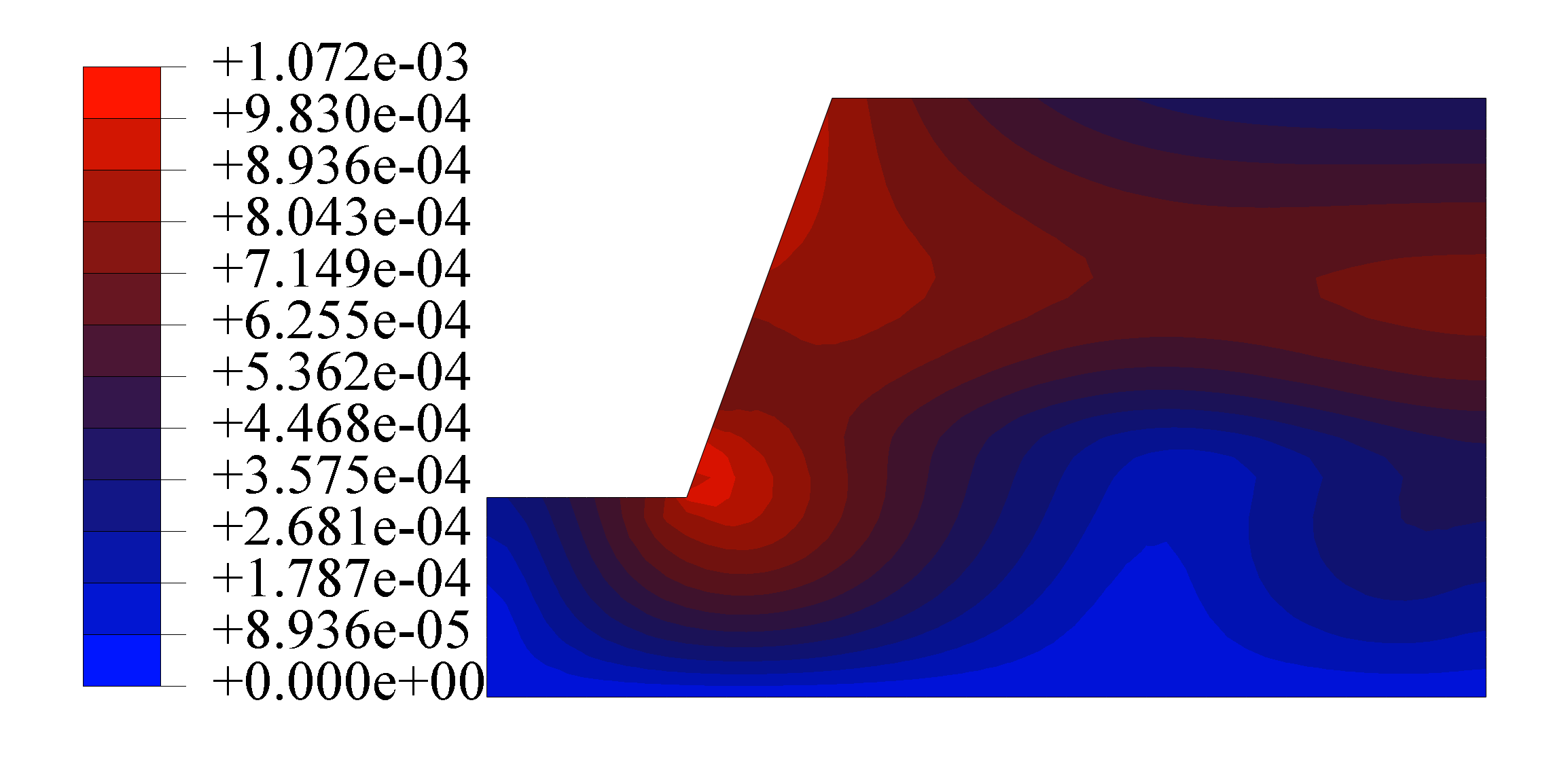

Supplement: Supplementary file 1 [file sensors-26-00421-s001.zip › Supplementary Materials/U70.png]

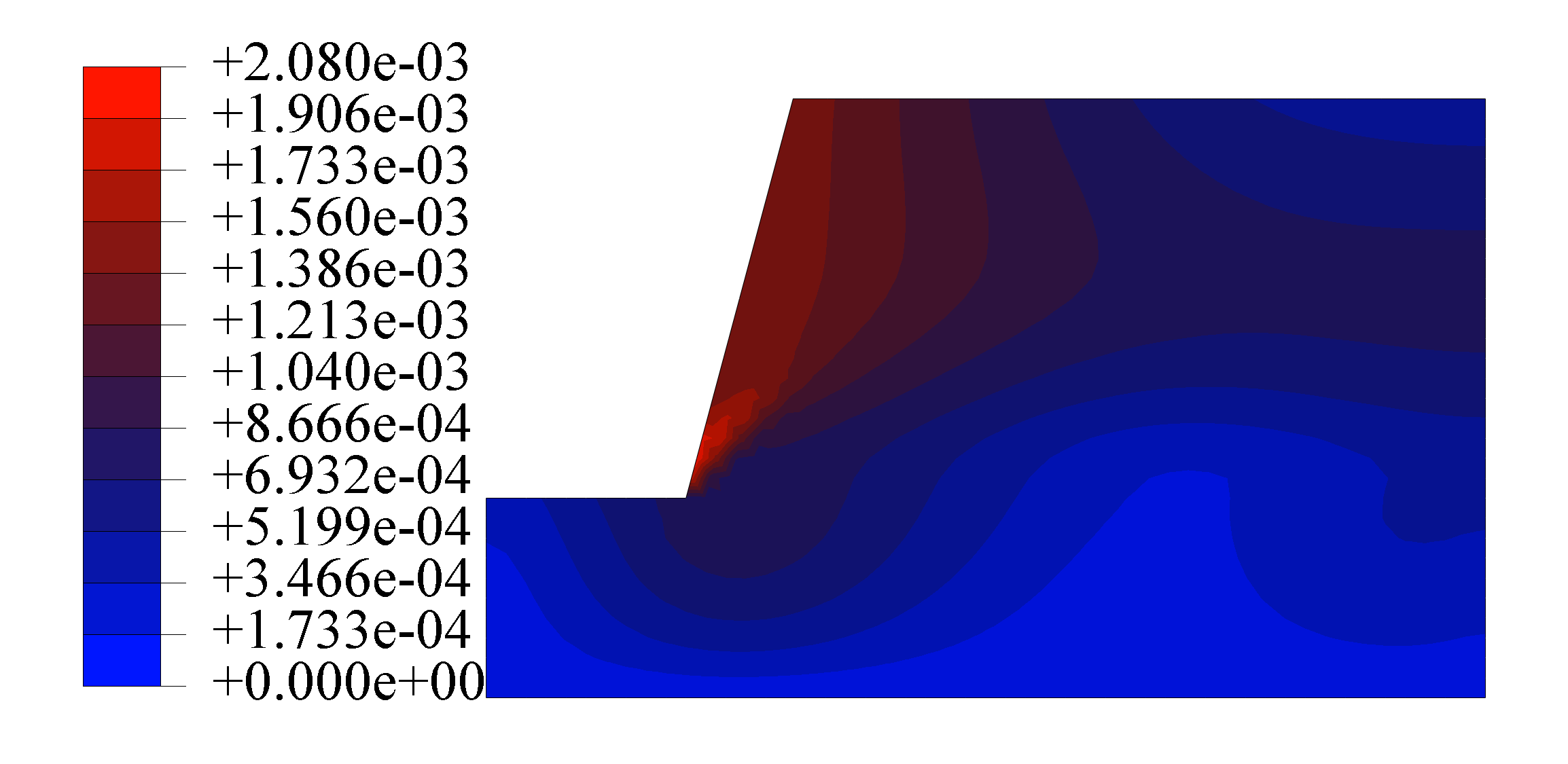

Supplement: Supplementary file 1 [file sensors-26-00421-s001.zip › Supplementary Materials/U75.png]

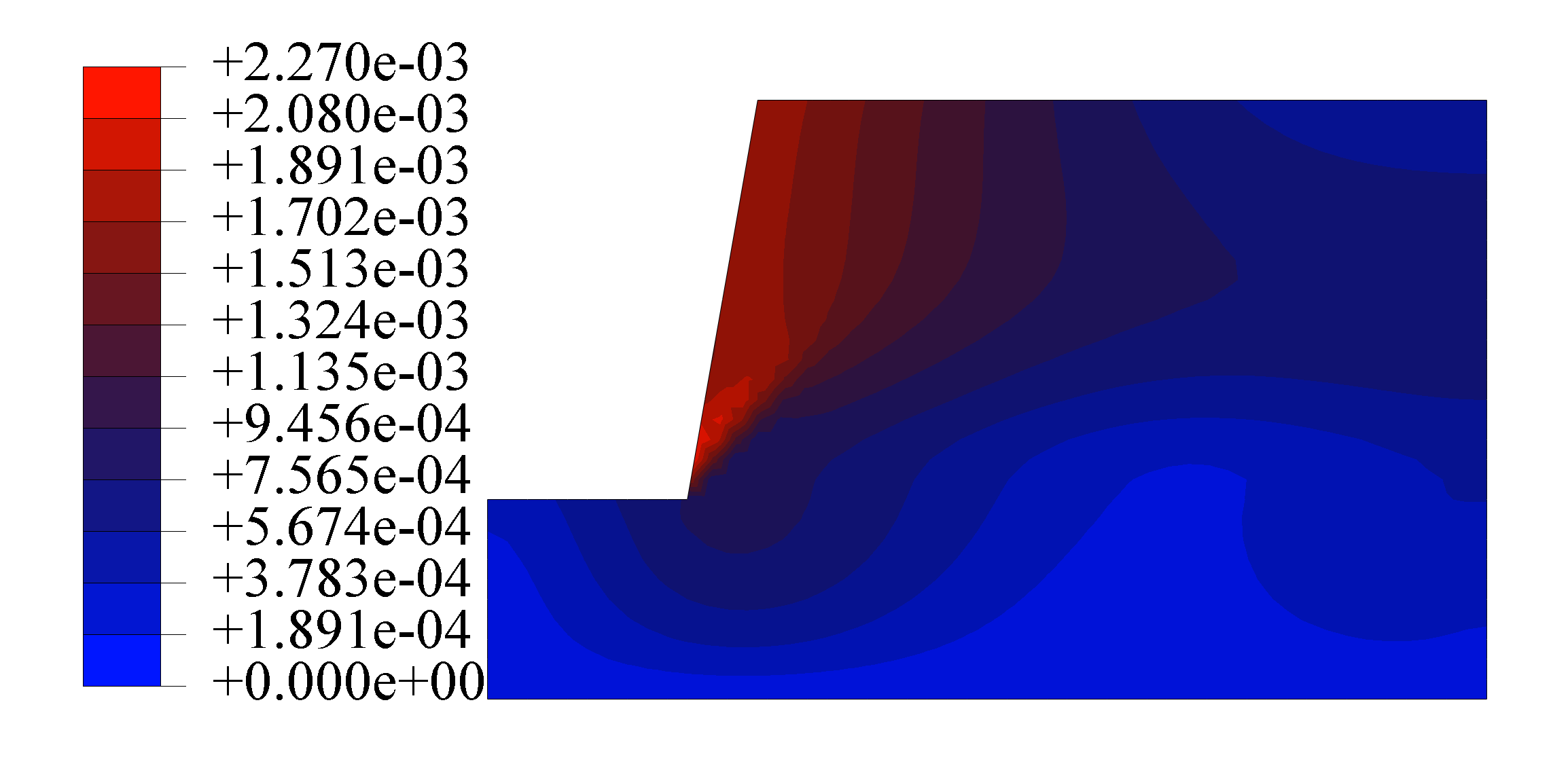

Supplement: Supplementary file 1 [file sensors-26-00421-s001.zip › Supplementary Materials/U80.png]

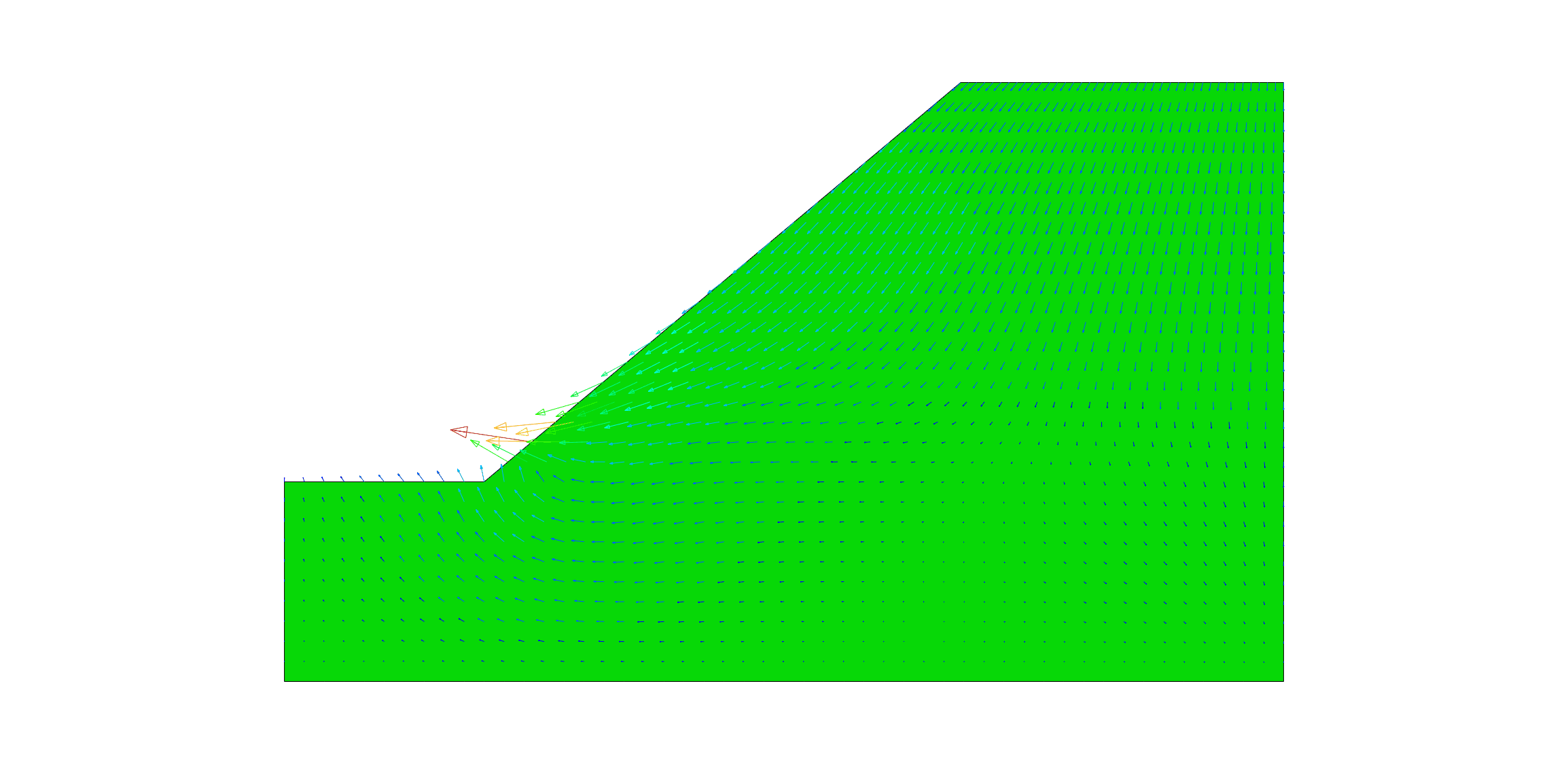

Supplement: Supplementary file 1 [file sensors-26-00421-s001.zip › Supplementary Materials/UH40.png]

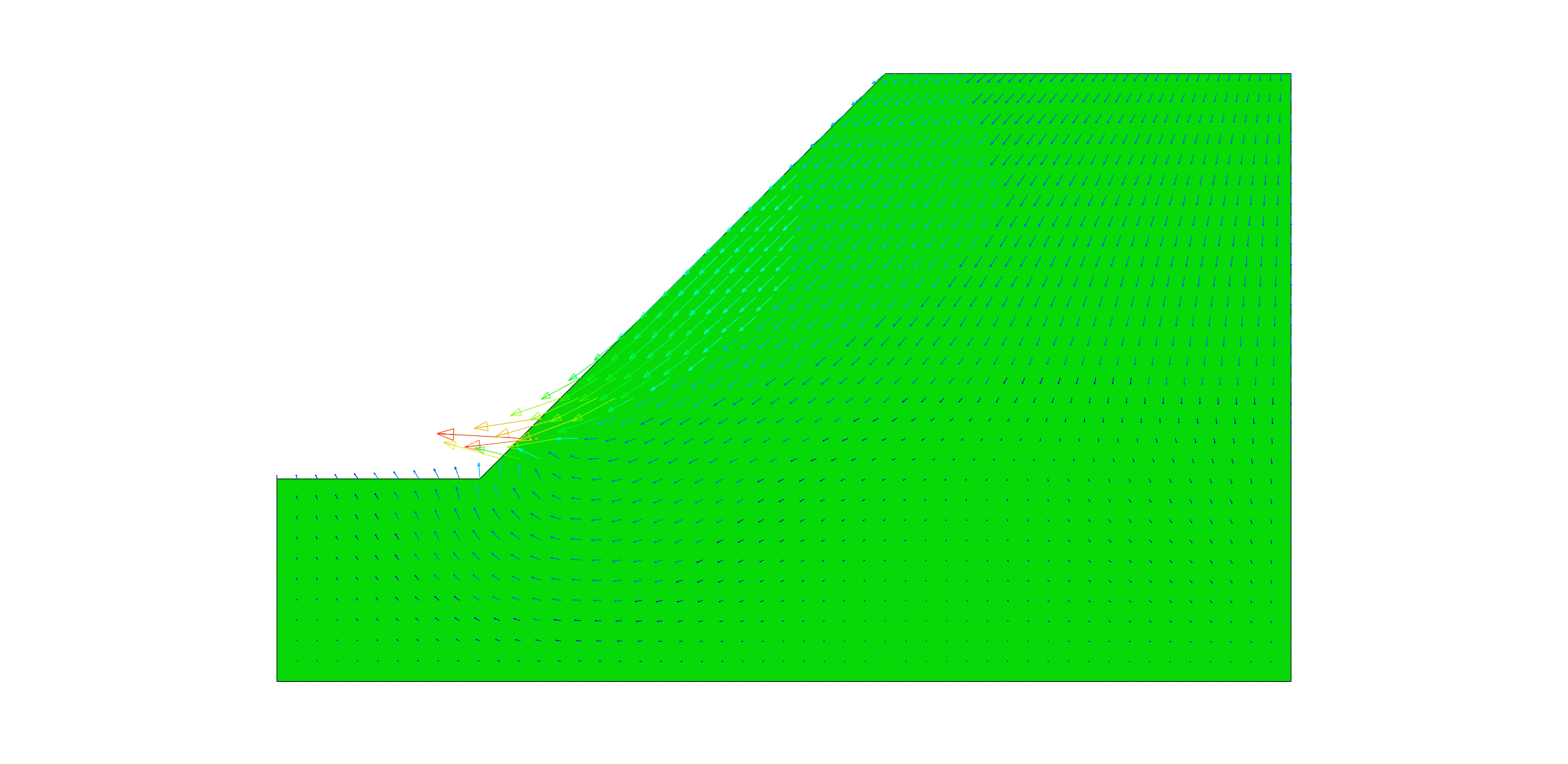

Supplement: Supplementary file 1 [file sensors-26-00421-s001.zip › Supplementary Materials/UH45.png]

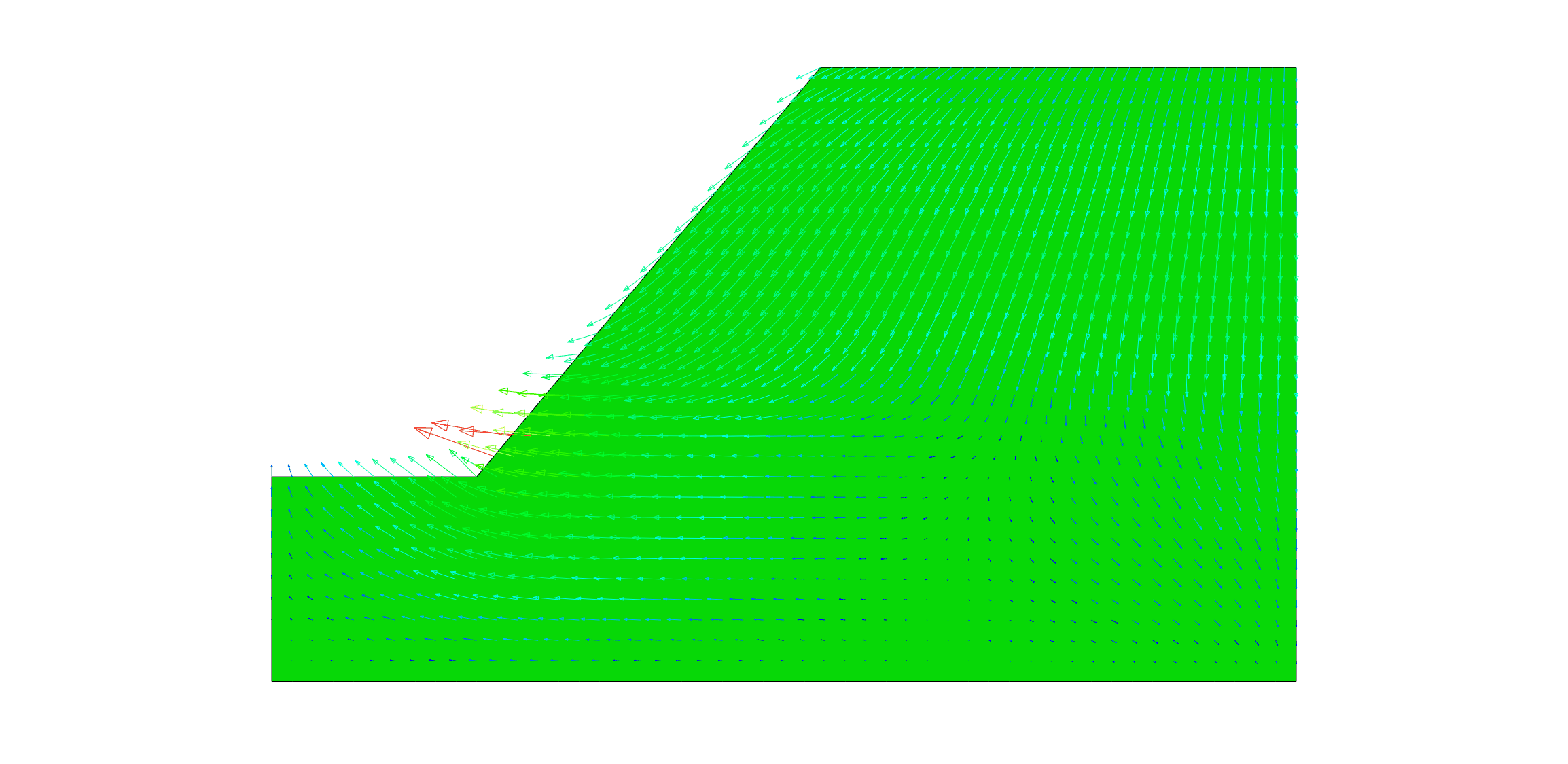

Supplement: Supplementary file 1 [file sensors-26-00421-s001.zip › Supplementary Materials/UH50.png]

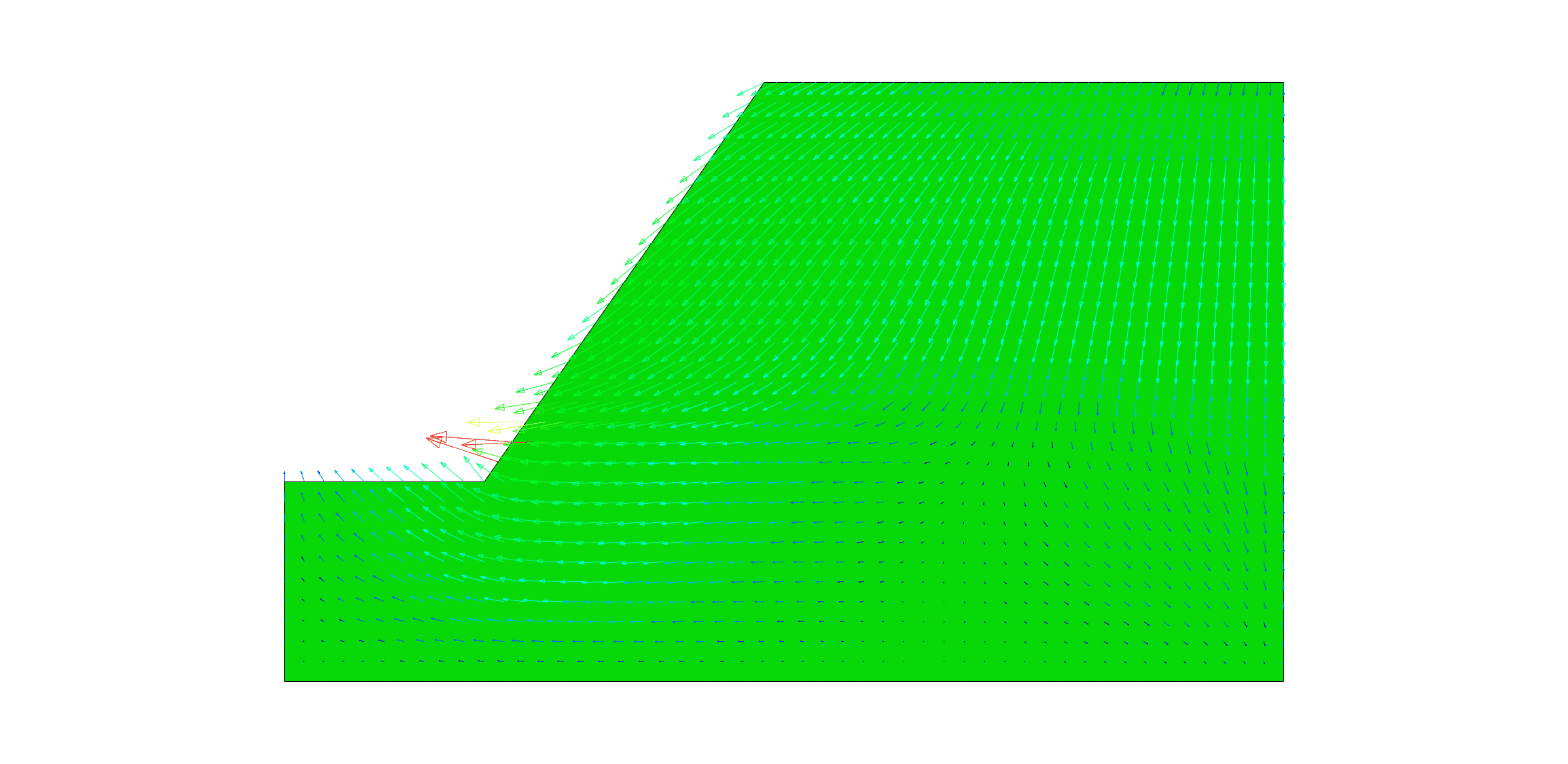

Supplement: Supplementary file 1 [file sensors-26-00421-s001.zip › Supplementary Materials/UH55.png]

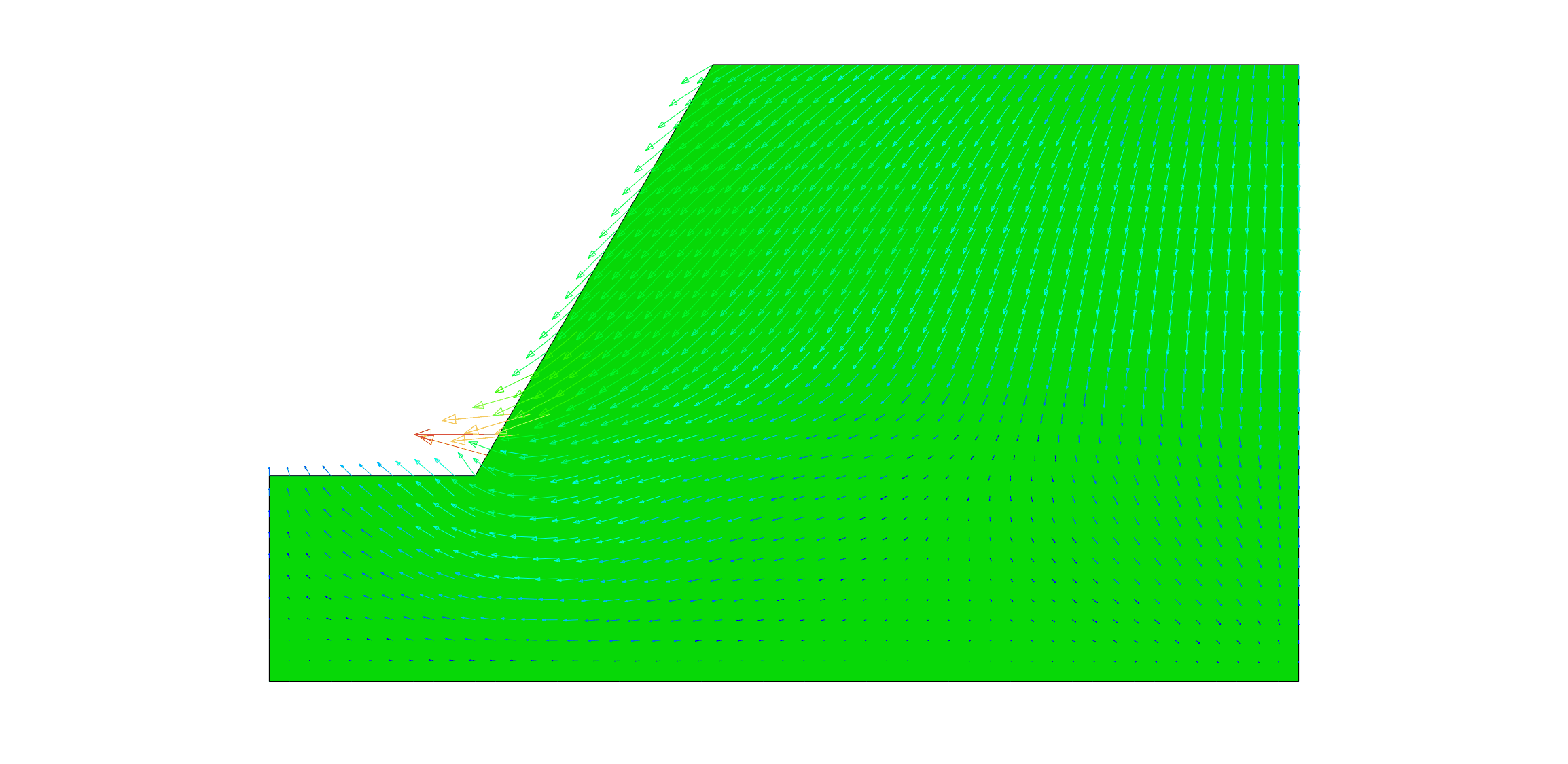

Supplement: Supplementary file 1 [file sensors-26-00421-s001.zip › Supplementary Materials/UH60.png]

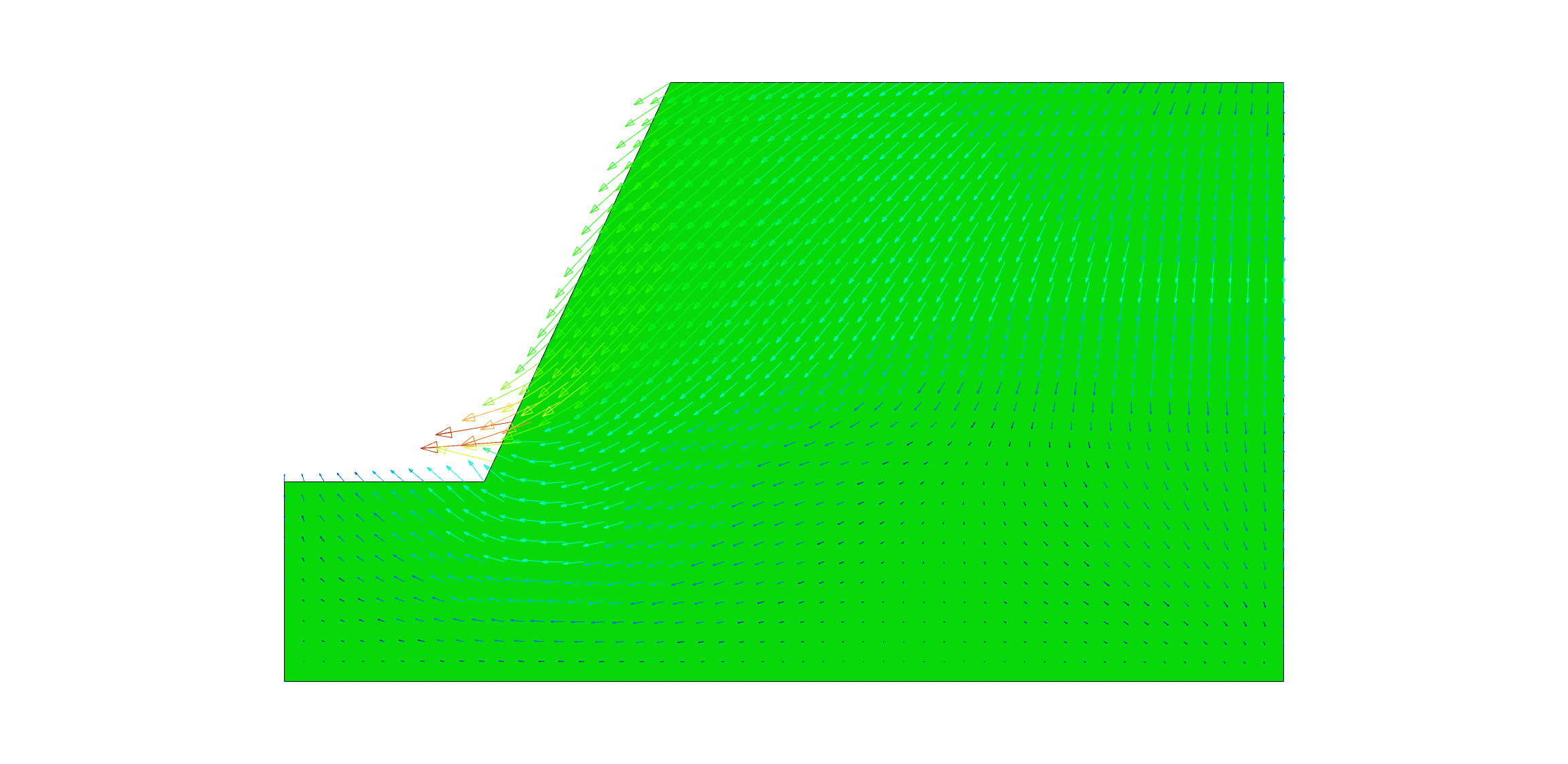

Supplement: Supplementary file 1 [file sensors-26-00421-s001.zip › Supplementary Materials/UH65.png]

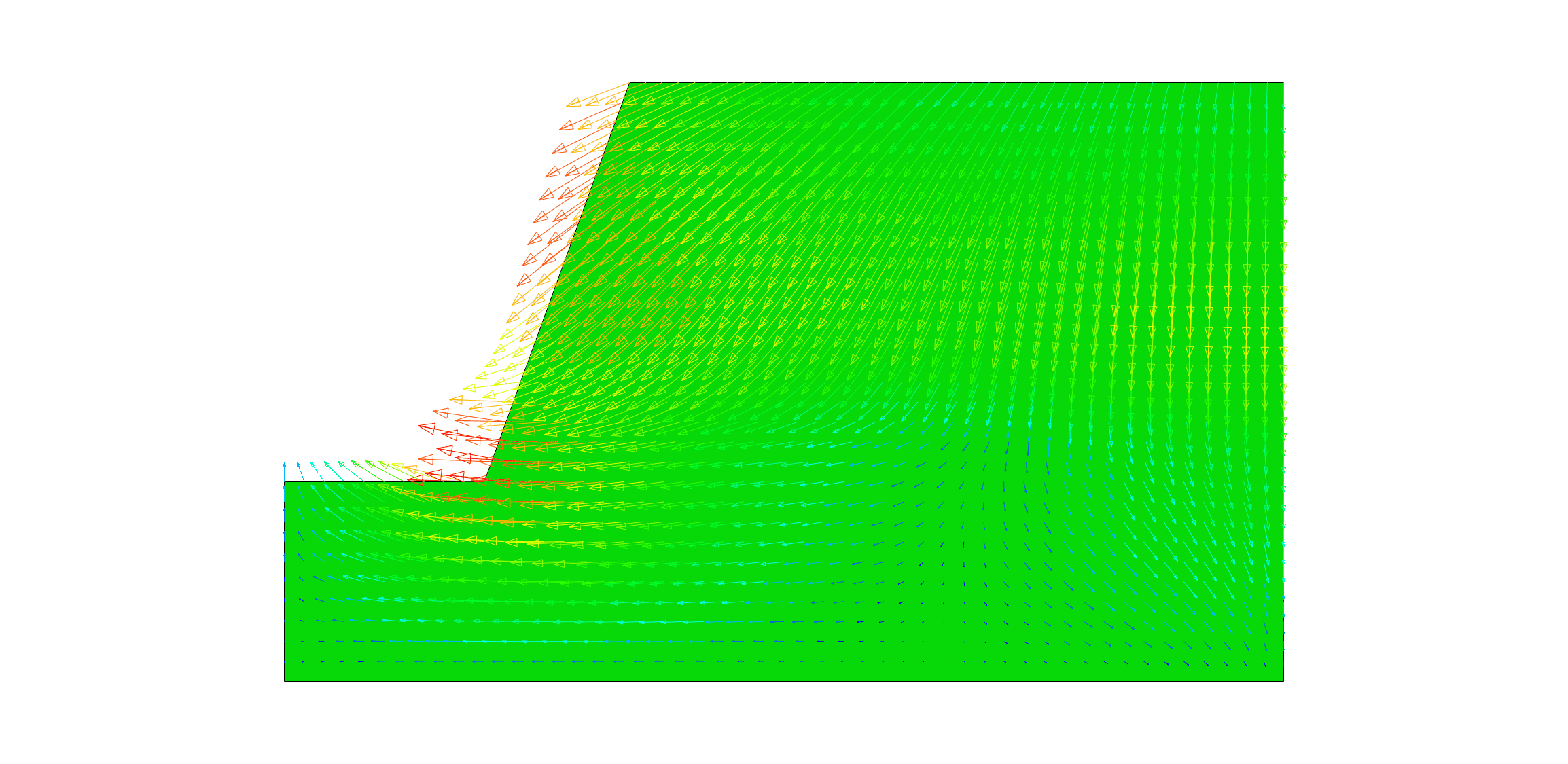

Supplement: Supplementary file 1 [file sensors-26-00421-s001.zip › Supplementary Materials/UH70.png]

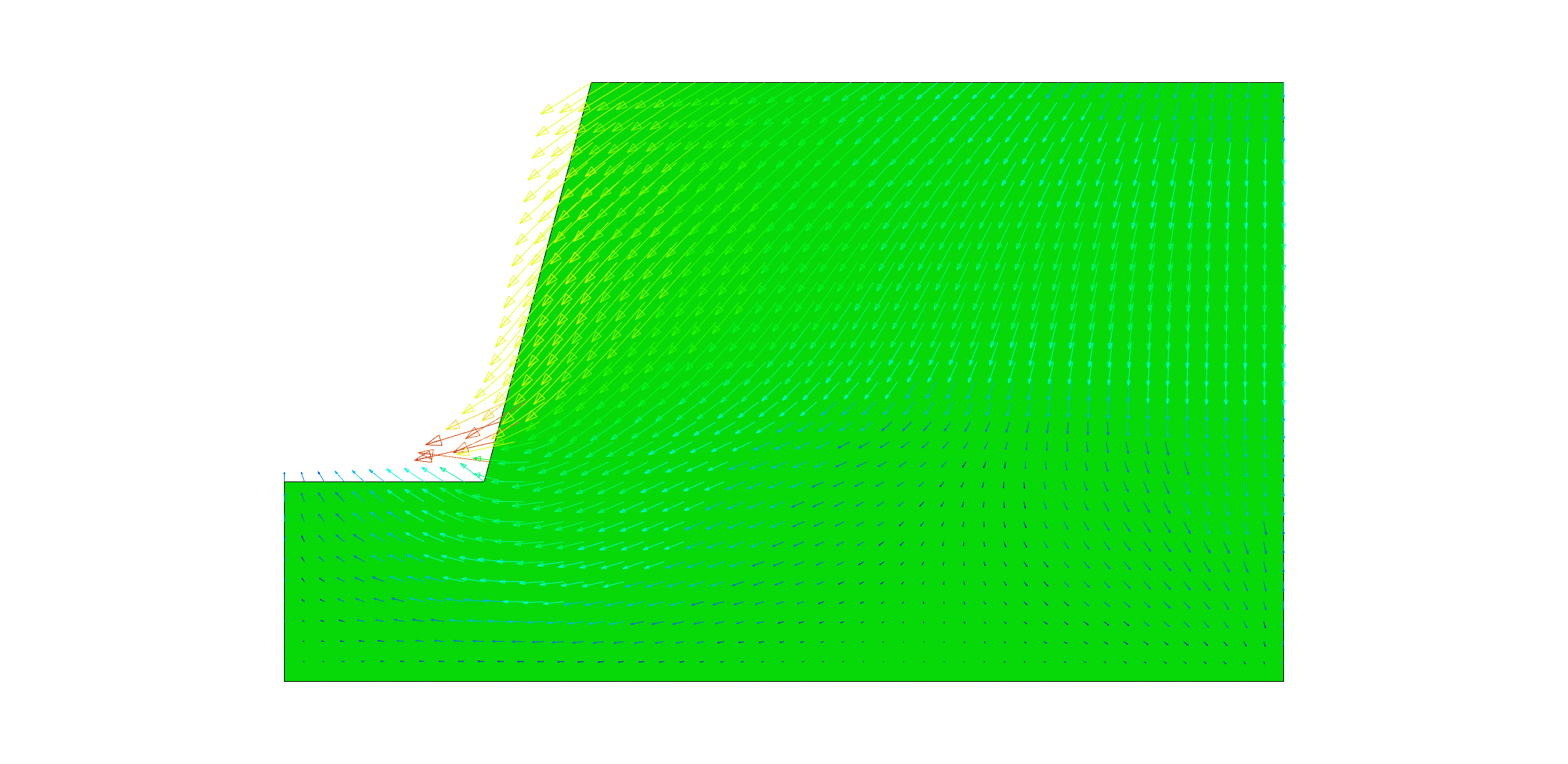

Supplement: Supplementary file 1 [file sensors-26-00421-s001.zip › Supplementary Materials/UH75.png]

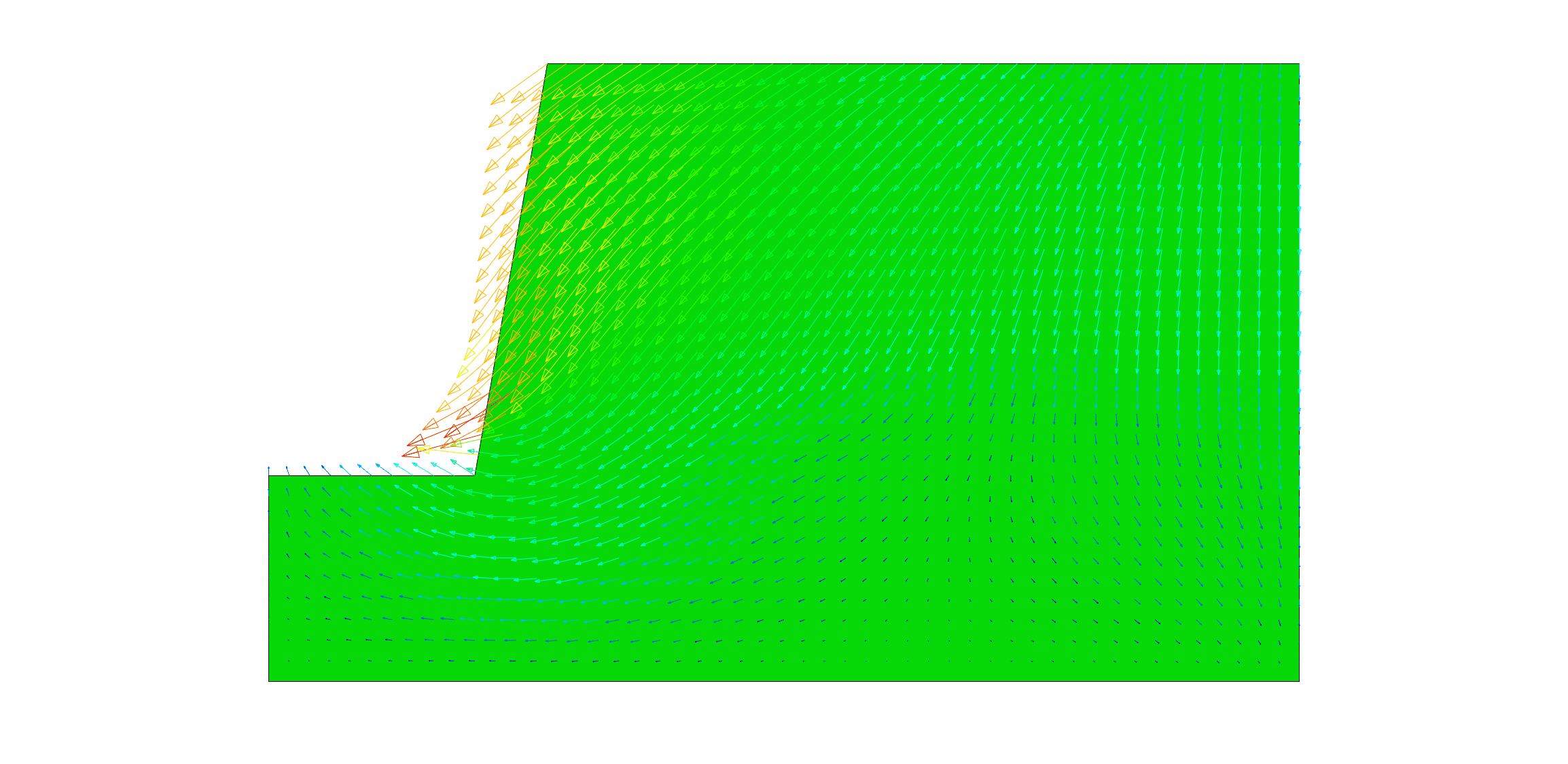

Supplement: Supplementary file 1 [file sensors-26-00421-s001.zip › Supplementary Materials/UH80.png]
