# Supplementary material for: Digital Twin Modeling for Landslide Risk Scenarios in Mountainous Regions
Source: Sensors (Basel). 2026 Jan 8;26(2):421. doi: 10.3390/s26020421 (PMC12845812; doi:10.3390/s26020421)
Supplement: Supplementary file 1 [file sensors-26-00421-s001.zip › Supplementary Materials/Instructions.doc]

(Nomenclature)por: Pore pressure.40-80: Slope gradient (in degrees). The same applies to the following.S11: Applied stress along the X-axis.S22: Stress component along the Y-axis.S33: Stress component along the Z-axis.SM: Paradigmatic stress under initial conditions.SMH: Paradigmatic stress after sustained rainfall.U: Displacement under initial conditions.UH: Displacement after sustained rainfall.

(Table Descriptions)Table: Saturation-dependent parameters for rainfall-induced landslidesThis table presents the saturation-dependent parameters used in the ABAQUS numerical simulation.Table: Simulated paradigmatic stress data pre- and post-failureThis table contains the paradigmatic stress data, simulated using ABAQUS, for the conditions before and after slope failure under intense rainfall.
